# Supplementary material for: Acid sphingomyelinase deactivation post-ischemia promotes brain angiogenesis and remodeling by small extracellular vesicles
Source: Basic Res Cardiol. 2022 Aug 29;117(1):43. doi: 10.1007/s00395-022-00950-7 (PMC9424180; doi:10.1007/s00395-022-00950-7)
Supplement: Supplementary file 1 — Supplementary file1 (PDF 4183 KB) [file 395_2022_950_MOESM1_ESM.pdf]

SUPPLEMENTARY FIGURES

Acid sphingomyelinase deactivation post-ischemia promotes brain angiogenesis and remodeling by small extracellular vesicles

Basic Research in Cardiology

Ayan Mohamud Yusuf,[1,2] Nina Hagemann,[1,2] Xiaoni Zhang,[1,2] Maria Zafar,[1,2] Tanja Hussner,[1,2] Carolin Bromkamp,[1,2] Carlotta Martiny,[1,2] Tobias Tertel,[3] Verena Börger,[3] Fabian Schumacher,[4,5,6] Fiorella A. Solari,[7] Mike Hasenberg,[8] Christoph Kleinschnitz,[1,2] Thorsten R. Doeppner,[1,2,9] Burkhard Kleuser,[5] Albert Sickmann,[7,10,11] Matthias Gunzer,[7,8] Bernd Giebel,[3] Richard Kolesnick,[12] Erich Gulbins,[4] Dirk M. Hermann[1,2]*

[1]Department of Neurology, [2]Center for Translational and Behavioral Neurosciences, [3]Institute of Transfusion Medicine and [4]Institute of Molecular Biology, University Hospital Essen, Essen, Germany, [5]Department of Toxicology, University of Potsdam, Nuthetal, Germany, [6]Institute of Pharmacy, Freie Universität Berlin, Berlin, Germany, [7]Leibniz-Institut für Analytische Wissenschaften-ISAS-e.V., Dortmund, Germany, [8]Institute of Immunology and Experimental Imaging, University Hospital Essen, Essen, Germany, [9]Department of Neurology, University Medicine Göttingen, Göttingen, Germany, [10]Medizinisches Proteom-Center (MPC), Ruhr University, Bochum, Germany, [11]Department of Chemistry, College of Physical Sciences, University of Aberdeen, Aberdeen, Scotland, U.K., [12]Memorial Sloan Kettering Cancer Center, New York, New York, U.S.A.; *corresponding author.

Correspondence: Prof. Dirk M. Hermann, MD, Department of Neurology, University Hospital Essen, Hufelandstr. 55, D-45122 Essen, Germany, E-Mail: dirk.hermann@uk-essen.de

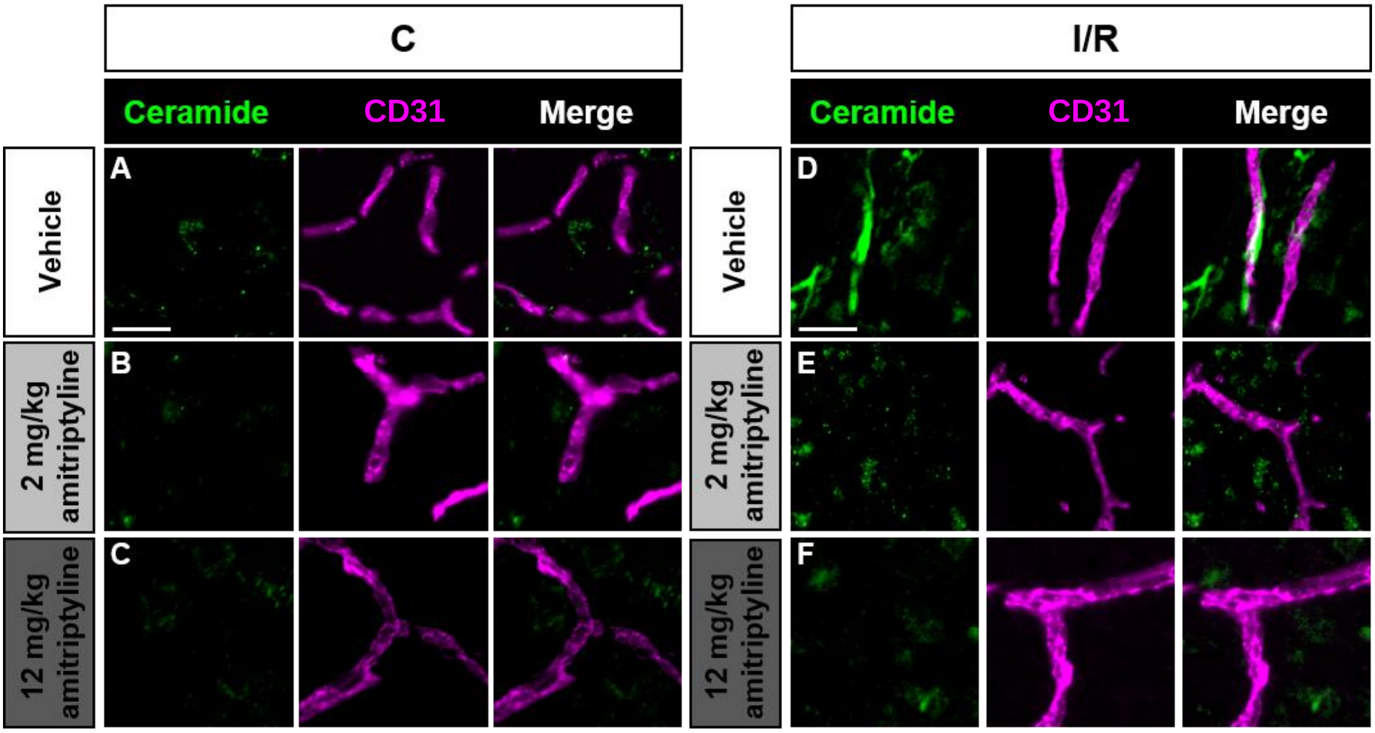

Supplementary Figure 1. Acid sphingomyelinase (Asm) product ceramide accumulates in cerebral microvessels after ischemia/ reperfusion (I/R) *in vivo*. Immunohistochemistry for ceramide in the contralateral non-ischemic control striatum (C) and reperfused ischemic striatum (I/R) of C57BL/6j mice exposed to transient middle cerebral artery occlusion (MCAO), which were intraperitoneally treated with vehicle or amitriptyline (2 or 12 mg/kg b.w., b.i.d.) immediately after I/R, followed by animal sacrifice after 24 hours. Scale bar, 50 µm. Data are representative for 3 independent studies.

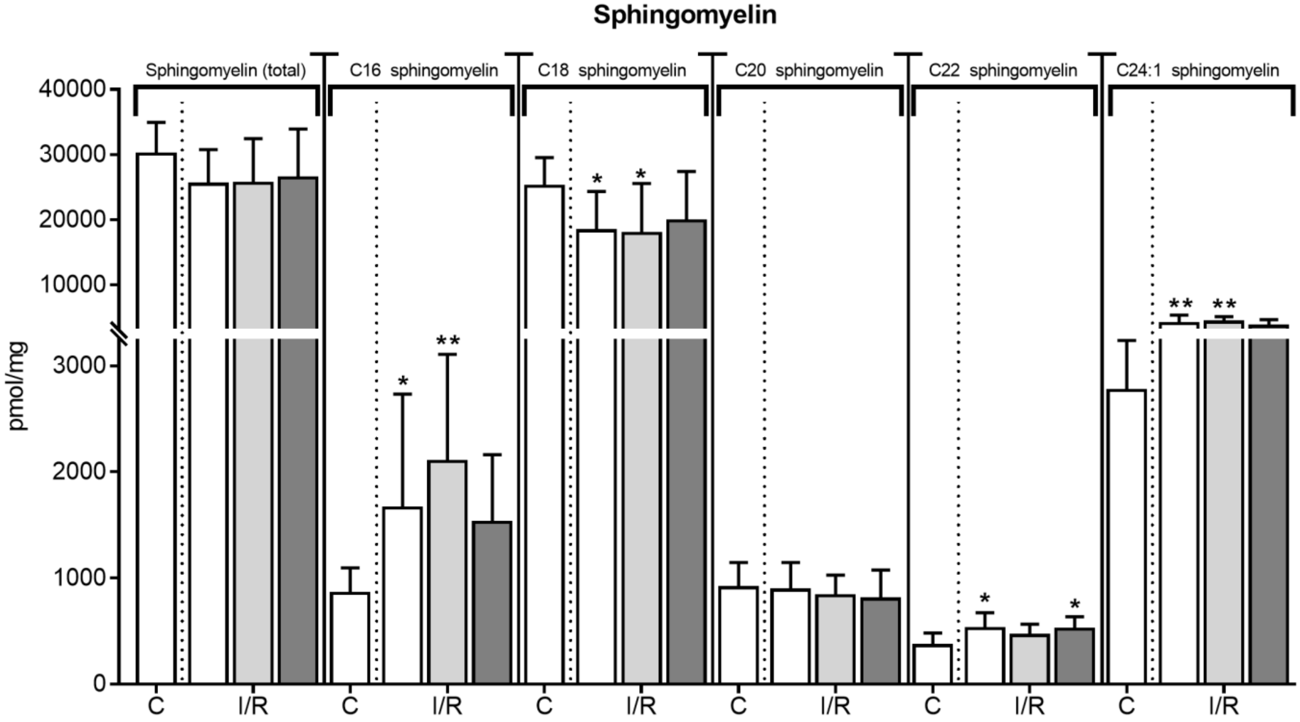

Supplementary Figure 2. Asm inhibitor amitriptyline does not change cerebral sphingomyelin levels after I/R *in vivo*. Total sphingomyelin, C16 sphingomyelin, C18 sphingomyelin, C20 sphingomyelin, C22 sphingomyelin and C24:1 sphingomyelin content in the contralateral non-ischemic control striatum (C) and reperfused ischemic striatum (I/R) measured by liquid chromatography tandem-mass spectrometry (LC-MS/MS) in C57BL/6j mice exposed to transient MCAO, which were intraperitoneally treated with vehicle or amitriptyline (2 or 12 mg/kg b.w., b.i.d.) starting 24 hours post-MCAO, followed by animal sacrifice after 14 days. Data are means ± SD values. *p≤0.05/**p≤0.01 compared with non-ischemic C (n=7-9 animals/group; analyzed by one-way ANOVA followed by LSD tests).

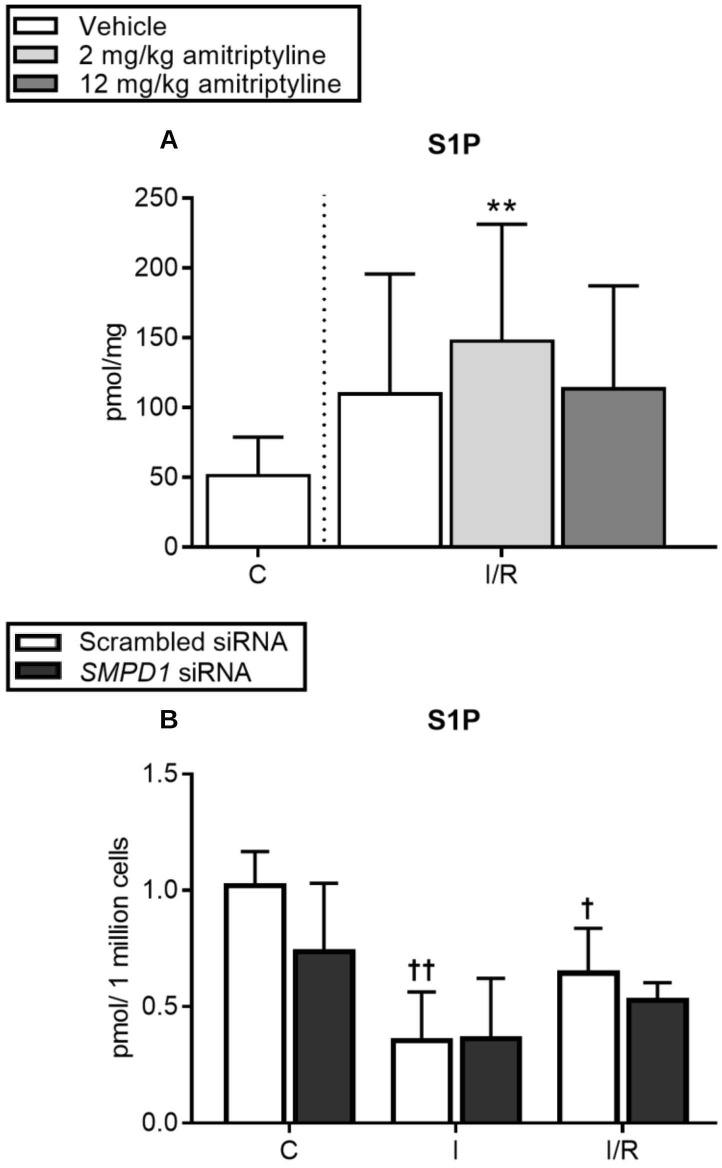

Supplementary Figure 3. S1P levels are not changed by Asm inhibitor amitriptyline after I/R *in vivo* and by *SMPD1* knockdown in cerebral microvascular endothelial cells *in vitro*. S1P content, measured by LC-MS/MS in **(A)** the reperfused ischemic striatum (labeled I/R) and contralateral non-ischemic striatum (labeled C), which were intraperitoneally treated with vehicle or amitriptyline (2 or 12 mg/kg b.w., b.i.d.) with 24 hours delay, followed by animal sacrifice after 14 days and in **(B)** hCMEC/D3 transfected with scrambled siRNA or *SMPD1* siRNA and exposed to non-ischemic control condition (C), OGD (that is, ischemia; I) or ischemia followed by reoxygenation/glucose re-supplementation (I/R). Data are means ± SD values. $^{**}p \leq 0.01$ compared with non-ischemic C (n=7-8 animals/group [in **(A)**] and $^{\dagger}p \leq 0.05$/$^{\dagger\dagger}p \leq 0.01$ compared with corresponding C (n=3 independent samples/ group [in **(B)**]; analyzed by two-way ANOVA followed by LSD tests).

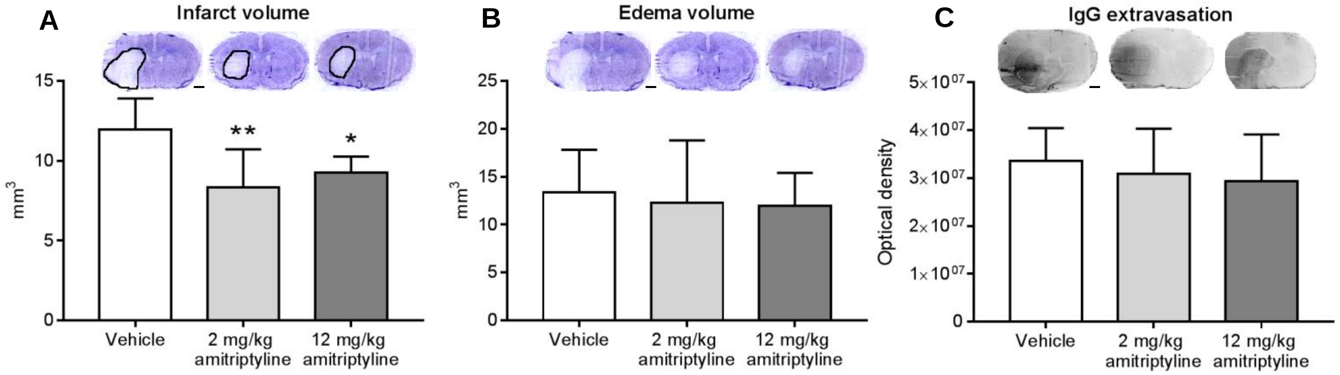

Supplementary Figure 4. Asm inhibitor amitriptyline reduces ischemic injury *in vivo*, when administered immediately after I/R. (A) Infarct volume and **(B)** brain edema evaluated on cresyl violet-stained brain sections, and **(C)** serum IgG extravasation as a marker of blood-brain barrier permeability by immunohistochemistry in the reperfused ischemic striatum of mice exposed to transient MCAO, which were intraperitoneally treated with vehicle or amitriptyline (2 or 12 mg/kg b.w., b.i.d.) immediately after MCAO, followed by animal sacrifice after 24 hours. Representative brain sections are also shown. Data are means ± SD values. *p≤0.05/**p≤0.01 compared with corresponding vehicle (n=7-8 animals/group; analyzed by one-way ANOVA followed by LSD tests). Scale bar, 1000 µm.

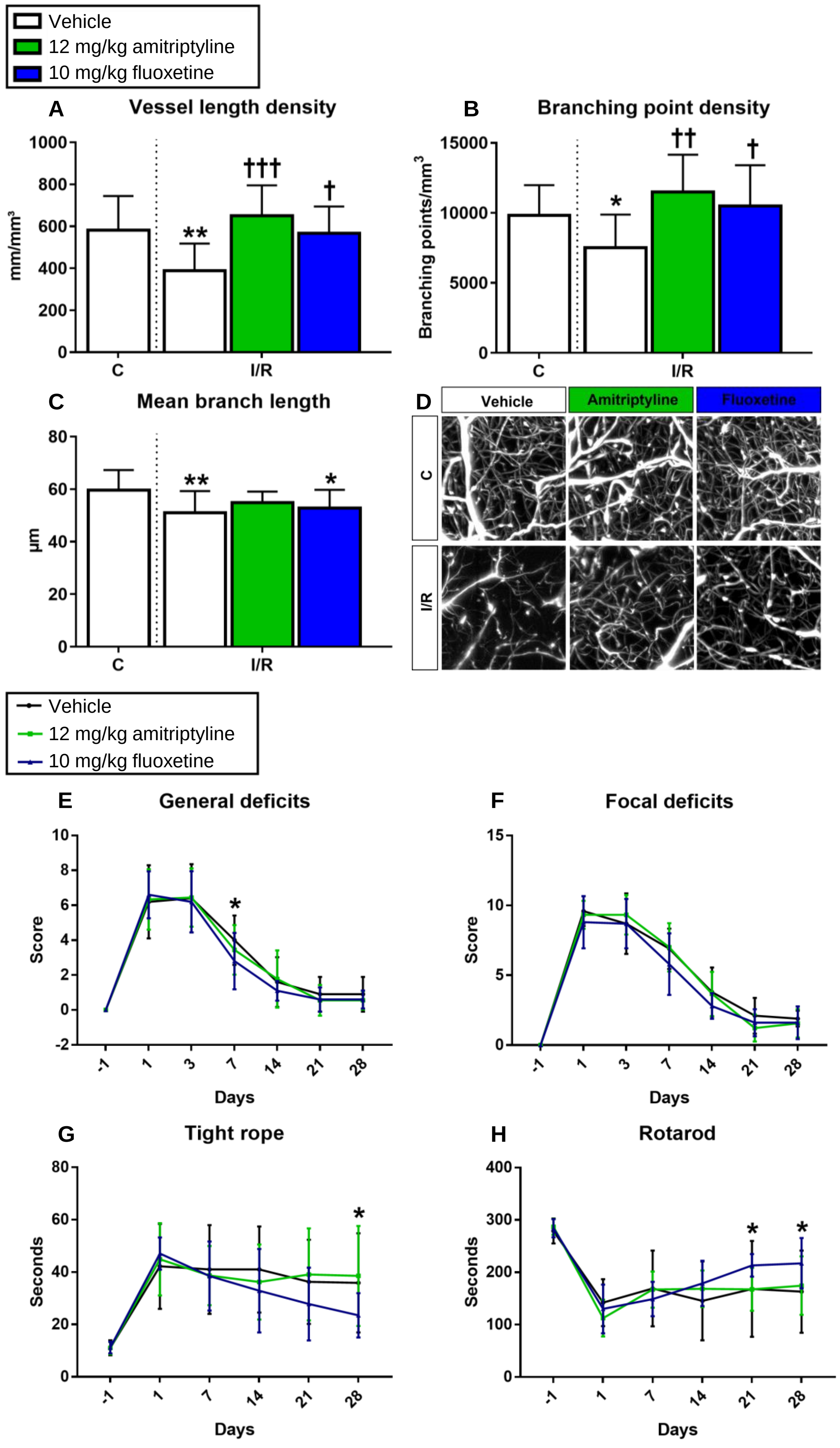

Supplementary Figure 5. Asm inhibitor fluoxetine induces angiogenesis and enhances motor-coordination performance after I/R. (A) Total microvascular length, **(B)** branching point density and **(C)** mean microvascular branch length, evaluated by LSM in I/R and C of wildtype MCAO mice treated with vehicle, amitriptyline (12 mg/kg b.w.) or fluoxetine (10 mg/kg b.w.). Maximum projection LSM images are illustrated in **(D). (E)** General and **(F)** focal neurological deficits examined by Clark's neurological score and **(G, H)** motor-coordination deficits evaluated by tight rope and Rotarod tests. Treatment was initiated 24 hours after MCAO and continued for 28 days. Data are means ± S.D. values. *p≤0.05/**p≤0.01 compared with non-ischemic C; †p≤0.05/††p≤0.01/†††p≤0.001 compared with corresponding vehicle [in **(A-C)**] or *p≤0.05 fluoxetine compared with vehicle [in **(E-H)**] (n=7-8 animals/group; analyzed by one-way ANOVA followed by LSD tests ([in **(A-C)**]; n=9-10 animals/group; analyzed by repeated measurement ANOVA followed by two-tailed t-tests [in **(E-H)**]).

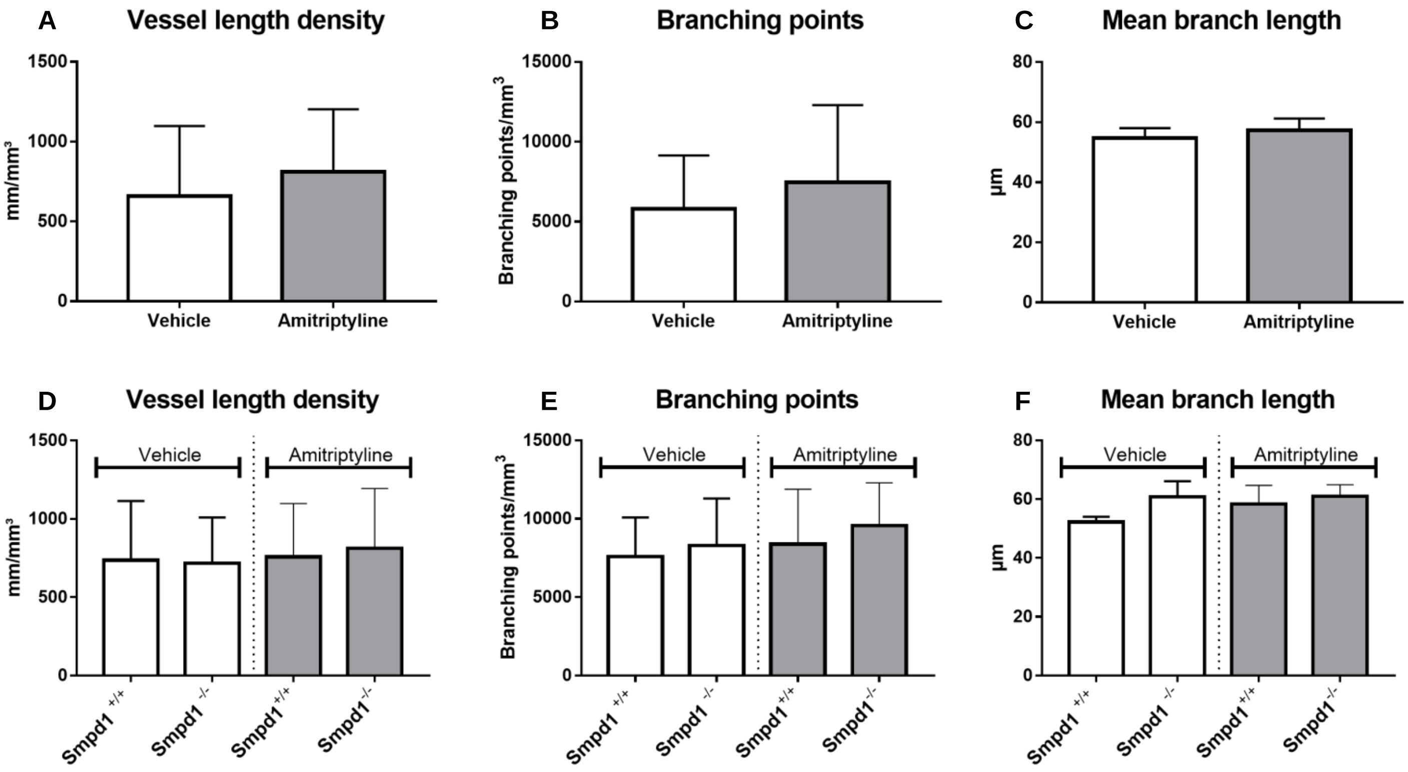

Supplementary Figure 6. Asm inhibitor amitriptyline does not modulate the cerebral microvessel characteristics in healthy mice. (A) Total microvascular length, **(B)** branching point density and **(C)** mean microvascular branch length, evaluated by LSM in healthy C57bl/6 mice treated with vehicle or amitriptyline (12 mg/kg b.w., b.i.d.) for 14 days. **(D)** Microvascular length, **(E)** branching point density and **(F)** mean branch length in *Smpd1*$^{+/+}$ (wildtype) and *Smpd1*$^{-/-}$ (that is, ASM-deficient) healthy mice treated with vehicle or amitriptyline (12 mg/kg b.w., b.i.d.) for 14 days. Data are means ± SD values (n=3-4 animals/group; analyzed by one-way ANOVA followed by LSD tests).

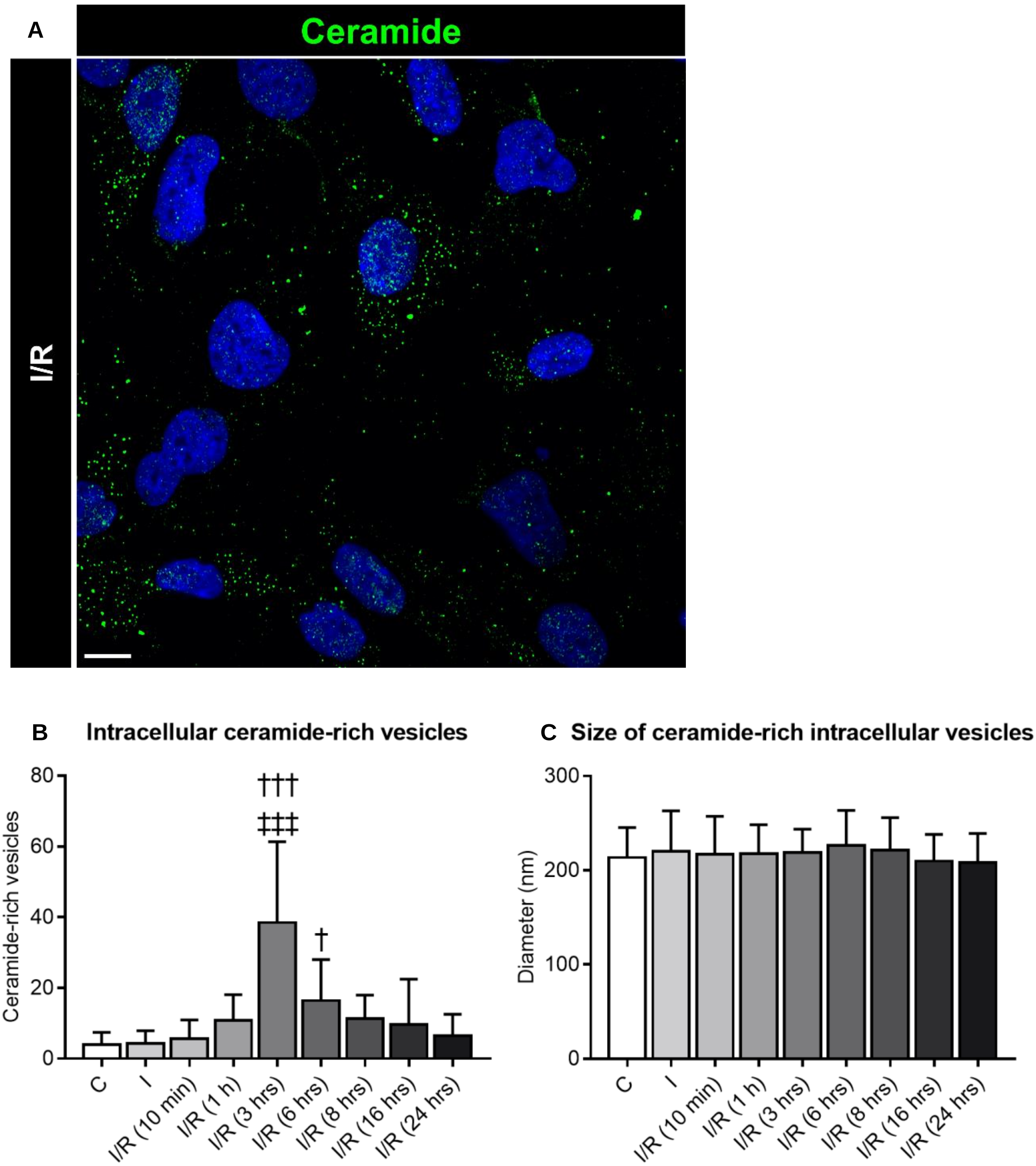

Supplementary Figure 7. I/R induces the intracellular formation of ceramide-rich vesicles in cerebral microvascular endothelial cells *in vitro*. (A) Immunohistochemistry for ceramide (in green) in hCMEC/D3 exposed to 24 hours OGD (that is, ischemia) followed by 3 hours reoxygenation/glucose re-supplementation (I/R). Scale bar, 10 µM. **(B)** Number and **(C)** size of intracellular ceramide-rich vesicles evaluated by Cell Profiler in hCMEC/D3 exposed to non-ischemic control condition (C), 24 hours ischemia (I) or 24 hours ischemia followed by different durations of reoxygenation/glucose re-supplementation (I/R). Data are means ± SD values. $^{\dagger}p \leq 0.05$/$^{\dagger\dagger\dagger}p \leq 0.001$ compared with corresponding C; $^{\ddagger\ddagger\ddagger}p \leq 0.001$ compared with corresponding I (n=3-8 independent samples/group [in **(B)**]; n=3-4 independent samples/group [in **(C)**]; analyzed by one-way ANOVA followed by LSD tests).

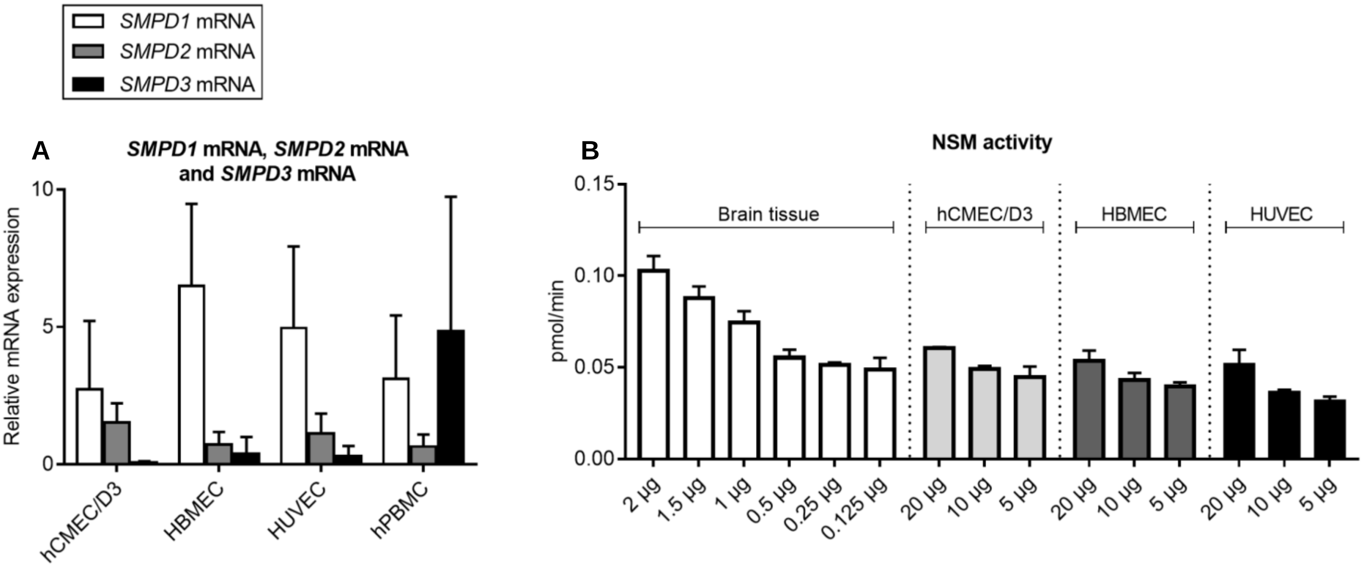

Supplementary Figure 8. ASM is the predominant sphingomyelinase in endothelial cells. (A) *SMPD1*, *SMPD2* and *SMPD3* mRNA level evaluated by polymerase-chain reaction (PCR) in hCMEC/D3, primary human brain microvascular endothelial cells (HBMEC), human umbilical vein endothelial cells (HUVEC) and human peripheral blood mononuclear cells (hPBMC) and **(B)** magnesium dependent NSM activity examined using BODIPY-labeled sphingomyelin as substrate in whole brain tissue, hCMEC/D3, HBMEC and HUVEC. Data are means ± SD values (n=3 independent samples/group).

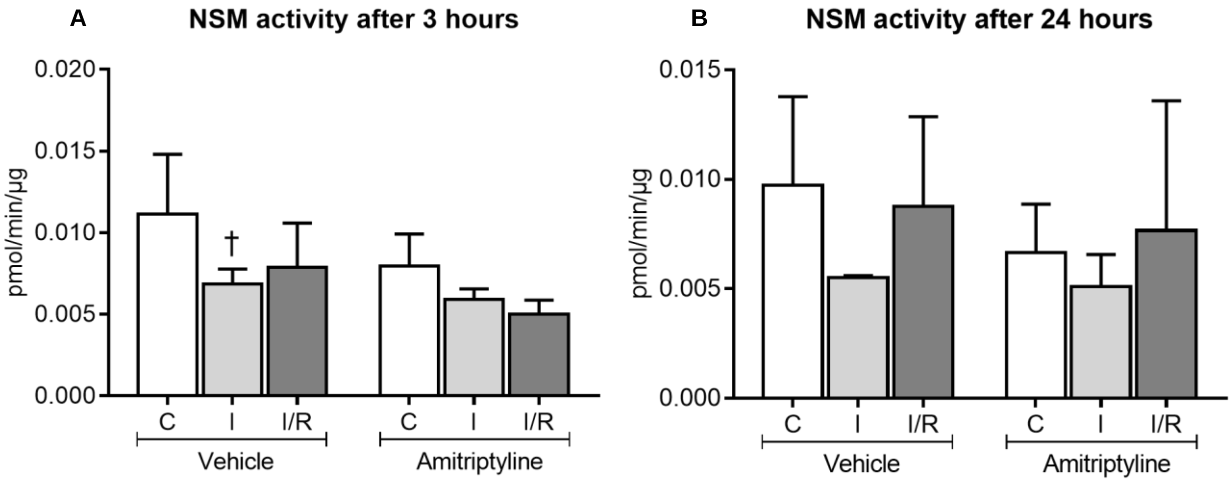

Supplementary Figure 9. Amitriptyline does not affect NSM activity in cerebral microvascular endothelial cells *in vitro*. Magnesium dependent NSM activity examined in hCMEC/D3 exposed to **(A)** non-ischemic control condition (C), 3 hours OGD (that is, ischemia; I), or 24 hours ischemia followed by 3 hours reoxygenation/glucose re-supplementation (I/R), or to **(B)** non-ischemic C, 24 hours ischemia (I) or 24 hours ischemia followed by 24 hours reoxygenation/glucose re-supplementation (I/R), which were treated with vehicle or amitriptyline (50 µM) during 3 hours (in **(A)**) or 24 hours (in **(B)**). Data are means ± SD values (n=3 independent samples/group). †$p \leq 0.05$ compared with corresponding C (n=3 independent samples/group; analyzed by one-way ANOVA followed by LSD tests).

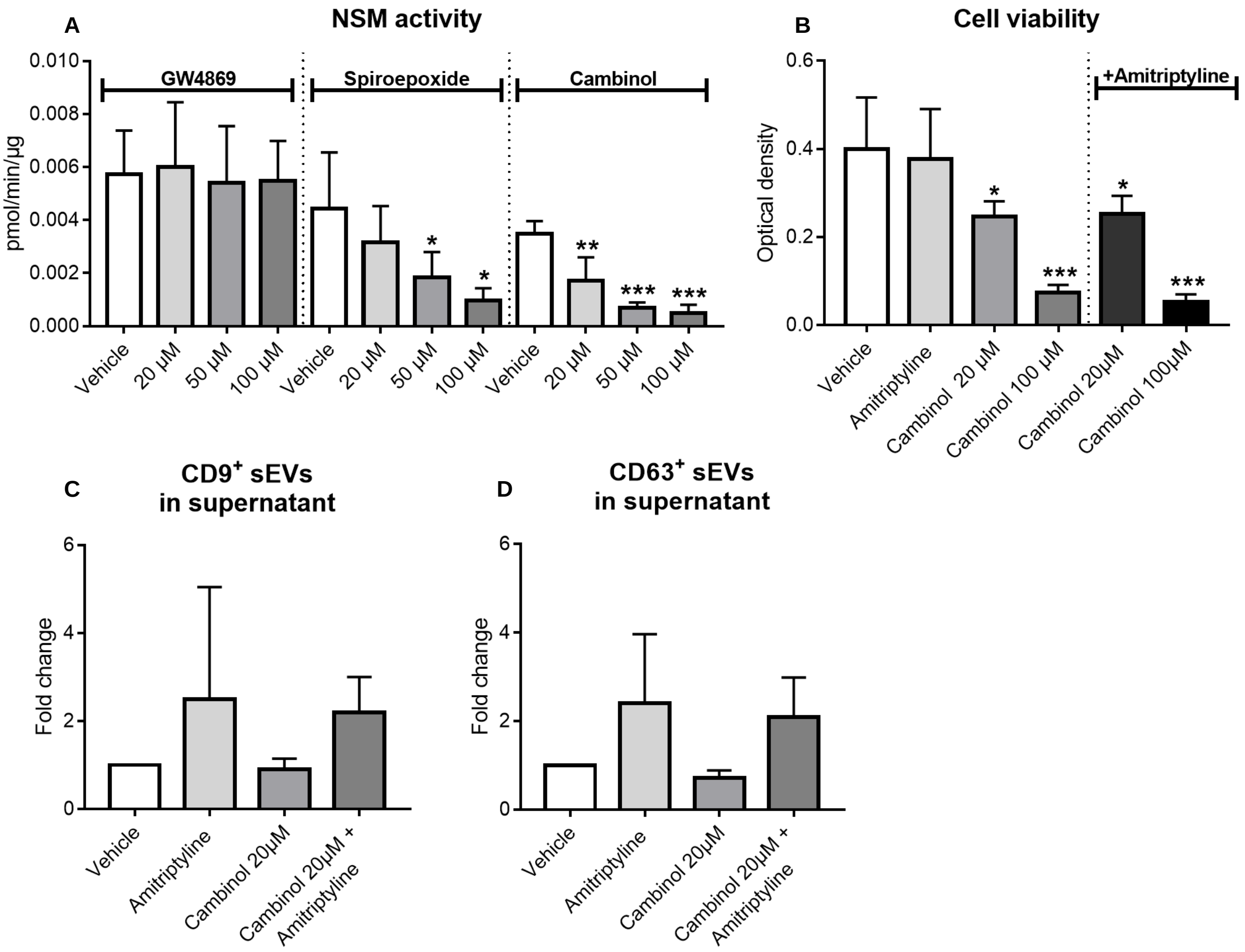

Supplementary Figure 10. NSM inhibition does not influence sEV release in cerebral microvascular endothelial cells *in vitro*. (A) Magnesium dependent NSM activity examined in hCMEC/D3 treated with indicated concentrations of GW4869, spiroepoxide or cambinol using BODIPY-labeled sphingomyelin as substrate. **(B)** Endothelial viability assessed by the 3-(4,5-dimethylthiazol-2-yl)-2,5-diphenyltetrazolium bromide (MTT) assay in hCMEC/D3 treated with vehicle, amitriptyline (50 µM) and/ or cambinol (20 µM or 100 µM). Concentration of **(C)** $CD9^{+}$ and **(D)** $CD63^{+}$ sEVs in the supernatant of hCMEC/D3 treated with vehicle, amitriptyline (50 µM) and/ or cambinol (20 µM). sEV concentration was evaluated by AMNIS flow cytometry. Data are means ± SD values. *p≤0.05/**p≤0.01/***p≤0.001 compared with vehicle (n=3 independent samples/group [in **(A, B)**]; n=3-7 independent samples/group [in **(C, D)**]; analyzed by one-way or two-way ANOVA followed by LSD tests [in **(A, B)**] or two-tailed paired t tests [in **(C, D)**]).

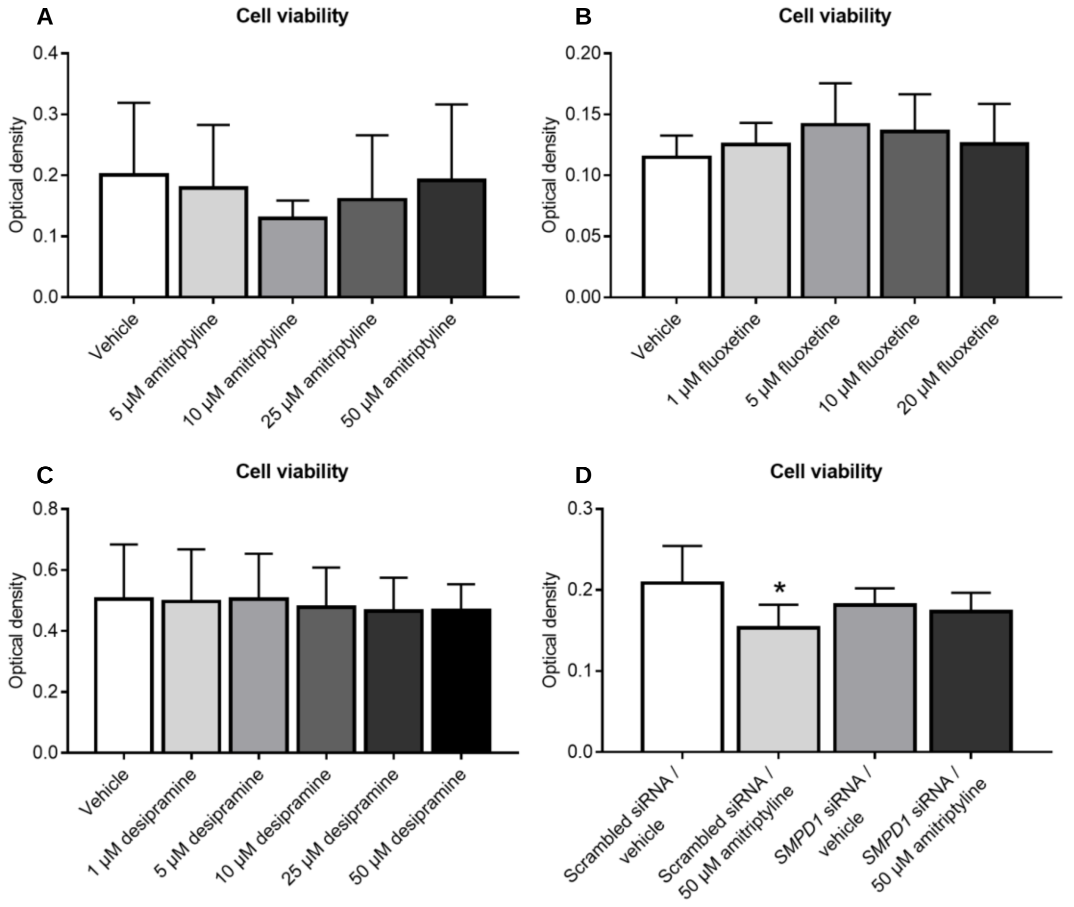

Supplementary Figure 11. ASM inhibitors do not influence the survival of human cerebral microvascular endothelial cells *in vitro*. Endothelial viability assessed by MTT assay in non-ischemic hCMEC/D3 treated with **(A)** vehicle or amitriptyline at defined doses (0-50 µM), **(B)** vehicle or fluoxetine at defined doses (0-20 µM) or **(C)** vehicle or desipramine at defined doses (0-50 µM), or in **(D)** in non-ischemic hCMEC/D3 transfected with scrambled siRNA or *SMPD1* siRNA that were treated with vehicle or amitriptyline (50 µM). Data are means ± SD values. *p≤0.05 compared with scrambled siRNA/ vehicle (n=3 independent samples/group [in **(A, B)**]; n=4 independent samples/group [in **(C, D)**]; analyzed by one-way ANOVA followed by LSD tests).

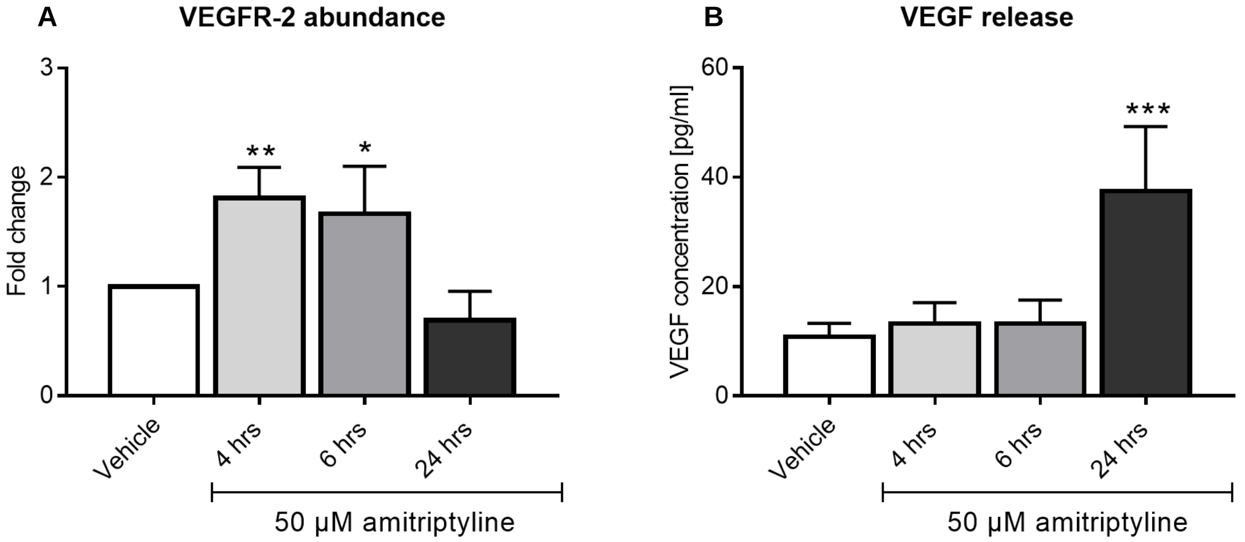

Supplementary Figure 12. ASM inhibitor amitriptyline increases VEGFR2 abundance and VEGF secretion by mouse cerebral microvascular endothelial cells. (A) VEGFR2 abundance evaluated by Western blot and **(B)** VEGF concentration in supernatant evaluated by enzyme-linked immunosorbent assay (ELISA) of non-ischemic mouse brain endothelial cells belonging to the cell line bEND5, which were treated with vehicle or amitriptyline for defined exposure times (0-24 hours) at a 50 µM concentration. Data are means ± SD values. *p≤0.05/**p≤0.01/***p≤0.001 compared with corresponding vehicle (n=4 independent samples/group [in **(A-B)**]; analyzed by one-way ANOVA followed by two-tailed paired t-tests [in **(A)**] or LSD tests [in (**B**)]).

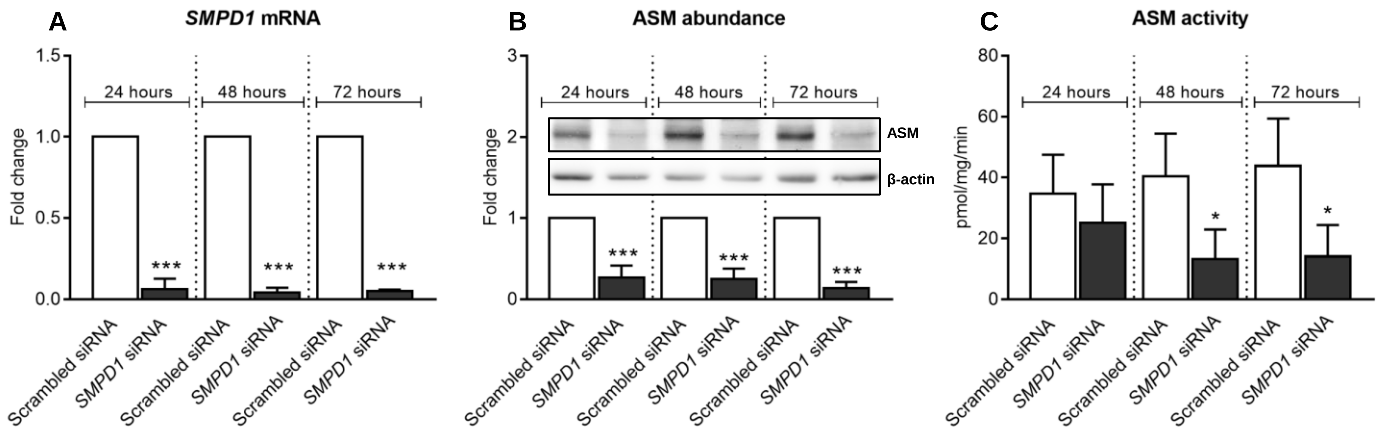

Supplementary Figure 13. *SMPD1* mRNA level, ASM abundance and ASM activity are effectively downregulated by small-interfering RNA (siRNA) *in vitro*. (A) *SMPD1* mRNA expression, evaluated by polymerase-chain reaction (PCR), **(B)** ASM abundance, determined by Western blot and **(C)** ASM activity, examined using BODIPY-labeled sphingomyelin as substrate, in hCMEC/D3 transfected with scrambled siRNA and *SMPD1* siRNA for 24-72 hours. Data are means ± SD values. *p≤0.05/***p≤0.001 compared with scrambled siRNA (n=3 independent samples/ group [in **(A)**]; n=4 independent samples/ group [in **(B)**]; n=3-6 independent samples/ group [in **(C)**]; analyzed by two-tailed paired [in **(A, B)**] or unpaired [in **(C)**] t tests).

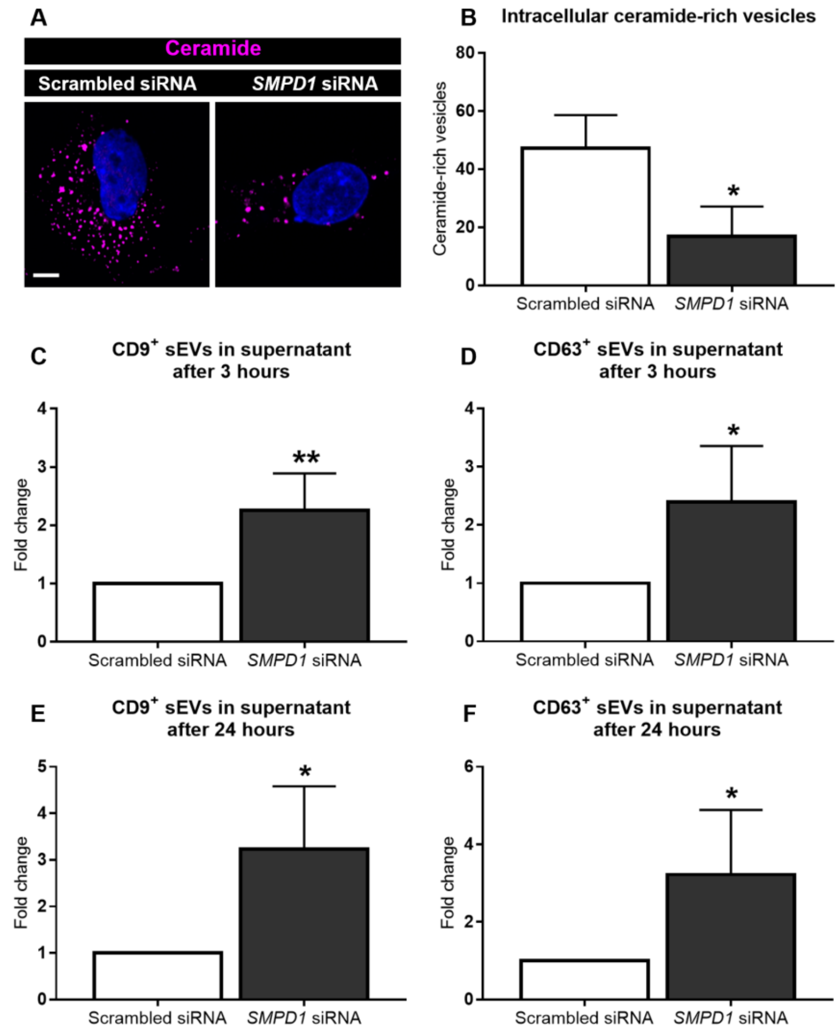

Supplementary Figure 14. ***SMPD1*** **knockdown reduces the intracellular accumulation of ceramide-rich vesicles after I/R** ***in vitro*** **and promotes the extracellular release of vesicles with immunofluorescence exosome characteristics. (A)** Immunohistochemistry for ceramide (in magenta) in hCMEC/D3 exposed to 24 hours OGD (that is, ischemia) followed by 3 hours reoxygenation/glucose re-supplementation, which were transfected with scrambled siRNA (used as control) or *SMPD1* siRNA. Nuclei were counterstained with Hoechst 33342 (in blue). Note that the number of intracellular ceramide-rich vesicles is reduced by *SMPD1* knockdown. Shown is a representative result from 3 independent studies. The density of vesicles evaluated by Cell Profiler is shown in **(B)**. Particle concentration of **(C, E)** $CD9^+$ and **(D, F)** $CD63^+$ sEVs in the supernatant of hCMEC/D3 exposed to 24 hours ischemia followed by 3 hours reperfusion (in **(C, D)**) or 24 hours reperfusion (in **(E, F)**), which were transfected with scrambled siRNA (used as control) or *SMPD1* siRNA. The particle concentration was evaluated by AMNIS image flow cytometry. Note that the number of $CD9^+$ and $CD63^+$ sEVs is increased by *SMPD1* knockdown. Data are means ± SD values. *p≤0.05/**p≤0.01 compared with corresponding scrambled siRNA (n=3 independent samples/ group [in **(B)**]; n=4 independent samples/ group [in **(C-F)**]; analyzed by two-tailed unpaired [in **(B)**] or paired [in **(C-F)**] t tests). Scale bar, 10 µm (in **(A)**).

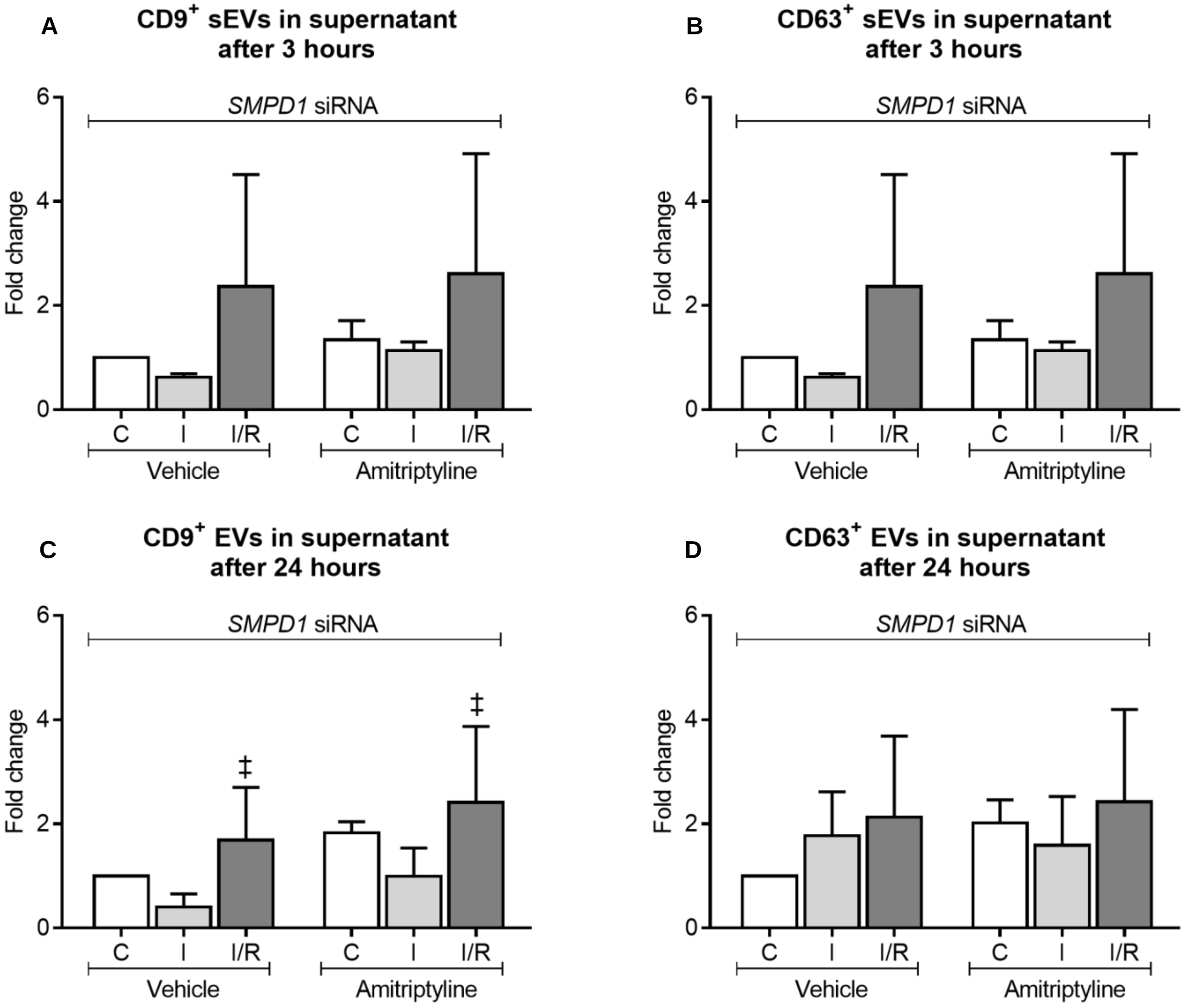

Supplementary Figure 15. ASM inhibitor amitriptyline does not modify sEV release after *SMPD1* knockdown in cerebral microvascular endothelial cells *in vitro*. Concentration of **(A, C)** CD9^{+} and **(B, D)** CD63^{+} sEVs in the supernatant of hCMEC/D3 exposed to 3 hours control condition (C), 3 hours OGD (that is, ischemia, I) or 24 hours/3 hours ischemia followed by reoxygenation/glucose re-supplementation (I/R) (in **(A, B)**) or 24 hours C, 24 hours I or 24 hours/24 hours I/R (in **(C, D)**) and treated with vehicle or amitriptyline (50 µM) after siRNA-mediated *SMPD1* knockdown. sEV concentration was evaluated by AMNIS flow cytometry. Data are means ± SD values. **‡**p≤0.05 compared with corresponding I (n=4 independent samples/group; analyzed by two-way ANOVA followed by two-tailed paired t tests).

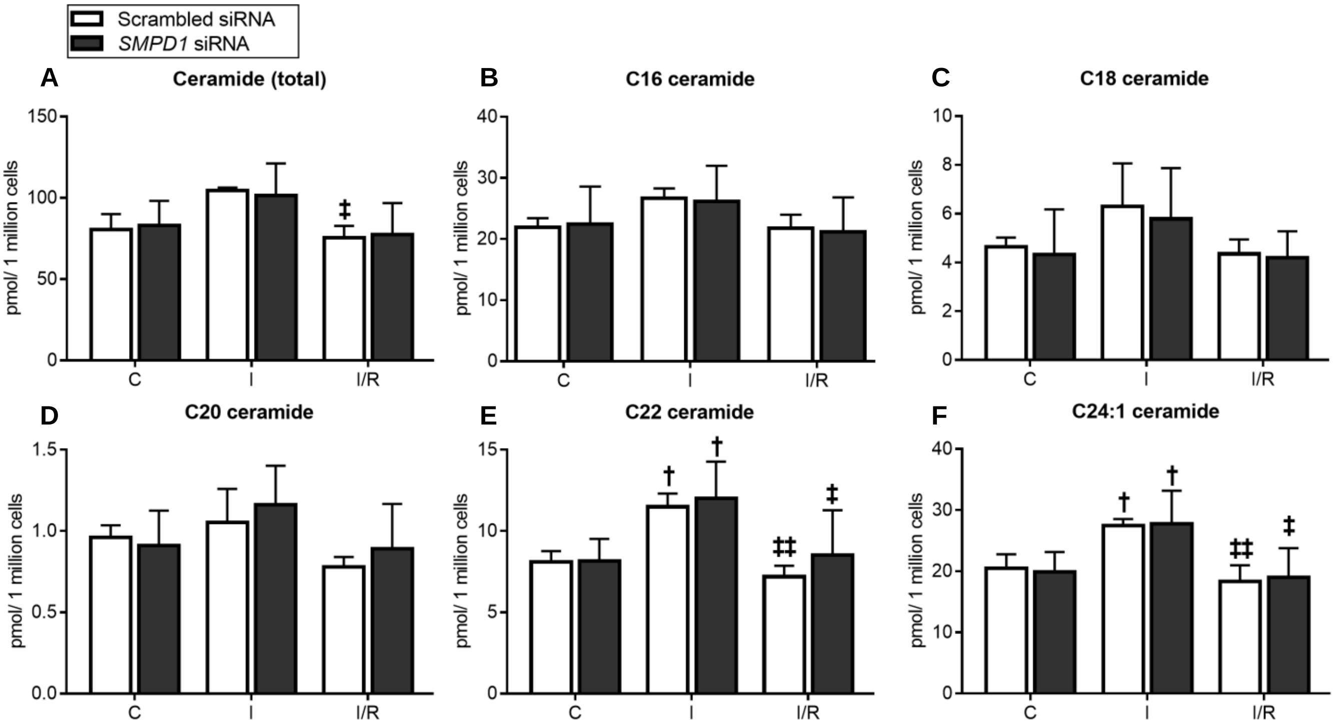

Supplementary Figure 16. *SMPD1* knockdown does not change overall ceramide levels in cerebral microvascular endothelial cells *in vitro*. (A) Total ceramide, **(B)** C16 ceramide, **(C)** C18 ceramide, **(D)** C20 ceramide, **(E)** C22 ceramide and **(F)** C24:1 ceramide content of hCMEC/D3 exposed to non-ischemic control condition (C), OGD (that is, ischemia; I) or ischemia followed by reoxygenation/glucose re-supplementation (I/R), which were transfected with scrambled siRNA (used as control) or *SMPD1* siRNA. Ceramide levels were measured by LC-MS/MS. Data are means ± SD values. $^{\dagger}p \leq 0.05$ compared with corresponding C; $^{\ddagger}p \leq 0.05$/$^{\ddagger\ddagger}p \leq 0.001$ compared with corresponding I (n=3 independent samples/ group [in **(A-F)**]; analyzed by two-way ANOVA followed by LSD tests).

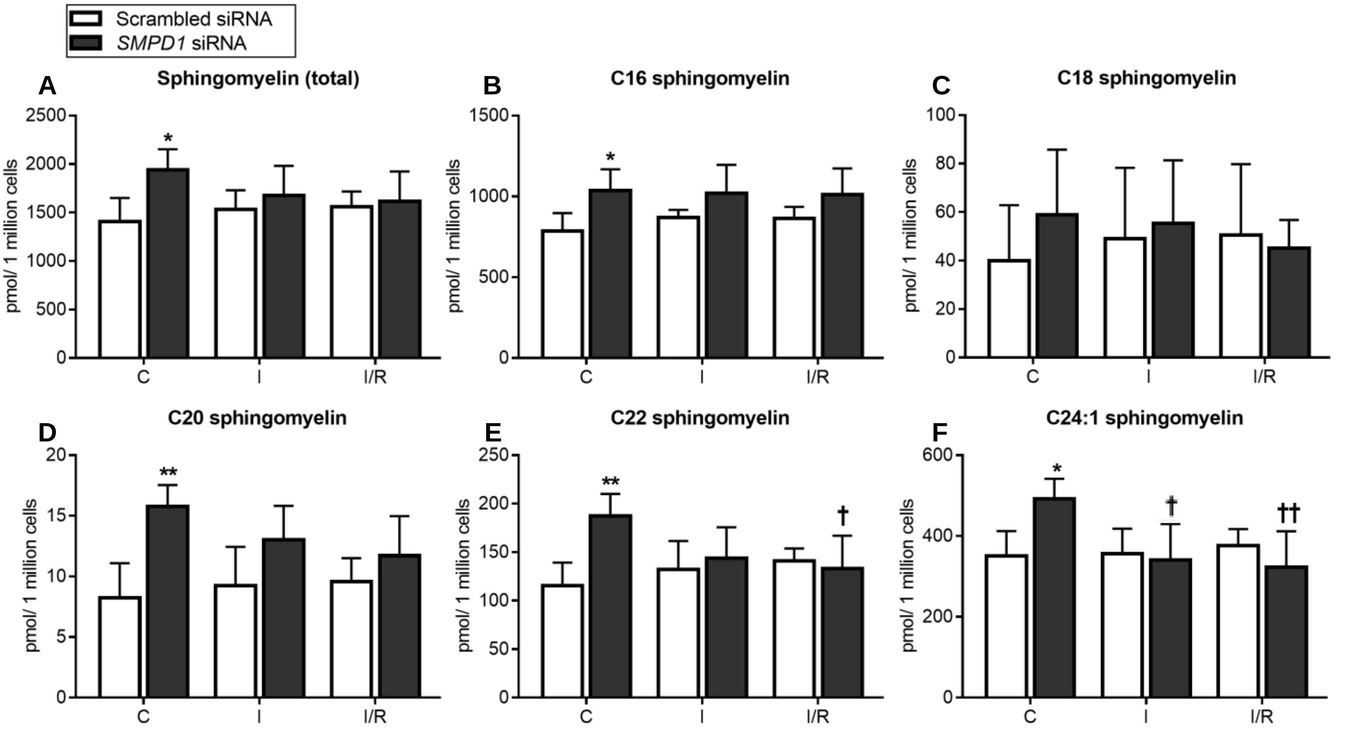

Supplementary Figure 17. *SMPD1* knockdown increases sphingomyelin levels in cerebral microvascular endothelial cells *in vitro*. **(A)** Total sphingomyelin, **(B)** C16 sphingomyelin, **(C)** C18 sphingomyelin, **(D)** C20 sphingomyelin, **(E)** C22 sphingomyelin and **(F)** C24:1 sphingomyelin content of hCMEC/D3 exposed to non-ischemic control condition (C), OGD (that is, ischemia; I) or ischemia followed by reoxygenation/glucose re-supplementation (I/R), which were transfected with scrambled siRNA (used as control) or *SMPD1* siRNA. Sphingomyelin levels were measured by LC-MS/MS. Data are means ± SD values. *$p \leq 0.05$/**$p \leq 0.01$ compared with corresponding scrambled siRNA; $^{\dagger}p \leq 0.05$/$^{\dagger\dagger}p \leq 0.01$ compared with corresponding C (n=3 independent samples/ group [in **(A-F)**]; analyzed by two-way ANOVA followed by LSD tests).

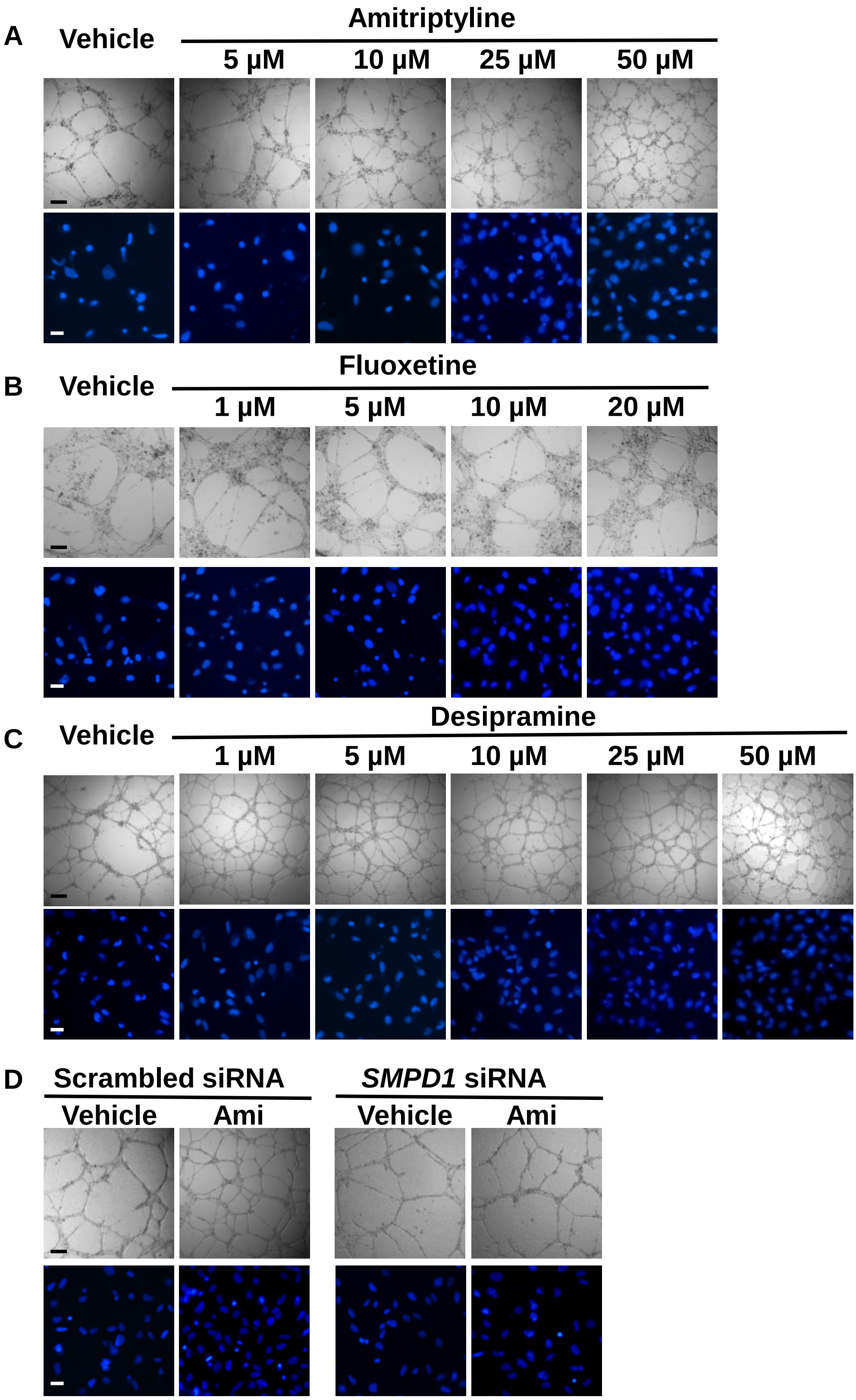

Supplementary Figure 18. Amitriptyline, fluoxetine and desipramine promote cerebral angiogenesis *in vitro* in an ASM dependent way. Representative examples of matrigel-based tube formation and migration assays of hCMEC/D3 treated with vehicle, **(A)** amitriptyline (0-50 µM), **(B)** fluoxetine (0-20 µM) or **(C)** desipramine (0-50 µM) or of **(D)** hCMEC/D3 transfected with scrambled siRNA or *SMPD1* siRNA which were exposed to vehicle or amitriptyline (Ami, 50 µM). Scale bars in tube formation photographs, 200 µm; in transwell migration photographs, 20 µm.

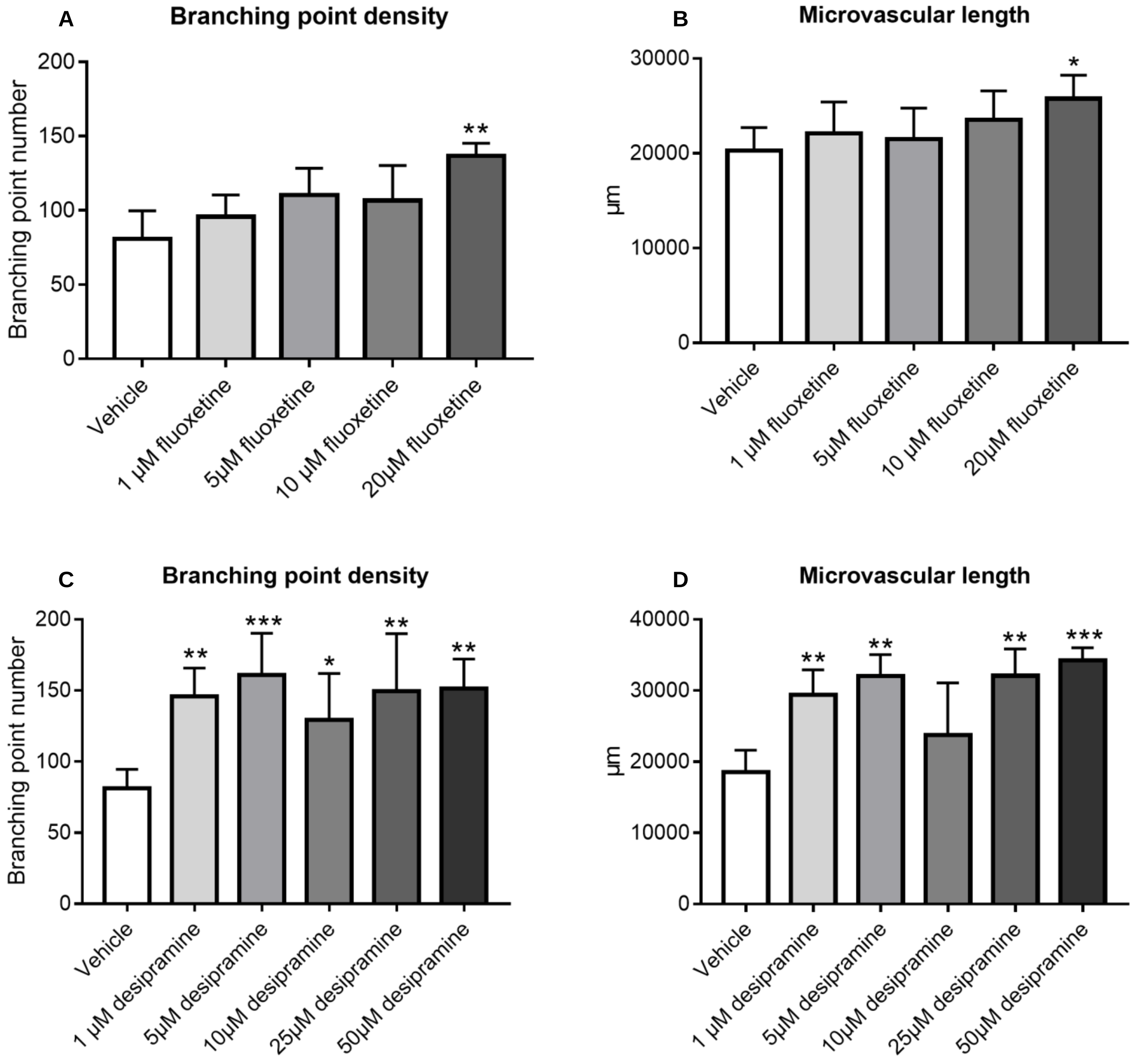

Supplementary Figure 19. ASM inhibitors fluoxetine and desipramine promote cerebral angiogenesis *in vitro*. (A, C) Branching point density and **(B, D)** microvascular length evaluated in the matrigel-based tube formation assay in hCMEC/D3 exposed to vehicle, fluoxetine (0-20 µM) or desipramine (0-50 µM). Data are means ± SD values. *p≤0.05/**p≤0.01/***p≤0.001 compared with corresponding vehicle (n=3-4 independent samples/ group; analyzed by one-way ANOVA followed by LSD tests).

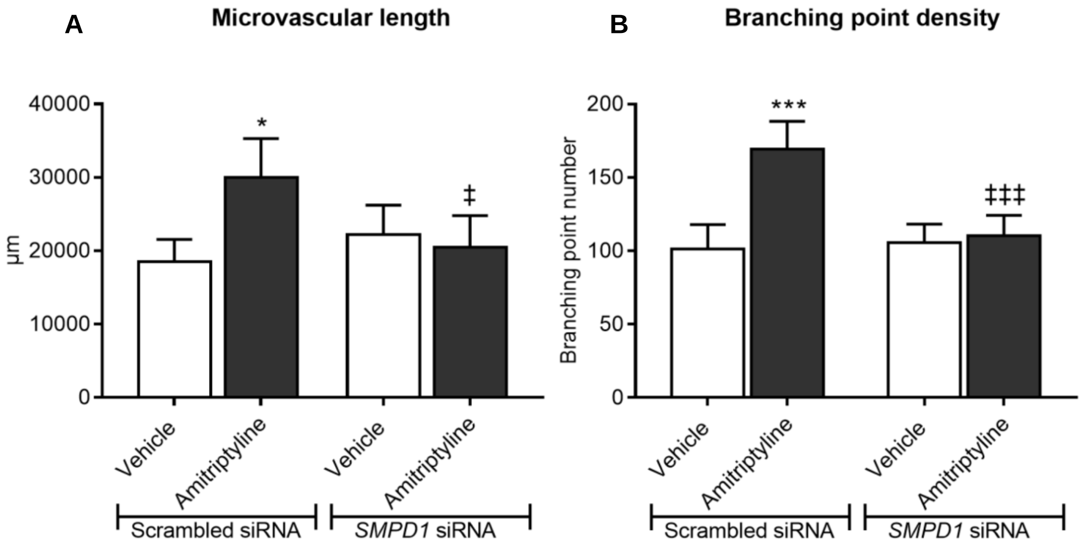

Supplementary Figure 20. Amitriptyline promotes cerebral angiogenesis *in vitro* in an ASM dependent way. (A) Microvascular length and **(B)** branching point density evaluated in the matrigel-based tube formation assay of non-ischemic hCMEC/D3 transfected with scrambled siRNA (used as control) or *SMPD1* siRNA which were exposed to vehicle or amitriptyline (50 µM). Representative photographs for tube formation assays are shown in Figure 4. Data are means ± SD values. *$p \leq 0.05$/***$p \leq 0.001$ compared with corresponding vehicle; ‡$p \leq 0.05$/‡‡‡$p \leq 0.001$ compared with corresponding scrambled siRNA (n=5-8 independent samples/group; analyzed by two-way ANOVA followed by LSD tests).

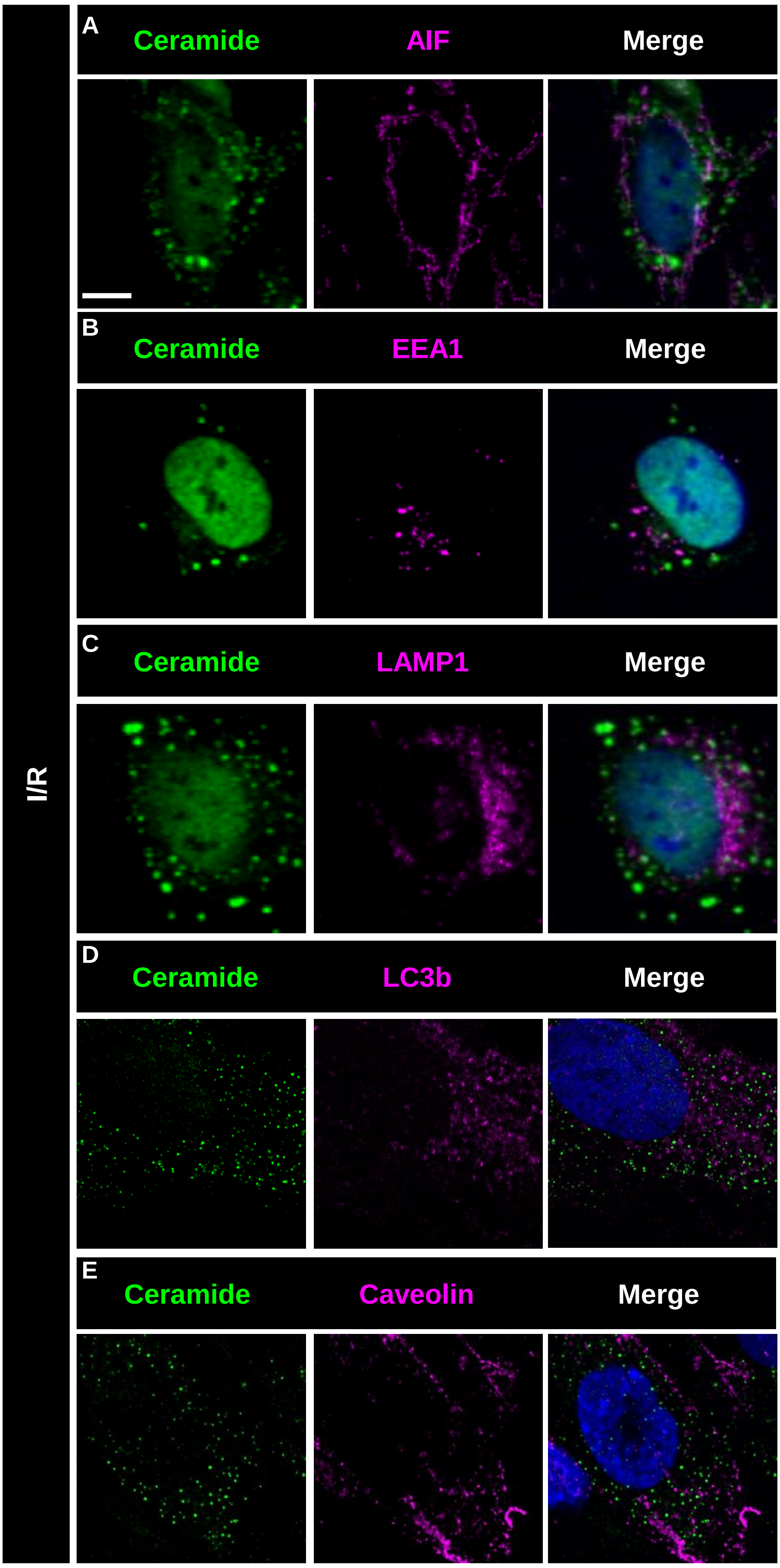

Supplementary Figure 21. Intracellular ceramide-rich vesicles do not express mitochondrial, early endosome, lysosome, autophagosome and caveolae markers. Immunocytochemistry for ceramide (in green) and **(A)** the mitochondrial marker apoptosis-inducing factor (AIF), **(B)** the early endosome marker early endosome antigen-1 (EEA1), **(C)** the lysosome marker lysosomal-associated membrane protein-1 (LAMP1), **(D)** the autophagosome marker microtubule-associated protein light chain-3b (LC3b) and **(E)** the caveolae marker caveolin (all in magenta) of hCMEC/D3 exposed to 24 hours OGD (that is, ischemia) followed by 3 hours reoxygenation/glucose re-supplementation (I/R). In the merged photomicrographs, no double labeled cells are visible. Nuclei were counterstained with Hoechst 33342 (in blue). Scale bar, 5 µm. Shown are representative results from 4 independent studies.

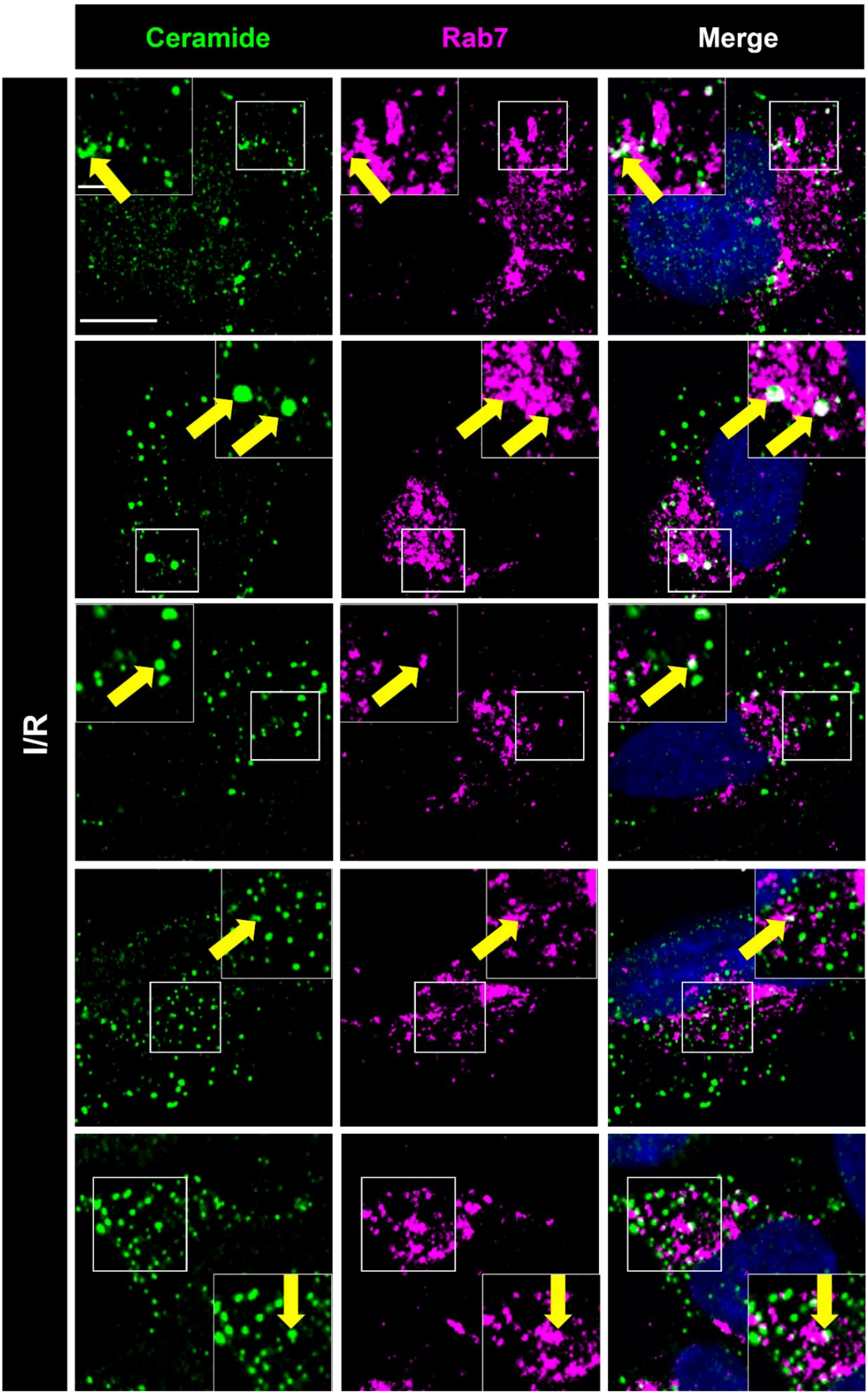

Supplementary Figure 22. Intracellular ceramide-rich vesicles co-localize with late endosome marker Rab7. Immunohistochemistry for ceramide (in green) and the late endosome marker Rab7 (in magenta) in hCMEC/D3 exposed to 24 hours ischemia followed by 3 hours reoxygenation/glucose re-supplementation (I/R). In the merged photographs, double labeled cells are shown in white (selected cells labeled with arrow; nuclei were counterstained in blue with Hoechst 33342). Scale bar in overview photograph, 10 µm; in magnification, 5 µm.

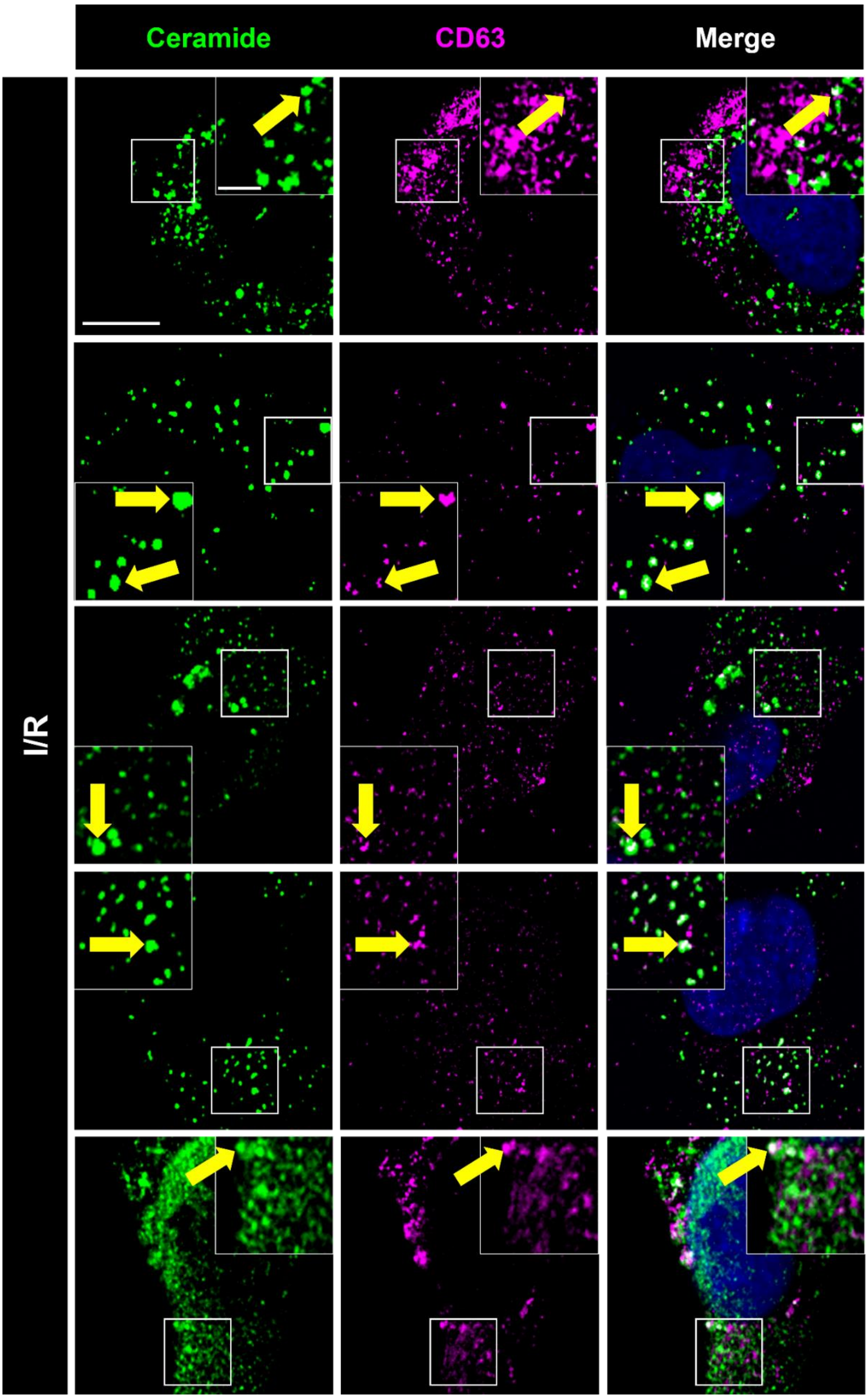

Supplementary Figure 23. Intracellular ceramide-rich vesicles co-localize with MVB marker CD63. Immunohistochemistry for ceramide (in green) and the multivesicular body (MVB) marker CD63 (in magenta) of hCMEC/D3 exposed to 24 hours ischemia followed by 3 hours reoxygenation/glucose re-supplementation (I/R). In the merged photographs, double labeled cells are shown in white (selected cells labeled with arrow; nuclei were counterstained in blue with Hoechst 33342). Scale bar in overview photograph, 10 µm; in magnification, 5 µm.

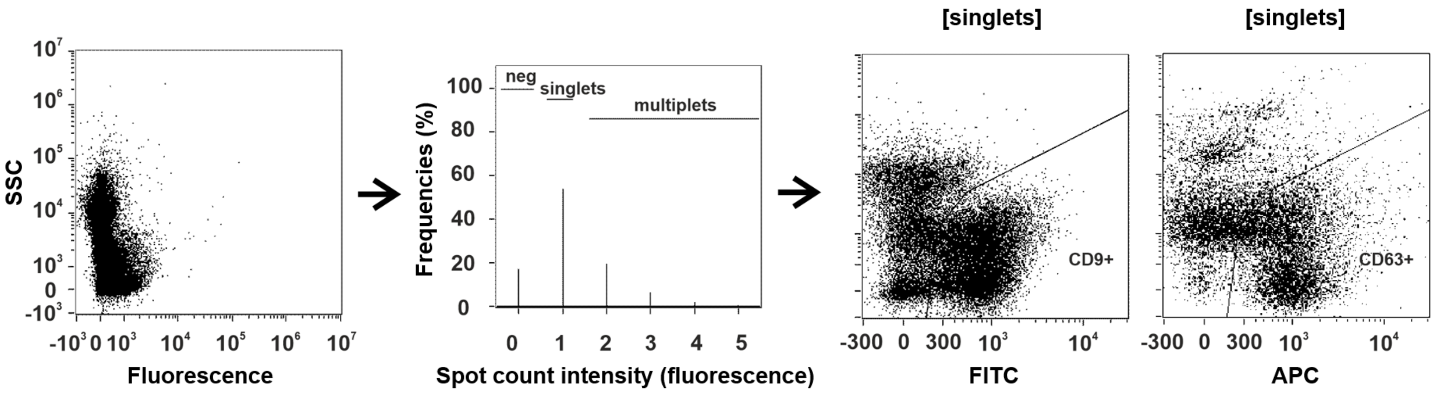

Supplementary Figure 24. Gating strategy for evaluating sEVs by ImageStreamX flow cytometry. From all recorded signals (1st plot from left), signals not showing spot count signal or signal multiplets were excluded (2nd plot from left). In the two representative plots on the right, side scatter (SSC) intensities of single objects are plotted against the fluorescence intensities of $CD9^+$ (labeled with FITC) or $CD63^+$ (labeled with APC) objects. Shown are representative results under control conditions from 4-9 independent studies.

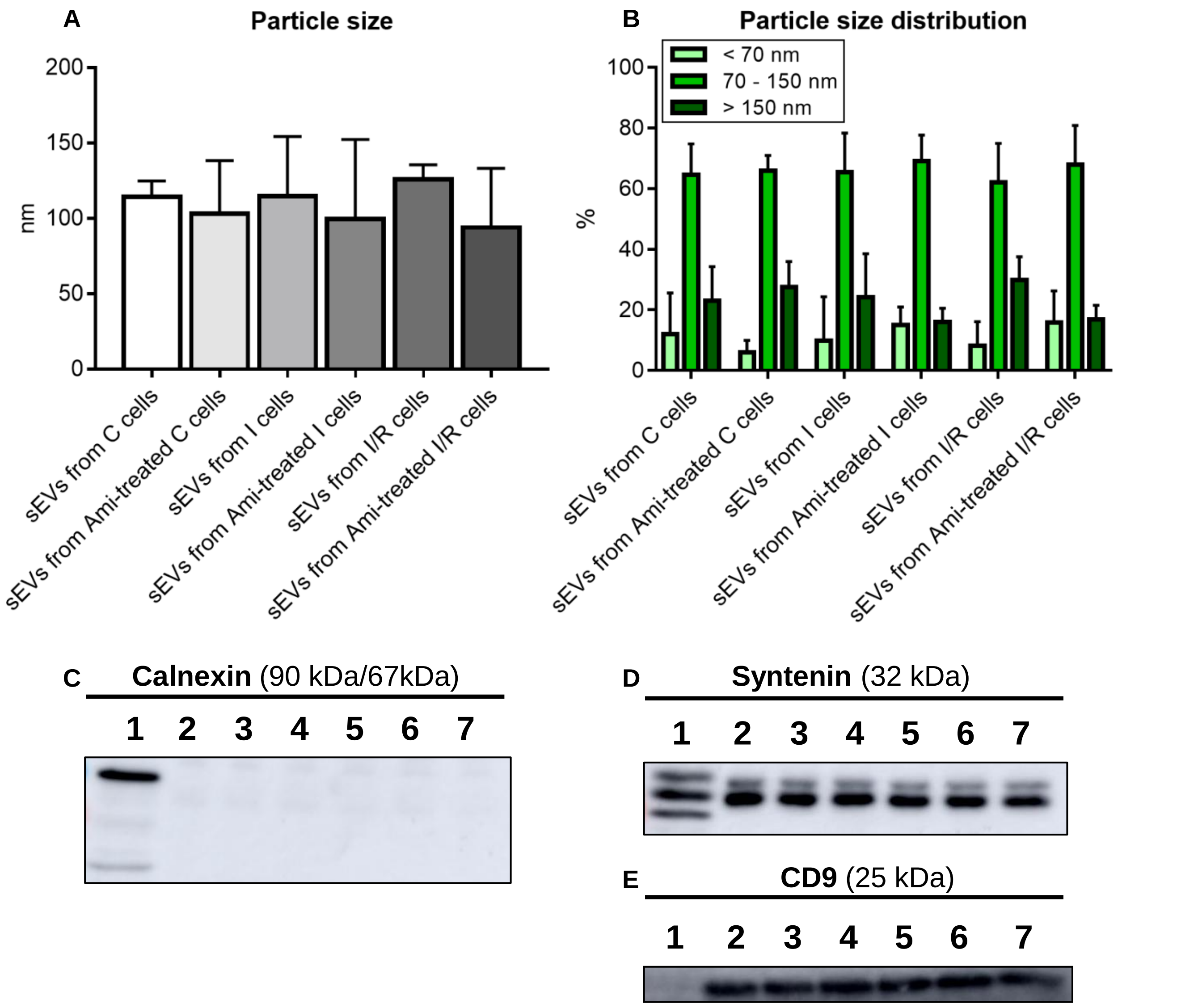

Supplementary Figure 25. sEVs released from cerebral microvascular endothelial cells have the physicochemical properties and protein expression characteristics of exosomes. (A) Particle size and **(B)** particle size distribution (<70 nm, 70 – 150 nm and >150 nm) evaluated by nanoparticle tracking analysis (NTA) of sEVs obtained from supernatants of hCMEC/D3 exposed to non-ischemic control condition (C), OGD (that is, ischemia; I) or ischemia followed by reoxygenation/glucose re-supplementation (I/R) which were treated with vehicle or amitriptyline (Ami, 50 µM). Western blots for the **(C)** the cellular contamination marker calnexin, and **(D, E)** the exosomal markers syntenin and CD9 using protein samples obtained from (1) hCMEC/D3 lysates, (2) sEVs from vehicle-treated non-ischemic control hCMEC/D3, (3) sEVs from amitriptyline-treated control hCMEC/D3, (4) sEVs from vehicle-treated ischemic hCMEC/D3, (5) sEVs from amitriptyline-treated ischemic hCMEC/D3, (6) sEVs from vehicle-treated I/R hCMEC/D3 and (7) sEVs from amitriptyline-treated I/R hCMEC/D3. Note the presence of 32 kDa synthenin and 25 kDa CD9, but absence of 90 kDa/ 67 kDa calnexin in sEVs samples. Data are means ± SD values. No significant differences were noted between groups (n=3 independent samples/group; analyzed by one-way ANOVA).

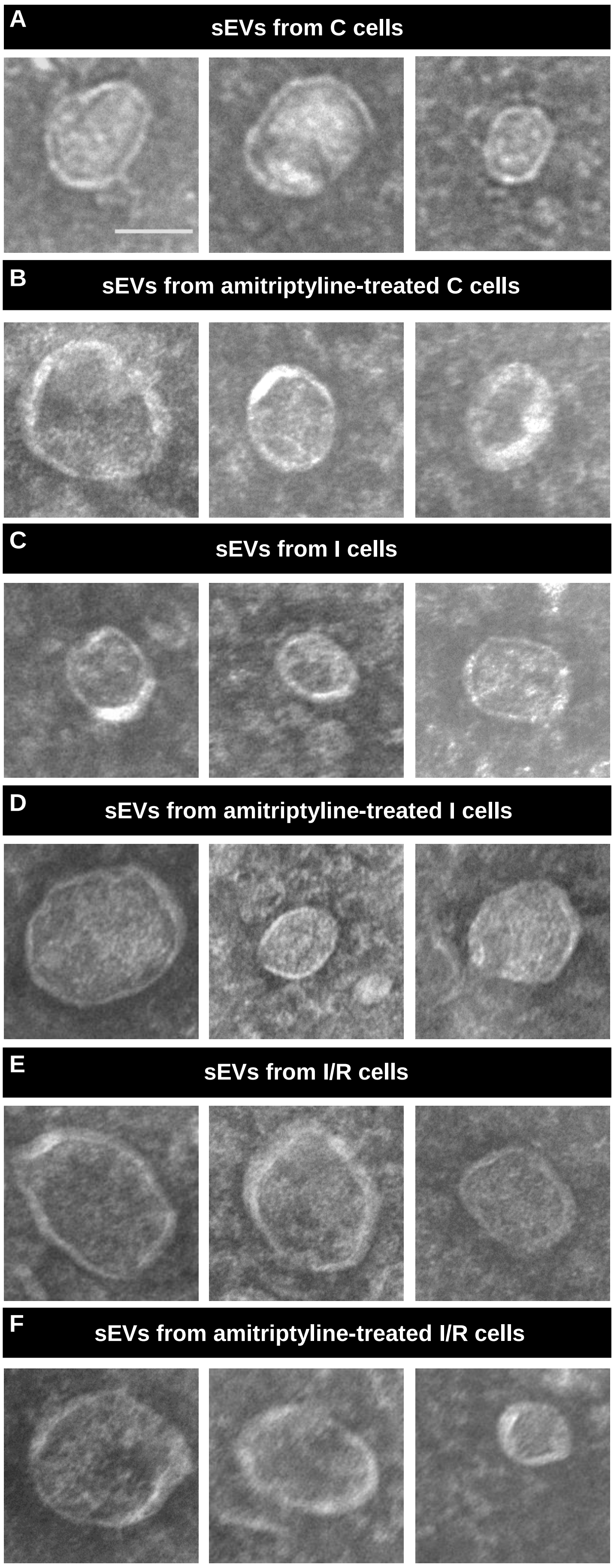

Supplementary Figure 26. sEVs released from cerebral microvascular endothelial cells have the electron microscopic size and appearance of exosomes. Transmission electron microscopy showing representative sEVs obtained from supernatants of hCMEC/D3 exposed to non-ischemic control condition (C), OGD (that is, ischemia; I) or ischemia followed by reoxygenation/glucose re-supplementation (I/R), which had been treated with vehicle or amitriptyline (50 µM), while cells were cultured. The typical cup shape double contrast is suggestive for a double-layered membrane structure that is found in sEVs. Scale bar, 100 nm.

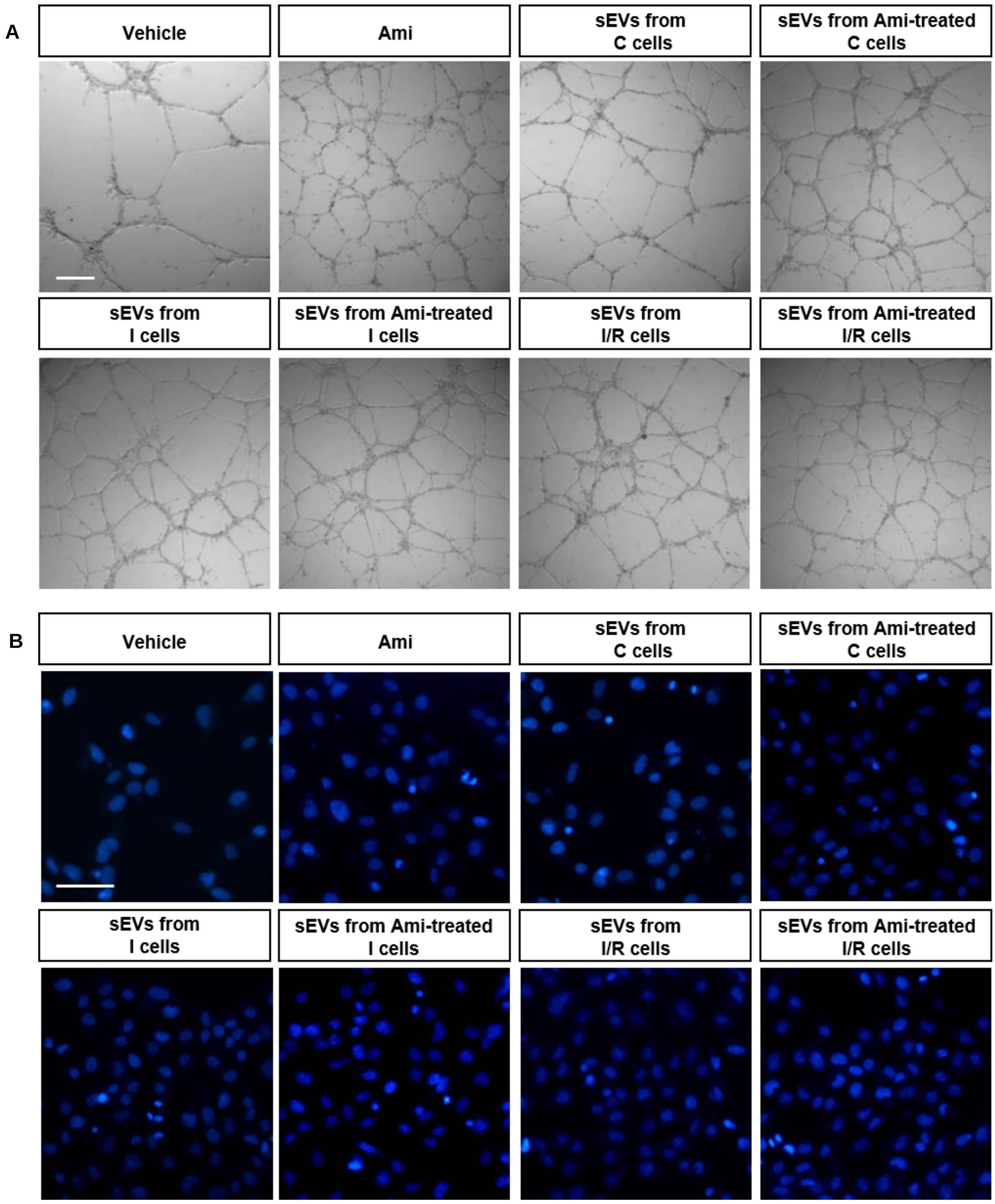

Supplementary Figure 27. sEVs obtained from supernatants of cerebral microvascular endothelial cells exposed to ASM inhibitor amitriptyline have angiogenic activity that resembles sEVs released by endothelial cells during I/R. Representative photographs of **(A)** matrigel-based tube formation and **(B)** transwell migration assays of hCMEC/D3, which were treated with vehicle, amitriptyline (Ami, 50 µM) or sEVs (25 µg protein/ ml) isolated from the supernatants of hCMEC/D3 that had been cultured in non-ischemic control condition (C), OGD (that is, ischemia; I) or ischemia followed by reoxygenation/glucose re-supplementation (I/R) and had been treated with vehicle or amitriptyline (50 µM) while supernatants were collected. A quantitative analysis is shown in **Figure 7**. Scale bar in **(A)**, 200 µm; in **(B)**, 20 µm.

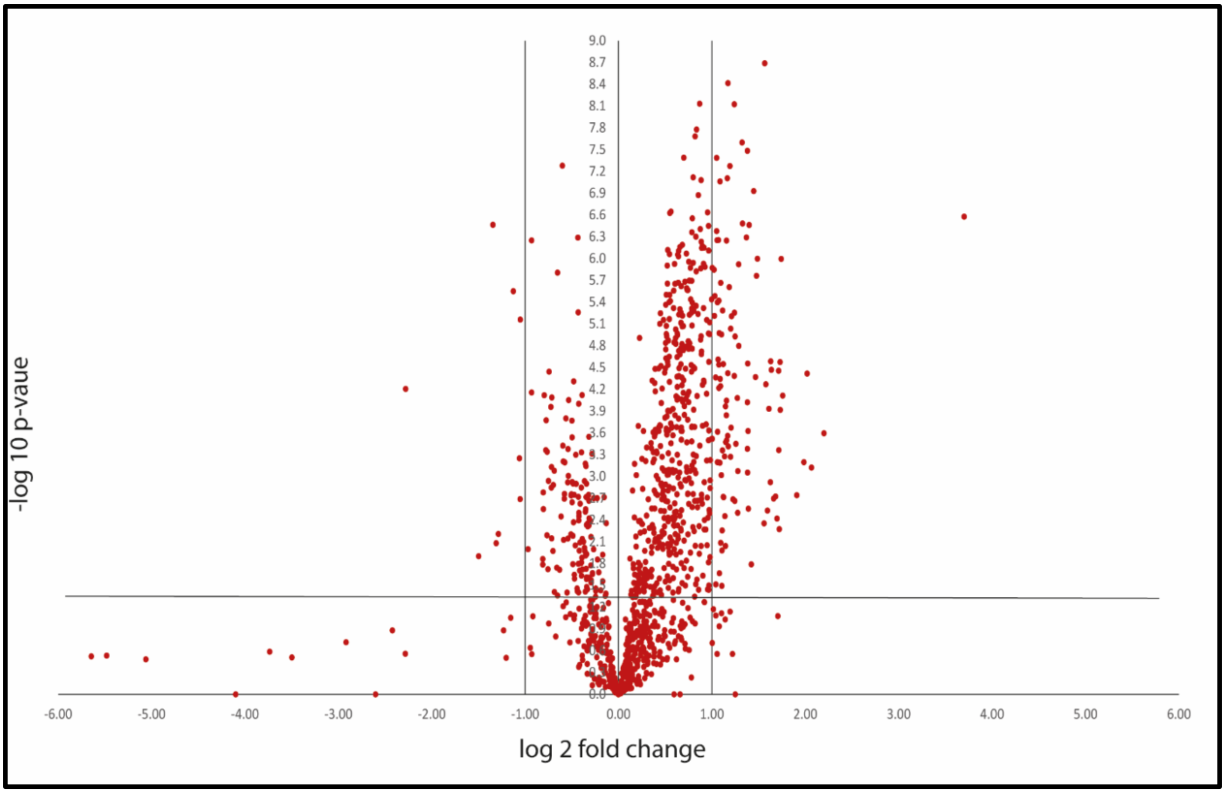

Supplementary Figure 28. Volcano plot showing differentially regulated proteins in sEVs released from hCMEC/D3 cultured under non-ischemic control conditions that had been exposed to vehicle or amitriptyline (50 µM). Proteins were evaluated by LC-MS-based proteomics. Proteins with statistically significant differential regulation (≥2-fold, p value ≤0.05; analyzed by two-tailed t tests) are located in the top left and right sectors (n=6 independent samples/group).
