# Supplementary material for: Acid sphingomyelinase deactivation post-ischemia promotes brain angiogenesis and remodeling by small extracellular vesicles
Source: Basic Res Cardiol. 2022 Aug 29;117(1):43. doi: 10.1007/s00395-022-00950-7 (PMC9424180; doi:10.1007/s00395-022-00950-7)
Supplement: Supplementary file 2 — Supplementary file2 (PDF 709 KB) [file 395_2022_950_MOESM2_ESM.pdf]

SUPPLEMENTARY TABLES

Acid sphingomyelinase deactivation post-ischemia promotes brain angiogenesis and remodeling by small extracellular vesicles

Basic Research in Cardiology

Ayan Mohamud Yusuf,[1,2] Nina Hagemann,[1,2] Xiaoni Zhang,[1,2] Maria Zafar,[1,2] Tanja Hussner,[1,2] Carolin Bromkamp,[1,2] Carlotta Martiny,[1,2] Tobias Tertel,[3] Verena Börger,[3] Fabian Schumacher,[4,5,6] Fiorella A. Solari,[7] Mike Hasenberg,[8] Christoph Kleinschnitz,[1,2] Thorsten R. Doeppner,[1,2,9] Burkhard Kleuser,[5] Albert Sickmann,[7,10,11] Matthias Gunzer,[7,8] Bernd Giebel,[3] Richard Kolesnick,[12] Erich Gulbins,[4] Dirk M. Hermann[1,2]*

[1]Department of Neurology, [2]Center for Translational and Behavioral Neurosciences, [3]Institute of Transfusion Medicine and [4]Institute of Molecular Biology, University Hospital Essen, Essen, Germany, [5]Department of Toxicology, University of Potsdam, Nuthetal, Germany, [6]Institute of Pharmacy, Freie Universität Berlin, Berlin, Germany, [7]Leibniz-Institut für Analytische Wissenschaften-ISAS-e.V., Dortmund, Germany, [8]Institute of Immunology and Experimental Imaging, University Hospital Essen, Essen, Germany, [9]Department of Neurology, University Medicine Göttingen, Göttingen, Germany, [10]Medizinisches Proteom-Center (MPC), Ruhr University, Bochum, Germany, [11]Department of Chemistry, College of Physical Sciences, University of Aberdeen, Aberdeen, Scotland, U.K., [12]Memorial Sloan Kettering Cancer Center, New York, New York, U.S.A.; *corresponding author.

Correspondence: Prof. Dirk M. Hermann, MD, Department of Neurology, University Hospital Essen, Hufelandstr. 55, D-45122 Essen, Germany, E-Mail: dirk.hermann@uk-essen.de

Supplementary Table 1. Proteins significantly upregulated in sEVs released by hCMEC/D3 exposed to amitriptyline.

Association according to KEGG pathway database:	
(blue)	Phagosome
(green)	Lysosome
(yellow)	Protein export
(peach)	Antigen processing and presentation
(red)	Epstein-Barr virus infection
(grey-blue)	Viral myocarditis

Accession	Description	Protein name	Log 2 fold change	P-value
Q8NE71	ATP-binding cassette sub-family F member 1	ABCF1	1.12	0.00
P11310	Medium-chain specific acyl-CoA dehydrogenase, mitochondrial	ACADM	1.73	0.00
Q13443	Disintegrin and metalloproteinase domain-containing protein 9	ADAM9	1.38	0.00
Q13155	Aminoacyl tRNA synthase complex-interacting multifunctional protein 2	AIMP2	1.09	0.00
Q13740	CD166 antigen	ALCAM	1.20	0.00
P09972	Fructose-bisphosphate aldolase C	ALDOC	1.04	0.03
P15144	Aminopeptidase N	AN	1.10	0.00
Q9BXS5	AP-1 complex subunit mu-1	AP1M1	1.06	0.00
Q92572	AP-3 complex subunit sigma-1	AP3S1	1.14	0.00
P05026	Sodium/potassium-transporting ATPase subunit beta-1	ATP1B1	1.00	0.00
P61769	Beta-2-microglobulin	B2M	1.21	0.00
Q05682	Caldesmon	CALD1	1.14	0.00
P27824	Calnexin	CANX	1.28	0.00
Q6NZI2	Caveolae-associated protein 1	CAVIN1	1.63	0.00
P45973	Chromobox protein homolog 5	CBX5	1.33	0.00
P16070	CD44 antigen	CD44	1.09	0.00
P15529	Membrane cofactor protein	CD46	1.28	0.00
P08174	Complement decay-accelerating factor	CD55	1.38	0.00
P48960	CD97 antigen	CD97	1.17	0.00
Q14008	Cytoskeleton-associated protein 5	CKAP5	1.11	0.00
Q6UVK1	Chondroitin sulfate proteoglycan 4	CSPG4	1.40	0.00
P07858	Cathepsin B	CTSB	1.06	0.00
P07339	Cathepsin D	CTSD	1.07	0.00
Q14247	Src substrate cortactin	CTTN	1.42	0.02
P17844	Probable ATP-dependent RNA helicase DDX5	DDX5	1.03	0.00
P31689	DnaJ homolog subfamily A member 1	DNAJA1	1.09	0.00
P23919	Thymidylate kinase	DTYMK	1.56	0.00
Q9BY44	Eukaryotic translation initiation factor 2A	EIF2A	1.23	0.00
P19525	Interferon-induced, double-stranded RNA-activated protein kinase	EIF2AK2	1.74	0.00
O75821	Eukaryotic translation initiation factor 3 subunit G	EIF3G	1.11	0.01
Q7L2H7	Eukaryotic translation initiation factor 3 subunit M	EIF3M	1.66	0.00
Q04637	Eukaryotic translation initiation factor 4 gamma 1	EIF4G1	1.05	0.00
P13804	Electron transfer flavoprotein subunit alpha, mitochondrial	ETFA	1.16	0.00
Q96A65	Exocyst complex component 4	EXOC4	2.02	0.00
Q13451	Peptidyl-prolyl cis-trans isomerase FKBP5	FKBP5	1.09	0.00
P35637	RNA-binding protein FUS	FUS	1.47	0.00
P51114	Fragile X mental retardation syndrome-related protein 1	FXR1	1.08	0.00
P60520	Gamma-aminobutyric acid receptor-associated protein-like 2	GABARAPL2	1.58	0.00
Q96RP9	Elongation factor G, mitochondrial	GFM1	1.06	0.00
P16278	Beta-galactosidase	GLB1	1.68	0.00
Q14344	Guanine nucleotide-binding protein subunit alpha-13	GNA13	2.20	0.00
P08236	Beta-glucuronidase	GUSB	1.98	0.00
P19367	Hexokinase-1	HK1	1.64	0.00
P01889	HLA class I histocompatibility antigen, B alpha chain	HLA-B	1.24	0.00
P10321	HLA class I histocompatibility antigen, C alpha chain	HLA-C	1.25	0.00
P51991	Heterogeneous nuclear ribonucleoprotein A3	HNRNPA3	1.63	0.00
P31943	Heterogeneous nuclear ribonucleoprotein H	HNRNPH1	1.08	0.00
P14866	Heterogeneous nuclear ribonucleoprotein L	HNRNPL	1.00	0.00
Q00839	Heterogeneous nuclear ribonucleoprotein U	HNRNPU	1.07	0.00
Q16666	Gamma-interferon-inducible protein 16	IFI16	1.38	0.00
Q9Y6M1	Insulin-like growth factor 2 mRNA-binding protein 2	IGF2BP2	1.29	0.00
P08648	Integrin alpha-5	ITGA5	1.38	0.00
P33176	Kinesin-1 heavy chain	KIF5B	1.19	0.00
P52292	Importin subunit alpha-1	KPNA2	1.39	0.00
P11279	Lysosome-associated membrane glycoprotein 1	LAMP1	1.24	0.00
Q9UHB6	LIM domain and actin-binding protein 1	LIMA1	1.38	0.00
Q8N1G4	Leucine-rich repeat-containing protein 47	LRRC47	1.11	0.01
P27816	Microtubule-associated protein 4	MAP4	1.32	0.00
P43243	Matrin-3	MATR3	1.73	0.00
P43121	Cell surface glycoprotein MUC18	MCAM	1.20	0.00
Q14566	DNA replication licensing factor MCM6	MCM6	1.26	0.00
O95297	Myelin protein zero-like protein 1	MPZL1	1.15	0.00
P42285	Exosome RNA helicase MTR4	MTREX	1.61	0.00
P20591	Interferon-induced GTP-binding protein Mx1	MX1	1.37	0.00
Q92542	Nicastrin	NCSTN	1.72	0.00
P09874	Poly [ADP-ribose] polymerase 1	PARP1	1.06	0.00

Comparison of n=6 different samples/group obtained from 10 independent cell cultures.

Supplementary Table 1. Proteins significantly upregulated in sEVs released by hCMEC/D3 exposed to amitriptyline.

Association according to KEGG pathway database:	
(blue)	Phagosome
(green)	Lysosome
(yellow)	Protein export
(peach)	Antigen processing and presentation
(red)	Epstein-Barr virus infection
(grey-blue)	Viral myocarditis

Accession	Description	Protein name	Log 2 fold change	P-value
O60664	Perilipin-3	PLIN3	1.04	0.00
P30876	DNA-directed RNA polymerase II subunit RPB2	POLR2B	1.15	0.00
Q06830	Peroxiredoxin-1	PRDX1	1.05	0.00
O75475	PC4 and SFRS1-interacting protein	PSIP1	1.57	0.00
P28066	Proteasome subunit alpha type-5	PSMA5	1.70	0.00
Q06323	Proteasome activator complex subunit 1	PSME1	1.39	0.00
Q9UKM9	RNA-binding protein Raly	RALY	1.06	0.00
Q14498	RNA-binding protein 39	RBM39	1.27	0.00
Q04206	Transcription factor p65	RELA	1.18	0.00
P13489	Ribonuclease inhibitor	RNH1	1.17	0.00
P27635	60S ribosomal protein L10	RPL10	1.00	0.00
P35268	60S ribosomal protein L22	RPL22	1.17	0.00
P36578	60S ribosomal protein L4	RPL4	1.11	0.00
P62841	40S ribosomal protein S15	RPS15	1.72	0.01
Q9P2E9	Ribosome-binding protein 1	RRBP1	1.10	0.00
Q14108	Lysosome membrane protein 2	SCARB2	1.48	0.00
P53985	Monocarboxylate transporter 1	SLC16A1	1.12	0.00
Q9H2H9	Sodium-coupled neutral amino acid transporter 1	SLC38A1	1.21	0.00
Q96QD8	Sodium-coupled neutral amino acid transporter 2	SLC38A2	1.07	0.00
Q15043	Zinc transporter ZIP14	SLC39A14	1.13	0.00
P08195	4F2 cell-surface antigen heavy chain	SLC3A2	1.45	0.00
P30825	High affinity cationic amino acid transporter 1	SLC7A1	1.08	0.02
Q01650	Large neutral amino acids transporter small subunit 1	SLC7A5	1.16	0.00
O60264	SWI/SNF-related matrix-associated actin-dependent regulator of chromatin subfamily A member 5	SMARCA5	1.03	0.00
Q8TAQ2	SWI/SNF complex subunit SMARCC2	SMARCC2	1.16	0.00
O95347	Structural maintenance of chromosomes protein 2	SMC2	1.76	0.00
Q9UQE7	Structural maintenance of chromosomes protein 3	SMC3	1.91	0.00
Q13501	Sequestosome-1	SQSTM1	3.70	0.00
P61011	Signal recognition particle 54 kDa protein	SRP54	1.15	0.00
Q9UHB9	Signal recognition particle subunit SRP68	SRP68	1.17	0.00
O76094	Signal recognition particle subunit SRP72	SRP72	1.09	0.00
P05455	Lupus La protein	SSB	1.00	0.00
O15400	Syntaxin-7	STX7	1.15	0.01
Q13428	Treacle protein	TCOF1	1.49	0.00
Q9Y5L0	Transportin-3	TNPO3	1.60	0.00
P06753	Tropomyosin alpha-3 chain	TPM3	1.09	0.01
Q13263	Transcription intermediary factor 1-beta	TRIM28	1.29	0.00
P49411	Elongation factor Tu, mitochondrial	TUFM	1.02	0.00
P26368	Splicing factor U2AF 65 kDa subunit	U2AF2	1.72	0.00
O94874	E3 UFM1-protein ligase 1	UFL1	1.08	0.00
Q16739	Ceramide glucosyltransferase	UGCG	1.11	0.03
Q08AM6	Protein VAC14 homolog	VAC14	1.24	0.00
Q15836	Vesicle-associated membrane protein 3	VAMP3	2.07	0.00
P08670	Vimentin	VIM	1.19	0.00
O75436	Vacuolar protein sorting-associated protein 26A	VPS26A	1.25	0.00

Comparison of n=6 different samples/group obtained from 10 independent cell cultures.

Supplementary Table 2. Proteins significantly downregulated in sEVs released by hCMEC/D3 exposed to amitriptyline.

Association according to KEGG pathway database:	
(yellow)	Extracellular matrix - receptor interaction and focal adhesion

Accession	Description	Protein name	Log 2 fold change	P-value
P24821	Tenascin	TNC	-1.05	0.00
P02647	Apolipoprotein A-I	APOA1	-1.05	0.00
P08572	Collagen alpha-2(IV) chain	COL4A2	-1.06	0.00
O00622	CCN family member 1	CCN1	-1.13	0.00
Q96N76	Urocanate hydratase	UROC1	-1.29	0.01
P58107	Epiplakin	EPPK1	-1.31	0.01
P02788	Lactotransferrin	LTF	-1.34	0.00
O76074	cGMP-specific 3',5'-cyclic phosphodiesterase	PDE5A	-1.50	0.01
P26022	Pentraxin-related protein PTX3	PTX3	-2.28	0.00

Comparison of n=6 different samples/group obtained from 10 independent cell cultures.

Supplementary Table 3. Differentially regulated protein networks identified by KEGG pathway database analysis.

Term ID	Term description	Observed gene count	Background gene count	Strength	False discovery rate	Matching proteins in the network
hsa04145	Phagosome	7	145	0.93	0.0014	VAMP3, CANX, ITGA5, LAMP1, STX7, HLA-C, HLA-B
hsa04142	Lysosome	8	123	1.06	0.00013	CTSD, SCARB2, GUSB, GLB1, AP3S1, LAMP1,CTSB, AP1M1
hsa03060	Protein export	3	23	1.36	0.0123	SRP68, SRP72, SRP54
hsa04612	Antigen processing and presentation	6	66	1.2	0.00027	CANX, CTSB, HLA-C, PSME1, HLA-B, B2M
hsa05169	Epstein-Barr virus infection	7	194	0.8	0.0061	EIF2AK2, HLA-C, POLR2B, RELA, CD44, HLA-B, VIM
hsa05416	Viral myocarditis	4	56	1.1	0.0123	CD55, HLA-C, HLA-B, EIF4G1

Term ID	Term description	Observed gene count	Background gene count	Strength	False discovery rate	Matching proteins in the network
hsa04512	ECM-receptor interaction	2	81	1.73	0.0125	TNC,COL4A2
hsa04510	Focal adhesion	2	197	1.34	0.0353	TNC,COL4A2

Comparison of n=6 different samples/group obtained from 10 independent cell cultures.

Supplementary Table 4. List of all proteins identified by label free proteomics analysis.

Accession	Description	Coverage [%]	# PSMs	# Unique Peptides	Amitriptyline 1	Amitriptyline 2	Amitriptyline 3	Amitriptyline 4	Amitriptyline 5	Amitriptyline 6	Vehicle 1	Vehicle 2	Vehicle 3	Vehicle 4	Vehicle 5	Vehicle 6	Average amitriptyline	Average vehicle	Ratio amitriptyline/ vehicle	Log 2 fold change (amitriptyline /vehicle)	P-value
P68371	Tubulin beta-4B chain OS=Homo sapiens OX=9606 GN=TUBB4B PE=1 SV=1	81	5349	2	1.70E+09	1.58E+09	1.79E+09	1.67E+09	1.72E+09	1.81E+09	1.39E+09	1.39E+09	1.58E+09	1.43E+09	1.55E+09	1.68E+09	1.71E+09	1.50E+09	1.14	0.19	0.00672027
Q71U36	Tubulin alpha-1A chain OS=Homo sapiens OX=9606 GN=TUBA1A PE=1 SV=1	77	3804	2	7.25E+06	8.77E+06	1.75E+07	1.02E+07	1.02E+07	1.47E+07	1.08E+07	1.02E+07	1.36E+07	6.14E+06	1.58E+07	1.32E+07	1.14E+07	1.16E+07	0.98	-0.02	0.927398077
Q9BQE3	Tubulin alpha-1C chain OS=Homo sapiens OX=9606 GN=TUBA1C PE=1 SV=1	73	3200	2	1.78E+09	1.89E+09	1.99E+09	1.71E+09	1.82E+09	1.86E+09	1.55E+09	1.60E+09	1.61E+09	1.62E+09	1.75E+09	1.55E+09	1.84E+09	1.61E+09	1.14	0.19	0.000963406
Q7L7L0	Histone H2A type 3 OS=Homo sapiens OX=9606 GN=HIST3H2A PE=1 SV=3	64	2655	2	2.31E+08	2.19E+08	3.22E+08	2.42E+08	2.00E+08	1.76E+08	2.05E+08	2.78E+08	2.58E+08	3.62E+08	2.67E+08	1.64E+08	2.32E+08	2.55E+08	0.91	-0.14	0.505091271
P10412	Histone H1.4 OS=Homo sapiens OX=9606 GN=H1-4 PE=1 SV=2	39	706	2	1.89E+09	1.91E+09	1.70E+09	1.82E+09	1.81E+09	1.75E+09	1.60E+09	1.69E+09	1.70E+09	1.62E+09	1.60E+09	1.58E+09	1.81E+09	1.63E+09	1.11	0.15	0.001566557
P04259	Keratin, type II cytoskeletal 6B OS=Homo sapiens OX=9606 GN=KRT6B PE=1 SV=5	31	563	2	4.07E+06	9.78E+05	1.35E+06			2.30E+06	6.20E+05	4.04E+06	2.78E+06	2.15E+06	1.07E+06		2.17E+06	2.13E+06	1.02	0.03	0.966063547
Q562R1	Beta-actin-like protein 2 OS=Homo sapiens OX=9606 GN=ACTBL2 PE=1 SV=2	32	1264	2	1.15E+06	4.90E+05		1.31E+06	3.10E+05	1.15E+06	1.75E+05	8.19E+05	1.05E+06	2.51E+05			8.83E+05	5.74E+05	1.54	0.62	0.329252562
Q13310	Polyadenylate-binding protein 4 OS=Homo sapiens OX=9606 GN=PABPC4 PE=1 SV=1	14	88	2	1.88E+06	2.49E+06	1.84E+06	2.07E+06	2.14E+06	1.65E+06	1.74E+06	1.85E+06	1.61E+06	1.73E+06	1.23E+06	1.49E+06	2.01E+06	1.61E+06	1.25	0.32	0.023859109
P62820	Ras-related protein Rab-1A OS=Homo sapiens OX=9606 GN=RAB1A PE=1 SV=3	54	91	2	6.67E+06	8.09E+06	4.82E+06	5.06E+06	4.88E+06	4.99E+06	6.53E+06	8.04E+06	4.64E+06	5.16E+06	4.89E+06	5.34E+06	5.75E+06	5.77E+06	1.00	0.00	0.984813058
P08754	Guanine nucleotide-binding protein G(i) subunit alpha OS=Homo sapiens OX=9606 GN=GNAI3 PE=1 SV=3	21	120	2		1.66E+06	2.31E+06	2.28E+06	1.12E+06	1.23E+06	1.53E+06	1.67E+06	1.26E+06	1.62E+06		9.11E+05	1.72E+06	1.40E+06	1.23	0.30	0.304238845
P62873	Guanine nucleotide-binding protein G(I)/G(S)/G(T) subunit beta-1 OS=Homo sapiens OX=9606 GN=GNB1 PE=1 SV=3	21	174	2	7.07E+06	6.56E+06	6.88E+06	7.16E+06	7.33E+06	8.12E+06	7.13E+06	7.19E+06	6.71E+06	8.14E+06	6.80E+06	6.19E+06	7.19E+06	7.03E+06	1.02	0.03	0.652075449
O43488	Aflatoxin B1 aldehyde reductase member 2 OS=Homo sapiens OX=9606 GN=AKR7A2 PE=1 SV=3	15	34	2	1.25E+07	1.47E+07	1.20E+07	1.29E+07	1.13E+07	9.13E+06	1.03E+07	8.81E+06	1.01E+07	7.81E+06	7.67E+06	6.67E+06	1.21E+07	8.57E+06	1.41	0.49	0.004860741
Q15836	Vesicle-associated membrane protein 3 OS=Homo sapiens OX=9606 GN=VAMP3 PE=1 SV=3	24	56	2	3.17E+06	4.53E+06	5.86E+06	6.42E+06	5.78E+06	8.20E+06	8.46E+05		8.62E+05	1.61E+06	7.29E+05	2.70E+06	5.66E+06	1.35E+06	4.19	2.07	0.000754519
P50148	Guanine nucleotide-binding protein G(q) subunit alpha OS=Homo sapiens OX=9606 GN=GNAQ PE=1 SV=4	16	32	2	4.48E+06	6.55E+06	4.74E+06	4.71E+06	3.69E+06	4.47E+06	2.77E+06	3.21E+06	3.17E+06	3.90E+06	2.29E+06	3.44E+06	4.77E+06	3.13E+06	1.53	0.61	0.006232056
P28482	Mitogen-activated protein kinase 1 OS=Homo sapiens OX=9606 GN=MAPK1 PE=1 SV=3	14	74	2	7.49E+06	7.48E+06	7.84E+06	8.75E+06	7.28E+06	7.87E+06	8.94E+06	8.37E+06	9.63E+06	8.19E+06	9.13E+06	8.29E+06	7.79E+06	8.76E+06	0.89	-0.17	0.01196294
Q16629	Serine/arginine-rich splicing factor 7 OS=Homo sapiens OX=9606 GN=SRSF7 PE=1 SV=1	16	72	2	1.47E+07	1.20E+07	8.62E+06	9.13E+06	9.02E+06	9.60E+06	9.31E+06	8.31E+06	4.70E+06	8.15E+06	6.44E+06	6.95E+06	1.05E+07	7.31E+06	1.44	0.52	0.023709968
P09972	Fructose-bisphosphate aldolase C OS=Homo sapiens OX=9606 GN=ALDOC PE=1 SV=2	17	54	2	2.39E+06					2.78E+06				1.29E+06	1.48E+06	9.99E+05	2.58E+06	1.26E+06	2.06	1.04	0.030700266
P31040	Succinate dehydrogenase [ubiquinone] flavoprotein subunit, mitochondrial OS=Homo sapiens OX=9606 GN=SDHA PE=1 SV=2	6	20	2	4.37E+06	2.70E+06	2.37E+06	1.85E+06	1.80E+06	3.67E+06	3.10E+06	1.77E+06	6.09E+05	2.17E+06	2.00E+06	2.20E+06	2.79E+06	1.97E+06	1.41	0.50	0.158411389
O43809	Cleavage and polyadenylation specificity factor subunit 5 OS=Homo sapiens OX=9606 GN=NUDT21 PE=1 SV=1	21	14	2	1.71E+06	2.76E+06	2.91E+06	2.78E+06	2.99E+06	1.50E+06	1.40E+06	1.31E+06	1.98E+06	1.57E+06	8.67E+05	1.16E+06	2.44E+06	1.38E+06	1.77	0.82	0.00904725
Q9NRY6	Phospholipid scramblase 3 OS=Homo sapiens OX=9606 GN=PLSCR3 PE=1 SV=2	11	25	2	6.49E+06	3.68E+06	5.38E+06	1.77E+06	3.85E+06	5.26E+06	2.48E+06	3.15E+06	5.13E+06	2.55E+06	4.17E+06	3.60E+06	4.40E+06	3.51E+06	1.25	0.33	0.293607572
P58107	Epiplakin OS=Homo sapiens OX=9606 GN=EPPK1 PE=1 SV=3	5	34	2	1.11E+06	7.30E+05	1.85E+06	1.10E+06	7.03E+05	1.82E+06	5.03E+06	2.50E+06	2.12E+06	2.07E+06	3.13E+06	3.26E+06	1.22E+06	3.02E+06	0.40	-1.31	0.008304223
Q01650	Large neutral amino acids transporter small subunit 1 OS=Homo sapiens OX=9606 GN=SLC7A5 PE=1 SV=2	6	34	2	5.84E+06	6.43E+06	7.64E+06	7.02E+06	6.73E+06	6.97E+06	3.22E+06	3.04E+06	3.40E+06	3.69E+06	2.48E+06	2.39E+06	6.77E+06	3.04E+06	2.23	1.16	5.65275E-07
P28161	Glutathione S-transferase Mu 2 OS=Homo sapiens OX=9606 GN=GSTM2 PE=1 SV=2	13	54	2	2.88E+07	2.43E+07	2.28E+07	2.08E+07	2.16E+07	2.31E+07	4.14E+07	3.48E+07	3.19E+07	2.76E+07	3.23E+07	3.26E+07	2.36E+07	3.34E+07	0.71	-0.50	0.001766335
P46778	60S ribosomal protein L21 OS=Homo sapiens OX=9606 GN=RPL21 PE=1 SV=2	14	50	2	2.27E+07	2.98E+07	3.00E+07	3.77E+07	2.95E+07	2.83E+07	1.55E+07	1.71E+07	1.72E+07	1.68E+07	1.32E+07	1.13E+07	2.97E+07	1.52E+07	1.96	0.97	0.000232639
Q13765	Nascent polypeptide-associated complex subunit alpha OS=Homo sapiens OX=9606 GN=NACA PE=1 SV=1	13	36	2	1.03E+07	1.14E+07	8.21E+06	1.21E+07	1.28E+07	1.03E+07	7.53E+06	7.70E+06	7.42E+06	7.13E+06	6.17E+06	5.83E+06	1.08E+07	6.96E+06	1.56	0.64	0.001031881
Q15286	Ras-related protein Rab-35 OS=Homo sapiens OX=9606 GN=RAB35 PE=1 SV=1	23	63	2	1.62E+06	1.57E+06	2.59E+06	2.60E+06	2.68E+06	2.96E+06	1.20E+06	1.56E+06	1.57E+06	2.25E+06	1.60E+06	1.15E+06	2.34E+06	1.56E+06	1.50	0.59	0.025175228
P21964	Catechol O-methyltransferase OS=Homo sapiens OX=9606 GN=COMT PE=1 SV=2	12	16	2	7.52E+05	5.21E+06	1.38E+06	3.17E+06	1.29E+06	5.96E+06	2.23E+06	8.44E+06	3.10E+06	5.18E+06	7.75E+06	6.81E+06	2.96E+06	5.59E+06	0.53	-0.92	0.084567692
P53999	Activated RNA polymerase II transcriptional coactivator p15 OS=Homo sapiens OX=9606 GN=SUB1 PE=1 SV=3	19	28	2	8.04E+06	6.16E+06	3.29E+06	6.73E+06	3.76E+06	6.25E+06	6.15E+06	6.27E+06	3.29E+06	1.57E+06	3.10E+06	4.00E+06	5.71E+06	4.06E+06	1.40	0.49	0.15204
O14617	AP-3 complex subunit delta-1 OS=Homo sapiens OX=9606 GN=AP3D1 PE=1 SV=1	3	32	2	6.69E+06	7.81E+06	6.39E+06	4.07E+06	5.34E+06	5.52E+06	3.17E+06	4.50E+06	4.22E+06	3.47E+06	4.84E+06	3.47E+06	5.97E+06	3.95E+06	1.51	0.60	0.010057096
Q16772	Glutathione S-transferase A3 OS=Homo sapiens OX=9606 GN=GSTA3 PE=1 SV=3	21	263	2	3.54E+07	4.39E+07	6.24E+07	6.21E+07	6.53E+07	6.47E+07	4.47E+07	5.02E+07	8.98E+07	8.96E+07	9.45E+07	7.06E+07	5.56E+07	7.32E+07	0.76	-0.40	0.124095599
P10301	Ras-related protein R-Ras OS=Homo sapiens OX=9606 GN=RRAS PE=1 SV=1	20	28	2	3.74E+06	4.33E+06	3.71E+06	4.14E+06	3.36E+06	4.89E+06	3.35E+06	2.13E+06	1.82E+06	2.75E+06	2.59E+06	1.56E+06	4.03E+06	2.36E+06	1.70	0.77	0.000838833
Q08170	Serine/arginine-rich splicing factor 4 OS=Homo sapiens OX=9606 GN=SRSF4 PE=1 SV=2	11	75	2	2.55E+07	2.80E+07	3.10E+07	3.31E+07	3.72E+07	3.08E+07	2.53E+07	2.62E+07	2.83E+07	2.63E+07	2.41E+07	2.37E+07	3.09E+07	2.57E+07	1.21	0.27	0.022541621
P13693	Translationally-controlled tumor protein OS=Homo sapiens OX=9606 GN=TPT1 PE=1 SV=1	31	10	2	4.54E+06	3.91E+06	3.52E+06	4.06E+06	1.68E+06	4.73E+06	1.96E+06	3.99E+06	3.09E+06	2.22E+06	6.05E+06	5.26E+06	3.74E+06	3.76E+06	0.99	-0.01	0.980630094
Q9HC07	Transmembrane protein 165 OS=Homo sapiens OX=9606 GN=TMEM165 PE=1 SV=1	14	18	2	6.87E+06	7.12E+06	5.89E+06	7.69E+06	7.63E+06	6.85E+06	5.41E+06	5.87E+06	7.03E+06	5.82E+06	7.84E+06	3.76E+06	7.01E+06	5.95E+06	1.18	0.24	0.139131246
Q6EMK4	Vasorin OS=Homo sapiens OX=9606 GN=VASN PE=1 SV=1	4	18	2	4.60E+06	3.95E+06	2.46E+06	3.63E+06	3.18E+06	3.00E+06	3.64E+06	4.95E+06	8.11E+05	1.64E+06	5.59E+05	2.76E+06	3.47E+06	2.39E+06	1.45	0.54	0.203006177
P48059	LIM and senescent cell antigen-like-containing domain protein 1 OS=Homo sapiens OX=9606 GN=LIMS1 PE=1 SV=4	12	46	2	1.12E+07	7.79E+06	7.85E+06	9.96E+06	9.95E+06	9.23E+06	1.31E+07	1.16E+07	1.58E+07	1.02E+07	1.67E+07	1.03E+07	9.33E+06	1.29E+07	0.72	-0.47	0.022645577
Q9BWD1	Acetyl-CoA acetyltransferase, cytosolic OS=Homo sapiens OX=9606 GN=ACAT2 PE=1 SV=2	13	6	2	2.76E+06	2.23E+06	1.64E+06	2.47E+06	1.71E+06	2.31E+06	4.73E+06	2.91E+06	1.23E+06	1.69E+06	1.10E+06	9.05E+05	2.19E+06	2.10E+06	1.04	0.06	0.889511115
P55809	Succinyl-CoA:3-ketoacid coenzyme A transferase 1, mitochondrial OS=Homo sapiens OX=9606 GN=OXCT1 PE=1 SV=1	9	10	2	3.56E+06	2.72E+06	2.61E+06	2.80E+06	2.33E+06	4.23E+06	2.57E+06	7.20E+05	1.48E+06	1.49E+06	2.42E+06	2.03E+06	3.04E+06	1.78E+06	1.71	0.77	0.01120172
P11310	Medium-chain specific acyl-CoA dehydrogenase, mitochondrial OS=Homo sapiens OX=9606 GN=ACADM PE=1 SV=1	10	14	2	1.59E+06	2.26E+06	1.85E+06	1.62E+06	1.50E+06	1.77E+06	3.89E+05	4.10E+05	9.44E+05	2.55E+05	8.95E+05	2.98E+05	1.76E+06	5.32E+05	3.32	1.73	2.66998E-05
Q9NRV9	Heme-binding protein 1 OS=Homo sapiens OX=9606 GN=HEBP1 PE=1 SV=1	17	10	2	4.40E+06	9.23E+05	2.82E+06	4.35E+06	3.96E+06	3.64E+06	6.30E+06	5.55E+06	4.23E+06	3.86E+06	4.06E+06	4.00E+06	3.35E+06	4.67E+06	0.72	-0.48	0.083183856
P31942	Heterogeneous nuclear ribonucleoprotein H3 OS=Homo sapiens OX=9606 GN=HNRNPH3 PE=1 SV=2	8	32	2	5.41E+06	4.94E+06	4.51E+06	4.36E+06	4.76E+06	3.97E+06	3.30E+06	2.78E+06	2.89E+06	3.07E+06	3.05E+06	1.86E+06	4.66E+06	2.82E+06	1.65	0.72	8.53264E-05
P35222	Catenin beta-1 OS=Homo sapiens OX=9606 GN=CTNNB1 PE=1 SV=1	6	52	2	4.48E+06	3.52E+06	3.39E+06	3.57E+06	5.25E+06	5.79E+06	2.76E+06	2.80E+06	4.04E+06	2.93E+06	2.78E+06	2.05E+06	4.33E+06	2.89E+06	1.50	0.58	0.017518357
Q15833	Syntaxin-binding protein 2 OS=Homo sapiens OX=9606 GN=STXBP2 PE=1 SV=2	6	32	2	4.64E+06	4.42E+06	4.54E+06	4.25E+06	3.87E+06	4.20E+06	5.12E+06	5.17E+06	3.30E+06	4.11E+06	3.56E+06	4.40E+06	4.32E+06	4.28E+06	1.01	0.01	0.902249295
P29966	Myristoylated alanine-rich C-kinase substrate OS=Homo sapiens OX=9606 GN=MARCKS PE=1 SV=4	12	18	2	2.29E+06	3.07E+06	6.00E+06	6.44E+06	4.80E+06	4.95E+06	4.49E+06	4.97E+06	5.67E+06	4.65E+06	4.08E+06	4.04E+06	4.59E+06	4.65E+06	0.99	-0.02	0.936491304
Q14520	Hyaluronan-binding protein 2 OS=Homo sapiens OX=9606 GN=HABP2 PE=1 SV=1	4	30	2	8.43E+06	8.76E+06	8.48E+06	1.31E+07	9.24E+06	4.91E+06	1.40E+07	6.73E+06	1.37E+07	1.13E+07	8.29E+05	3.31E+06	8.82E+06	8.29E+06	1.06	0.09	0.839489266
Q13247	Serine/arginine-rich splicing factor 6 OS=Homo sapiens OX=9606 GN=SRSF6 PE=1 SV=2	15	51	2	4.53E+06	5.09E+06	4.19E+06	4.62E+06	4.02E+06	3.95E+06	4.84E+06	4.42E+06	3.99E+06	4.59E+06	3.83E+06	3.51E+06	4.40E+06	4.20E+06	1.05	0.07	0.466719361
P22105	Tenascin-X OS=Homo sapiens OX=9606 GN=TNXB PE=1 SV=5	2	34	2	4.40E+06	4.90E+06	6.75E+06	6.34E+06	4.84E+06	5.15E+06		7.79E+06	7.59E+06	7.40E+06	6.61E+06	6.81E+06	5.40E+06	7.24E+06	0.75	-0.42	0.003179203
P60903	Protein S100-A10 OS=Homo sapiens OX=9606 GN=S100A10 PE=1 SV=2	28	47	2	1.54E+07	1.90E+07	1.29E+07	1.63E+07	1.48E+07	1.13E+07	9.55E+06	1.09E+07	5.99E+06	1.01E+07	6.56E+06	7.49E+06	1.49E+07	8.42E+06	1.77	0.83	0.00090011
P02042	Hemoglobin subunit delta OS=Homo sapiens OX=9606 GN=HBD PE=1 SV=2	13	1536	2	1.17E+09	1.12E+09	1.00E+09	9.20E+08	1.15E+09	1.11E+09	1.24E+09	1.28E+09	1.23E+09	1.36E+09	1.24E+09	1.43E+09	1.08E+09	1.30E+09	0.83	-0.26	0.001988061
P19827	Inter-alpha-trypsin inhibitor heavy chain H1 OS=Homo sapiens OX=9606 GN=ITIH1 PE=1 SV=3	3	28	2	3.22E+06	3.37E+06	5.34E+06	6.63E+06	2.51E+06	5.38E+06	7.86E+06	7.66E+06	3.15E+06	6.84E+06	6.66E+06	5.64E+06	4.41E+06	6.30E+06	0.70	-0.52	0.078461488
P48147	Prolyl endopeptidase OS=Homo sapiens OX=9606 GN=PREP PE=1 SV=2	4	37	2	3.93E+06	4.10E+06	2.74E+06	2.99E+06	2.92E+06	4.78E+06	4.01E+06	4.84E+06	3.66E+06	3.73E+06	3.99E+06	4.24E+06	3.57E+06	4.08E+06	0.88	-0.19	0.219969684
P52565	Rho GDP-dissociation inhibitor 1 OS=Homo sapiens OX=9606 GN=ARHGDIA PE=1 SV=3	16	28	2	4.13E+06	4.81E+06	6.03E+06	4.53E+06	5.70E+06	4.80E+06	3.62E+06	3.84E+06	4.07E+06	3.88E+06	2.84E+06	5.55E+06	5.00E+06	3.97E+06	1.26	0.33	0.052468929
Q01844	RNA-binding protein EWS OS=Homo sapiens OX=9606 GN=EWSR1 PE=1 SV=1	4	26	2	7.39E+06	7.37E+06	8.02E+06	7.27E+06	6.40E+06	6.57E+06	3.99E+06	4.55E+06	3.26E+06	4.09E+06	3.52E+06	3.46E+06	7.17E+06	3.81E+06	1.88	0.91	1.18302E-06
Q09028	Histone-binding protein RBBP4 OS=Homo sapiens OX=9606 GN=RBBP4 PE=1 SV=3	16	41	2			2.95E+06	2.93E+06		1.37E+06				1.49E+06	2.49E+06		2.42E+06	1.99E+06	1.22	0.28	0.599353373
P28065	Proteasome subunit beta type-9 OS=Homo sapiens OX=9606 GN=PSMB9 PE=1 SV=2	14	20	2	3.83E+06	3.88E+06	3.01E+06	3.84E+06	3.44E+06	3.53E+06	3.62E+06	4.36E+06	3.17E+06	3.74E+06	3.36E+06	1.18E+06	3.59E+06	3.24E+06	1.11	0.15	0.480994747
Q96N76	Urocanate hydratase OS=Homo sapiens OX=9606 GN=UROC1 PE=1 SV=1	5	14	2	7.91E+05	8.26E+05	1.86E+06	1.30E+06	1.44E+06	8.62E+05	4.06E+06	1.94E+06	3.61E+06	3.11E+06	3.03E+06	1.51E+06	1.18E+06	2.88E+06	0.41	-1.29	0.006216674
P23258	Tubulin gamma-1 chain OS=Homo sapiens OX=9606 GN=TUBG1 PE=1 SV=2	11	8	2	6.33E+05	9.53E+05	2.14E+06	9.58E+05	1.75E+06	3.56E+06	6.86E+05	3.61E+05		4.35E+05			1.13E+06	4.94E+05	2.29	1.19	0.072918266
Q14112	Nidogen-2 OS=Homo sapiens OX=9606 GN=NID2 PE=1 SV=3	3	6	2	6.45E+05		1.88E+06	1.28E+06	5.37E+05		1.71E+06		4.62E+05	5.83E+05	6.67E+05	1.99E+06	1.08E+06	1.08E+06	1.00	0.00	0.995317394
P15531	Nucleoside diphosphate kinase A OS=Homo sapiens OX=9606 GN=NME1 PE=1 SV=1	30	32	2	6.10E+06	7.47E+06	7.81E+06	9.56E+06	8.11E+06	8.98E+06	8.61E+06	8.24E+06	7.75E+06	7.02E+06	7.72E+06	6.69E+06	8.01E+06	7.67E+06	1.04	0.06	0.574551007
P07203	Glutathione peroxidase 1 OS=Homo sapiens OX=9606 GN=GPX1 PE=1 SV=4	18	21	2	1.48E+06	2.98E+06	2.97E+06	4.07E+06	2.45E+06	2.23E+06	5.10E+06	5.03E+06	5.28E+06	3.75E+06	4.42E+06	2.97E+06	2.70E+06	4.42E+06	0.61	-0.71	0.007129705
Q9BXN1	Asporin OS=Homo sapiens OX=9606 GN=ASPN PE=1 SV=2	13	4	2	1.57E+06	5.51E+05		6.24E+05		9.06E+05	1.63E+06	1.43E+06		1.22E+06	5.88E+05	6.97E+05	9.12E+05	1.11E+06	0.82	-0.29	0.538505832
Q9BZQ8	Protein Niban 1 OS=Homo sapiens OX=9606 GN=NIBAN1 PE=1 SV=1	4	16	2	2.94E+06	1.48E+06	2.01E+06	2.53E+06	2.11E+06	2.42E+06	2.82E+06	2.21E+06	2.32E+06	2.62E+06	2.07E+06	1.51E+06	2.25E+06	2.26E+06	1.00	-0.01	0.973296315
Q96DI7	U5 small nuclear ribonucleoprotein 40 kDa protein OS=Homo sapiens OX=9606 GN=SNRNP40 PE=1 SV=1	11	8	2		1.37E+06	6.47E+05			2.75E+06	5.11E+05	7.03E+05		6.81E+05	1.06E+06	4.48E+05	1.59E+06	6.81E+05	2.33	1.22	0.277857593
P32754	4-hydroxyphenylpyruvate dioxygenase OS=Homo sapiens OX=9606 GN=HPD PE=1 SV=2	9	18	2	2.93E+06	2.99E+06	2.78E+06	3.08E+06	2.49E+06	2.97E+06	4.76E+06	4.71E+06	4.54E+06	3.45E+06	3.89E+06	2.53E+06	2.87E+06	3.98E+06	0.72	-0.47	0.02561566
Q96T76	MMS19 nucleotide excision repair protein homolog OS=Homo sapiens OX=9606 GN=MMS19 PE=1 SV=2	4	6	2	1.08E+06	2.00E+06	1.48E+06	1.77E+06	1.45E+06	1.58E+06	1.25E+06	1.52E+06	1.36E+06	8.46E+05	1.09E+06	7.99E+05	1.56E+06	1.14E+06	1.36	0.45	0.037089734
O14763	Tumor necrosis factor receptor superfamily member 10B OS=Homo sapiens OX=9606 GN=TNFRSF10B PE=1 SV=2	7	17	2	7.29E+06	4.71E+06	5.46E+06	4.62E+06	3.90E+06	4.62E+06	3.38E+06	2.75E+06	2.96E+06	2.33E+06	3.67E+06	3.18E+06	5.10E+06	3.05E+06	1.67	0.74	0.006265415
P68402	Platelet-activating factor acetylhydrolase IB subunit beta OS=Homo sapiens OX=9606 GN=PAFAH1B2 PE=1 SV=1	19	6	2				8.67E+05	1.03E+06		1.02E+06	1.32E+06	1.18E+06		1.75E+06	7.05E+05	9.47E+05	1.20E+06	0.79	-0.34	0.246945279
P07954	Fumarate hydratase, mitochondrial OS=Homo sapiens OX=9606 GN=FH PE=1 SV=3	7	18	2	6.53E+06	7.77E+06	4.23E+06	5.83E+06	5.59E+06	6.40E+06	1.00E+07	8.59E+06	7.71E+06	7.69E+06	7.55E+06	7.02E+06	6.06E+06	8.10E+06	0.75	-0.42	0.010547801
P20645	Cation-dependent mannose-6-phosphate receptor OS=Homo sapiens OX=9606 GN=M6PR PE=1 SV=1	12	42	2	5.35E+06	5.03E+06	3.93E+06	4.41E+06	3.92E+06	4.58E+06	4.56E+06	4.01E+06	4.26E+06	3.58E+06	3.90E+06	2.38E+06	4.54E+06	3.78E+06	1.20	0.26	0.084349745
P04637	Cellular tumor antigen p53 OS=Homo sapiens OX=9606 GN=TP53 PE=1 SV=4	9	17	2	3.95E+06	5.32E+06	4.00E+06	3.69E+06	3.02E+06	5.58E+06	2.95E+06	3.14E+06	1.68E+06	2.14E+06	2.56E+06	6.86E+05	4.26E+06	2.19E+06	1.94	0.96	0.003724725
P61081	NEDD8-conjugating enzyme Ubc12 OS=Homo sapiens OX=9606 GN=UBE2M PE=1 SV=1	15	16	2	2.91E+06	2.68E+06	1.92E+06	2.43E+06	2.85E+06	3.30E+06	2.90E+06	2.40E+06	2.34E+06	3.00E+06	2.19E+06	1.91E+06	2.68E+06	2.46E+06	1.09	0.13	0.403916909
P47914	60S ribosomal protein L29 OS=Homo sapiens OX=9606 GN=RPL29 PE=1 SV=2	14	30	2	1.21E+07	1.20E+07	1.28E+07	1.45E+07	1.19E+07	1.24E+07	7.23E+06	7.29E+06	5.61E+06	7.08E+06	6.66E+06	6.18E+06	1.26E+07	6.68E+06	1.89	0.92	7.07417E-07
O15127	Secretory carrier-associated membrane protein 2 OS=Homo sapiens OX=9606 GN=SCAMP2 PE=1 SV=2	10	4	2	2.76E+06	2.60E+06	2.88E+06	2.68E+06	3.05E+06	3.01E+06	2.28E+06	2.11E+06	1.92E+06		2.11E+06	1.83E+06	2.83E+06	2.05E+06	1.38	0.47	6.17135E-05
P50851	Lipopolysaccharide-responsive and beige-like anchor protein OS=Homo sapiens OX=9606 GN=LRBA PE=1 SV=4	1	4	2	1.44E+06	7.10E+05			5.52E+05	5.34E+05	1.15E+06		4.95E+05	7.81E+05	3.82E+05	3.61E+05	8.10E+05	6.34E+05	1.28	0.35	0.528086604
Q9UI10	Translation initiation factor eIF-2B subunit delta OS=Homo sapiens OX=9606 GN=EIF2B4 PE=1 SV=2	9	6	2	3.14E+06		1.79E+06	1.94E+06	2.70E+06								2.39E+06				
Q9H0D6	5'-3' exoribonuclease 2 OS=Homo sapiens OX=9606 GN=XRN2 PE=1 SV=1	3	14	2	4.29E+06	4.27E+06	3.79E+06	3.75E+06	8.78E+05	1.98E+06	2.98E+06	2.93E+06	2.27E+06	1.41E+06	3.69E+06	2.01E+06	3.16E+06	2.55E+06	1.24	0.31	0.382087325
Q9UIA9	Exportin-7 OS=Homo sapiens OX=9606 GN=XPO7 PE=1 SV=3	3	18	2	2.70E+06	2.98E+06	3.04E+06	2.32E+06	3.76E+06	2.99E+06	4.10E+06	1.69E+06	3.32E+06	2.46E+06	3.52E+06	2.52E+06	2.97E+06	2.94E+06	1.01	0.01	0.942642798
Q6XQN6	Nicotinate phosphoribosyltransferase OS=Homo sapiens OX=9606 GN=NAPRT PE=1 SV=2	6	20	2	4.82E+06	2.34E+06	2.24E+06	3.09E+06	2.03E+06	2.89E+06	4.96E+06	4.31E+06	1.21E+06	1.89E+06	2.36E+06	2.54E+06	2.90E+06	2.88E+06	1.01	0.01	0.974287847
P27824	Calnexin OS=Homo sapiens OX=9606 GN=CANX PE=1 SV=2	5	12	2	1.27E+06	1.33E+06	2.15E+06	2.26E+06	1.66E+06	1.91E+06	1.10E+06		5.60E+05	5.43E+05	8.90E+05	5.42E+05	1.76E+06	7.28E+05	2.42	1.28	0.000847091
P30086	Phosphatidylethanolamine-binding protein 1 OS=Homo sapiens OX=9606 GN=PEBP1 PE=1 SV=3	15	24	2	7.72E+06	6.61E+06	7.36E+06	9.24E+06	7.41E+06	6.86E+06	8.16E+06	8.99E+06	9.48E+06	9.81E+06	9.56E+06	8.31E+06	7.53E+06	9.05E+06	0.83	-0.27	0.010091167
Q9C005	Protein dpy-30 homolog OS=Homo sapiens OX=9606 GN=DPY30 PE=1 SV=1	36	20	2	2.12E+06	1.66E+06	3.49E+06	3.40E+06	2.11E+06	1.64E+06	3.13E+06	8.72E+05	1.69E+06	1.82E+06	1.52E+06	5.19E+05	2.40E+06	1.59E+06	1.51	0.59	0.137075982
Q5VW32	BRO1 domain-containing protein BROX OS=Homo sapiens OX=9606 GN=BROX PE=1 SV=1	10	16	2	1.80E+06	2.31E+06	2.54E+06	2.00E+06	2.40E+06	2.06E+06	1.77E+06	1.73E+06	1.26E+06	1.44E+06	1.70E+06	1.49E+06	2.19E+06	1.57E+06	1.40	0.48	0.001555842
Q8WWI5	Choline transporter-like protein 1 OS=Homo sapiens OX=9606 GN=SLC44A1 PE=1 SV=1	4	40	2	7.70E+06	8.24E+06	4.04E+06	5.64E+06	5.93E+06	5.33E+06	5.94E+06	5.93E+06	2.77E+06	3.89E+06	3.81E+06	4.38E+06	6.15E+06	4.45E+06	1.38	0.46	0.066931701
Q92544	Transmembrane 9 superfamily member 4 OS=Homo sapiens OX=9606 GN=TM9SF4 PE=1 SV=2	6	16	2	4.61E+06	2.15E+06	2.70E+06	2.03E+06	1.10E+06	2.30E+06	1.55E+06	1.52E+06	1.60E+06	6.96E+05	1.75E+06	1.63E+06	2.48E+06	1.46E+06	1.70	0.77	0.087713963
Q9UJ70	N-acetyl-D-glucosamine kinase OS=Homo sapiens OX=9606 GN=NAGK PE=1 SV=4	7	20	2	2.15E+06	2.52E+06	2.20E+06	2.20E+06	2.44E+06	2.28E+06	7.29E+05	2.60E+06	2.12E+06	2.09E+06	1.16E+06	1.87E+06	2.30E+06	1.76E+06	1.31	0.39	0.115645377
Q16739	Ceramide glucosyltransferase OS=Homo sapiens OX=9606 GN=UGCG PE=1 SV=1	7	6	2	2.26E+06	2.70E+06	1.44E+06	2.18E+06	8.03E+05	2.13E+06	5.61E+05	1.69E+06	4.32E+05		1.51E+06	2.71E+05	1.92E+06	8.92E+05	2.15	1.11	0.032208519
Q9Y295	Developmentally-regulated GTP-binding protein 1 OS=Homo sapiens OX=9606 GN=DRG1 PE=1 SV=1	11	6	2	1.33E+06	1.52E+06	1.18E+06	1.30E+06	1.01E+06	9.53E+05			7.79E+05			1.08E+06	1.22E+06	9.31E+05	1.31	0.38	0.265019588
P00750	Tissue-type plasminogen activator OS=Homo sapiens OX=9606 GN=PLAT PE=1 SV=1	6	4	2	1.55E+06	2.11E+06	1.91E+06	1.79E+06	1.48E+06	1.55E+06	2.15E+06	2.35E+06	2.12E+06	2.09E+06	1.43E+06	1.54E+06	1.73E+06	1.95E+06	0.89	-0.17	0.26612819
Q14103	Heterogeneous nuclear ribonucleoprotein D0 OS=Homo sapiens OX=9606 GN=HNRNPD PE=1 SV=1	8	76	2	1.85E+07	2.07E+07	2.27E+07	1.96E+07	1.95E+07	2.05E+07	1.66E+07	1.49E+07	1.02E+07	1.08E+07	1.49E+07	1.11E+07	2.03E+07	1.31E+07	1.55	0.63	0.000503618
P10909	Clusterin OS=Homo sapiens OX=9606 GN=CLU PE=1 SV=1	8	29	2	6.61E+06	4.29E+06	2.88E+06	2.10E+06	4.92E+06	4.03E+06	7.13E+06	6.44E+06	5.49E+06	3.84E+06	3.55E+06	1.31E+07	4.14E+06	6.59E+06	0.63	-0.67	0.160151503
P13797	Plastin-3 OS=Homo sapiens OX=9606 GN=PLS3 PE=1 SV=4	9	23	2	2.54E+06	2.84E+06	4.43E+06	4.71E+06	1.03E+06	1.59E+06	2.42E+06	1.48E+06	1.45E+06	3.81E+06	4.31E+06	1.53E+06	2.86E+06	2.50E+06	1.14	0.19	0.664528672
Q14699	Raftlin OS=Homo sapiens OX=9606 GN=RFTN1 PE=1 SV=4	6	4	2	2.35E+06	4.91E+05	1.81E+06	1.07E+06	1.36E+06	1.57E+06		6.41E+05	1.04E+06	1.18E+06	5.33E+05	4.25E+05	1.44E+06	7.63E+05	1.89	0.92	0.05350748
P51608	Methyl-CpG-binding protein 2 OS=Homo sapiens OX=9606 GN=MECP2 PE=1 SV=1	7	26	2	5.35E+06	4.72E+06	4.04E+06	3.46E+06	2.94E+06	4.17E+06	2.18E+06	2.97E+06	2.08E+06	2.89E+06	2.85E+06	2.55E+06	4.11E+06	2.59E+06	1.59	0.67	0.005574675
Q9P2E9	Ribosome-binding protein 1 OS=Homo sapiens OX=9606 GN=RRBP1 PE=1 SV=5	2	24	2	1.99E+06	2.82E+06	3.23E+06	4.05E+06	3.20E+06	3.47E+06	2.02E+06	1.18E+06	2.15E+06	1.39E+06	8.80E+05	1.13E+06	3.13E+06	1.46E+06	2.14	1.10	0.000961755
O14817	Tetraspanin-4 OS=Homo sapiens OX=9606 GN=TSPAN4 PE=1 SV=1	14	8	2	2.87E+06	2.75E+06	2.00E+06		3.28E+06	2.60E+06	2.65E+06	3.56E+06	3.39E+06		3.05E+06	2.58E+06	2.70E+06	3.05E+06	0.89	-0.17	0.25665275
Q92859	Neogenin OS=Homo sapiens OX=9606 GN=NEO1 PE=1 SV=2	2	4	2	2.91E+06	3.33E+06	1.68E+06	3.49E+06	2.49E+06	2.20E+06	3.16E+06	4.01E+06	3.47E+06	3.06E+06	2.64E+06	3.18E+06	2.68E+06	3.26E+06	0.82	-0.28	0.12702878
O75874	Isocitrate dehydrogenase [NADP] cytoplasmic OS=Homo sapiens OX=9606 GN=IDH1 PE=1 SV=2	8	26	2	3.60E+06	3.88E+06	4.37E+06	3.83E+06	3.84E+06	5.83E+06	4.92E+06	4.88E+06	3.06E+06	4.10E+06	5.61E+06	3.94E+06	4.22E+06	4.42E+06	0.96	-0.06	0.706768167
Q9NVJ2	ADP-ribosylation factor-like protein 8B OS=Homo sapiens OX=9606 GN=ARL8B PE=1 SV=1	14	14	2	3.93E+06	6.29E+06	4.90E+06	4.42E+06	3.13E+06	6.22E+06	3.00E+06	3.34E+06	2.76E+06	2.44E+06	3.78E+06	2.79E+06	4.82E+06	3.02E+06	1.60	0.67	0.015670394

Supplementary Table 4. List of all proteins identified by label free proteomics analysis.

Accession	Description	Coverage [%]	# PSMs	# Unique Peptides	Amitriptyline 1	Amitriptyline 2	Amitriptyline 3	Amitriptyline 4	Amitriptyline 5	Amitriptyline 6	Vehicle 1	Vehicle 2	Vehicle 3	Vehicle 4	Vehicle 5	Vehicle 6	Average amitriptyline	Average vehicle	Ratio amitriptyline/ vehicle	Log 2 fold change (amitriptyline /vehicle)	P-value
P08237	ATP-dependent 6-phosphofructokinase, muscle type OS=Homo sapiens OX=9606 GN=PFKM PE=1 SV=2	4	31	2	3.36E+06	3.55E+06	3.32E+06	3.58E+06	3.29E+06	2.64E+06	3.18E+06	3.14E+06	3.20E+06	3.29E+06	3.08E+06	2.54E+06	3.29E+06	3.07E+06	1.07	0.10	0.248096493
P49888	Sulfotransferase 1E1 OS=Homo sapiens OX=9606 GN=SULT1E1 PE=1 SV=1	6	70	2	1.12E+07	1.10E+07	2.36E+07	2.45E+07	2.68E+07	1.84E+07	2.86E+07	2.99E+07	4.45E+07	3.68E+07	2.91E+07	2.73E+07	1.92E+07	3.27E+07	0.59	-0.76	0.006451522
Q92974	Rho guanine nucleotide exchange factor 2 OS=Homo sapiens OX=9606 GN=ARHGEF2 PE=1 SV=4	3	6	2		4.36E+05	1.83E+06	1.48E+06	9.46E+05	2.16E+06	1.24E+06	3.49E+05	6.85E+05	4.71E+05	3.60E+05	7.24E+05	1.37E+06	6.38E+05	2.15	1.10	0.076597122
O60664	Perilipin-3 OS=Homo sapiens OX=9606 GN=PLIN3 PE=1 SV=3	6	18	2	2.96E+06	2.74E+06	2.63E+06	3.40E+06	2.92E+06	2.30E+06	2.02E+06	1.29E+06	9.25E+05	1.41E+06	1.40E+06	1.20E+06	2.82E+06	1.37E+06	2.06	1.04	4.33151E-05
P23468	Receptor-type tyrosine-protein phosphatase delta OS=Homo sapiens OX=9606 GN=PTPRD PE=1 SV=2	1	26	2	2.83E+06	2.39E+06	3.21E+06	2.65E+06	2.95E+06	1.93E+06	3.83E+06	3.83E+06	3.17E+06	2.58E+06	3.22E+06	2.98E+06	2.66E+06	3.27E+06	0.81	-0.30	0.049632035
O95297	Myelin protein zero-like protein 1 OS=Homo sapiens OX=9606 GN=MPZL1 PE=1 SV=1	10	18	2	2.53E+06	2.47E+06	3.28E+06	3.67E+06	3.31E+06	2.95E+06	1.31E+06	1.33E+06	1.75E+06	1.40E+06	1.09E+06	1.34E+06	3.03E+06	1.37E+06	2.21	1.15	0.000108535
P60866	40S ribosomal protein S20 OS=Homo sapiens OX=9606 GN=RPS20 PE=1 SV=1	19	54	2	1.83E+07	1.75E+07	1.57E+07	1.92E+07	1.64E+07	1.70E+07	1.26E+07	1.42E+07	1.47E+07	1.10E+07	1.11E+07	1.60E+07	1.73E+07	1.33E+07	1.31	0.39	0.002775952
P52272	Heterogeneous nuclear ribonucleoprotein M OS=Homo sapiens OX=9606 GN=HNRNPM PE=1 SV=3	3	34	2	1.05E+07	1.20E+07	7.04E+06	8.88E+06	6.65E+06	7.03E+06	6.09E+06	5.54E+06	3.63E+06	3.48E+06	3.90E+06	3.82E+06	8.68E+06	4.41E+06	1.97	0.98	0.003213492
Q16836	Hydroxyacyl-coenzyme A dehydrogenase, mitochondrial OS=Homo sapiens OX=9606 GN=HADH PE=1 SV=3	10	22	2	3.70E+06	4.23E+06	4.87E+06	2.96E+06	4.27E+06	4.96E+06	3.84E+06	4.09E+06	4.18E+06	4.14E+06	4.20E+06	4.48E+06	4.16E+06	4.15E+06	1.00	0.00	0.978286341
P13489	Ribonuclease inhibitor OS=Homo sapiens OX=9606 GN=RNH1 PE=1 SV=2	6	12	2	3.03E+06	3.61E+06	2.81E+06	3.12E+06	3.87E+06	2.69E+06	9.20E+05	1.65E+06	1.67E+06	1.90E+06	9.40E+05	1.42E+06	3.19E+06	1.42E+06	2.25	1.17	3.7909E-05
Q14444	Caprin-1 OS=Homo sapiens OX=9606 GN=CAPRIN1 PE=1 SV=2	5	8	2	2.74E+06	1.99E+06	3.38E+06	2.83E+06	3.38E+06	3.04E+06	2.46E+06	1.79E+06	2.11E+06	1.20E+06	1.91E+06	1.54E+06	2.89E+06	1.83E+06	1.58	0.66	0.003485861
P35637	RNA-binding protein FUS OS=Homo sapiens OX=9606 GN=FUS PE=1 SV=1	6	14	2	5.74E+06	5.13E+06	4.98E+06	4.81E+06	4.08E+06	3.61E+06	2.27E+06	1.56E+06	1.70E+06	1.85E+06	1.76E+06	1.12E+06	4.72E+06	1.71E+06	2.76	1.47	4.28385E-05
Q7RTV2	Glutathione S-transferase A5 OS=Homo sapiens OX=9606 GN=GSTA5 PE=1 SV=1	7	270	2	5.97E+07	5.78E+07	5.22E+07	5.68E+07	5.27E+07	4.54E+07	7.24E+07	6.94E+07	7.65E+07	8.07E+07	6.28E+07	4.92E+07	5.41E+07	6.85E+07	0.79	-0.34	0.024471466
Q8TBC4	NEDD8-activating enzyme E1 catalytic subunit OS=Homo sapiens OX=9606 GN=UBA3 PE=1 SV=2	9	13	2		1.03E+06	7.96E+05	3.26E+06	8.38E+05	2.29E+06	5.20E+05	6.24E+05	1.75E+06	1.76E+06	3.05E+06	1.33E+06	1.64E+06	1.51E+06	1.09	0.12	0.831342996
P61966	AP-1 complex subunit sigma-1A OS=Homo sapiens OX=9606 GN=AP1S1 PE=1 SV=1	15	5	2																	
Q7L2E3	ATP-dependent RNA helicase DHX30 OS=Homo sapiens OX=9606 GN=DHX30 PE=1 SV=1	4	4	2		3.88E+05	1.56E+06	2.77E+05	1.44E+06	8.54E+05	1.02E+06	3.29E+05	4.23E+05	3.67E+05	5.17E+05	5.50E+05	9.04E+05	5.35E+05	1.69	0.76	0.245743931
O14879	Interferon-induced protein with tetratricopeptide repeats 3 OS=Homo sapiens OX=9606 GN=IFIT3 PE=1 SV=1	5	6	2		1.43E+06	1.18E+06	1.58E+06	2.09E+06	1.35E+06			5.32E+05			9.50E+05	1.53E+06	7.41E+05	2.06	1.04	0.082308324
P46060	Ran GTPase-activating protein 1 OS=Homo sapiens OX=9606 GN=RANGAP1 PE=1 SV=1	4	4	2	2.40E+06	3.19E+06	1.62E+06	2.89E+06	2.75E+06	1.33E+06	1.96E+06	6.28E+05	1.63E+06	1.49E+06	9.12E+05	5.74E+05	2.37E+06	1.20E+06	1.97	0.98	0.012914068
P35244	Replication protein A 14 kDa subunit OS=Homo sapiens OX=9606 GN=RPA3 PE=1 SV=1	30	13	2	4.84E+06	2.05E+06	1.42E+06	1.85E+06	1.62E+06	2.12E+06	2.84E+06	1.86E+06	1.48E+06	1.58E+06	1.56E+06	1.36E+06	2.31E+06	1.78E+06	1.30	0.38	0.373051958
P11233	Ras-related protein Ral-A OS=Homo sapiens OX=9606 GN=RALA PE=1 SV=1	19	8	2	1.40E+06	1.95E+06	2.30E+06	2.06E+06	1.39E+06	2.13E+06	1.34E+06	1.04E+06	7.84E+05		1.83E+06	6.05E+05	1.87E+06	1.12E+06	1.67	0.74	0.024126592
P46783	40S ribosomal protein S10 OS=Homo sapiens OX=9606 GN=RPS10 PE=1 SV=1	15	58	2	1.40E+07	9.03E+06	5.81E+06	1.05E+07	6.21E+06	1.02E+07	1.30E+07	5.92E+06	6.69E+06	3.93E+06	5.19E+06	5.44E+06	9.30E+06	6.69E+06	1.39	0.47	0.180156815
O75821	Eukaryotic translation initiation factor 3 subunit G OS=Homo sapiens OX=9606 GN=EIF3G PE=1 SV=2	13	6	2	1.42E+06	5.93E+05	1.07E+06	1.53E+06	1.20E+06	1.87E+06	7.54E+05	4.96E+05	6.39E+05	3.85E+05	6.48E+05	6.40E+05	1.28E+06	5.94E+05	2.16	1.11	0.01045088
Q99439	Calponin-2 OS=Homo sapiens OX=9606 GN=CNN2 PE=1 SV=4	13	25	2	7.21E+06	6.48E+06	6.20E+06	6.41E+06	6.43E+06	5.39E+06	7.33E+06	3.22E+06	3.80E+06	3.37E+06	4.59E+06	4.59E+06	6.35E+06	4.48E+06	1.42	0.50	0.028127179
P22694	cAMP-dependent protein kinase catalytic subunit beta OS=Homo sapiens OX=9606 GN=PRKACB PE=1 SV=2	9	10	2	2.68E+06	2.59E+06	2.14E+06	1.39E+06	1.04E+06	1.82E+06	3.33E+06	3.16E+06	3.04E+06	1.56E+06	3.15E+06	1.97E+06	1.95E+06	2.70E+06	0.72	-0.47	0.091664469
P42167	Lamina-associated polypeptide 2, isoforms beta/gamma OS=Homo sapiens OX=9606 GN=TMPO PE=1 SV=2	9	12	2	1.81E+06	4.74E+06	4.73E+06	5.04E+06	1.91E+06	1.74E+06	4.04E+06	4.56E+06	9.86E+05	4.55E+06	9.12E+05	3.37E+06	3.33E+06	3.07E+06	1.08	0.12	0.795342912
P62847	40S ribosomal protein S24 OS=Homo sapiens OX=9606 GN=RPS24 PE=1 SV=1	20	53	2	1.47E+07	1.35E+07	8.13E+06	1.12E+07	1.06E+07	1.10E+07	8.79E+06	1.03E+07	4.09E+06	4.07E+06	6.75E+06	5.43E+06	1.15E+07	6.57E+06	1.75	0.81	0.005679177
Q12805	EGF-containing fibulin-like extracellular matrix protein 1 OS=Homo sapiens OX=9606 GN=EFEMP1 PE=1 SV=2	7	38	2	6.75E+06	5.72E+06	5.52E+06	5.18E+06	5.93E+06	5.52E+06	9.72E+06	7.99E+06	9.32E+06	7.23E+06	8.43E+06	7.68E+06	5.77E+06	8.39E+06	0.69	-0.54	0.000419632
O75436	Vacuolar protein sorting-associated protein 26A OS=Homo sapiens OX=9606 GN=VPS26A PE=1 SV=2	9	7	2	2.20E+06	3.25E+06	2.25E+06	2.56E+06	3.64E+06	2.60E+06	1.27E+06		5.58E+05	1.91E+06	4.91E+05	1.57E+06	2.75E+06	1.16E+06	2.37	1.25	0.002173935
Q9UHB6	LIM domain and actin-binding protein 1 OS=Homo sapiens OX=9606 GN=LIMA1 PE=1 SV=1	3	8	2	1.71E+06	3.05E+06	3.19E+06	3.00E+06	2.93E+06	2.57E+06			1.16E+06	1.03E+06	1.13E+06	8.99E+05	2.74E+06	1.05E+06	2.61	1.38	0.000417889
Q15637	Splicing factor 1 OS=Homo sapiens OX=9606 GN=SF1 PE=1 SV=4	5	10	2		4.52E+05	2.67E+06	6.63E+05	1.91E+06	9.01E+05	3.24E+05	5.89E+05	4.22E+05	1.23E+06	8.76E+05	5.05E+05	1.32E+06	6.58E+05	2.01	1.00	0.196465508
P01116	GTPase KRas OS=Homo sapiens OX=9606 GN=KRAS PE=1 SV=1	20	4	2	5.94E+06	5.75E+06	3.17E+06	3.74E+06	3.16E+06	6.39E+06	4.13E+06	5.64E+06	2.71E+06	3.31E+06	2.80E+06	3.38E+06	4.69E+06	3.66E+06	1.28	0.36	0.205712945
Q9Y2V2	Calcium-regulated heat-stable protein 1 OS=Homo sapiens OX=9606 GN=CARHSP1 PE=1 SV=2	18	33	2	3.58E+06	5.70E+06	5.02E+06	6.87E+06	5.12E+06	4.47E+06	4.94E+06	5.38E+06	4.99E+06	3.25E+06	5.12E+06	4.48E+06	5.13E+06	4.69E+06	1.09	0.13	0.451481331
P62306	Small nuclear ribonucleoprotein F OS=Homo sapiens OX=9606 GN=SNRPF PE=1 SV=1	24	28	2	6.90E+06	5.69E+06	4.66E+06	5.54E+06	4.52E+06	5.70E+06	4.08E+06	4.06E+06	3.17E+06	4.53E+06	3.65E+06	2.24E+06	5.50E+06	3.62E+06	1.52	0.60	0.003112069
O95747	Serine/threonine-protein kinase OSR1 OS=Homo sapiens OX=9606 GN=OXSR1 PE=1 SV=1	8	3	2				2.38E+05		8.58E+05	3.47E+05						5.48E+05	3.47E+05	1.58	0.66	
O43149	Zinc finger ZZ-type and EF-hand domain-containing protein 1 OS=Homo sapiens OX=9606 GN=ZZEF1 PE=1 SV=6	1	8	2	4.25E+06	4.57E+06					9.68E+05		6.27E+06	4.77E+06			4.41E+06	4.00E+06	1.10	0.14	0.821824898
P35080	Profilin-2 OS=Homo sapiens OX=9606 GN=PFN2 PE=1 SV=3	27	8	2	2.41E+06	3.90E+05	2.63E+06	1.91E+06	1.89E+06	1.48E+06	5.44E+05	1.79E+06	1.36E+06	5.26E+05	1.87E+06		1.78E+06	1.22E+06	1.46	0.55	0.228073902
P23919	Thymidylate kinase OS=Homo sapiens OX=9606 GN=DTYMK PE=1 SV=4	11	6	2	2.94E+06	4.06E+06	2.44E+06	3.19E+06	1.38E+06	2.23E+06	8.28E+05	9.57E+05	7.09E+05	1.12E+06	9.84E+05		2.71E+06	9.19E+05	2.95	1.56	0.004428635
P55735	Protein SEC13 homolog OS=Homo sapiens OX=9606 GN=SEC13 PE=1 SV=3	7	25	2	5.31E+06	4.51E+06	5.39E+06	5.92E+06	5.09E+06	4.94E+06	3.58E+06	2.43E+06	3.88E+06	3.48E+06	3.24E+06	2.83E+06	5.20E+06	3.24E+06	1.60	0.68	5.55916E-05
P61077	Ubiquitin-conjugating enzyme E2 D3 OS=Homo sapiens OX=9606 GN=UBE2D3 PE=1 SV=1	20	13	2	6.07E+06	7.24E+06	3.40E+06	6.96E+06	3.47E+06	4.51E+06	3.16E+06	5.01E+06	1.23E+06	1.97E+06	3.62E+06	2.70E+06	5.27E+06	2.95E+06	1.79	0.84	0.02630549
Q04828	Aldo-keto reductase family 1 member C1 OS=Homo sapiens OX=9606 GN=AKR1C1 PE=1 SV=1	6	53	2	2.32E+07	2.60E+07	2.85E+07	3.19E+07	2.89E+07	2.66E+07	3.91E+07	4.12E+07	4.39E+07	4.26E+07	3.83E+07	3.38E+07	2.75E+07	3.98E+07	0.69	-0.53	8.91519E-05
P61970	Nuclear transport factor 2 OS=Homo sapiens OX=9606 GN=NUTF2 PE=1 SV=1	28	18	2	8.46E+06	7.74E+06	7.80E+06	1.19E+07	8.57E+06	8.73E+06	6.61E+06	8.30E+06	8.46E+06	8.39E+06	9.86E+06	7.82E+06	8.86E+06	8.24E+06	1.08	0.11	0.433027879
Q9UBI6	Guanine nucleotide-binding protein G(I)/G(S)/G(O) subunit gamma-12 OS=Homo sapiens OX=9606 GN=GNG12 PE=1 SV=3	32	24	2	4.59E+06	3.99E+06	3.58E+06	4.50E+06	3.76E+06	4.63E+06	2.32E+06	3.57E+06	3.32E+06	3.42E+06	2.77E+06	2.48E+06	4.18E+06	2.98E+06	1.40	0.49	0.001910277
P11245	Arylamine N-acetyltransferase 2 OS=Homo sapiens OX=9606 GN=NAT2 PE=1 SV=1	9	18	2	6.90E+06	3.30E+06	6.59E+06	7.16E+06	5.92E+06	5.19E+06	1.23E+07	6.10E+06	7.96E+06	9.15E+06	6.90E+06	6.13E+06	5.84E+06	8.09E+06	0.72	-0.47	0.081767135
Q9UN36	Protein NDRG2 OS=Homo sapiens OX=9606 GN=NDRG2 PE=1 SV=2	7	19	2	2.13E+06	2.60E+06	3.21E+06	3.78E+06	1.98E+06	3.26E+06	5.65E+06	5.04E+06	5.54E+06	5.37E+06	5.14E+06	2.86E+06	2.83E+06	4.93E+06	0.57	-0.80	0.002814837
P48960	CD97 antigen OS=Homo sapiens OX=9606 GN=CD97 PE=1 SV=4	4	15	2	3.11E+06	2.37E+06	1.90E+06	2.29E+06	2.48E+06	2.87E+06	1.30E+06	9.24E+05			1.12E+06		2.50E+06	1.11E+06	2.25	1.17	0.000277016
Q9Y383	Putative RNA-binding protein Luc7-like 2 OS=Homo sapiens OX=9606 GN=LUC7L2 PE=1 SV=2	6	6	2	7.96E+05	9.21E+05	1.14E+06	1.45E+06	1.35E+06	1.06E+06	1.00E+06	6.95E+05	7.99E+05	1.31E+06	7.97E+05	6.12E+05	1.12E+06	8.68E+05	1.29	0.37	0.11362792
P02788	Lactotransferrin OS=Homo sapiens OX=9606 GN=LTF PE=1 SV=6	3	122	2	2.54E+07	2.56E+07	2.74E+07	3.06E+07	2.61E+07	3.00E+07	6.43E+07	6.73E+07	7.68E+07	6.72E+07	7.56E+07	6.82E+07	2.75E+07	6.99E+07	0.39	-1.34	3.44529E-07
O75531	Barrier-to-autointegration factor OS=Homo sapiens OX=9606 GN=BANF1 PE=1 SV=1	40	6	2	2.14E+06	3.80E+06	1.96E+06	1.73E+06	2.08E+06	2.98E+06	2.13E+06	2.88E+06	1.99E+06	1.03E+06	3.16E+06	2.03E+06	2.45E+06	2.20E+06	1.11	0.15	0.592104179
Q7Z4W1	L-xylulose reductase OS=Homo sapiens OX=9606 GN=DCXR PE=1 SV=2	9	66	2	2.28E+07	2.69E+07	2.01E+07	2.15E+07	2.00E+07	2.04E+07	3.37E+07	3.36E+07	2.52E+07	2.79E+07	3.69E+07	2.67E+07	2.20E+07	3.07E+07	0.72	-0.48	0.004278787
P08603	Complement factor H OS=Homo sapiens OX=9606 GN=CFH PE=1 SV=4	2	4	2												2.09E+06					
P06727	Apolipoprotein A-IV OS=Homo sapiens OX=9606 GN=APOA4 PE=1 SV=3	6	44	2	4.21E+06	4.04E+06	4.54E+06	6.71E+06	5.07E+06	5.06E+06	6.30E+06	8.30E+06	8.69E+06	7.81E+06	6.53E+06	6.60E+06	4.94E+06	7.37E+06	0.67	-0.58	0.001739952
P19525	Interferon-induced, double-stranded RNA-activated protein kinase OS=Homo sapiens OX=9606 GN=EIF2AK2 PE=1 SV=2	4	4	2	2.21E+06	2.34E+06	2.64E+06	2.95E+06	2.56E+06	2.93E+06	6.32E+05	8.45E+05	5.70E+05	1.30E+06	4.45E+05	8.84E+05	2.60E+06	7.79E+05	3.35	1.74	1.01492E-06
Q00796	Sorbitol dehydrogenase OS=Homo sapiens OX=9606 GN=SORD PE=1 SV=4	6	38	2	8.68E+06	9.75E+06	1.12E+07	1.06E+07	9.75E+06	9.22E+06	1.33E+07	1.29E+07	1.38E+07	1.31E+07	1.05E+07	1.08E+07	9.87E+06	1.24E+07	0.80	-0.33	0.004862077
P08183	ATP-dependent translocase ABCB1 OS=Homo sapiens OX=9606 GN=ABCB1 PE=1 SV=3	3	10	2	1.00E+06	2.49E+06	7.25E+05	1.28E+06		2.06E+06	8.01E+05	6.22E+05	1.23E+06	2.11E+06		5.00E+05	1.51E+06	1.05E+06	1.43	0.52	0.331419497
Q9BPW8	Protein NipSnap homolog 1 OS=Homo sapiens OX=9606 GN=NIPSNAP1 PE=1 SV=1	13	9	2	3.97E+06	3.39E+06	1.64E+06	2.18E+06	9.27E+05	2.77E+06		2.24E+06	1.01E+06	1.38E+06	8.77E+05	2.14E+06	2.48E+06	1.53E+06	1.62	0.69	0.117038791
P09455	Retinol-binding protein 1 OS=Homo sapiens OX=9606 GN=RBP1 PE=1 SV=2	14	19	2	4.84E+06	5.09E+06	4.06E+06	5.06E+06	3.31E+06	4.20E+06	4.87E+06	7.70E+06	2.18E+06	3.56E+06	3.04E+06	3.93E+06	4.43E+06	4.21E+06	1.05	0.07	0.80855918
P48723	Heat shock 70 kDa protein 13 OS=Homo sapiens OX=9606 GN=HSPA13 PE=1 SV=1	5	18	2	2.93E+06	2.64E+06	2.47E+06	2.83E+06	2.49E+06	2.11E+06	3.52E+06	2.93E+06	3.38E+06	3.44E+06	2.98E+06	2.50E+06	2.58E+06	3.13E+06	0.82	-0.28	0.022127497
P08236	Beta-glucuronidase OS=Homo sapiens OX=9606 GN=GUSB PE=1 SV=2	4	14	2	3.52E+06	3.21E+06	4.11E+06	3.52E+06	4.38E+06	1.77E+06	8.65E+05	8.62E+05		4.56E+05	1.19E+06	9.47E+05	3.42E+06	8.64E+05	3.96	1.98	0.00063788
P28838	Cytosol aminopeptidase OS=Homo sapiens OX=9606 GN=LAP3 PE=1 SV=3	5	14	2	2.97E+06	2.06E+06	2.65E+06	3.45E+06	2.63E+06	2.18E+06	1.56E+06	4.87E+06	1.57E+06	3.13E+06	3.12E+06	2.11E+06	2.66E+06	2.73E+06	0.97	-0.04	0.904474887
Q06323	Proteasome activator complex subunit 1 OS=Homo sapiens OX=9606 GN=PSME1 PE=1 SV=1	8	11	2	3.15E+06	3.02E+06	3.58E+06	2.95E+06	3.45E+06	3.14E+06	8.69E+05	9.79E+05	9.76E+05	2.07E+06	1.25E+06		3.22E+06	1.23E+06	2.62	1.39	0.00023719
Q8N1G4	Leucine-rich repeat-containing protein 47 OS=Homo sapiens OX=9606 GN=LRRC47 PE=1 SV=1	4	4	2	2.66E+06	2.54E+06	3.20E+06	3.10E+06	3.19E+06	1.18E+06	4.57E+05	1.60E+06	1.66E+06	1.38E+06		1.03E+06	2.64E+06	1.23E+06	2.16	1.11	0.005533421
P30825	High affinity cationic amino acid transporter 1 OS=Homo sapiens OX=9606 GN=SLC7A1 PE=1 SV=1	4	6	2	8.61E+05	1.56E+06	1.75E+06	2.34E+06	2.62E+06	1.12E+06	8.21E+05	7.94E+05	1.17E+06	8.02E+05	6.91E+05	5.73E+05	1.71E+06	8.09E+05	2.11	1.08	0.021586988
P26583	High mobility group protein B2 OS=Homo sapiens OX=9606 GN=HMGB2 PE=1 SV=2	14	24	2	3.40E+06	3.29E+06	3.53E+06	4.45E+06	3.25E+06	2.88E+06	3.81E+06	3.89E+06	3.65E+06	3.77E+06	4.29E+06	3.04E+06	3.47E+06	3.74E+06	0.93	-0.11	0.332486326
P23284	Peptidyl-prolyl cis-trans isomerase B OS=Homo sapiens OX=9606 GN=PPIB PE=1 SV=2	12	31	2	6.58E+06	4.97E+06	5.85E+06	3.87E+06	5.19E+06	4.59E+06	4.40E+06	1.96E+06	4.36E+06	3.27E+06	2.63E+06	3.33E+06	5.17E+06	3.32E+06	1.56	0.64	0.00721025
Q9H2H9	Sodium-coupled neutral amino acid transporter 1 OS=Homo sapiens OX=9606 GN=SLC38A1 PE=1 SV=1	4	24	2	4.85E+06	6.02E+06	6.52E+06	6.73E+06	8.38E+06	5.55E+06	3.48E+06	2.18E+06	1.38E+06	3.40E+06	3.13E+06	2.93E+06	6.34E+06	2.75E+06	2.31	1.21	0.000214129
Q9Y3Z3	Deoxynucleoside triphosphate triphosphohydrolase SAMHD1 OS=Homo sapiens OX=9606 GN=SAMHD1 PE=1 SV=2	4	24	2	2.51E+06	3.02E+06	4.35E+06	4.30E+06	2.52E+06	2.18E+06	3.35E+06	3.10E+06	1.83E+06	2.21E+06	1.94E+06	2.49E+06	3.15E+06	2.49E+06	1.26	0.34	0.191912525
P45877	Peptidyl-prolyl cis-trans isomerase C OS=Homo sapiens OX=9606 GN=PPIC PE=1 SV=1	10	32	2	4.38E+06	6.43E+06	3.27E+06	4.40E+06	3.70E+06	3.77E+06	7.85E+06	1.04E+07	5.31E+06	5.18E+06	5.22E+06	4.37E+06	4.33E+06	6.39E+06	0.68	-0.56	0.086702087
P55769	NHP2-like protein 1 OS=Homo sapiens OX=9606 GN=SNU13 PE=1 SV=3	16	29	2	3.02E+06	3.52E+06	4.35E+06	5.34E+06	4.13E+06	3.61E+06	3.98E+06	3.92E+06	3.42E+06	3.24E+06	2.14E+06	3.22E+06	4.00E+06	3.32E+06	1.20	0.27	0.144910012
P61201	COP9 signalosome complex subunit 2 OS=Homo sapiens OX=9606 GN=COPS2 PE=1 SV=1	5	12	2	5.65E+05	1.21E+06	2.01E+06	1.18E+06	1.49E+06	1.33E+06	9.50E+05	1.24E+06	1.50E+06	8.85E+05	9.75E+05	8.74E+05	1.30E+06	1.07E+06	1.21	0.28	0.325838248
P48509	CD151 antigen OS=Homo sapiens OX=9606 GN=CD151 PE=1 SV=3	8	30	2	1.37E+07	1.24E+07	1.22E+07	1.28E+07	1.33E+07	1.98E+07	8.62E+06	7.80E+06	9.69E+06	9.74E+06	9.31E+06	6.86E+06	1.40E+07	8.67E+06	1.62	0.69	0.004306027
Q14160	Protein scribble homolog OS=Homo sapiens OX=9606 GN=SCRIB PE=1 SV=4	2	12	2	2.59E+08	2.91E+08	2.61E+08	1.92E+08	1.82E+08	2.63E+08	1.95E+08	2.75E+08	2.03E+08	2.11E+08	2.50E+08	2.38E+08	2.41E+08	2.29E+08	1.06	0.08	0.577298634
P00742	Coagulation factor X OS=Homo sapiens OX=9606 GN=F10 PE=1 SV=2	6	42	2	7.75E+06	8.56E+06	8.00E+06	5.52E+06	6.97E+06	8.48E+06	1.15E+07	9.68E+06	1.10E+07	7.97E+06	1.16E+07	5.60E+06	7.55E+06	9.56E+06	0.79	-0.34	0.102753823
Q08AM6	Protein VAC14 homolog OS=Homo sapiens OX=9606 GN=VAC14 PE=1 SV=1	4	14	2	2.20E+06	2.13E+06	2.42E+06	2.53E+06	2.05E+06	2.37E+06			1.16E+06	1.08E+06	9.34E+05	6.96E+05	2.29E+06	9.67E+05	2.36	1.24	4.15152E-05
Q04206	Transcription factor p65 OS=Homo sapiens OX=9606 GN=RELA PE=1 SV=2	4	9	2	3.38E+06	4.00E+06	3.77E+06	2.78E+06	3.24E+06	4.47E+06	9.47E+05	2.56E+06	1.02E+06	2.18E+06	1.91E+06	9.59E+05	3.61E+06	1.60E+06	2.26	1.18	0.000386348
P25685	DnaJ homolog subfamily B member 1 OS=Homo sapiens OX=9606 GN=DNAJB1 PE=1 SV=4	6	30	2	4.40E+06	4.34E+06	4.34E+06	5.44E+06	5.07E+06	5.05E+06	2.79E+06	3.03E+06	2.86E+06	2.77E+06	2.96E+06	3.13E+06	4.77E+06	2.92E+06	1.63	0.71	0.000107835
P08572	Collagen alpha-2(IV) chain OS=Homo sapiens OX=9606 GN=COL4A2 PE=1 SV=4	1	38	2	2.91E+06	2.64E+06	2.21E+06	2.95E+06	2.23E+06	2.42E+06	6.06E+06	7.01E+06	4.80E+06	4.52E+06	4.88E+06	4.77E+06	2.56E+06	5.34E+06	0.48	-1.06	0.000562783
P30048	Thioredoxin-dependent peroxide reductase, mitochondrial OS=Homo sapiens OX=9606 GN=PRDX3 PE=1 SV=3	10	6	2	4.51E+06	3.53E+06	2.06E+06	1.92E+06	2.31E+06	2.43E+06	2.48E+06	2.01E+06	1.32E+06	1.13E+06	1.98E+06	1.48E+06	2.79E+06	1.73E+06	1.61	0.69	0.055012773
P00450	Ceruloplasmin OS=Homo sapiens OX=9606 GN=CP PE=1 SV=1	3	26	2	2.55E+06	2.72E+06	2.83E+06	2.59E+06	2.11E+06	3.20E+06	3.42E+06	2.66E+06	2.94E+06	3.65E+06	2.76E+06	3.34E+06	2.67E+06	3.13E+06	0.85	-0.23	0.062951218
P35590	Tyrosine-protein kinase receptor Tie-1 OS=Homo sapiens OX=9606 GN=TIE1 PE=1 SV=1	3	15	2	2.73E+06	2.67E+06	4.49E+06	3.13E+06	2.94E+06	2.25E+06	3.41E+06	3.48E+06	5.03E+06	3.90E+06	3.37E+06	2.19E+06	3.04E+06	3.56E+06	0.85	-0.23	0.308773458
Q14558	Phosphoribosyl pyrophosphate synthase-associated protein 1 OS=Homo sapiens OX=9606 GN=PRPSAP1 PE=1 SV=2	7	11	2	5.47E+06	1.15E+07	6.53E+06	6.14E+06	6.62E+06	1.09E+07	4.95E+06	1.50E+07	5.59E+06	6.41E+06	7.79E+06	6.07E+06	7.85E+06	7.64E+06	1.03	0.04	0.913759857
P29279	CCN family member 2 OS=Homo sapiens OX=9606 GN=CCN2 PE=1 SV=2	5	36	2	1.43E+07	1.22E+07	1.60E+07	1.24E+07	1.24E+07	1.34E+07	2.15E+07	2.36E+07	2.65E+07	2.07E+07	2.24E+07	1.88E+07	1.35E+07	2.22E+07	0.61	-0.72	0.000110528
Q3LXA3	Triokinase/FMN cyclase OS=Homo sapiens OX=9606 GN=TKFC PE=1 SV=2	4	12	2	1.43E+06	2.41E+06	1.84E+06	1.95E+06	2.15E+06	1.70E+06	3.25E+06	2.77E+06	3.30E+06	2.67E+06	2.55E+06	2.64E+06	1.91E+06	2.86E+06	0.67	-0.58	0.000629046
Q13618	Cullin-3 OS=Homo sapiens OX=9606 GN=CUL3 PE=1 SV=2	5	4	2	1.97E+06	7.76E+05	1.13E+06	7.69E+05	7.00E+05		9.65E+05					6.01E+05	1.07E+06	7.83E+05	1.37	0.45	0.389921565
P19623	Spermidine synthase OS=Homo sapiens OX=9606 GN=SRM PE=1 SV=1	6	26	2	3.81E+06	3.74E+06	3.44E+06	4.18E+06	3.12E+06	2.67E+06	5.37E+06	4.56E+06	2.69E+06	3.13E+06	3.49E+06	2.64E+06	3.49E+06	3.65E+06	0.96	-0.06	0.765110479
Q9NZA1	Chloride intracellular channel protein 5 OS=Homo sapiens OX=9606 GN=CLIC5 PE=1 SV=3	4	6	2		6.13E+05	4.42E+05	6.84E+05			6.63E+05	4.60E+05	3.96E+05	3.25E+05	5.56E+05		5.79E+05	4.80E+05	1.21	0.27	0.33925833
Q03167	Transforming growth factor beta receptor type 3 OS=Homo sapiens OX=9606 GN=TGFBR3 PE=1 SV=3	3	6	2	2.77E+06	2.34E+06	3.49E+06	2.65E+06	2.50E+06	2.41E+06	3.31E+06	1.61E+06	1.46E+06	2.25E+06	1.54E+06	2.60E+06	2.69E+06	2.13E+06	1.26	0.34	0.141342199
O00186	Syntaxin-binding protein 3 OS=Homo sapiens OX=9606 GN=STXBP3 PE=1 SV=2	5	13	2		4.14E+05	1.05E+06		6.73E+05	7.52E+05			2.00E+05	3.84E+05	4.86E+05		7.22E+05	3.57E+05	2.02	1.02	0.067471556
P08559	Pyruvate dehydrogenase E1 component subunit alpha, somatic form, mitochondrial OS=Homo sapiens OX=9606 GN=PDHA1 PE=1 SV=3	6	5	2	1.33E+08	1.56E+08	1.24E+08	1.83E+08	1.08E+08	1.35E+08	2.23E+08	2.36E+08	1.82E+08	2.11E+08	1.97E+08	1.46E+08	1.40E+08	1.99E+08	0.70	-0.51	0.006281812
Q7Z2Z2	Elongation factor-like GTPase 1 OS=Homo sapiens OX=9606 GN=EFL1 PE=1 SV=2	3	5	2	2.00E+06	1.63E+06	1.28E+06	1.33E+06	1.11E+06	1.78E+06	1.33E+06	1.07E+06	7.69E+05	1.66E+06	1.62E+06	1.16E+06	1.52E+06	1.27E+06	1.20	0.26	0.225343263
P05455	Lupus La protein OS=Homo sapiens OX=9606 GN=SSB PE=1 SV=2	4	8	2	2.11E+06	1.97E+06	3.04E+06	2.78E+06	2.22E+06	2.21E+06	1.16E+06	1.01E+06	1.64E+06	1.34E+06	1.18E+06	8.16E+05	2.39E+06	1.19E+06	2.01	1.00	0.000303836
Q63HN8	E3 ubiquitin-protein ligase RNF213 OS=Homo sapiens OX=9606 GN=RNF213 PE=1 SV=3	0	8	2																	
P04066	Tissue alpha-L-fucosidase OS=Homo sapiens OX=9606 GN=FUCA1 PE=1 SV=4	5	14	2	2.52E+06	2.26E+06	2.08E+06	2.21E+06	1.94E+06	2.08E+06	3.80E+06	3.71E+06	3.25E+06	1.40E+06	2.43E+06	1.84E+06	2.18E+06	2.74E+06	0.80	-0.33	0.236693727
Q5JTH9	RRP12-like protein OS=Homo sapiens OX=9606 GN=RRP12 PE=1 SV=2	4	4	2	5.22E+05		5.30E+05	4.15E+05		4.89E+05							4.89E+05				
P13497	Bone morphogenetic protein 1 OS=Homo sapiens OX=9606 GN=BMP1 PE=1 SV=2	3	8	2	1.48E+07	1.68E+07	1.05E+07	1.17E+07	1.08E+07	1.08E+07	1.26E+07	1.29E+07	9.53E+06	8.84E+06	8.31E+06	9.10E+06	1.26E+07	1.02E+07	1.23	0.30	0.114051355
P10646	Tissue factor pathway inhibitor OS=Homo sapiens OX=9606 GN=TFPI PE=1 SV=1	6	28	2	4.13E+06	4.84E+06	3.00E+06	3.07E+06	3.10E+06	3.15E+06	5.09E+06	5.70E+06	4.10E+06	3.73E+06	3.33E+06	3.59E+06	3.55E+06	4.26E+06	0.83	-0.26	0.182847344
Q16563	Synaptophysin-like protein 1 OS=Homo sapiens OX=9606 GN=SYPL1 PE=1 SV=1	15	16	2	2.02E+06		1.67E+06	3.59E+06	4.07E+06	2.67E+06	1.53E+06	1.72E+06	1.02E+06	8.16E+05	1.75E+06	1.77E+06	2.80E+06	1.44E+06	1.95	0.97	0.036330681
Q93009	Ubiquitin carboxyl-terminal hydrolase 7 OS=Homo sapiens OX=9606 GN=USP7 PE=1 SV=2	3	4	2	2.36E+06	1.64E+06	1.18E+06	7.41E+05	1.43E+06	1.42E+06	1.23E+06	1.34E+06	1.29E+06	5.47E+05	5.87E+05	9.14E+05	1.46E+06	9.84E+05	1.49	0.57	0.102716953
O60610	Protein diaphanous homolog 1 OS=Homo sapiens OX=9606 GN=DIAPH1 PE=1 SV=2	2	12	2	9.77E+05	7.62E+05	5.38E+05	7.86E+05	5.68E+05	6.13E+05	7.43E+05	4.54E+05	3.75E+05	5.27E+05		6.12E+05	7.07E+05	5.42E+05	1.30	0.38	0.110086066
P13473	Lysosome-associated membrane glycoprotein 2 OS=Homo sapiens OX=9606 GN=LAMP2 PE=1 SV=2	7	8	2	2.10E+06		1.52E+06	1.66E+06	1.85E+06	1.31E+06			4.56E+05	4.41E+05	1.63E+06	1.37E+06	1.69E+06	9.74E+05	1.73	0.79	0.098951116

Supplementary Table 4. List of all proteins identified by label free proteomics analysis.

Accession	Description	Coverage [%]	# PSMs	# Unique Peptides	Amitriptyline 1	Amitriptyline 2	Amitriptyline 3	Amitriptyline 4	Amitriptyline 5	Amitriptyline 6	Vehicle 1	Vehicle 2	Vehicle 3	Vehicle 4	Vehicle 5	Vehicle 6	Average amitriptyline	Average vehicle	Ratio amitriptyline/ vehicle	Log 2 fold change (amitriptyline /vehicle)	P-value
Q86UP2	Kinectin OS=Homo sapiens OX=9606 GN=KTN1 PE=1 SV=1	2	24	2	2.46E+06	2.63E+06	1.77E+06	1.98E+06	2.40E+06	2.08E+06	3.20E+06	3.46E+06	4.07E+06	2.85E+06	3.49E+06	2.99E+06	2.22E+06	3.34E+06	0.66	-0.59	0.000622413
P61011	Signal recognition particle 54 kDa protein OS=Homo sapiens OX=9606 GN=SRP54 PE=1 SV=1	4	14	2	5.49E+06	5.85E+06	4.31E+06	4.41E+06	4.56E+06	3.88E+06	2.99E+06	3.16E+06	2.52E+06	1.93E+06	1.35E+06	8.93E+05	4.75E+06	2.14E+06	2.22	1.15	0.000338126
P27144	Adenylate kinase 4, mitochondrial OS=Homo sapiens OX=9606 GN=AK4 PE=1 SV=1	13	10	2		1.90E+06	1.78E+06	1.90E+06	1.74E+06	1.57E+06	8.20E+05	1.08E+06	8.41E+05	9.82E+05	1.13E+06	6.88E+05	1.78E+06	9.23E+05	1.93	0.95	7.00256E-06
P07947	Tyrosine-protein kinase Yes OS=Homo sapiens OX=9606 GN=YES1 PE=1 SV=3	4	61	2	2.61E+06	2.97E+06	1.83E+06	3.08E+06	1.80E+06	3.72E+06	2.46E+06	1.78E+06	1.78E+06	2.20E+06	2.29E+06	3.06E+06	2.67E+06	2.26E+06	1.18	0.24	0.293676934
P26358	DNA (cytosine-5)-methyltransferase 1 OS=Homo sapiens OX=9606 GN=DNMT1 PE=1 SV=2	2	5	2	9.56E+06	1.31E+07	9.21E+06	1.39E+07	1.16E+07	8.94E+06	1.14E+07	1.67E+07	1.03E+07	1.82E+07	9.85E+06	8.86E+06	1.10E+07	1.26E+07	0.88	-0.18	0.4317191
Q53EL6	Programmed cell death protein 4 OS=Homo sapiens OX=9606 GN=PDCD4 PE=1 SV=2	7	3	2	7.20E+05	7.47E+05	1.05E+06	7.92E+05	6.87E+05	6.48E+05	6.10E+05	5.09E+05	4.83E+05	5.63E+05	4.54E+05	5.47E+05	7.74E+05	5.28E+05	1.47	0.55	0.006947303
P62750	60S ribosomal protein L23a OS=Homo sapiens OX=9606 GN=RPL23A PE=1 SV=1	16	13	2	2.01E+06	3.20E+06	4.58E+06	3.61E+06	4.56E+06	3.52E+06	3.35E+06	2.58E+06	3.53E+06	2.07E+06	2.50E+06	2.39E+06	3.58E+06	2.74E+06	1.31	0.39	0.100604684
P51610	Host cell factor 1 OS=Homo sapiens OX=9606 GN=HCFC1 PE=1 SV=2	1	6	2	5.52E+05	6.93E+05	7.11E+05	8.62E+05	1.00E+06	6.99E+05	3.41E+05	4.28E+05	4.96E+05		3.59E+05		7.53E+05	4.06E+05	1.85	0.89	0.001781073
Q14247	Src substrate cortactin OS=Homo sapiens OX=9606 GN=CTTN PE=1 SV=2	4	6	2	4.76E+05	2.40E+06	3.21E+06	3.12E+06	3.31E+06	2.73E+06		1.74E+06		4.98E+05	5.49E+05	1.00E+06	2.54E+06	9.48E+05	2.68	1.42	0.016237802
P22352	Glutathione peroxidase 3 OS=Homo sapiens OX=9606 GN=GPX3 PE=1 SV=2	8	393	2	1.95E+07	4.04E+07	4.12E+07	2.03E+07	2.56E+07	3.63E+07	3.69E+07	3.53E+07	6.21E+07	3.54E+07	4.91E+07	4.96E+07	3.06E+07	4.47E+07	0.68	-0.55	0.039728575
Q9UKK3	Protein mono-ADP-ribosyltransferase PARP4 OS=Homo sapiens OX=9606 GN=PARP4 PE=1 SV=3	2	8	2	2.21E+06	2.90E+06	3.27E+06	1.90E+06	9.43E+05	2.16E+06	1.46E+06	1.95E+06		4.65E+05	1.21E+06	6.30E+05	2.23E+06	1.14E+06	1.96	0.97	0.031716787
Q9HA64	Ketosamine-3-kinase OS=Homo sapiens OX=9606 GN=FN3KRP PE=1 SV=2	10	8	2	3.07E+06	2.92E+06	2.94E+06	2.53E+06	2.44E+06	2.32E+06	2.26E+06	2.04E+06	2.70E+06	2.27E+06	3.12E+06	2.64E+06	2.70E+06	2.50E+06	1.08	0.11	0.357277828
Q9UDY4	DnaJ homolog subfamily B member 4 OS=Homo sapiens OX=9606 GN=DNAJB4 PE=1 SV=1	6	16	2	2.79E+06	4.11E+06	2.55E+06	3.76E+06	2.43E+06	3.97E+06	3.23E+06	2.79E+06	2.62E+06	2.55E+06	2.18E+06	2.82E+06	3.27E+06	2.70E+06	1.21	0.28	0.136321765
P63173	60S ribosomal protein L38 OS=Homo sapiens OX=9606 GN=RPL38 PE=1 SV=2	33	24	2	6.91E+06	8.91E+06	1.10E+07	1.27E+07	1.11E+07	1.09E+07	6.86E+06	6.07E+06	9.12E+06	7.97E+06	8.14E+06	4.21E+06	1.03E+07	7.06E+06	1.45	0.54	0.015947836
Q96KG9	N-terminal kinase-like protein OS=Homo sapiens OX=9606 GN=SCYL1 PE=1 SV=1	4	4	2	9.96E+05	1.25E+06	1.15E+06	1.06E+06	9.06E+05	9.96E+05	7.44E+05	7.87E+05	8.02E+05	8.63E+05	8.29E+05	5.91E+05	1.06E+06	7.69E+05	1.38	0.46	0.001253744
Q8NBI5	Solute carrier family 43 member 3 OS=Homo sapiens OX=9606 GN=SLC43A3 PE=1 SV=2	5	18	2	5.74E+06	5.90E+06	5.41E+06	5.13E+06	5.29E+06	5.06E+06	4.20E+06	3.98E+06	3.67E+06	4.13E+06	3.34E+06	3.41E+06	5.42E+06	3.79E+06	1.43	0.52	1.18136E-05
P39059	Collagen alpha-1(XV) chain OS=Homo sapiens OX=9606 GN=COL15A1 PE=1 SV=2	1	34	2	1.13E+07	1.23E+07	8.66E+06	9.25E+06	9.67E+06	9.40E+06	1.42E+07	1.56E+07	1.13E+07	1.32E+07	1.14E+07	1.19E+07	1.01E+07	1.29E+07	0.78	-0.36	0.010780158
P04180	Phosphatidylcholine-sterol acyltransferase OS=Homo sapiens OX=9606 GN=LCAT PE=1 SV=1	4	71	2	1.35E+07	1.52E+07	2.86E+07	1.52E+07	1.37E+07	1.39E+07	2.26E+07	2.35E+07	2.54E+07	2.44E+07	1.63E+07	1.86E+07	1.67E+07	2.18E+07	0.77	-0.38	0.105951358
Q15046	Lysine--tRNA ligase OS=Homo sapiens OX=9606 GN=KARS1 PE=1 SV=3	5	8	2	2.99E+06	4.24E+06	4.48E+06	5.18E+06	4.11E+06	3.27E+06	3.57E+06	3.99E+06	4.41E+06	4.29E+06	2.60E+06	9.07E+05	4.04E+06	3.29E+06	1.23	0.30	0.2725313
Q8N8N7	Prostaglandin reductase 2 OS=Homo sapiens OX=9606 GN=PTGR2 PE=1 SV=1	6	5	2		5.54E+05	4.93E+05	4.03E+05	3.07E+05	4.17E+05	1.29E+06				5.07E+05	7.07E+05	4.35E+05	8.36E+05	0.52	-0.94	0.228612787
Q9BRL6	Serine/arginine-rich splicing factor 8 OS=Homo sapiens OX=9606 GN=SRSF8 PE=1 SV=1	5	30	2	9.24E+06	1.16E+07	1.06E+07	1.15E+07	1.13E+07	9.80E+06	1.13E+07	1.27E+07	1.15E+07	1.28E+07	1.07E+07	1.10E+07	1.07E+07	1.17E+07	0.91	-0.13	0.091034611
Q8N1F7	Nuclear pore complex protein Nup93 OS=Homo sapiens OX=9606 GN=NUP93 PE=1 SV=2	2	24	2	2.36E+06	3.34E+06	1.81E+06	3.20E+06	3.51E+06	2.74E+06	1.09E+06	2.32E+06	2.73E+06	3.10E+06	2.76E+06	6.49E+05	2.83E+06	2.11E+06	1.34	0.42	0.176452279
Q13428	Treacle protein OS=Homo sapiens OX=9606 GN=TCOF1 PE=1 SV=3	1	12	2	4.03E+06	3.93E+06	3.10E+06	4.14E+06	3.65E+06	3.43E+06	1.06E+06	1.18E+06	7.72E+05	1.52E+06	1.61E+06	1.81E+06	3.71E+06	1.32E+06	2.80	1.49	1.00794E-06
P59998	Actin-related protein 2/3 complex subunit 4 OS=Homo sapiens OX=9606 GN=ARPC4 PE=1 SV=3	16	40	2	9.60E+06	9.65E+06	6.17E+06	7.92E+06	6.43E+06	6.02E+06	9.66E+06	8.99E+06	6.65E+06	6.65E+06	6.90E+06	5.87E+06	7.63E+06	7.45E+06	1.02	0.03	0.849522163
Q9UKG1	DCC-interacting protein 13-alpha OS=Homo sapiens OX=9606 GN=APPL1 PE=1 SV=1	4	11	2	1.28E+06	1.73E+06	1.73E+06	1.90E+06	1.90E+06	1.61E+06	1.45E+06		1.68E+06		2.16E+06		1.69E+06	1.76E+06	0.96	-0.06	0.775288131
Q92572	AP-3 complex subunit sigma-1 OS=Homo sapiens OX=9606 GN=AP3S1 PE=1 SV=1	12	17	2	3.86E+06	4.72E+06	2.72E+06	3.73E+06	3.31E+06	2.87E+06	1.23E+06	1.14E+06	1.98E+06	2.44E+06	1.21E+06	1.64E+06	3.53E+06	1.61E+06	2.20	1.14	0.000531902
P19367	Hexokinase-1 OS=Homo sapiens OX=9606 GN=HK1 PE=1 SV=3	3	4	2	3.13E+06	2.81E+06	2.68E+06	2.41E+06	2.91E+06	3.17E+06	1.54E+06	5.78E+05	6.01E+05	4.57E+05	7.80E+05	1.55E+06	2.85E+06	9.18E+05	3.11	1.64	3.41232E-05
Q08722	Leukocyte surface antigen CD47 OS=Homo sapiens OX=9606 GN=CD47 PE=1 SV=1	5	48	2	9.50E+06	1.04E+07	1.48E+07	1.07E+07	1.34E+07	9.27E+06	8.13E+06	8.44E+06	8.77E+06	7.23E+06	7.90E+06	5.30E+06	1.13E+07	7.63E+06	1.49	0.57	0.007746523
O76074	cGMP-specific 3',5'-cyclic phosphodiesterase OS=Homo sapiens OX=9606 GN=PDE5A PE=1 SV=2	3	9	2	1.03E+06		3.54E+05		4.80E+05	3.33E+05	2.10E+06		1.71E+06		1.27E+06	1.12E+06	5.49E+05	1.55E+06	0.35	-1.50	0.012534034
Q9UKM9	RNA-binding protein Raly OS=Homo sapiens OX=9606 GN=RALY PE=1 SV=1	6	15	2	7.09E+06	7.58E+06	8.06E+06	8.79E+06	8.26E+06	7.94E+06	4.93E+06	5.13E+06	2.10E+06	1.68E+06	4.87E+06	4.17E+06	7.95E+06	3.81E+06	2.08	1.06	0.000636003
Q96JB2	Conserved oligomeric Golgi complex subunit 3 OS=Homo sapiens OX=9606 GN=COG3 PE=1 SV=3	4	4	2																	
Q13451	Peptidyl-prolyl cis-trans isomerase FKBP5 OS=Homo sapiens OX=9606 GN=FKBP5 PE=1 SV=2	4	6	2	1.49E+06	1.10E+06	1.05E+06	1.35E+06	1.26E+06		5.90E+05	5.41E+05	6.33E+05				1.25E+06	5.88E+05	2.12	1.09	0.000650481
Q12846	Syntaxin-4 OS=Homo sapiens OX=9606 GN=STX4 PE=1 SV=2	7	16	2	2.94E+06	3.41E+06	3.81E+06	3.40E+06	3.31E+06	4.01E+06	2.67E+06	2.81E+06	2.33E+06	2.51E+06	1.94E+06	7.44E+06	3.48E+06	3.28E+06	1.06	0.08	0.82510637
Q9Y2T2	AP-3 complex subunit mu-1 OS=Homo sapiens OX=9606 GN=AP3M1 PE=1 SV=1	4	14	2	1.09E+06	9.78E+05	2.17E+06	1.73E+06	1.85E+06	2.01E+06	7.76E+05	1.34E+06	1.43E+06	6.68E+05	1.07E+06	9.94E+05	1.64E+06	1.05E+06	1.56	0.65	0.035494254
Q06278	Aldehyde oxidase OS=Homo sapiens OX=9606 GN=AOX1 PE=1 SV=2	2	16	2	9.74E+05	1.28E+06	1.51E+06	1.18E+06			1.63E+06	2.22E+06	2.07E+06	2.19E+06	2.52E+06	1.84E+06	1.24E+06	2.08E+06	0.59	-0.75	0.001151674
Q9UJU6	Drebrin-like protein OS=Homo sapiens OX=9606 GN=DBNL PE=1 SV=1	6	15	2																	
P10619	Lysosomal protective protein OS=Homo sapiens OX=9606 GN=CTSA PE=1 SV=2	5	6	2	3.91E+06	5.76E+06	4.01E+06	3.69E+06	2.68E+06	1.03E+06	2.19E+06	2.38E+06	2.46E+06	2.56E+06	7.96E+05	7.39E+05	3.51E+06	1.85E+06	1.89	0.92	0.053986254
Q7L1Q6	Basic leucine zipper and W2 domain-containing protein 1 OS=Homo sapiens OX=9606 GN=BZW1 PE=1 SV=1	5	21	2	6.16E+06	2.55E+06	6.23E+06	1.76E+06	6.88E+06	1.66E+06	5.53E+06	1.66E+06	5.89E+06	5.84E+06	5.14E+06	4.38E+06	4.21E+06	4.74E+06	0.89	-0.17	0.666941036
O15400	Syntaxin-7 OS=Homo sapiens OX=9606 GN=STX7 PE=1 SV=4	8	5	2	2.07E+06	2.04E+06	1.94E+06	1.86E+06	1.79E+06	5.45E+05	7.99E+05	8.03E+05	9.77E+05	8.06E+05	4.67E+05		1.71E+06	7.71E+05	2.21	1.15	0.009068842
Q7L9L4	MOB kinase activator 1B OS=Homo sapiens OX=9606 GN=MOB1B PE=1 SV=3	9	28	2	1.60E+06	2.26E+06	1.99E+06	2.92E+06	2.35E+06	2.73E+06	2.53E+06	2.65E+06	2.71E+06	2.90E+06	3.58E+06	2.06E+06	2.31E+06	2.74E+06	0.84	-0.25	0.158502927
Q9UNF0	Protein kinase C and casein kinase substrate in neurons protein 2 OS=Homo sapiens OX=9606 GN=PACSIN2 PE=1 SV=2	4	7	2	1.82E+06	1.92E+06	2.44E+06	2.62E+06	2.35E+06	1.78E+06	1.07E+06	1.04E+06	1.06E+06	9.77E+05	1.45E+06	1.03E+06	2.16E+06	1.10E+06	1.95	0.96	0.000317653
P53634	Dipeptidyl peptidase 1 OS=Homo sapiens OX=9606 GN=CTSC PE=1 SV=2	5	6	2	1.93E+06	2.17E+06	8.51E+05	2.28E+06	2.32E+06	2.47E+06	1.92E+06	1.99E+06	2.85E+05	1.35E+06	1.72E+06	1.25E+06	2.00E+06	1.42E+06	1.41	0.50	0.130759669
P15529	Membrane cofactor protein OS=Homo sapiens OX=9606 GN=CD46 PE=1 SV=3	4	14	2	2.92E+06	2.41E+06	2.21E+06	4.32E+06	2.92E+06	4.21E+06	1.43E+06	1.30E+06	1.15E+06	1.65E+06	1.34E+06	9.66E+05	3.17E+06	1.31E+06	2.42	1.28	0.003167779
Q99986	Serine/threonine-protein kinase VRK1 OS=Homo sapiens OX=9606 GN=VRK1 PE=1 SV=1	6	8	2		4.57E+05	8.95E+05	7.30E+05	6.34E+05	6.32E+05	5.35E+05	4.17E+05	4.46E+05	3.39E+05	4.14E+05	3.16E+05	6.70E+05	4.11E+05	1.63	0.70	0.01831036
O43837	Isocitrate dehydrogenase [NAD] subunit beta, mitochondrial OS=Homo sapiens OX=9606 GN=IDH3B PE=1 SV=2	5	18	2	2.31E+06	2.34E+06	1.66E+06	2.41E+06	2.21E+06	1.33E+06	1.25E+06	1.26E+06	1.05E+06	1.32E+06		6.05E+05	2.04E+06	1.10E+06	1.86	0.90	0.00237797
Q9H267	Vacuolar protein sorting-associated protein 33B OS=Homo sapiens OX=9606 GN=VPS33B PE=1 SV=2	5	3	2	2.68E+06	1.28E+06	1.52E+06	1.25E+06	8.49E+05	2.30E+06	2.32E+06	1.91E+06	9.45E+05	1.12E+06	1.37E+06	1.44E+06	1.65E+06	1.52E+06	1.09	0.12	0.720299849
P56199	Integrin alpha-1 OS=Homo sapiens OX=9606 GN=ITGA1 PE=1 SV=2	2	11	2	3.56E+06	3.33E+06	3.52E+06	4.00E+06	3.47E+06	2.57E+06	2.54E+06	2.42E+06	2.15E+06	1.10E+06	1.52E+06	1.97E+06	3.41E+06	1.95E+06	1.75	0.81	0.000633867
O60313	Dynamin-like 120 kDa protein, mitochondrial OS=Homo sapiens OX=9606 GN=OPA1 PE=1 SV=3	3	7	2	3.26E+06	2.05E+06	1.52E+06	3.14E+06	2.96E+06	2.13E+06		4.76E+06		2.54E+06	4.96E+06	1.23E+06	2.51E+06	3.37E+06	0.74	-0.43	0.418604386
Q14C86	GTPase-activating protein and VPS9 domain-containing protein 1 OS=Homo sapiens OX=9606 GN=GAPVD1 PE=1 SV=2	2	6	2	1.90E+06	1.89E+06	1.45E+06	1.98E+06	2.29E+06	2.15E+06				1.29E+06			1.94E+06	1.29E+06	1.51	0.60	
P13987	CD59 glycoprotein OS=Homo sapiens OX=9606 GN=CD59 PE=1 SV=1	16	12	2		5.12E+05		2.30E+07	2.05E+07	1.84E+07		5.11E+05	1.77E+07				1.56E+07	9.08E+06	1.72	0.78	0.58845335
P36915	Guanine nucleotide-binding protein-like 1 OS=Homo sapiens OX=9606 GN=GNL1 PE=1 SV=2	3	6	2	3.16E+06	3.45E+06	2.61E+06	2.94E+06	2.06E+06	2.55E+06	2.44E+06	2.67E+06	2.31E+06	1.78E+06	2.04E+06	1.57E+06	2.79E+06	2.14E+06	1.31	0.39	0.032533996
Q15102	Platelet-activating factor acetylhydrolase IB subunit gamma OS=Homo sapiens OX=9606 GN=PAFAH1B3 PE=1 SV=1	8	16	2	4.44E+06	4.22E+06	4.61E+06	4.05E+06	3.86E+06	4.90E+06	4.54E+06	4.23E+06	4.02E+06	3.70E+06	3.30E+06	4.24E+06	4.35E+06	4.00E+06	1.09	0.12	0.18410744
P09012	U1 small nuclear ribonucleoprotein A OS=Homo sapiens OX=9606 GN=SNRPA PE=1 SV=3	6	28	2	5.24E+06	5.09E+06	7.11E+06	7.16E+06	5.57E+06	4.25E+06	1.86E+06	1.64E+06	5.17E+06	4.29E+06	2.12E+06	3.12E+06	5.74E+06	3.03E+06	1.89	0.92	0.005371269
Q13620	Cullin-4B OS=Homo sapiens OX=9606 GN=CUL4B PE=1 SV=4	2	6	2	7.34E+05	7.60E+05	8.12E+05	9.73E+05	7.13E+05	7.44E+05	9.05E+05	6.15E+05	5.64E+05	4.52E+05	7.21E+05	3.67E+05	7.89E+05	6.04E+05	1.31	0.39	0.070792404
P22061	Protein-L-isoaspartate(D-aspartate) O-methyltransferase OS=Homo sapiens OX=9606 GN=PCMT1 PE=1 SV=4	7	28	2	4.27E+06	5.45E+06	5.38E+06	5.84E+06	5.45E+06	4.84E+06	5.86E+06	5.83E+06	5.64E+06	5.84E+06	5.08E+06	4.28E+06	5.21E+06	5.42E+06	0.96	-0.06	0.548697345
Q9UGM3	Deleted in malignant brain tumors 1 protein OS=Homo sapiens OX=9606 GN=DMBT1 PE=1 SV=2	9	3	2							2.04E+06	2.04E+06	1.40E+06	2.65E+06				2.03E+06			
P45973	Chromobox protein homolog 5 OS=Homo sapiens OX=9606 GN=CBX5 PE=1 SV=1	11	22	2	8.46E+06	7.86E+06	9.13E+06	7.26E+06	9.28E+06	8.75E+06	3.86E+06	2.68E+06	4.45E+06	2.70E+06	3.35E+06	3.17E+06	8.46E+06	3.37E+06	2.51	1.33	3.30351E-07
P42765	3-ketoacyl-CoA thiolase, mitochondrial OS=Homo sapiens OX=9606 GN=ACAA2 PE=1 SV=2	5	6	2	3.78E+06		3.59E+06	2.04E+06	3.66E+06	4.32E+06	1.08E+06	1.43E+06	1.03E+06	3.22E+06	3.34E+06	1.05E+06	3.48E+06	1.86E+06	1.87	0.90	0.023318943
P11172	Uridine 5'-monophosphate synthase OS=Homo sapiens OX=9606 GN=UMPS PE=1 SV=1	4	8	2	3.69E+06	3.95E+06	3.36E+06	3.94E+06	2.77E+06	4.25E+06	3.23E+06	3.40E+06	3.23E+06		3.16E+06	3.15E+06	3.66E+06	3.23E+06	1.13	0.18	0.104645707
Q04446	1,4-alpha-glucan-branching enzyme OS=Homo sapiens OX=9606 GN=GBE1 PE=1 SV=3	5	3	2	1.14E+06		1.08E+06	2.57E+05	1.06E+06	6.14E+05							8.32E+05				
O15305	Phosphomannomutase 2 OS=Homo sapiens OX=9606 GN=PMM2 PE=1 SV=1	7	21	2	9.22E+06	1.01E+07	1.03E+07	1.26E+07	9.85E+06	8.21E+06	1.27E+07	1.35E+07	1.28E+07	1.25E+07	1.34E+07	1.16E+07	1.01E+07	1.27E+07	0.79	-0.34	0.004652396
Q9BVJ7	Dual specificity protein phosphatase 23 OS=Homo sapiens OX=9606 GN=DUSP23 PE=1 SV=1	15	5	2	2.13E+06	2.06E+06	1.73E+06	1.56E+06	9.82E+05	1.92E+06		1.63E+06	5.14E+05	1.73E+06	7.60E+05	5.30E+05	1.73E+06	1.03E+06	1.67	0.74	0.065199107
Q5M9N0	Coiled-coil domain-containing protein 158 OS=Homo sapiens OX=9606 GN=CCDC158 PE=2 SV=2	3	35	2	1.09E+08	1.23E+08	1.98E+08	2.29E+08	1.87E+08	1.79E+08	2.47E+08	2.41E+08	2.82E+08	2.53E+08	2.39E+08	2.31E+08	1.71E+08	2.49E+08	0.69	-0.54	0.007174991
P43246	DNA mismatch repair protein Msh2 OS=Homo sapiens OX=9606 GN=MSH2 PE=1 SV=1	2	21	2	1.78E+07	2.09E+07	1.92E+07	2.14E+07	2.05E+07	1.85E+07	2.56E+07	2.57E+07	3.10E+07	2.97E+07	2.55E+07	2.13E+07	1.97E+07	2.65E+07	0.75	-0.42	0.003534954
Q9H845	Complex I assembly factor ACAD9, mitochondrial OS=Homo sapiens OX=9606 GN=ACAD9 PE=1 SV=1	3	12	2	3.51E+08	3.57E+08	3.41E+08	2.94E+08	2.82E+08	3.23E+08	6.42E+08	6.35E+08	4.64E+08	5.78E+08	5.60E+08	4.58E+08	3.25E+08	5.56E+08	0.58	-0.78	0.000434855
P60520	Gamma-aminobutyric acid receptor-associated protein-like 2 OS=Homo sapiens OX=9606 GN=GABARAPL2 PE=1 SV=1	12	17	2	4.19E+06	5.77E+06	4.83E+06	5.73E+06	6.14E+06	4.06E+06	9.02E+05	1.01E+06	2.73E+06	1.79E+06	2.81E+06	1.05E+06	5.12E+06	1.72E+06	2.98	1.58	5.40329E-05
Q14289	Protein-tyrosine kinase 2-beta OS=Homo sapiens OX=9606 GN=PTK2B PE=1 SV=2	2	20	2	8.39E+06	8.13E+06	1.06E+07	1.33E+07	1.23E+07	1.08E+07	1.66E+07	1.63E+07	2.13E+07	1.70E+07	1.75E+07	1.33E+07	1.06E+07	1.70E+07	0.62	-0.69	0.00083523
P09496	Clathrin light chain A OS=Homo sapiens OX=9606 GN=CLTA PE=1 SV=1	6	24	2	1.60E+07	1.83E+07	1.36E+07	1.18E+07	1.48E+07	1.46E+07	2.00E+07	1.98E+07	2.43E+07	1.70E+07	2.13E+07	1.52E+07	1.49E+07	1.96E+07	0.76	-0.40	0.015601422
P46976	Glycogenin-1 OS=Homo sapiens OX=9606 GN=GYG1 PE=1 SV=4	5	4	2	3.34E+06	3.24E+06	3.65E+06	4.75E+06	3.99E+06	1.91E+06	3.89E+06	4.69E+06	4.44E+06	5.55E+06	3.48E+06	3.38E+06	3.48E+06	4.24E+06	0.82	-0.28	0.169241516
O95347	Structural maintenance of chromosomes protein 2 OS=Homo sapiens OX=9606 GN=SMC2 PE=1 SV=2	2	5	2	1.17E+06	1.59E+06	1.54E+06	1.12E+06	1.24E+06	1.17E+06	5.02E+05	3.87E+05				2.70E+05	1.31E+06	3.86E+05	3.38	1.76	7.71827E-05
Q8WZ42	Titin OS=Homo sapiens OX=9606 GN=TTN PE=1 SV=4	0	11	2	2.97E+06	3.63E+06	2.74E+06	3.23E+06	2.04E+06	1.35E+06	5.28E+06	2.98E+06	4.08E+06	2.07E+06	5.35E+06	4.30E+06	2.66E+06	4.01E+06	0.66	-0.59	0.061343705
Q12882	Dihydropyrimidine dehydrogenase [NADP(+)] OS=Homo sapiens OX=9606 GN=DPYD PE=1 SV=2	2	6	2	3.33E+05	4.99E+05	4.48E+05	6.44E+05	5.78E+05	3.11E+05	2.70E+05	9.20E+05	4.27E+05	8.64E+05		5.78E+05	4.69E+05	6.12E+05	0.77	-0.38	0.336009406
P25398	40S ribosomal protein S12 OS=Homo sapiens OX=9606 GN=RPS12 PE=1 SV=3	14	6	2	3.22E+06	2.87E+06	3.77E+06	3.92E+06	3.47E+06	2.74E+06	3.46E+06	2.58E+06	3.42E+06	2.45E+06	2.90E+06	2.61E+06	3.33E+06	2.90E+06	1.15	0.20	0.135191235
Q96SN8	CDK5 regulatory subunit-associated protein 2 OS=Homo sapiens OX=9606 GN=CDK5RAP2 PE=1 SV=5	1	5	2	1.14E+08	1.23E+08	1.08E+08	1.14E+08	1.18E+08	1.08E+08	1.49E+08	1.47E+08	1.50E+08	1.55E+08	1.36E+08	1.20E+08	1.14E+08	1.43E+08	0.80	-0.32	0.001797956
P04350	Tubulin beta-4A chain OS=Homo sapiens OX=9606 GN=TUBB4A PE=1 SV=2	78	4364	3	7.59E+07	7.21E+07	4.38E+07	5.07E+07	5.37E+07	6.12E+07	4.05E+07	3.75E+07	3.17E+07	4.15E+07	4.38E+07	3.76E+07	5.96E+07	3.88E+07	1.54	0.62	0.008213349
Q13885	Tubulin beta-2A chain OS=Homo sapiens OX=9606 GN=TUBB2A PE=1 SV=1	62	4377	3	3.49E+06	4.02E+06	4.07E+06	4.28E+06	4.93E+06	4.06E+06	3.19E+06	5.09E+06	3.48E+06	4.60E+06	4.13E+06	4.17E+06	4.14E+06	4.11E+06	1.01	0.01	0.933456377
Q16778	Histone H2B type 2-E OS=Homo sapiens OX=9606 GN=HIST2H2BE PE=1 SV=3	56	6292	3	1.26E+09	1.32E+09	1.07E+09	1.26E+09	1.21E+09	1.24E+09	1.31E+09	1.40E+09	1.41E+09	1.67E+09	1.82E+09	1.11E+09	1.23E+09	1.45E+09	0.84	-0.25	0.082991654
Q8IUE6	Histone H2A type 2-B OS=Homo sapiens OX=9606 GN=HIST2H2AB PE=1 SV=3	63	414	3	2.24E+08	2.40E+08	2.14E+08	1.39E+08	1.82E+08	2.06E+08	2.44E+08	2.82E+08	2.21E+08	1.66E+08	2.50E+08	2.12E+08	2.01E+08	2.29E+08	0.88	-0.19	0.221812514
Q14240	Eukaryotic initiation factor 4A-II OS=Homo sapiens OX=9606 GN=EIF4A2 PE=1 SV=2	36	429	3	1.33E+07	1.11E+07	9.42E+06	1.25E+07	1.35E+07	1.08E+07	1.14E+07	1.22E+07	9.48E+06	1.01E+07	8.77E+06	8.16E+06	1.18E+07	1.00E+07	1.17	0.23	0.083816263
P10321	HLA class I histocompatibility antigen, C alpha chain OS=Homo sapiens OX=9606 GN=HLA-C PE=1 SV=3	36	444	3	5.37E+07	5.50E+07	4.31E+07	4.16E+07	4.37E+07	4.51E+07	2.27E+07	2.35E+07	2.00E+07	1.88E+07	1.58E+07	1.79E+07	4.70E+07	1.98E+07	2.38	1.25	1.18481E-05
P31150	Rab GDP dissociation inhibitor alpha OS=Homo sapiens OX=9606 GN=GDI1 PE=1 SV=2	31	206	3	5.97E+06	8.43E+06	8.86E+06	9.74E+06	8.40E+06	8.98E+06	1.08E+07	6.25E+06	8.77E+06	1.04E+07	8.32E+06	7.38E+06	8.40E+06	8.65E+06	0.97	-0.04	0.781109985
P62136	Serine/threonine-protein phosphatase PP1-alpha catalytic subunit OS=Homo sapiens OX=9606 GN=PPP1CA PE=1 SV=1	44	160	3	4.28E+07	3.98E+07	4.04E+07	4.01E+07	4.45E+07	4.47E+07	4.19E+07	4.19E+07	2.97E+07	3.50E+07	3.25E+07	3.09E+07	4.21E+07	3.53E+07	1.19	0.25	0.026594184
P20742	Pregnancy zone protein OS=Homo sapiens OX=9606 GN=PZP PE=1 SV=4	6	2368	3	5.93E+08	4.52E+08	3.65E+08	4.71E+08	4.11E+08	6.98E+08	6.05E+08	5.68E+08	3.27E+08	3.76E+08	5.59E+08	5.40E+08	4.98E+08	4.96E+08	1.00	0.01	0.972491362
P18085	ADP-ribosylation factor 4 OS=Homo sapiens OX=9606 GN=ARF4 PE=1 SV=3	45	332	3	2.34E+07	2.46E+07	2.71E+07	3.04E+07	2.81E+07	2.50E+07	2.34E+07	2.29E+07	2.34E+07	2.47E+07	2.34E+07	2.03E+07	2.64E+07	2.30E+07	1.15	0.20	0.023175988
Q9H0U4	Ras-related protein Rab-1B OS=Homo sapiens OX=9606 GN=RAB1B PE=1 SV=1	58	123	3	7.61E+07	8.93E+07	7.79E+07	6.34E+07	6.94E+07	7.01E+07	6.29E+07	6.07E+07	4.33E+07	4.74E+07	5.42E+07	4.61E+07	7.44E+07	5.25E+07	1.42	0.50	0.001287557
P35443	Thrombospondin-4 OS=Homo sapiens OX=9606 GN=THBS4 PE=1 SV=2	9	168	3	8.44E+06	7.79E+06	6.97E+06	1.03E+07	7.00E+06	9.97E+06	1.47E+07	1.18E+07	8.34E+06	8.61E+06	8.61E+06	9.05E+06	8.41E+06	1.02E+07	0.83	-0.28	0.178884902
P62879	Guanine nucleotide-binding protein G(I)/G(S)/G(T) subunit beta-2 OS=Homo sapiens OX=9606 GN=GNB2 PE=1 SV=3	27	206	3	6.34E+07	6.20E+07	5.83E+07	6.54E+07	6.18E+07	5.23E+07	6.54E+07	5.83E+07	5.55E+07	5.56E+07	4.78E+07	5.13E+07	6.05E+07	5.56E+07	1.09	0.12	0.148702095
P08567	Pleckstrin OS=Homo sapiens OX=9606 GN=PLEK PE=1 SV=3	10	40	3	2.12E+07	8.55E+06	1.36E+07	6.25E+06	1.93E+07	1.82E+07	3.75E+07	2.43E+07	1.36E+07	1.35E+07	1.81E+07	1.78E+07	1.45E+07	2.08E+07	0.70	-0.52	0.193424095
Q07021	Complement component 1 Q subcomponent-binding protein, mitochondrial OS=Homo sapiens OX=9606 GN=C1QBP PE=1 SV=1	25	40	3	3.06E+06	2.77E+06	6.81E+06	4.91E+06	7.85E+06	6.38E+06	2.20E+06	5.69E+06	1.92E+06	1.90E+06	2.77E+06	1.91E+06	5.29E+06	2.73E+06	1.94	0.95	0.036029525
P31153	S-adenosylmethionine synthase isoform type-2 OS=Homo sapiens OX=9606 GN=MAT2A PE=1 SV=1	17	124	3	1.93E+07	2.27E+07	1.98E+07	2.10E+07	2.01E+07	1.93E+07	2.59E+07	2.38E+07	2.56E+07	1.56E+07	2.27E+07	2.40E+07	2.04E+07	2.29E+07	0.89	-0.17	0.167193047
P20020	Plasma membrane calcium-transporting ATPase 1 OS=Homo sapiens OX=9606 GN=ATP2B1 PE=1 SV=4	8	62	3	6.19E+06	7.00E+06	6.35E+06	7.64E+06	6.93E+06	6.86E+06	4.62E+06	3.90E+06	4.73E+06	4.80E+06	4.14E+06	3.80E+06	6.83E+06	4.33E+06	1.58	0.66	4.977E-06
P15169	Carboxypeptidase N catalytic chain OS=Homo sapiens OX=9606 GN=CPN1 PE=1 SV=1	8	352	3	1.55E+08	9.48E+07	1.50E+08	9.23E+07	1.34E+08	1.22E+08	2.62E+08	1.32E+08	2.37E+08	1.39E+08	2.03E+08	2.10E+08	1.25E+08	1.97E+08	0.63	-0.66	0.018125241
Q9H2A2	2-aminomuconic semialdehyde dehydrogenase OS=Homo sapiens OX=9606 GN=ALDH8A1 PE=1 SV=1	10	97	3	2.31E+07	2.10E+07	1.22E+07	1.37E+07	1.22E+07	1.45E+07	2.75E+07	2.52E+07	1.48E+07	1.74E+07	1.82E+07	1.77E+07	1.61E+07	2.01E+07	0.80	-0.32	0.184816247
P35858	Insulin-like growth factor-binding protein complex acid labile subunit OS=Homo sapiens OX=9606 GN=IGFALS PE=1 SV=1	4	99	3	1.93E+07	1.75E+07	1.84E+07	1.74E+07	1.57E+07	1.97E+07	1.97E+07	2.27E+07	1.56E+07	2.03E+07	1.73E+07	1.87E+07	1.80E+07	1.91E+07	0.95	-0.08	0.407016903
Q15417	Calponin-3 OS=Homo sapiens OX=9606 GN=CNN3 PE=1 SV=1	21	26	3	3.52E+06	4.34E+06	8.02E+06	5.89E+06	5.16E+06	7.82E+06	4.58E+06	3.37E+06	4.60E+06	3.62E+06	3.19E+06	2.91E+06	5.79E+06	3.71E+06	1.56	0.64	0.038394801
Q92747	Actin-related protein 2/3 complex subunit 1A OS=Homo sapiens OX=9606 GN=ARPC1A PE=2 SV=2	17	44	3	9.27E+06	6.10E+06	8.02E+06	9.03E+06	7.54E+06	7.88E+06	1.00E+07	9.17E+06	9.54E+06	1.13E+07	5.31E+06	7.56E+06	7.97E+06	8.82E+06	0.90	-0.15	0.412274795
P43007	Neutral amino acid transporter A OS=Homo sapiens OX=9606 GN=SLC1A4 PE=1 SV=1	12	36	3	4.56E+06	5.10E+06	1.15E+07	5.65E+06	5.21E+06	4.98E+06	5.70E+06	5.74E+06	5.09E+06	3.62E+06	3.03E+06	6.61E+06	6.17E+06	4.96E+06	1.24	0.31	0.352695405
P00747	Plasminogen OS=Homo sapiens OX=9606 GN=PLG PE=1 SV=2	6	80	3	2.67E+07	2.37E+07	3.02E+07	3.31E+07	3.00E+07	2.73E+07	4.01E+07	4.24E+07	4.21E+07	4.27E+07	3.47E+07	3.20E+07	2.85E+07	3.90E+07	0.73	-0.45	0.001267297
P12277	Creatine kinase B-type OS=Homo sapiens OX=9606 GN=CKB PE=1 SV=1	18	28	3	3.69E+06	4.36E+05	1.40E+06	1.56E+06	1.76E+06	2.26E+06	2.96E+06	1.22E+06	1.67E+06	4.66E+06	1.42E+06	2.91E+06	1.85E+06	2.47E+06	0.75	-0.42	0.393617959

Supplementary Table 4. List of all proteins identified by label free proteomics analysis.

Accession	Description	Coverage [%]	# PSMs	# Unique Peptides	Amitriptyline 1	Amitriptyline 2	Amitriptyline 3	Amitriptyline 4	Amitriptyline 5	Amitriptyline 6	Vehicle 1	Vehicle 2	Vehicle 3	Vehicle 4	Vehicle 5	Vehicle 6	Average amitriptyline	Average vehicle	Ratio amitriptyline/ vehicle	Log 2 fold change (amitriptyline /vehicle)	P-value
P63000	Ras-related C3 botulinum toxin substrate 1 OS=Homo sapiens OX=9606 GN=RAC1 PE=1 SV=1	32	167	3	1.79E+07	1.57E+07	2.01E+07	1.65E+07	2.04E+07	2.06E+07	1.59E+07	1.37E+07	1.34E+07	2.28E+07	1.93E+07	1.74E+07	1.85E+07	1.71E+07	1.08	0.12	0.42026623
P62841	40S ribosomal protein S15 OS=Homo sapiens OX=9606 GN=RPS15 PE=1 SV=2	47	26	3	3.27E+06	5.01E+06	8.01E+06	7.10E+06	1.06E+07	6.31E+06	1.46E+06	2.96E+06	1.61E+06	1.78E+06	2.61E+06	1.79E+06	6.71E+06	2.04E+06	3.30	1.72	0.005324168
Q15404	Ras suppressor protein 1 OS=Homo sapiens OX=9606 GN=RSU1 PE=1 SV=3	18	29	3	7.41E+06	4.48E+06	4.96E+06	4.55E+06	5.36E+06	7.44E+06	1.04E+07	6.71E+06	5.89E+06	5.43E+06	5.33E+06	8.81E+06	5.70E+06	7.09E+06	0.80	-0.31	0.20287752
P31949	Protein S100-A11 OS=Homo sapiens OX=9606 GN=S100A11 PE=1 SV=2	54	24	3	8.61E+06	1.14E+07	9.65E+06	1.50E+07	1.41E+07	1.13E+07	7.54E+06	6.68E+06	7.26E+06	6.10E+06	6.39E+06	5.01E+06	1.17E+07	6.50E+06	1.80	0.85	0.002666145
P84095	Rho-related GTP-binding protein RhoG OS=Homo sapiens OX=9606 GN=RHOG PE=1 SV=1	24	33	3	4.73E+06	5.01E+06	3.07E+06	2.99E+06	3.57E+06	4.28E+06	3.83E+06	3.86E+06	3.58E+06	3.38E+06	2.18E+06	3.15E+06	3.94E+06	3.33E+06	1.18	0.24	0.191194835
Q9UBF2	Coatomer subunit gamma-2 OS=Homo sapiens OX=9606 GN=COPG2 PE=1 SV=1	8	40	3	6.94E+05	2.83E+05	8.91E+05	6.19E+05	1.25E+06	5.74E+05				3.02E+05			7.18E+05	3.02E+05	2.38	1.25	#DIV/0!
Q9GZS3	WD repeat-containing protein 61 OS=Homo sapiens OX=9606 GN=WDR61 PE=1 SV=1	23	16	3	2.14E+06	5.11E+06	2.03E+06	4.60E+06	3.37E+06	6.92E+06	2.87E+06	1.57E+06	2.31E+06	1.58E+06	4.04E+06	2.78E+06	4.03E+06	2.53E+06	1.59	0.67	0.122496316
P15927	Replication protein A 32 kDa subunit OS=Homo sapiens OX=9606 GN=RPA2 PE=1 SV=1	22	56	3	1.06E+07	9.71E+06	9.30E+06	1.04E+07	9.73E+06	1.28E+07	1.02E+07	9.23E+06	6.87E+06	9.28E+06	1.10E+07	5.91E+06	1.04E+07	8.76E+06	1.19	0.25	0.117672167
P62330	ADP-ribosylation factor 6 OS=Homo sapiens OX=9606 GN=ARF6 PE=1 SV=2	26	42	3	2.15E+06	1.06E+07	7.30E+06	4.05E+06	7.36E+06	4.72E+06	8.79E+06	9.68E+06	7.09E+06	4.89E+06	1.00E+07	8.31E+06	6.03E+06	8.13E+06	0.74	-0.43	0.184684461
Q8TAQ2	SWI/SNF complex subunit SMARCC2 OS=Homo sapiens OX=9606 GN=SMARCC2 PE=1 SV=1	4	14	3	6.48E+06	5.92E+06	5.77E+06	7.20E+06	4.67E+06	5.94E+06	2.73E+06	2.63E+06	2.21E+06	2.82E+06	3.00E+06	2.74E+06	6.00E+06	2.69E+06	2.23	1.16	9.06918E-05
P21810	Biglycan OS=Homo sapiens OX=9606 GN=BGN PE=1 SV=2	13	34	3	4.09E+06	4.35E+06	3.99E+06	5.36E+06	4.59E+06	6.28E+06	1.21E+07	5.59E+06	4.99E+06	8.68E+06	6.73E+06	7.98E+06	4.78E+06	7.68E+06	0.62	-0.68	0.038928016
Q9UHX1	Poly(U)-binding-splicing factor PUF60 OS=Homo sapiens OX=9606 GN=PUF60 PE=1 SV=1	12	18	3	3.79E+06	3.77E+06	2.48E+06	2.61E+06	2.48E+06	3.11E+06	2.28E+06	2.02E+06	1.27E+06	1.26E+06	1.45E+06	1.35E+06	3.04E+06	1.61E+06	1.89	0.92	0.001200311
P42285	Exosome RNA helicase MTR4 OS=Homo sapiens OX=9606 GN=MTREX PE=1 SV=3	5	12	3	3.09E+06	3.37E+06	4.06E+06	4.75E+06	5.08E+06	3.52E+06	1.65E+06	1.33E+06	1.20E+06	1.65E+06		6.84E+05	3.98E+06	1.30E+06	3.06	1.61	0.000117013
P21291	Cysteine and glycine-rich protein 1 OS=Homo sapiens OX=9606 GN=CSRP1 PE=1 SV=3	25	62	3	1.04E+07	9.40E+06	8.88E+06	7.95E+06	8.89E+06	9.35E+06	1.14E+07	9.83E+06	6.78E+06	9.81E+06	8.28E+06	8.63E+06	9.15E+06	9.11E+06	1.00	0.01	0.960538045
P17858	ATP-dependent 6-phosphofructokinase, liver type OS=Homo sapiens OX=9606 GN=PFKL PE=1 SV=6	9	49	3	1.82E+07	1.79E+07	1.92E+07	1.88E+07	1.73E+07	1.68E+07	2.21E+07	2.23E+07	2.23E+07	2.20E+07	1.92E+07	1.73E+07	1.80E+07	2.09E+07	0.86	-0.21	0.020948453
P32970	CD70 antigen OS=Homo sapiens OX=9606 GN=CD70 PE=1 SV=2	20	30	3	1.86E+07	1.13E+07	1.19E+07	1.16E+07	1.21E+07	1.14E+07	1.12E+07	8.49E+06	6.30E+06	5.81E+06	8.42E+06	9.84E+06	1.28E+07	8.34E+06	1.54	0.62	0.01192165
P62304	Small nuclear ribonucleoprotein E OS=Homo sapiens OX=9606 GN=SNRPE PE=1 SV=1	52	155	3	2.05E+07	1.82E+07	1.41E+07	1.18E+07	1.39E+07	2.10E+07	1.32E+07	1.50E+07	1.21E+07	1.20E+07	1.17E+07	1.49E+07	1.66E+07	1.31E+07	1.26	0.34	0.082594046
P42892	Endothelin-converting enzyme 1 OS=Homo sapiens OX=9606 GN=ECE1 PE=1 SV=2	7	31	3	5.52E+06	6.38E+06	3.66E+06	5.03E+06	5.27E+06	6.74E+06	4.78E+06	4.95E+06	1.77E+06	2.15E+06	4.78E+06	1.90E+06	5.43E+06	3.39E+06	1.60	0.68	0.029490098
P08708	40S ribosomal protein S17 OS=Homo sapiens OX=9606 GN=RPS17 PE=1 SV=2	29	14	3	1.13E+07	1.01E+07	7.76E+06	8.73E+06	9.87E+06	9.01E+06	6.50E+06	4.98E+06	3.76E+06	4.95E+06	5.07E+06	5.15E+06	9.46E+06	5.07E+06	1.87	0.90	5.49507E-05
O14828	Secretory carrier-associated membrane protein 3 OS=Homo sapiens OX=9606 GN=SCAMP3 PE=1 SV=3	16	26	3	9.22E+06	8.90E+06	6.76E+06	5.95E+06	5.74E+06	5.75E+06	6.17E+06	4.88E+06	3.37E+06	4.07E+06	3.78E+06	6.36E+05	7.05E+06	3.82E+06	1.85	0.89	0.009062845
Q99715	Collagen alpha-1(XII) chain OS=Homo sapiens OX=9606 GN=COL12A1 PE=1 SV=2	2	18	3	2.71E+06	2.87E+06	1.81E+06	1.95E+06	1.27E+06	1.85E+06	4.92E+06	1.88E+06	3.35E+06	4.70E+06	5.32E+06	4.21E+06	2.08E+06	4.06E+06	0.51	-0.97	0.010035328
P54578	Ubiquitin carboxyl-terminal hydrolase 14 OS=Homo sapiens OX=9606 GN=USP14 PE=1 SV=3	11	13	3	4.88E+06	8.00E+05	3.31E+06	4.02E+06	4.04E+06	3.42E+06	4.46E+06	9.48E+05	2.06E+06	4.26E+06	3.44E+06	2.48E+06	3.41E+06	2.94E+06	1.16	0.21	0.567349181
Q13443	Disintegrin and metalloproteinase domain-containing protein 9 OS=Homo sapiens OX=9606 GN=ADAM9 PE=1 SV=1	7	11	3	6.02E+06	3.13E+06	4.14E+06	4.21E+06	5.99E+06	5.46E+06	1.78E+06	2.37E+06	1.52E+06	2.39E+06	1.23E+06	1.82E+06	4.82E+06	1.85E+06	2.61	1.38	0.000883313
P48735	Isocitrate dehydrogenase [NADP], mitochondrial OS=Homo sapiens OX=9606 GN=IDH2 PE=1 SV=2	12	16	3	8.07E+06	7.62E+06	5.75E+06	5.16E+06	7.08E+06	5.65E+06	5.15E+06	2.95E+06	3.15E+06	4.54E+06	4.41E+06	2.38E+06	6.56E+06	3.76E+06	1.74	0.80	0.001747295
Q99627	COP9 signalosome complex subunit 8 OS=Homo sapiens OX=9606 GN=COPS8 PE=1 SV=1	28	18	3	1.59E+06	1.82E+06	9.21E+05	9.16E+05	7.90E+05	1.01E+06	7.07E+05				6.16E+05		1.17E+06	6.62E+05	1.77	0.83	0.030582465
Q86Y82	Syntaxin-12 OS=Homo sapiens OX=9606 GN=STX12 PE=1 SV=1	15	46	3	7.71E+06	6.97E+06	8.61E+06	1.01E+07	8.83E+06	6.06E+06	5.94E+06	4.74E+06	6.24E+06	5.87E+06	4.26E+06	4.68E+06	8.05E+06	5.29E+06	1.52	0.61	0.003671413
Q12906	Interleukin enhancer-binding factor 3 OS=Homo sapiens OX=9606 GN=ILF3 PE=1 SV=3	8	14	3	4.27E+06	5.29E+06	6.83E+06	4.64E+06	5.31E+06	3.90E+06	2.77E+06	2.79E+06	2.01E+06	2.61E+06	2.40E+06	2.74E+06	5.04E+06	2.55E+06	1.97	0.98	0.001455152
P14923	Junction plakoglobin OS=Homo sapiens OX=9606 GN=JUP PE=1 SV=3	10	39	3	3.99E+06	5.24E+06	2.88E+06	3.26E+06	5.90E+06	3.71E+06	1.97E+06	5.34E+06	1.43E+06	3.03E+06	2.72E+06	2.13E+06	4.16E+06	2.77E+06	1.50	0.59	0.089176842
P02748	Complement component C9 OS=Homo sapiens OX=9606 GN=C9 PE=1 SV=2	4	132	3	6.27E+07	6.17E+07	4.65E+07	5.40E+07	4.64E+07	5.22E+07	8.79E+07	8.75E+07	6.38E+07	6.45E+07	6.17E+07	6.05E+07	5.39E+07	7.10E+07	0.76	-0.40	0.023226034
Q13425	Beta-2-syntrophin OS=Homo sapiens OX=9606 GN=SNTB2 PE=1 SV=1	10	18	3	4.64E+06	5.69E+06	4.10E+06	3.35E+06	3.46E+06	4.92E+06	3.22E+06	4.35E+06	1.65E+06	1.92E+06	3.20E+06	2.69E+06	4.36E+06	2.84E+06	1.54	0.62	0.019035898
P62318	Small nuclear ribonucleoprotein Sm D3 OS=Homo sapiens OX=9606 GN=SNRPD3 PE=1 SV=1	32	68	3	3.52E+07	3.71E+07	3.50E+07	3.16E+07	4.61E+07	5.00E+07	2.68E+07	2.82E+07	2.68E+07	2.99E+07	2.18E+07	2.23E+07	3.92E+07	2.60E+07	1.51	0.59	0.00469065
P12821	Angiotensin-converting enzyme OS=Homo sapiens OX=9606 GN=ACE PE=1 SV=1	2	20	3	9.35E+06	8.17E+06	4.80E+06	8.74E+06	7.11E+06	6.21E+06	9.16E+06	7.56E+06	6.54E+06	6.35E+06	7.71E+06	5.37E+06	7.40E+06	7.12E+06	1.04	0.06	0.756807776
P52788	Spermine synthase OS=Homo sapiens OX=9606 GN=SMS PE=1 SV=2	20	10	3	1.42E+06	1.07E+06	6.34E+05	1.06E+06	8.10E+05	1.64E+06	1.33E+06			5.46E+05	8.68E+05	6.52E+05	1.11E+06	8.49E+05	1.30	0.38	0.304421139
P56537	Eukaryotic translation initiation factor 6 OS=Homo sapiens OX=9606 GN=EIF6 PE=1 SV=1	22	20	3	1.09E+07	1.14E+07	7.61E+06	9.90E+06	8.97E+06	1.01E+07	8.15E+06	8.81E+06	8.57E+06	7.42E+06	8.71E+06	6.76E+06	9.82E+06	8.07E+06	1.22	0.28	0.027317347
P25325	3-mercaptopyruvate sulfurtransferase OS=Homo sapiens OX=9606 GN=MPST PE=1 SV=3	16	23	3	4.51E+06	8.40E+06	2.51E+06	3.48E+06	3.86E+06	5.10E+06	5.29E+06	4.51E+06	2.40E+06	5.08E+06	4.45E+06	4.96E+06	4.64E+06	4.45E+06	1.04	0.06	0.841475641
P62070	Ras-related protein R-Ras2 OS=Homo sapiens OX=9606 GN=RRAS2 PE=1 SV=1	21	56	3	1.52E+07	1.58E+07	1.69E+07	1.71E+07	1.59E+07	1.48E+07	1.20E+07	1.18E+07	5.86E+06	1.33E+07	1.19E+07	8.65E+06	1.60E+07	1.06E+07	1.51	0.59	0.004012113
P28066	Proteasome subunit alpha type-5 OS=Homo sapiens OX=9606 GN=PSMA5 PE=1 SV=3	17	48	3	2.50E+07	2.49E+07	2.12E+07	2.53E+07	2.56E+07	7.09E+06	6.80E+06	7.02E+06	7.89E+06	6.72E+06	6.19E+06	5.17E+06	2.15E+07	6.63E+06	3.24	1.70	0.003796693
P28062	Proteasome subunit beta type-8 OS=Homo sapiens OX=9606 GN=PSMB8 PE=1 SV=3	13	36	3	9.14E+06	7.92E+06	1.14E+07	1.01E+07	1.03E+07	9.85E+06	7.86E+06	9.38E+06	8.91E+06	9.82E+06	8.68E+06	7.70E+06	9.77E+06	8.72E+06	1.12	0.16	0.104466351
Q15366	Poly(rC)-binding protein 2 OS=Homo sapiens OX=9606 GN=PCBP2 PE=1 SV=1	17	68	3	1.36E+07	1.16E+07	9.14E+06	9.14E+06	8.63E+06	9.61E+06	9.56E+06	5.60E+06	5.48E+06	8.69E+06	5.86E+06	7.08E+06	1.03E+07	7.04E+06	1.46	0.55	0.011962552
P62995	Transformer-2 protein homolog beta OS=Homo sapiens OX=9606 GN=TRA2B PE=1 SV=1	13	36	3	9.27E+06	1.01E+07	1.05E+07	1.26E+07	1.03E+07	1.01E+07	9.34E+06	1.21E+07	1.05E+07	1.07E+07	1.22E+07	7.62E+06	1.05E+07	1.04E+07	1.00	0.01	0.964546162
Q9BT78	COP9 signalosome complex subunit 4 OS=Homo sapiens OX=9606 GN=COPS4 PE=1 SV=1	13	30	3	5.20E+06	5.59E+06	4.81E+06	5.14E+06	4.67E+06	4.07E+06	4.67E+06	4.81E+06	4.62E+06	3.31E+06	4.76E+06	2.20E+06	4.91E+06	4.06E+06	1.21	0.27	0.123988575
P24752	Acetyl-CoA acetyltransferase, mitochondrial OS=Homo sapiens OX=9606 GN=ACAT1 PE=1 SV=1	11	14	3	5.76E+06	3.55E+06	4.13E+06	4.34E+06	3.78E+06	3.40E+06	3.80E+06	2.60E+06	2.36E+06	3.48E+06	9.22E+05	1.91E+06	4.16E+06	2.51E+06	1.66	0.73	0.014460507
Q14498	RNA-binding protein 39 OS=Homo sapiens OX=9606 GN=RBM39 PE=1 SV=2	8	28	3	3.47E+06	3.25E+06	4.14E+06	5.20E+06	3.96E+06	3.94E+06	1.82E+06	1.99E+06	1.69E+06	2.31E+06	1.44E+06	6.58E+05	3.99E+06	1.65E+06	2.42	1.27	8.35261E-05
P60660	Myosin light polypeptide 6 OS=Homo sapiens OX=9606 GN=MYL6 PE=1 SV=2	25	70	3	2.36E+07	2.42E+07	2.38E+07	2.49E+07	2.46E+07	2.26E+07	1.82E+07	2.06E+07	1.87E+07	2.20E+07	2.00E+07	1.99E+07	2.39E+07	1.99E+07	1.20	0.27	0.00023778
P51570	Galactokinase OS=Homo sapiens OX=9606 GN=GALK1 PE=1 SV=1	8	187	3	6.26E+07	7.38E+07	6.10E+07	5.45E+07	5.89E+07	5.70E+07	8.83E+07	9.21E+07	8.21E+07	8.70E+07	9.88E+07	6.72E+07	6.13E+07	8.59E+07	0.71	-0.49	0.001252825
Q06828	Fibromodulin OS=Homo sapiens OX=9606 GN=FMOD PE=1 SV=2	9	76	3	1.52E+07	1.40E+07	1.14E+07	1.51E+07	1.54E+07	1.70E+07	1.92E+07	2.41E+07	1.89E+07	1.72E+07	1.94E+07	1.66E+07	1.47E+07	1.92E+07	0.76	-0.39	0.007278878
Q16822	Phosphoenolpyruvate carboxykinase [GTP], mitochondrial OS=Homo sapiens OX=9606 GN=PCK2 PE=1 SV=4	6	24	3	4.59E+06	3.85E+06	3.89E+06	4.80E+06	4.64E+06	5.28E+06	3.83E+06	9.54E+05	1.75E+06	2.22E+06	2.30E+06	2.64E+06	4.51E+06	2.28E+06	1.97	0.98	0.00113164
Q8NFW8	N-acylneuraminate cytidylyltransferase OS=Homo sapiens OX=9606 GN=CMAS PE=1 SV=2	10	30	3	7.39E+06	8.88E+06	7.60E+06	8.82E+06	4.68E+06	7.06E+06	4.70E+06	5.79E+06	6.25E+06	6.44E+06	6.90E+06	5.80E+06	7.40E+06	5.98E+06	1.24	0.31	0.078832666
P24534	Elongation factor 1-beta OS=Homo sapiens OX=9606 GN=EEF1B2 PE=1 SV=3	16	42	3	1.08E+07	1.17E+07	1.48E+07	1.08E+07	1.07E+07	1.34E+07	9.42E+06	1.03E+07	1.01E+07	7.86E+06	8.19E+06	9.06E+06	1.20E+07	9.16E+06	1.31	0.39	0.007319431
Q93088	Betaine--homocysteine S-methyltransferase 1 OS=Homo sapiens OX=9606 GN=BHMT PE=1 SV=2	12	34	3	4.15E+06	4.29E+06	4.55E+06	4.46E+06	3.69E+06	3.26E+06	5.98E+06	5.90E+06	5.62E+06	5.52E+06	5.57E+06	4.22E+06	4.07E+06	5.47E+06	0.74	-0.43	0.001907594
Q14344	Guanine nucleotide-binding protein subunit alpha-13 OS=Homo sapiens OX=9606 GN=GNA13 PE=1 SV=2	16	133	3	3.73E+06	3.80E+06	3.37E+06	4.01E+06	3.33E+06	1.92E+06	7.67E+05	8.10E+05	6.35E+05		9.18E+05	5.27E+05	3.36E+06	7.31E+05	4.59	2.20	0.00025449
P42357	Histidine ammonia-lyase OS=Homo sapiens OX=9606 GN=HAL PE=1 SV=1	9	17	3	9.51E+05	2.44E+06	1.65E+06	2.54E+06	3.19E+06	2.51E+06	2.04E+06	1.81E+06	2.50E+06	2.06E+06	4.44E+06	1.40E+06	2.21E+06	2.38E+06	0.93	-0.10	0.773003589
P06753	Tropomyosin alpha-3 chain OS=Homo sapiens OX=9606 GN=TPM3 PE=1 SV=2	16	16	3	3.55E+06	1.82E+06	3.35E+06	3.65E+06	2.68E+06	1.59E+06	1.47E+06	1.08E+06	1.64E+06	8.20E+05	1.48E+06		2.78E+06	1.30E+06	2.14	1.09	0.008213688
Q15043	Zinc transporter ZIP14 OS=Homo sapiens OX=9606 GN=SLC39A14 PE=1 SV=3	9	18	3	2.23E+06	3.02E+06	3.21E+06	2.99E+06	3.05E+06	4.53E+06	1.55E+06	1.54E+06	1.60E+06	1.09E+06	1.56E+06	1.38E+06	3.17E+06	1.45E+06	2.18	1.13	0.001938642
Q14258	E3 ubiquitin/ISG15 ligase TRIM25 OS=Homo sapiens OX=9606 GN=TRIM25 PE=1 SV=2	7	26	3	4.94E+06	7.26E+06	6.45E+06	4.37E+06	6.24E+06	6.73E+06	2.70E+06	3.11E+06	4.68E+06	4.62E+06	2.41E+06	3.82E+06	6.00E+06	3.56E+06	1.69	0.75	0.002399013
P30876	DNA-directed RNA polymerase II subunit RPB2 OS=Homo sapiens OX=9606 GN=POLR2B PE=1 SV=1	4	16	3	3.38E+06	2.89E+06	2.35E+06	3.11E+06	3.10E+06	2.10E+06	1.79E+06	1.26E+06	1.07E+06	1.30E+06	1.35E+06	8.47E+05	2.82E+06	1.27E+06	2.23	1.15	0.000144073
P50213	Isocitrate dehydrogenase [NAD] subunit alpha, mitochondrial OS=Homo sapiens OX=9606 GN=IDH3A PE=1 SV=1	9	44	3	7.38E+06	6.77E+06	5.93E+06	7.00E+06	6.61E+06	6.31E+06	4.43E+06	4.86E+06	3.82E+06	4.38E+06	4.07E+06	3.60E+06	6.66E+06	4.19E+06	1.59	0.67	5.50661E-06
P26368	Splicing factor U2AF 65 kDa subunit OS=Homo sapiens OX=9606 GN=U2AF2 PE=1 SV=4	9	20	3	7.50E+06	6.12E+06	4.55E+06	4.56E+06	4.30E+06	7.44E+06	2.80E+06	2.74E+06	1.22E+06	6.51E+05	1.01E+06	2.06E+06	5.75E+06	1.75E+06	3.29	1.72	0.000434999
Q9UBQ5	Eukaryotic translation initiation factor 3 subunit K OS=Homo sapiens OX=9606 GN=EIF3K PE=1 SV=1	13	48	3	1.11E+07	1.30E+07	1.14E+07	9.71E+06	1.12E+07	1.12E+07	5.96E+06	8.94E+06	6.15E+06	5.96E+06	5.50E+06	6.26E+06	1.13E+07	6.46E+06	1.74	0.80	3.12453E-05
O00743	Serine/threonine-protein phosphatase 6 catalytic subunit OS=Homo sapiens OX=9606 GN=PPP6C PE=1 SV=1	11	23	3	7.37E+06	8.13E+06	4.96E+06	4.59E+06	6.09E+06	4.63E+06	5.66E+06	5.41E+06	3.98E+06	4.36E+06	4.42E+06	4.88E+06	5.96E+06	4.78E+06	1.25	0.32	0.123621273
Q01581	Hydroxymethylglutaryl-CoA synthase, cytoplasmic OS=Homo sapiens OX=9606 GN=HMGCS1 PE=1 SV=2	8	12	3	3.44E+06	2.87E+06	2.07E+06	3.21E+06	3.50E+06	3.33E+06	3.84E+06	3.10E+06	2.46E+06	3.01E+06	4.17E+06	3.08E+06	3.07E+06	3.28E+06	0.94	-0.09	0.551378719
Q8WXF1	Paraspeckle component 1 OS=Homo sapiens OX=9606 GN=PSPC1 PE=1 SV=1	9	34	3	2.04E+06	1.41E+06	2.19E+06	3.01E+06	1.66E+06	1.87E+06	1.92E+06	8.06E+05	1.06E+06		1.06E+06	1.07E+06	2.03E+06	1.18E+06	1.71	0.78	0.018844376
Q9H4G4	Golgi-associated plant pathogenesis-related protein 1 OS=Homo sapiens OX=9606 GN=GLIPR2 PE=1 SV=3	31	46	3	4.73E+06	5.24E+06	4.39E+06	5.07E+06	4.68E+06	4.59E+06	3.83E+06	4.21E+06	4.69E+06	4.85E+06	3.89E+06	3.52E+06	4.78E+06	4.16E+06	1.15	0.20	0.036839379
O43681	ATPase ASNA1 OS=Homo sapiens OX=9606 GN=ASNA1 PE=1 SV=2	11	38	3	5.02E+06	8.14E+06	7.14E+06	6.94E+06	4.99E+06	6.08E+06	4.61E+06	5.92E+06	4.61E+06	4.63E+06	4.02E+06	5.95E+06	6.39E+06	4.96E+06	1.29	0.37	0.044075559
Q9Y266	Nuclear migration protein nudC OS=Homo sapiens OX=9606 GN=NUDC PE=1 SV=1	12	26	3	9.67E+06	1.09E+07	1.25E+07	9.19E+06	1.11E+07	7.72E+06	5.47E+06	6.95E+06	5.23E+06	7.00E+06	4.94E+06	3.78E+06	1.02E+07	5.56E+06	1.83	0.87	0.000378411
P24928	DNA-directed RNA polymerase II subunit RPB1 OS=Homo sapiens OX=9606 GN=POLR2A PE=1 SV=2	2	7	3	1.83E+06	1.39E+06	2.05E+06	2.48E+06	2.34E+06	1.59E+06		1.85E+06	1.86E+06	1.63E+06	1.77E+06	1.34E+06	1.95E+06	1.69E+06	1.15	0.20	0.234559605
Q7L5N1	COP9 signalosome complex subunit 6 OS=Homo sapiens OX=9606 GN=COPS6 PE=1 SV=1	17	23	3	6.92E+06	8.18E+06	7.36E+06	5.36E+06	7.01E+06	4.40E+06	7.10E+06	4.36E+06	4.23E+06	6.48E+06	4.76E+06	5.25E+06	6.54E+06	5.36E+06	1.22	0.29	0.146007342
O95372	Acyl-protein thioesterase 2 OS=Homo sapiens OX=9606 GN=LYPLA2 PE=1 SV=1	18	13	3	2.36E+06	4.70E+06	5.16E+06	3.54E+06	4.05E+06	5.29E+06	6.12E+06	5.87E+06	6.71E+06	3.72E+06	5.30E+06	4.22E+06	4.18E+06	5.32E+06	0.79	-0.35	0.112929322
P19784	Casein kinase II subunit alpha' OS=Homo sapiens OX=9606 GN=CSNK2A2 PE=1 SV=1	12	15	3	6.34E+07	1.48E+08	6.95E+07	4.96E+07	6.84E+07	1.19E+08	8.41E+07	1.94E+08	3.99E+06	4.54E+07	1.43E+08	1.00E+08	8.63E+07	9.50E+07	0.91	-0.14	0.790388108
Q05519	Serine/arginine-rich splicing factor 11 OS=Homo sapiens OX=9606 GN=SRSF11 PE=1 SV=1	8	24	3	8.74E+06	7.06E+06	6.56E+06	5.45E+06	4.80E+06	5.22E+06	3.81E+06	4.28E+06	4.63E+06	2.55E+06	3.90E+06	4.07E+06	6.31E+06	3.87E+06	1.63	0.70	0.00765212
P08123	Collagen alpha-2(I) chain OS=Homo sapiens OX=9606 GN=COL1A2 PE=1 SV=7	3	12	3	1.69E+06	1.65E+06	2.03E+06	1.81E+06	1.68E+06	1.77E+06	3.25E+06	2.74E+06	3.76E+06	2.08E+06	2.86E+06	8.95E+06	1.77E+06	3.94E+06	0.45	-1.15	0.088473523
Q9Y5X3	Sorting nexin-5 OS=Homo sapiens OX=9606 GN=SNX5 PE=1 SV=1	10	40	3	6.43E+06	6.32E+06	3.75E+06	3.91E+06	4.07E+06	4.64E+06	6.16E+06	6.38E+06	4.31E+06	5.23E+06	4.82E+06	4.29E+06	4.85E+06	5.20E+06	0.93	-0.10	0.591266506
P35625	Metalloproteinase inhibitor 3 OS=Homo sapiens OX=9606 GN=TIMP3 PE=1 SV=2	16	40	3	5.91E+06	6.52E+06	5.85E+06	6.06E+06	6.59E+06	5.77E+06	7.90E+06	8.52E+06	1.32E+07	7.76E+06	7.55E+06	1.19E+07	6.12E+06	9.46E+06	0.65	-0.63	0.019232046
P37837	Transaldolase OS=Homo sapiens OX=9606 GN=TALDO1 PE=1 SV=2	12	42	3	9.87E+06	1.04E+07	9.93E+06	9.17E+06	9.26E+06	8.91E+06	7.17E+06	6.61E+06	6.74E+06	4.16E+06	6.50E+06	5.65E+06	9.60E+06	6.14E+06	1.56	0.64	0.000160045
P84098	60S ribosomal protein L19 OS=Homo sapiens OX=9606 GN=RPL19 PE=1 SV=1	17	32	3	1.57E+07	1.58E+07	1.50E+07	1.76E+07	1.49E+07	1.40E+07	9.77E+06	9.29E+06	1.15E+07	1.11E+07	1.00E+07	9.07E+06	1.55E+07	1.01E+07	1.53	0.61	9.46844E-06
Q96KP1	Exocyst complex component 2 OS=Homo sapiens OX=9606 GN=EXOC2 PE=1 SV=1	6	9	3	2.91E+06	1.13E+06	1.94E+06	5.53E+05	1.90E+06	2.82E+06	1.68E+06	7.22E+05	1.37E+06	2.71E+05	7.30E+05	2.27E+06	1.88E+06	1.17E+06	1.60	0.68	0.177895094
P02647	Apolipoprotein A-I OS=Homo sapiens OX=9606 GN=APOA1 PE=1 SV=1	15	6	3	3.16E+06	3.59E+06	2.33E+06	3.00E+06	4.17E+06	4.06E+06	8.49E+06	8.66E+06	6.93E+06	3.96E+06	7.00E+06	7.10E+06	3.39E+06	7.02E+06	0.48	-1.05	0.002048351
P09382	Galectin-1 OS=Homo sapiens OX=9606 GN=LGALS1 PE=1 SV=2	26	90	3	3.17E+07	3.24E+07	3.37E+07	3.36E+07	3.64E+07	3.07E+07	2.79E+07	2.72E+07	2.82E+07	2.89E+07	2.58E+07	2.30E+07	3.31E+07	2.68E+07	1.23	0.30	0.000399451
Q8TAT6	Nuclear protein localization protein 4 homolog OS=Homo sapiens OX=9606 GN=NPLOC4 PE=1 SV=3	7	24	3	3.37E+06	2.39E+06	2.33E+06	3.68E+06	2.01E+06	3.09E+06	1.68E+06	1.79E+06	2.81E+06	3.12E+06	1.48E+06	1.26E+06	2.81E+06	2.03E+06	1.39	0.47	0.084756209
Q92466	DNA damage-binding protein 2 OS=Homo sapiens OX=9606 GN=DDB2 PE=1 SV=1	10	14	3	5.29E+06	5.72E+06	5.36E+06	4.12E+06	4.69E+06	3.89E+06	4.01E+06	3.87E+06	4.11E+06	4.43E+06	4.19E+06	3.43E+06	4.85E+06	4.01E+06	1.21	0.27	0.038324733
Q14624	Inter-alpha-trypsin inhibitor heavy chain H4 OS=Homo sapiens OX=9606 GN=ITIH4 PE=1 SV=4	3	167	3	1.55E+08	1.34E+08	1.11E+08	1.73E+08	1.38E+08	1.09E+08	1.59E+08	1.43E+08	2.16E+08	2.36E+08	1.44E+08	1.52E+08	1.37E+08	1.75E+08	0.78	-0.35	0.082923911
Q13557	Calcium/calmodulin-dependent protein kinase type II subunit delta OS=Homo sapiens OX=9606 GN=CAMK2D PE=1 SV=3	10	6	3	1.84E+06	1.06E+06	2.07E+06	2.32E+06	1.56E+06	2.02E+06	1.72E+06	1.07E+06	1.12E+06	1.58E+06	1.43E+06	1.37E+06	1.81E+06	1.38E+06	1.31	0.39	0.074753917
O95084	Serine protease 23 OS=Homo sapiens OX=9606 GN=PRSS23 PE=1 SV=1	9	38	3	5.55E+06	5.15E+06	4.81E+06	5.70E+06	4.80E+06	5.01E+06	1.08E+07	9.62E+06	9.50E+06	1.05E+07	9.60E+06	9.04E+06	5.17E+06	9.85E+06	0.52	-0.93	5.63232E-07
P27658	Collagen alpha-1(VIII) chain OS=Homo sapiens OX=9606 GN=COL8A1 PE=1 SV=2	7	64	3	1.24E+07	1.27E+07	7.58E+06	8.73E+06	8.28E+06	1.15E+07	1.46E+07	1.20E+07	8.10E+06	8.23E+06	1.22E+07	1.27E+07	1.02E+07	1.13E+07	0.90	-0.15	0.447102809
P49773	Histidine triad nucleotide-binding protein 1 OS=Homo sapiens OX=9606 GN=HINT1 PE=1 SV=2	24	33	3	1.20E+07	1.53E+07	1.42E+07	1.56E+07	1.32E+07	1.39E+07	1.08E+07	1.32E+07	9.12E+06	1.24E+07	8.93E+06	1.04E+07	1.40E+07	1.08E+07	1.30	0.38	0.005186774
O75400	Pre-mRNA-processing factor 40 homolog A OS=Homo sapiens OX=9606 GN=PRPF40A PE=1 SV=2	4	44	3	5.02E+06	5.99E+06	4.91E+06	4.01E+06	6.58E+06	4.94E+06	5.25E+06	5.88E+06	4.48E+06	3.63E+06	4.34E+06	3.47E+06	5.24E+06	4.51E+06	1.16	0.22	0.195267676
O00391	Sulfhydryl oxidase 1 OS=Homo sapiens OX=9606 GN=QSOX1 PE=1 SV=3	5	46	3	8.67E+06	9.00E+06	6.76E+06	8.63E+06	8.76E+06	7.09E+06	1.30E+07	1.26E+07	1.09E+07	1.07E+07	1.08E+07	1.10E+07	8.15E+06	1.15E+07	0.71	-0.50	0.000170821
Q9NR19	Acetyl-coenzyme A synthetase, cytoplasmic OS=Homo sapiens OX=9606 GN=ACSS2 PE=1 SV=1	6	19	3	2.32E+06	3.03E+06	3.02E+06	3.51E+06	2.68E+06	2.44E+06	3.77E+06	3.69E+06	3.14E+06	3.89E+06	3.54E+06	2.79E+06	2.83E+06	3.47E+06	0.82	-0.29	0.028903981
Q14008	Cytoskeleton-associated protein 5 OS=Homo sapiens OX=9606 GN=CKAP5 PE=1 SV=3	2	10	3	6.38E+06	5.81E+06	5.81E+06	6.09E+06	5.81E+06	5.33E+06	4.33E+06	1.47E+06	3.03E+06	1.66E+06	3.24E+06	2.55E+06	5.87E+06	2.71E+06	2.16	1.11	0.000436591
Q96TA1	Protein Niban 2 OS=Homo sapiens OX=9606 GN=NIBAN2 PE=1 SV=3	6	11	3	2.19E+06	1.15E+06	9.30E+05	3.84E+05	2.18E+06	2.08E+06	3.23E+06	1.98E+06	1.56E+06	1.66E+06	1.55E+06	1.67E+06	1.48E+06	1.94E+06	0.76	-0.39	0.292581822
Q92882	Osteoclast-stimulating factor 1 OS=Homo sapiens OX=9606 GN=OSTF1 PE=1 SV=2	24	16	3	2.87E+06	3.20E+06	2.53E+06	3.28E+06	1.83E+06	2.02E+06	3.66E+06	3.69E+06	1.79E+06	1.86E+06	1.72E+06	2.75E+06	2.62E+06	2.58E+06	1.02	0.02	0.926527936
Q92905	COP9 signalosome complex subunit 5 OS=Homo sapiens OX=9606 GN=COPS5 PE=1 SV=4	14	12	3	3.85E+06	3.72E+06	3.08E+06	4.02E+06	4.06E+06	3.34E+06	3.76E+06	2.35E+06	7.62E+05	2.26E+06	3.33E+06	9.73E+05	3.68E+06	2.24E+06	1.64	0.72	0.031820873
O15145	Actin-related protein 2/3 complex subunit 3 OS=Homo sapiens OX=9606 GN=ARPC3 PE=1 SV=3	13	52	3	1.27E+07	1.21E+07	1.23E+07	1.18E+07	1.03E+07	1.03E+07	1.17E+07	1.10E+07	1.12E+07	1.05E+07	1.03E+07	9.65E+06	1.16E+07	1.07E+07	1.09	0.12	0.107525469
Q02413	Desmoglein-1 OS=Homo sapiens OX=9606 GN=DSG1 PE=1 SV=2	5	10	3	2.01E+06	2.05E+06	2.32E+05	1.34E+06	9.91E+05	1.73E+06	3.47E+05	1.83E+06	3.49E+05	1.58E+06	1.35E+06	3.62E+05	1.39E+06	9.69E+05	1.44	0.52	0.3164794
P53992	Protein transport protein Sec24C OS=Homo sapiens OX=9606 GN=SEC24C PE=1 SV=3	4	10	3	1.39E+06	4.83E+05	1.28E+06	1.28E+05	1.44E+06	1.08E+06	1.21E+06		3.26E+05	6.46E+05	6.90E+05	6.11E+05	9.66E+05	6.96E+05	1.39	0.47	0.330388582
P01009	Alpha-1-antitrypsin OS=Homo sapiens OX=9606 GN=SERPINA1 PE=1 SV=3	8	6	3							6.57E+05					1.06E+07		5.60E+06			
P35052	Glypican-1 OS=Homo sapiens OX=9606 GN=GPC1 PE=1 SV=2	8	29	3	6.39E+06	6.25E+06	5.66E+06	6.03E+06	4.05E+06	5.66E+06	6.10E+06	6.12E+06	4.05E+06	5.19E+06	3.73E+06	2.66E+06	5.67E+06	4.64E+06	1.22	0.29	0.15898109
Q86TI2	Dipeptidyl peptidase 9 OS=Homo sapiens OX=9606 GN=DPP9 PE=1 SV=3	4	27	3	5.30E+06	5.15E+06	4.77E+06	4.33E+06	4.25E+06	4.50E+06	4.52E+06	3.94E+06	3.64E+06	4.55E+06	4.49E+06	3.70E+06	4.72E+06	4.14E+06	1.14	0.19	0.0435091
Q76LX8	A disintegrin and metalloproteinase with thrombospondin motifs 13 OS=Homo sapiens OX=9606 GN=ADAMTS13 PE=1 SV=1	2	84	3	9.85E+06	1.37E+07	1.44E+07	1.52E+07	1.52E+07	1.46E+07	2.32E+07	2.10E+07	2.41E+07	1.78E+07	1.86E+07	1.88E+07	1.38E+07	2.06E+07	0.67	-0.57	0.000639763

Supplementary Table 4. List of all proteins identified by label free proteomics analysis.

Accession	Description	Coverage [%]	# PSMs	# Unique Peptides	Amitriptyline 1	Amitriptyline 2	Amitriptyline 3	Amitriptyline 4	Amitriptyline 5	Amitriptyline 6	Vehicle 1	Vehicle 2	Vehicle 3	Vehicle 4	Vehicle 5	Vehicle 6	Average amitriptyline	Average vehicle	Ratio amitriptyline/ vehicle	Log 2 fold change (amitriptyline /vehicle)	P-value
Q14117	Dihydropyrimidinase OS=Homo sapiens OX=9606 GN=DPYS PE=1 SV=1	6	40	3	3.43E+06	4.63E+06	5.54E+06	6.17E+06	5.23E+06	4.08E+06	6.61E+06	6.97E+06	8.02E+06	8.69E+06	7.28E+06	5.87E+06	4.85E+06	7.24E+06	0.67	-0.58	0.002029804
Q9NY12	H/ACA ribonucleoprotein complex subunit 1 OS=Homo sapiens OX=9606 GN=GAR1 PE=1 SV=1	16	30	3	4.55E+06	2.57E+06	4.22E+06	3.93E+06	3.79E+06	4.76E+06	2.37E+06	3.31E+06	2.52E+06	3.14E+06	2.82E+06	2.05E+06	3.97E+06	2.70E+06	1.47	0.56	0.008748343
P40926	Malate dehydrogenase, mitochondrial OS=Homo sapiens OX=9606 GN=MDH2 PE=1 SV=3	11	25	3	2.25E+06	9.30E+05	1.23E+06	1.56E+06	1.31E+06	1.33E+06		3.56E+06	7.83E+05	1.54E+06	1.15E+06	1.55E+06	1.44E+06	1.72E+06	0.84	-0.26	0.609708468
P78344	Eukaryotic translation initiation factor 4 gamma 2 OS=Homo sapiens OX=9606 GN=EIF4G2 PE=1 SV=1	3	8	3	1.11E+06	8.26E+05	1.50E+06	7.72E+05	9.79E+05	1.68E+06	6.40E+05	1.89E+06	2.91E+05		1.18E+06	6.40E+05	1.14E+06	9.28E+05	1.23	0.30	0.51971241
P20042	Eukaryotic translation initiation factor 2 subunit 2 OS=Homo sapiens OX=9606 GN=EIF2S2 PE=1 SV=2	9	22	3	5.03E+06	4.08E+06	5.94E+06	7.06E+06	6.62E+06	5.32E+06	3.77E+06	3.81E+06	4.53E+06	3.86E+06	3.74E+06	3.78E+06	5.68E+06	3.91E+06	1.45	0.54	0.009558287
O75832	26S proteasome non-ATPase regulatory subunit 10 OS=Homo sapiens OX=9606 GN=PSMD10 PE=1 SV=1	19	16	3	4.33E+06	5.20E+06	4.15E+06	4.56E+06	6.28E+06	5.32E+06	4.12E+06	4.53E+06	4.87E+06	4.10E+06	3.58E+06	2.36E+06	4.97E+06	3.93E+06	1.27	0.34	0.056710384
P26196	Probable ATP-dependent RNA helicase DDX6 OS=Homo sapiens OX=9606 GN=DDX6 PE=1 SV=2	8	15	3	3.29E+06	3.14E+06	2.29E+06	3.33E+06	1.83E+06	2.45E+06	2.21E+06	1.09E+06	6.47E+05	1.18E+06	1.60E+06	1.90E+06	2.72E+06	1.44E+06	1.89	0.92	0.003985184
Q9HC38	Glyoxalase domain-containing protein 4 OS=Homo sapiens OX=9606 GN=GLOD4 PE=1 SV=1	10	47	3	9.93E+06	1.02E+07	1.11E+07	9.23E+06	9.46E+06	1.08E+07	1.32E+07	1.28E+07	1.33E+07	1.19E+07	1.37E+07	1.05E+07	1.01E+07	1.26E+07	0.80	-0.32	0.002170711
P30044	Peroxiredoxin-5, mitochondrial OS=Homo sapiens OX=9606 GN=PRDX5 PE=1 SV=4	30	6	3	1.33E+06	6.28E+05	3.22E+06	2.47E+06	9.15E+05	3.91E+06					1.03E+06	2.48E+05	2.08E+06	6.37E+05	3.26	1.71	0.08373926
Q13045	Protein flightless-1 homolog OS=Homo sapiens OX=9606 GN=FLII PE=1 SV=2	3	26	3	5.45E+06	3.77E+06	4.98E+06	5.44E+06	4.84E+06	4.89E+06	4.21E+06	3.45E+06	2.83E+06	4.22E+06	3.43E+06	2.74E+06	4.89E+06	3.48E+06	1.41	0.49	0.003001446
P48444	Coatomer subunit delta OS=Homo sapiens OX=9606 GN=ARCN1 PE=1 SV=1	7	10	3	3.91E+06	3.70E+06	3.39E+06	3.58E+06	3.03E+06	2.16E+06	2.04E+06	1.78E+06	2.15E+06	2.59E+06	1.30E+06	8.04E+05	3.29E+06	1.78E+06	1.85	0.89	0.002042845
P83731	60S ribosomal protein L24 OS=Homo sapiens OX=9606 GN=RPL24 PE=1 SV=1	16	52	3	2.29E+07	2.14E+07	2.16E+07	2.21E+07	2.48E+07	2.06E+07	1.48E+07	1.55E+07	1.28E+07	1.39E+07	1.25E+07	1.08E+07	2.22E+07	1.34E+07	1.66	0.73	2.56297E-06
O00339	Matrilin-2 OS=Homo sapiens OX=9606 GN=MATN2 PE=1 SV=4	5	13	3		3.03E+06			1.05E+06			4.13E+06	1.56E+06		3.02E+06	1.19E+06	2.04E+06	2.48E+06	0.82	-0.28	0.751689528
Q9NQW7	Xaa-Pro aminopeptidase 1 OS=Homo sapiens OX=9606 GN=XPNPEP1 PE=1 SV=3	5	32	3	5.13E+06	5.81E+06	3.12E+06	3.92E+06	3.59E+06	3.14E+06	7.20E+06	8.32E+06	7.01E+06	6.54E+06	6.08E+06	5.48E+06	4.12E+06	6.77E+06	0.61	-0.72	0.001427047
P62851	40S ribosomal protein S25 OS=Homo sapiens OX=9606 GN=RPS25 PE=1 SV=1	22	93	3	5.64E+07	5.37E+07	6.71E+07	5.58E+07	7.96E+07	7.19E+07	4.00E+07	3.70E+07	4.91E+07	5.63E+07	4.21E+07	4.10E+07	6.41E+07	4.43E+07	1.45	0.53	0.004167885
Q14847	LIM and SH3 domain protein 1 OS=Homo sapiens OX=9606 GN=LASP1 PE=1 SV=2	13	42	3	5.68E+06	6.65E+06	6.00E+06	5.56E+06	5.79E+06	5.46E+06	3.43E+06	3.40E+06	4.39E+06	5.69E+06	3.31E+06	2.42E+06	5.86E+06	3.77E+06	1.55	0.63	0.004726468
Q96RP9	Elongation factor G, mitochondrial OS=Homo sapiens OX=9606 GN=GFM1 PE=1 SV=2	4	10	3	3.10E+06	2.99E+06	2.11E+06	2.58E+06	2.25E+06	2.17E+06	1.77E+06	8.56E+05	1.32E+06	1.15E+06	1.46E+06	7.14E+05	2.53E+06	1.21E+06	2.09	1.06	0.000243346
Q9Y371	Endophilin-B1 OS=Homo sapiens OX=9606 GN=SH3GLB1 PE=1 SV=1	8	18	3	1.53E+06	4.09E+06	3.57E+06	3.87E+06	3.40E+06	3.63E+06	3.24E+06	3.89E+06	3.64E+06	3.52E+06	3.00E+06	2.84E+06	3.35E+06	3.35E+06	1.00	0.00	0.985668439
P49902	Cytosolic purine 5'-nucleotidase OS=Homo sapiens OX=9606 GN=NT5C2 PE=1 SV=1	8	5	3	1.33E+06	5.91E+05	8.37E+05	6.10E+05	9.24E+05	6.89E+05			1.08E+06		8.37E+05	6.84E+05	8.30E+05	8.68E+05	0.96	-0.07	0.82136733
P16885	1-phosphatidylinositol 4,5-bisphosphate phosphodiesterase gamma-2 OS=Homo sapiens OX=9606 GN=PLCG2 PE=1 SV=4	3	14	3	2.60E+06	2.35E+06	2.25E+06	1.75E+06	1.82E+06	2.63E+06	3.10E+06	3.39E+06	4.39E+06	3.84E+06	2.75E+06	2.64E+06	2.23E+06	3.36E+06	0.67	-0.59	0.00746715
P05198	Eukaryotic translation initiation factor 2 subunit 1 OS=Homo sapiens OX=9606 GN=EIF2S1 PE=1 SV=3	9	62	3	1.57E+07	1.64E+07	1.48E+07	1.58E+07	1.60E+07	1.46E+07	1.44E+07	1.58E+07	1.49E+07	1.55E+07	1.41E+07	1.16E+07	1.56E+07	1.44E+07	1.08	0.11	0.134856856
P27701	CD82 antigen OS=Homo sapiens OX=9606 GN=CD82 PE=1 SV=1	15	18	3	3.48E+06	2.75E+06	5.80E+06	9.13E+06	4.64E+06	3.87E+06	2.62E+06	6.00E+06	8.98E+06	6.31E+06	4.27E+06	4.88E+06	4.95E+06	5.51E+06	0.90	-0.16	0.670783977
P49959	Double-strand break repair protein MRE11 OS=Homo sapiens OX=9606 GN=MRE11 PE=1 SV=3	6	6	3	1.83E+06	1.15E+06	4.95E+05	8.97E+05	1.09E+06	1.10E+06	1.04E+06	9.11E+05			2.38E+05	6.12E+05	1.09E+06	7.00E+05	1.56	0.64	0.159461392
P14324	Farnesyl pyrophosphate synthase OS=Homo sapiens OX=9606 GN=FDPS PE=1 SV=4	11	10	3	5.64E+05	8.04E+05	1.61E+06	1.70E+06	1.44E+06	2.21E+06	1.48E+06		1.65E+06	7.02E+05	1.30E+06	1.06E+06	1.39E+06	1.24E+06	1.12	0.17	0.627853302
Q6NZI2	Caveolae-associated protein 1 OS=Homo sapiens OX=9606 GN=CAVIN1 PE=1 SV=1	8	26	3	5.89E+06	6.94E+06	8.46E+06	8.54E+06	8.49E+06	6.84E+06	3.24E+06	2.50E+06	2.18E+06	2.57E+06	2.15E+06	1.95E+06	7.53E+06	2.43E+06	3.09	1.63	2.60723E-05
O60264	SWI/SNF-related matrix-associated actin-dependent regulator of chromatin subfamily A member 5 OS=Homo sapiens OX=9606 GN=SMARCA5 PE=1 SV=1	3	17	3	4.11E+06	4.77E+06	3.77E+06	4.28E+06	3.75E+06	3.72E+06	1.78E+06	2.12E+06	1.49E+06	2.06E+06	2.28E+06	2.20E+06	4.07E+06	1.99E+06	2.05	1.03	3.27802E-06
O75116	Rho-associated protein kinase 2 OS=Homo sapiens OX=9606 GN=ROCK2 PE=1 SV=4	3	7	3	7.20E+05	9.83E+05	1.19E+06	9.49E+05	4.24E+05	1.92E+06	2.33E+06	1.43E+06	1.82E+06	1.74E+06	1.54E+06	1.57E+06	1.03E+06	1.74E+06	0.59	-0.75	0.018961965
Q15057	Arf-GAP with coiled-coil, ANK repeat and PH domain-containing protein 2 OS=Homo sapiens OX=9606 GN=ACAP2 PE=1 SV=3	6	23	3	3.98E+06	7.92E+06	1.13E+07	1.85E+07	1.19E+07	9.56E+06	9.70E+06	8.77E+06	7.41E+06	3.37E+06	3.39E+06	5.91E+06	1.05E+07	6.42E+06	1.64	0.71	0.108334526
P23381	Tryptophan--tRNA ligase, cytoplasmic OS=Homo sapiens OX=9606 GN=WARS PE=1 SV=2	9	9	3	1.93E+06	1.76E+06	1.93E+06	1.43E+06	5.95E+05	1.37E+06	5.69E+05	3.57E+06		1.06E+06	6.82E+05	4.95E+05	1.50E+06	1.28E+06	1.18	0.24	0.729472475
O95445	Apolipoprotein M OS=Homo sapiens OX=9606 GN=APOM PE=1 SV=2	18	58	3	4.67E+07	5.28E+07	4.75E+07	4.04E+07	4.82E+07	3.66E+07	6.34E+07	6.73E+07	5.99E+07	4.70E+07	5.17E+07	4.94E+07	4.54E+07	5.64E+07	0.80	-0.32	0.024820283
P10643	Complement component C7 OS=Homo sapiens OX=9606 GN=C7 PE=1 SV=2	2	66	3	2.86E+07	2.55E+07	1.75E+07	2.02E+07	1.89E+07	2.26E+07	3.47E+07	4.28E+07	2.34E+07	2.49E+07	2.54E+07	2.71E+07	2.22E+07	2.97E+07	0.75	-0.42	0.066148483
O14744	Protein arginine N-methyltransferase 5 OS=Homo sapiens OX=9606 GN=PRMT5 PE=1 SV=4	5	20	3	9.49E+06	1.01E+07	8.38E+06	8.59E+06	8.66E+06	2.17E+06	9.44E+06	2.00E+06	1.51E+06	9.03E+06	7.46E+06	1.82E+06	7.89E+06	5.21E+06	1.52	0.60	0.201352765
P06132	Uroporphyrinogen decarboxylase OS=Homo sapiens OX=9606 GN=UROD PE=1 SV=2	9	34	3	7.46E+06	6.25E+06	5.11E+06	3.64E+06	6.43E+06	5.66E+06	7.21E+06	7.85E+06	3.98E+06	7.70E+06	6.87E+06	7.15E+06	5.76E+06	6.79E+06	0.85	-0.24	0.218458482
P23229	Integrin alpha-6 OS=Homo sapiens OX=9606 GN=ITGA6 PE=1 SV=5	3	12	3			6.07E+05	6.50E+05	7.27E+05		4.19E+05		6.23E+05	7.26E+05	5.38E+05	8.45E+05	6.61E+05	6.30E+05	1.05	0.07	0.7171384
P08514	Integrin alpha-IIb OS=Homo sapiens OX=9606 GN=ITGA2B PE=1 SV=3	4	6	3	3.92E+05		5.04E+05	2.18E+05	1.42E+06	1.11E+06	1.21E+06	7.58E+05		9.91E+05	5.52E+05	4.89E+06	7.29E+05	1.68E+06	0.43	-1.20	0.313874082
Q969P0	Immunoglobulin superfamily member 8 OS=Homo sapiens OX=9606 GN=IGSF8 PE=1 SV=1	5	23	3	3.35E+06	2.72E+06	3.64E+06	4.16E+06	4.29E+06	3.81E+06	2.21E+06	2.14E+06	2.03E+06	2.13E+06	2.16E+06	1.83E+06	3.66E+06	2.08E+06	1.76	0.82	0.000820177
Q00059	Transcription factor A, mitochondrial OS=Homo sapiens OX=9606 GN=TFAM PE=1 SV=1	12	30	3	7.65E+06	8.65E+06	8.05E+06	9.73E+06	8.79E+06	7.88E+06	3.69E+06	7.24E+06	7.11E+06	6.92E+06	3.76E+06	6.09E+06	8.46E+06	5.80E+06	1.46	0.54	0.00907494
P18077	60S ribosomal protein L35a OS=Homo sapiens OX=9606 GN=RPL35A PE=1 SV=2	22	73	3	1.62E+07	1.57E+07	1.60E+07	1.64E+07	1.83E+07	1.59E+07	1.46E+07	1.51E+07	1.59E+07	1.43E+07	1.35E+07	1.14E+07	1.64E+07	1.41E+07	1.16	0.21	0.015389441
Q99798	Aconitate hydratase, mitochondrial OS=Homo sapiens OX=9606 GN=ACO2 PE=1 SV=2	4	14	3	1.55E+06	1.89E+06	2.20E+06	1.45E+06	1.65E+06	2.29E+06	1.48E+06	1.54E+06	2.02E+06	1.62E+06	1.89E+06	1.19E+06	1.84E+06	1.62E+06	1.13	0.18	0.274144395
P07738	Bisphosphoglycerate mutase OS=Homo sapiens OX=9606 GN=BPGM PE=1 SV=2	12	30	3	4.99E+06	5.04E+06	5.21E+06	4.67E+06	5.19E+06	5.16E+06	6.92E+06	6.11E+06	6.09E+06	5.80E+06	5.91E+06	5.98E+06	5.04E+06	6.14E+06	0.82	-0.28	0.000489287
O60218	Aldo-keto reductase family 1 member B10 OS=Homo sapiens OX=9606 GN=AKR1B10 PE=1 SV=2	5	47	3	5.05E+07	5.07E+07	4.66E+07	5.32E+07	5.42E+07	3.95E+07	6.54E+07	6.43E+07	7.26E+07	7.95E+07	5.64E+07	5.72E+07	4.91E+07	6.59E+07	0.74	-0.42	0.004037189
Q9UNS2	COP9 signalosome complex subunit 3 OS=Homo sapiens OX=9606 GN=COPS3 PE=1 SV=3	10	9	3		3.81E+06	3.27E+06	9.85E+05	1.75E+06	3.46E+06	3.46E+06	2.78E+06	3.34E+06	3.87E+06	8.15E+05	4.12E+06	2.66E+06	3.07E+06	0.87	-0.21	0.589485633
P46063	ATP-dependent DNA helicase Q1 OS=Homo sapiens OX=9606 GN=RECQL PE=1 SV=3	5	26	3	2.27E+07	2.61E+07	2.46E+07	2.72E+07	2.37E+07	2.32E+07	2.39E+07	2.68E+07	2.48E+07	2.77E+07	2.39E+07	2.26E+07	2.46E+07	2.49E+07	0.99	-0.02	0.75712383
P62899	60S ribosomal protein L31 OS=Homo sapiens OX=9606 GN=RPL31 PE=1 SV=1	33	8	3	1.63E+06	5.84E+06	4.79E+06	5.55E+06	5.95E+06	5.41E+06	4.36E+06	4.54E+06	4.33E+06	5.21E+06	3.90E+06	3.01E+06	4.86E+06	4.22E+06	1.15	0.20	0.411989117
P00374	Dihydrofolate reductase OS=Homo sapiens OX=9606 GN=DHFR PE=1 SV=2	26	32	3	1.38E+07	1.53E+07	1.49E+07	1.19E+07	1.49E+07	1.45E+07	1.52E+07	1.54E+07	1.53E+07	1.68E+07	1.43E+07	1.11E+07	1.42E+07	1.47E+07	0.97	-0.05	0.638692333
P04003	C4b-binding protein alpha chain OS=Homo sapiens OX=9606 GN=C4BPA PE=1 SV=2	5	42	3	8.99E+06	1.13E+07	4.05E+07	2.42E+07	1.91E+07	2.33E+07	1.37E+07	7.87E+06	1.03E+07	1.05E+07	1.08E+07	1.89E+07	2.12E+07	1.20E+07	1.77	0.82	0.106291964
Q13144	Translation initiation factor eIF-2B subunit epsilon OS=Homo sapiens OX=9606 GN=EIF2B5 PE=1 SV=3	5	5	3	3.86E+06	1.36E+06		2.81E+06	2.85E+06	4.81E+06	4.70E+06		6.72E+05	9.80E+05	3.30E+06	3.78E+06	3.14E+06	2.69E+06	1.17	0.22	0.659111765
Q9UDY2	Tight junction protein ZO-2 OS=Homo sapiens OX=9606 GN=TJP2 PE=1 SV=2	2	15	3	2.39E+06	4.13E+06	4.34E+06	4.84E+06	5.06E+06	3.55E+06	3.92E+06	1.84E+06	4.41E+06	2.37E+06	3.85E+06	2.84E+06	4.05E+06	3.21E+06	1.26	0.34	0.170537399
P54727	UV excision repair protein RAD23 homolog B OS=Homo sapiens OX=9606 GN=RAD23B PE=1 SV=1	7	18	3	9.79E+06	1.15E+07	1.33E+07	1.52E+07	1.39E+07	9.91E+06	1.32E+07	1.32E+07	1.19E+07	1.38E+07	1.28E+07	1.17E+07	1.23E+07	1.28E+07	0.96	-0.06	0.606198214
P29144	Tripeptidyl-peptidase 2 OS=Homo sapiens OX=9606 GN=TPP2 PE=1 SV=4	2	6	3	3.27E+06	1.50E+06	3.85E+06	3.66E+06	3.30E+06	3.69E+06	2.75E+06	3.52E+05	3.78E+06	2.91E+06	4.28E+05	1.95E+06	3.21E+06	2.03E+06	1.58	0.66	0.11428255
P15151	Poliovirus receptor OS=Homo sapiens OX=9606 GN=PVR PE=1 SV=2	6	10	3	5.23E+06	5.87E+06	6.04E+06	6.52E+06	6.14E+06	5.69E+06	3.50E+06	4.12E+06	2.72E+06	2.79E+06	2.35E+06	2.70E+06	5.91E+06	3.03E+06	1.95	0.96	1.07461E-05
Q13509	Tubulin beta-3 chain OS=Homo sapiens OX=9606 GN=TUBB3 PE=1 SV=2	47	3130	4	3.59E+06	3.48E+06	2.09E+06	2.39E+06	1.48E+06	2.46E+06	3.29E+06	2.25E+06	1.45E+06	1.38E+06	1.06E+06	2.48E+06	2.58E+06	1.98E+06	1.30	0.38	0.2376746
Q5QNW6	Histone H2B type 2-F OS=Homo sapiens OX=9606 GN=HIST2H2BF PE=1 SV=3	56	6410	4	7.85E+09	6.59E+09	9.43E+09	7.36E+09	7.96E+09	7.69E+09	8.01E+09	7.00E+09	8.29E+09	7.38E+09	7.73E+09	7.48E+09	7.81E+09	7.65E+09	1.02	0.03	0.708002684
P13929	Beta-enolase OS=Homo sapiens OX=9606 GN=ENO3 PE=1 SV=5	31	397	4	1.55E+07	1.45E+07	2.07E+07	2.24E+07	2.13E+07	1.90E+07	2.69E+07	2.14E+07	2.25E+07	1.85E+07	2.29E+07	2.26E+07	1.89E+07	2.25E+07	0.84	-0.25	0.067197207
Q05639	Elongation factor 1-alpha 2 OS=Homo sapiens OX=9606 GN=EEF1A2 PE=1 SV=1	42	693	4	6.80E+06	5.47E+06	8.77E+06	5.51E+06	7.23E+06	5.20E+06	8.79E+06	5.14E+06	3.74E+06	3.13E+06	6.12E+06	3.98E+06	6.50E+06	5.15E+06	1.26	0.34	0.219976107
P04899	Guanine nucleotide-binding protein G(i) subunit alpha-2 OS=Homo sapiens OX=9606 GN=GNAI2 PE=1 SV=3	30	148	4	5.93E+07	6.23E+07	7.74E+07	6.77E+07	8.36E+07	5.95E+07	6.07E+07	5.52E+07	6.86E+07	6.24E+07	7.33E+07	6.46E+07	6.83E+07	6.41E+07	1.07	0.09	0.415255847
P31943	Heterogeneous nuclear ribonucleoprotein H OS=Homo sapiens OX=9606 GN=HNRNPH1 PE=1 SV=4	20	150	4	2.52E+07	2.34E+07	1.93E+07	2.04E+07	1.99E+07	2.16E+07	1.28E+07	1.12E+07	1.03E+07	9.23E+06	8.21E+06	9.97E+06	2.17E+07	1.03E+07	2.11	1.08	3.79739E-06
P02774	Vitamin D-binding protein OS=Homo sapiens OX=9606 GN=GC PE=1 SV=2	9	94	4	3.51E+07	2.37E+07	2.12E+07	2.90E+07	2.67E+07	3.18E+07	4.14E+07	3.57E+07	3.07E+07	3.41E+07	3.08E+07	2.93E+07	2.79E+07	3.37E+07	0.83	-0.27	0.0658469
P35241	Radixin OS=Homo sapiens OX=9606 GN=RDX PE=1 SV=1	19	221	4	4.70E+06	1.92E+06			1.21E+06	9.43E+05	1.39E+06	9.99E+05		7.45E+05	8.19E+05	1.32E+06	2.19E+06	1.06E+06	2.08	1.06	0.278437355
P62314	Small nuclear ribonucleoprotein Sm D1 OS=Homo sapiens OX=9606 GN=SNRPD1 PE=1 SV=1	55	183	4	5.98E+07	6.77E+07	7.79E+07	6.34E+07	7.18E+07	8.12E+07	4.09E+07	5.27E+07	5.14E+07	4.71E+07	5.61E+07	3.49E+07	7.03E+07	4.72E+07	1.49	0.57	0.000601081
P60033	CD81 antigen OS=Homo sapiens OX=9606 GN=CD81 PE=1 SV=1	28	74	4	3.14E+07	2.77E+07	1.89E+07	2.33E+07	2.74E+07	2.85E+07	2.30E+07	1.88E+07	1.30E+07	1.59E+07	1.70E+07	1.72E+07	2.62E+07	1.75E+07	1.50	0.58	0.003674527
Q00266	S-adenosylmethionine synthase isoform type-1 OS=Homo sapiens OX=9606 GN=MAT1A PE=1 SV=2	19	144	4	3.53E+07	3.40E+07	3.62E+07	3.55E+07	3.22E+07	2.47E+07	4.37E+07	4.43E+07	3.01E+07	4.05E+07	2.92E+07	3.67E+07	3.30E+07	3.74E+07	0.88	-0.18	0.201708997
P51148	Ras-related protein Rab-5C OS=Homo sapiens OX=9606 GN=RAB5C PE=1 SV=2	37	124	4	3.82E+07	4.11E+07	3.34E+07	3.22E+07	4.14E+07	3.60E+07	3.01E+07	2.99E+07	2.58E+07	2.63E+07	2.66E+07	2.19E+07	3.70E+07	2.68E+07	1.38	0.47	0.000536151
Q16576	Histone-binding protein RBBP7 OS=Homo sapiens OX=9606 GN=RBBP7 PE=1 SV=1	28	68	4	1.66E+07	1.78E+07	1.29E+07	1.50E+07	1.30E+07	1.56E+07	1.12E+07	1.31E+07	7.86E+06	9.68E+06	9.00E+06	8.76E+06	1.52E+07	9.93E+06	1.53	0.61	0.000839949
Q15436	Protein transport protein Sec23A OS=Homo sapiens OX=9606 GN=SEC23A PE=1 SV=2	12	66	4	2.59E+07	2.99E+07	2.97E+07	3.08E+07	2.46E+07	2.60E+07	2.29E+07	1.65E+07	1.39E+07	1.38E+07	1.87E+07	1.66E+07	2.78E+07	1.70E+07	1.63	0.71	0.00014234
Q15233	Non-POU domain-containing octamer-binding protein OS=Homo sapiens OX=9606 GN=NONO PE=1 SV=4	12	107	4	1.83E+07	1.90E+07	2.26E+07	2.61E+07	2.08E+07	1.80E+07	1.17E+07	1.33E+07	1.25E+07	1.34E+07	1.31E+07	8.48E+06	2.08E+07	1.21E+07	1.72	0.78	0.000329338
O15144	Actin-related protein 2/3 complex subunit 2 OS=Homo sapiens OX=9606 GN=ARPC2 PE=1 SV=1	23	70	4	1.19E+07	1.13E+07	1.53E+07	1.21E+07	1.54E+07	1.20E+07	1.08E+07	1.22E+07	1.38E+07	6.46E+06	1.40E+07	1.03E+07	1.30E+07	1.13E+07	1.15	0.21	0.235976115
P61769	Beta-2-microglobulin OS=Homo sapiens OX=9606 GN=B2M PE=1 SV=1	38	122	4	8.57E+07	8.34E+07	8.73E+07	7.20E+07	6.87E+07	9.52E+07	4.37E+07	4.30E+07	2.96E+07	3.39E+07	3.59E+07	2.66E+07	8.20E+07	3.55E+07	2.31	1.21	6.24067E-06
P51991	Heterogeneous nuclear ribonucleoprotein A3 OS=Homo sapiens OX=9606 GN=HNRNPA3 PE=1 SV=2	22	53	4	1.06E+07	1.42E+07	5.58E+06	1.18E+07	8.52E+06	9.57E+06	3.34E+06	3.31E+06	4.09E+06	5.76E+06	1.42E+06	1.59E+06	1.00E+07	3.25E+06	3.09	1.63	0.001201704
P61106	Ras-related protein Rab-14 OS=Homo sapiens OX=9606 GN=RAB14 PE=1 SV=4	33	50	4	2.16E+07	1.61E+07	1.87E+07	1.71E+07	1.90E+07	1.78E+07	1.19E+07	1.11E+07	1.59E+07	1.58E+07	1.27E+07	9.22E+06	1.84E+07	1.28E+07	1.44	0.53	0.002222368
O15143	Actin-related protein 2/3 complex subunit 1B OS=Homo sapiens OX=9606 GN=ARPC1B PE=1 SV=3	19	92	4	1.58E+07	2.03E+07	1.94E+07	1.67E+07	1.58E+07	1.83E+07	1.51E+07	1.78E+07	1.57E+07	1.13E+07	1.16E+07	1.44E+07	1.77E+07	1.43E+07	1.24	0.31	0.025025205
P06744	Glucose-6-phosphate isomerase OS=Homo sapiens OX=9606 GN=GPI PE=1 SV=4	12	64	4	1.02E+07	6.92E+06	1.14E+07	1.81E+07	1.21E+07	8.26E+06	1.35E+07	1.11E+07	1.46E+07	1.01E+07	1.23E+07	1.27E+07	1.12E+07	1.24E+07	0.90	-0.15	0.508463254
P50914	60S ribosomal protein L14 OS=Homo sapiens OX=9606 GN=RPL14 PE=1 SV=4	22	166	4	6.57E+07	8.24E+07	7.31E+07	7.94E+07	7.33E+07	6.91E+07	4.28E+07	5.18E+07	4.04E+07	4.62E+07	3.82E+07	3.66E+07	7.38E+07	4.27E+07	1.73	0.79	4.06751E-06
Q99829	Copine-1 OS=Homo sapiens OX=9606 GN=CPNE1 PE=1 SV=1	10	62	4	1.28E+07	8.10E+06	1.00E+07	5.82E+06	6.55E+06	8.61E+06	7.79E+06	6.38E+06	4.78E+06	4.20E+06	6.42E+06	5.05E+06	8.64E+06	5.77E+06	1.50	0.58	0.039803457
P05546	Heparin cofactor 2 OS=Homo sapiens OX=9606 GN=SERPIND1 PE=1 SV=3	7	187	4	6.27E+07	7.02E+07	5.85E+07	7.13E+07	7.12E+07	6.78E+07	7.65E+07	7.93E+07	8.66E+07	8.90E+07	8.80E+07	8.06E+07	6.69E+07	8.33E+07	0.80	-0.32	0.000284045
Q04917	14-3-3 protein eta OS=Homo sapiens OX=9606 GN=YWHAH PE=1 SV=4	24	61	4	1.17E+07	1.25E+07	6.08E+06	8.65E+06	8.37E+06	7.92E+06	1.14E+07	9.22E+06	8.40E+06	1.05E+07	5.59E+06	6.22E+06	9.20E+06	8.55E+06	1.08	0.11	0.644696961
P12268	Inosine-5'-monophosphate dehydrogenase 2 OS=Homo sapiens OX=9606 GN=IMPDH2 PE=1 SV=2	14	70	4	1.79E+07	1.85E+07	1.46E+07	1.71E+07	1.61E+07	1.52E+07	1.28E+07	1.46E+07	1.05E+07	1.20E+07	1.07E+07	8.56E+06	1.66E+07	1.15E+07	1.44	0.52	0.000948165
Q05682	Caldesmon OS=Homo sapiens OX=9606 GN=CALD1 PE=1 SV=3	8	22	4	3.14E+06	2.45E+06	4.74E+06	4.89E+06	3.72E+06	2.84E+06	1.51E+06	1.70E+06	3.04E+06	1.12E+06	1.50E+06	1.02E+06	3.63E+06	1.65E+06	2.20	1.14	0.003523827
P35221	Catenin alpha-1 OS=Homo sapiens OX=9606 GN=CTNNA1 PE=1 SV=1	8	24	4	1.73E+07	1.91E+07	1.82E+07	1.91E+07	1.79E+07	1.73E+07	1.94E+07	2.04E+07	1.87E+07	2.31E+07	2.02E+07	1.83E+07	1.81E+07	2.00E+07	0.91	-0.14	0.04429624
P40261	Nicotinamide N-methyltransferase OS=Homo sapiens OX=9606 GN=NNMT PE=1 SV=1	20	75	4	2.53E+07	2.90E+07	2.90E+07	2.78E+07	2.63E+07	2.82E+07	1.56E+07	1.46E+07	1.55E+07	1.51E+07	1.91E+07	1.33E+07	2.76E+07	1.55E+07	1.78	0.83	4.99702E-07
P49720	Proteasome subunit beta type-3 OS=Homo sapiens OX=9606 GN=PSMB3 PE=1 SV=2	29	50	4	1.07E+07	1.23E+07	9.89E+06	7.73E+06	8.75E+06	9.77E+06	1.07E+07	1.13E+07	9.39E+06	7.30E+06	1.08E+07	1.05E+07	9.87E+06	1.00E+07	0.99	-0.02	0.878413799
Q5JWF2	Guanine nucleotide-binding protein G(s) subunit alpha isoforms XLas OS=Homo sapiens OX=9606 GN=GNAS PE=1 SV=2	6	69	4	9.22E+06	1.06E+07	8.95E+06	8.88E+06	8.47E+06	9.13E+06	8.37E+06	7.69E+06	6.63E+06	6.84E+06	6.43E+06	6.78E+06	9.21E+06	7.12E+06	1.29	0.37	0.000638556
P20339	Ras-related protein Rab-5A OS=Homo sapiens OX=9606 GN=RAB5A PE=1 SV=2	37	87	4	7.82E+06	8.49E+06	8.06E+06	8.14E+06	7.48E+06	8.52E+06	7.36E+06	6.87E+06	7.79E+06	5.80E+06	6.49E+06	6.94E+06	8.09E+06	6.87E+06	1.18	0.23	0.005963896
O14974	Protein phosphatase 1 regulatory subunit 12A OS=Homo sapiens OX=9606 GN=PPP1R12A PE=1 SV=1	4	20	4	7.81E+06	3.32E+06	5.41E+06	3.06E+06	2.63E+06	1.84E+06	5.00E+06	1.72E+06	9.14E+05	3.86E+06	7.49E+05	2.46E+06	4.01E+06	2.45E+06	1.64	0.71	0.200776314
P02747	Complement C1q subcomponent subunit C OS=Homo sapiens OX=9606 GN=C1QC PE=1 SV=3	21	36	4	1.20E+06			5.59E+05	8.09E+05	4.88E+05	1.88E+07	3.58E+06	3.10E+06	1.49E+06	2.53E+06	2.00E+08	7.64E+05	3.82E+07	0.02	-5.64	0.300396701
Q96A65	Exocyst complex component 4 OS=Homo sapiens OX=9606 GN=EXOC4 PE=1 SV=1	7	14	4	3.90E+06	2.48E+06	2.69E+06	3.31E+06	3.06E+06	2.36E+06	1.73E+06	5.05E+05	3.82E+05	7.55E+05	3.17E+05	6.99E+05	2.97E+06	7.32E+05	4.06	2.02	3.84562E-05
P00751	Complement factor B OS=Homo sapiens OX=9606 GN=CFB PE=1 SV=2	9	84	4	1.61E+07	1.75E+07	1.46E+07	1.38E+07	1.40E+07	1.56E+07	1.82E+07	2.32E+07	2.41E+07	1.59E+07	2.44E+07	1.76E+07	1.53E+07	2.06E+07	0.74	-0.43	0.015932008
Q96QD8	Sodium-coupled neutral amino acid transporter 2 OS=Homo sapiens OX=9606 GN=SLC38A2 PE=1 SV=2	12	42	4	1.44E+07	1.74E+07	1.34E+07	1.21E+07	1.25E+07	1.45E+07	8.73E+06	7.75E+06	6.00E+06	6.47E+06	4.88E+06	6.23E+06	1.41E+07	6.67E+06	2.11	1.07	2.88716E-05
P15153	Ras-related C3 botulinum toxin substrate 2 OS=Homo sapiens OX=9606 GN=RAC2 PE=1 SV=1	35	116	4	5.76E+07	5.51E+07	5.48E+07	6.74E+07	5.78E+07	5.91E+07	5.93E+07	5.96E+07	5.96E+07	7.03E+07	5.59E+07	5.52E+07	5.86E+07	6.00E+07	0.98	-0.03	0.660346795
P50552	Vasodilator-stimulated phosphoprotein OS=Homo sapiens OX=9606 GN=VASP PE=1 SV=3	14	116	4	2.16E+07	2.12E+07	1.62E+07	1.55E+07	1.60E+07	1.82E+07	1.83E+07	1.77E+07	1.38E+07	1.78E+07	1.34E+07	1.67E+07	1.81E+07	1.63E+07	1.11	0.15	0.223982082
P04424	Argininosuccinate lyase OS=Homo sapiens OX=9606 GN=ASL PE=1 SV=4	11	52	4	1.16E+07	9.85E+06	1.17E+07	9.74E+06	1.01E+07	1.10E+07	1.82E+07	1.58E+07	1.62E+07	1.24E+07	1.43E+07	1.40E+07	1.07E+07	1.51E+07	0.70	-0.51	0.001821252
P12110	Collagen alpha-2(VI) chain OS=Homo sapiens OX=9606 GN=COL6A2 PE=1 SV=4	5	88	4	1.06E+07	1.65E+07	9.88E+06	9.65E+06	1.20E+07	1.29E+07	2.75E+07	2.68E+07	1.86E+07	1.50E+07	2.42E+07	1.33E+07	1.19E+07	2.09E+07	0.57	-0.81	0.013728395
P05026	Sodium/potassium-transporting ATPase subunit beta-1 OS=Homo sapiens OX=9606 GN=ATP1B1 PE=1 SV=1	21	39	4	8.26E+06	6.25E+06	1.06E+07	8.44E+06	8.98E+06	7.77E+06	6.14E+06	4.68E+06	4.16E+06	4.03E+06	2.38E+06	3.82E+06	8.38E+06	4.20E+06	2.00	1.00	0.000305952
Q9Y5L0	Transportin-3 OS=Homo sapiens OX=9606 GN=TNPO3 PE=1 SV=3	8	16	4	2.00E+06	2.10E+06	2.59E+06	8.89E+05	2.86E+06	2.66E+06	4.95E+05		1.49E+06	6.06E+05	6.88E+05	3.36E+05	2.18E+06	7.22E+05	3.02	1.60	0.002960487
O15162	Phospholipid scramblase 1 OS=Homo sapiens OX=9606 GN=PLSCR1 PE=1 SV=1	12	73	4	3.81E+07	3.25E+07	2.88E+07	2.88E+07	2.37E+07	3.09E+07	2.13E+07	1.74E+07	2.00E+07	1.29E+07	2.29E+07	1.58E+07	3.05E+07	1.84E+07	1.66	0.73	0.00074725
P51114	Fragile X mental retardation syndrome-related protein 1 OS=Homo sapiens OX=9606 GN=FXR1 PE=1 SV=3	11	78	4	1.36E+07	1.65E+07	1.55E+07	1.77E+07	1.65E+07	1.44E+07	7.18E+06	7.78E+06	7.57E+06	7.94E+06	7.71E+06	6.41E+06	1.57E+07	7.43E+06	2.11	1.08	1.06546E-05
P62854	40S ribosomal protein S26 OS=Homo sapiens OX=9606 GN=RPS26 PE=1 SV=3	44	123	4	5.79E+07	5.22E+07	4.75E+07	5.47E+07	5.00E+07	4.84E+07	3.36E+07	3.61E+07	2.95E+07	3.09E+07	3.01E+07	2.54E+07	5.18E+07	3.09E+07	1.67	0.74	2.71958E-06
P67870	Casein kinase II subunit beta OS=Homo sapiens OX=9606 GN=CSNK2B PE=1 SV=1	23	67	4	2.70E+07	3.08E+07	2.76E+07	3.10E+07	3.02E+07	2.74E+07	1.73E+07	1.80E+07	1.96E+07	1.83E+07	1.64E+07	1.37E+07	2.90E+07	1.72E+07	1.69	0.75	1.10671E-06
P48740	Mannan-binding lectin serine protease 1 OS=Homo sapiens OX=9606 GN=MASP1 PE=1 SV=3	9	102	4	3.53E+07	3.67E+07	3.73E+07	4.34E+07	3.84E+07	4.64E+07	4.52E+07	4.49E+07	4.72E+07	4.49E+07	4.97E+07	3.90E+07	3.96E+07	4.51E+07	0.88	-0.19	0.037053961

Supplementary Table 4. List of all proteins identified by label free proteomics analysis.

Accession	Description	Coverage [%]	# PSMs	# Unique Peptides	Amitriptyline 1	Amitriptyline 2	Amitriptyline 3	Amitriptyline 4	Amitriptyline 5	Amitriptyline 6	Vehicle 1	Vehicle 2	Vehicle 3	Vehicle 4	Vehicle 5	Vehicle 6	Average amitriptyline	Average vehicle	Ratio amitriptyline/ vehicle	Log 2 fold change (amitriptyline /vehicle)	P-value
Q9BXJ4	Complement C1q tumor necrosis factor-related protein 3 OS=Homo sapiens OX=9606 GN=C1QTNF3 PE=1 SV=1	13	74	4	2.71E+07	2.37E+07	2.01E+07	2.30E+07	2.01E+07	1.75E+07	3.27E+07	2.61E+07	2.44E+07	2.18E+07	1.86E+07	1.76E+07	2.19E+07	2.35E+07	0.93	-0.10	0.557221439
Q92542	Nicastrin OS=Homo sapiens OX=9606 GN=NCSTN PE=1 SV=2	10	10	4	6.13E+06	4.68E+06	4.07E+06	4.39E+06	5.85E+06	5.45E+06	1.24E+06	1.39E+06	1.33E+06	2.15E+06	1.36E+06	1.84E+06	5.09E+06	1.55E+06	3.28	1.72	3.50612E-05
P00738	Haptoglobin OS=Homo sapiens OX=9606 GN=HP PE=1 SV=1	10	91	4	3.01E+07	2.98E+07	2.57E+07	2.76E+07	2.37E+07	1.92E+07	3.19E+07	2.94E+07	2.56E+07	3.06E+07	2.07E+07	1.82E+07	2.60E+07	2.61E+07	1.00	0.00	0.989369582
Q13409	Cytoplasmic dynein 1 intermediate chain 2 OS=Homo sapiens OX=9606 GN=DYNC1I2 PE=1 SV=3	12	12	4	3.06E+06	5.11E+06	3.45E+06	2.03E+06	2.03E+06	4.08E+06		1.41E+06	1.86E+06	1.60E+06	3.79E+06	2.48E+06	3.29E+06	2.23E+06	1.48	0.56	0.136684388
P16070	CD44 antigen OS=Homo sapiens OX=9606 GN=CD44 PE=1 SV=3	6	142	4	4.09E+07	4.02E+07	3.89E+07	4.50E+07	4.18E+07	4.64E+07	2.12E+07	2.21E+07	2.06E+07	2.05E+07	1.83E+07	1.65E+07	4.22E+07	1.99E+07	2.12	1.09	8.68511E-08
O96019	Actin-like protein 6A OS=Homo sapiens OX=9606 GN=ACTL6A PE=1 SV=1	16	54	4	1.66E+07	1.95E+07	1.16E+07	8.42E+06	1.24E+07	1.45E+07	1.12E+07	1.43E+07	9.68E+06	8.90E+06	9.96E+06	9.43E+06	1.38E+07	1.06E+07	1.31	0.39	0.109866641
P26022	Pentraxin-related protein PTX3 OS=Homo sapiens OX=9606 GN=PTX3 PE=1 SV=3	14	28	4	1.67E+06	1.19E+06	4.85E+05	1.35E+06	1.65E+06	3.47E+06	9.31E+06	1.07E+07	5.79E+06	7.26E+06	7.28E+06	7.29E+06	1.64E+06	7.94E+06	0.21	-2.28	6.25913E-05
Q99805	Transmembrane 9 superfamily member 2 OS=Homo sapiens OX=9606 GN=TM9SF2 PE=1 SV=1	9	48	4	8.56E+06	7.88E+06	8.28E+06	8.04E+06	7.52E+06	8.58E+06	6.05E+06	6.04E+06	4.48E+06	5.49E+06	5.60E+06	4.17E+06	8.14E+06	5.30E+06	1.54	0.62	7.88823E-05
Q00577	Transcriptional activator protein Pur-alpha OS=Homo sapiens OX=9606 GN=PURA PE=1 SV=2	25	22	4	1.05E+07	1.09E+07	5.85E+06	5.95E+06	6.16E+06	7.87E+06	9.49E+06	8.84E+06	5.97E+06	9.55E+06	6.46E+06	5.28E+06	7.87E+06	7.60E+06	1.04	0.05	0.829657978
Q92522	Histone H1x OS=Homo sapiens OX=9606 GN=H1FX PE=1 SV=1	16	114	4	5.32E+07	5.80E+07	5.83E+07	4.88E+07	4.63E+07	4.95E+07	4.97E+07	5.09E+07	4.75E+07	4.23E+07	4.02E+07	3.82E+07	5.23E+07	4.48E+07	1.17	0.22	0.029697859
Q13085	Acetyl-CoA carboxylase 1 OS=Homo sapiens OX=9606 GN=ACACA PE=1 SV=2	3	23	4	4.93E+06	5.51E+06	6.90E+06	5.10E+06	6.78E+06	4.51E+06	5.26E+06	5.87E+06	5.36E+06	6.02E+06	6.61E+06	5.10E+06	5.62E+06	5.70E+06	0.99	-0.02	0.867454914
P61289	Proteasome activator complex subunit 3 OS=Homo sapiens OX=9606 GN=PSME3 PE=1 SV=1	17	73	4	1.15E+07	1.43E+07	9.00E+06	1.23E+07	1.16E+07	9.75E+06	9.43E+06	8.79E+06	8.27E+06	6.82E+06	5.64E+06	5.68E+06	1.14E+07	7.44E+06	1.53	0.62	0.003058654
P04632	Calpain small subunit 1 OS=Homo sapiens OX=9606 GN=CAPNS1 PE=1 SV=1	20	32	4	8.09E+06	6.43E+06	7.89E+06	7.54E+06	7.83E+06	7.12E+06	5.44E+06	4.11E+06	4.42E+06	5.24E+06	5.50E+06	4.33E+06	7.48E+06	4.84E+06	1.55	0.63	2.26971E-05
Q7Z2W4	Zinc finger CCCH-type antiviral protein 1 OS=Homo sapiens OX=9606 GN=ZC3HAV1 PE=1 SV=3	6	42	4	7.69E+06	8.72E+06	7.96E+06	9.03E+06	7.56E+06	8.36E+06	3.36E+06	3.87E+06	4.57E+06	4.65E+06	4.58E+06	4.44E+06	8.22E+06	4.24E+06	1.94	0.95	2.32232E-07
Q8NE71	ATP-binding cassette sub-family F member 1 OS=Homo sapiens OX=9606 GN=ABCF1 PE=1 SV=2	8	20	4	4.76E+06	3.93E+06	5.03E+06	6.96E+06	5.55E+06	5.82E+06	3.85E+06	1.87E+06	3.64E+06	3.15E+06	4.97E+05	1.70E+06	5.34E+06	2.45E+06	2.18	1.12	0.001916005
P62888	60S ribosomal protein L30 OS=Homo sapiens OX=9606 GN=RPL30 PE=1 SV=2	51	62	4	1.83E+07	1.70E+07	1.40E+07	1.46E+07	1.48E+07	1.72E+07	1.37E+07	1.35E+07	8.98E+06	1.18E+07	1.13E+07	9.87E+06	1.60E+07	1.15E+07	1.39	0.47	0.001729187
P40763	Signal transducer and activator of transcription 3 OS=Homo sapiens OX=9606 GN=STAT3 PE=1 SV=2	9	34	4	4.89E+06	6.78E+06	5.04E+06	5.30E+06	5.42E+06	4.39E+06	5.88E+06	7.17E+06	5.89E+06	6.80E+06	5.99E+06	5.98E+06	5.30E+06	6.28E+06	0.84	-0.24	0.037770284
Q96FW1	Ubiquitin thioesterase OTUB1 OS=Homo sapiens OX=9606 GN=OTUB1 PE=1 SV=2	19	26	4	7.77E+06	7.05E+06	8.09E+06	1.01E+07	7.81E+06	7.74E+06	8.51E+06	8.36E+06	8.38E+06	7.84E+06	7.69E+06	7.06E+06	8.10E+06	7.97E+06	1.02	0.02	0.802514473
Q12905	Interleukin enhancer-binding factor 2 OS=Homo sapiens OX=9606 GN=ILF2 PE=1 SV=2	15	42	4	9.74E+06	9.56E+06	1.30E+07	1.07E+07	7.77E+06	8.11E+06	1.26E+07	9.84E+06	9.95E+06	5.79E+06	1.09E+07	8.63E+06	9.81E+06	9.62E+06	1.02	0.03	0.874965993
O75340	Programmed cell death protein 6 OS=Homo sapiens OX=9606 GN=PDCD6 PE=1 SV=1	31	84	4	2.20E+07	2.25E+07	2.36E+07	1.97E+07	1.96E+07	1.77E+07	1.48E+07	1.55E+07	1.21E+07	1.40E+07	1.22E+07	2.05E+07	2.08E+07	1.48E+07	1.40	0.49	0.003738271
O95834	Echinoderm microtubule-associated protein-like 2 OS=Homo sapiens OX=9606 GN=EML2 PE=1 SV=1	9	36	4	7.87E+06	8.61E+06	7.93E+06	6.34E+06	5.89E+06	6.77E+06	9.98E+06	1.04E+07	7.77E+06	7.49E+06	7.60E+06	7.94E+06	7.24E+06	8.53E+06	0.85	-0.24	0.090156155
P62913	60S ribosomal protein L11 OS=Homo sapiens OX=9606 GN=RPL11 PE=1 SV=2	31	82	4	1.72E+07	1.78E+07	1.51E+07	1.35E+07	1.66E+07	1.33E+07	1.57E+07	1.50E+07	1.37E+07	1.35E+07	1.75E+07	1.87E+07	1.56E+07	1.57E+07	0.99	-0.01	0.912240929
Q9Y5P6	Mannose-1-phosphate guanyltransferase beta OS=Homo sapiens OX=9606 GN=GMPPB PE=1 SV=2	17	30	4	3.88E+06	4.75E+06	4.91E+06	3.60E+06	3.26E+06	3.76E+06	5.02E+06	5.78E+06	4.11E+06	6.92E+06	4.21E+06	3.37E+06	4.03E+06	4.90E+06	0.82	-0.28	0.179544866
Q9NR31	GTP-binding protein SAR1a OS=Homo sapiens OX=9606 GN=SAR1A PE=1 SV=1	31	64	4	1.40E+07	1.02E+07	9.49E+06	8.31E+06	9.59E+06	1.14E+07	9.35E+06	8.75E+06	6.72E+06	5.95E+06	9.36E+06	8.26E+06	1.05E+07	8.06E+06	1.30	0.38	0.038075372
O14950	Myosin regulatory light chain 12B OS=Homo sapiens OX=9606 GN=MYL12B PE=1 SV=2	29	65	4	2.18E+07	2.16E+07	2.18E+07	2.51E+07	2.14E+07	2.17E+07	2.15E+07	2.20E+07	2.21E+07	1.94E+07	1.84E+07	1.67E+07	2.22E+07	2.00E+07	1.11	0.15	0.068965072
P28072	Proteasome subunit beta type-6 OS=Homo sapiens OX=9606 GN=PSMB6 PE=1 SV=4	29	76	4	2.32E+07	1.50E+07	1.33E+07	1.20E+07	1.37E+07	1.75E+07	2.40E+07	2.29E+07	2.46E+07	2.00E+07	1.76E+07	1.29E+07	1.58E+07	2.04E+07	0.77	-0.37	0.093320352
Q07666	KH domain-containing, RNA-binding, signal transduction-associated protein 1 OS=Homo sapiens OX=9606 GN=KHDRBS1 PE=1 SV=1	12	30	4	1.23E+07	1.23E+07	1.18E+07	1.27E+07	1.02E+07	1.16E+07	8.84E+06	8.75E+06	8.21E+06	8.68E+06	7.06E+06	7.06E+06	1.18E+07	8.10E+06	1.46	0.54	2.24987E-05
P10809	60 kDa heat shock protein, mitochondrial OS=Homo sapiens OX=9606 GN=HSPD1 PE=1 SV=2	11	45	4	5.21E+06	1.06E+07	5.19E+06	4.57E+06	4.18E+06	3.87E+06	9.21E+06	6.96E+06	7.72E+06	9.40E+06	6.51E+06	9.72E+06	5.61E+06	8.25E+06	0.68	-0.56	0.05428114
P02746	Complement C1q subcomponent subunit B OS=Homo sapiens OX=9606 GN=C1QB PE=1 SV=3	23	24	4	7.51E+05	9.34E+05	6.46E+05	6.95E+05	9.15E+05	9.43E+05	8.47E+06	1.79E+06	9.93E+05	1.73E+06	2.27E+06	4.98E+07	8.14E+05	1.08E+07	0.08	-3.73	0.258666568
Q13740	CD166 antigen OS=Homo sapiens OX=9606 GN=ALCAM PE=1 SV=2	13	16	4	3.15E+06	4.11E+06	2.82E+06	2.98E+06	2.82E+06	2.11E+06	1.52E+06	2.02E+06	1.02E+06	1.28E+06	1.16E+06	8.32E+05	3.00E+06	1.30E+06	2.30	1.20	0.000537756
Q9Y285	Phenylalanine--tRNA ligase alpha subunit OS=Homo sapiens OX=9606 GN=FARSA PE=1 SV=3	11	39	4	1.39E+07	1.68E+07	1.53E+07	1.57E+07	1.61E+07	1.53E+07	1.18E+07	1.20E+07	8.52E+06	1.25E+07	1.05E+07	1.06E+07	1.55E+07	1.10E+07	1.41	0.50	0.000155366
O15427	Monocarboxylate transporter 4 OS=Homo sapiens OX=9606 GN=SLC16A3 PE=1 SV=1	10	63	4	1.25E+07	1.30E+07	1.28E+07	1.58E+07	1.56E+07	1.05E+07	9.39E+06	7.77E+06	1.10E+07	1.04E+07	8.47E+06	5.79E+06	1.34E+07	8.81E+06	1.52	0.60	0.002350571
P22570	NADPH:adrenodoxin oxidoreductase, mitochondrial OS=Homo sapiens OX=9606 GN=FDXR PE=1 SV=3	12	50	4	1.21E+07	7.90E+06	1.19E+07	1.19E+07	1.21E+07	6.34E+06	1.07E+07	1.04E+07	1.08E+07	8.30E+06	8.77E+06	4.87E+06	1.04E+07	8.98E+06	1.16	0.21	0.339028786
P62244	40S ribosomal protein S15a OS=Homo sapiens OX=9606 GN=RPS15A PE=1 SV=2	31	146	4	5.17E+07	5.71E+07	7.44E+07	7.29E+07	7.60E+07	6.92E+07	4.27E+07	4.70E+07	5.73E+07	5.67E+07	5.65E+07	4.01E+07	6.69E+07	5.01E+07	1.34	0.42	0.009614808
Q14566	DNA replication licensing factor MCM6 OS=Homo sapiens OX=9606 GN=MCM6 PE=1 SV=1	6	20	4	3.66E+06	3.09E+06	3.45E+06	3.92E+06	3.34E+06	3.12E+06	9.27E+05	1.93E+06	9.75E+05		1.21E+06	2.12E+06	3.43E+06	1.43E+06	2.39	1.26	0.000356056
Q9HD45	Transmembrane 9 superfamily member 3 OS=Homo sapiens OX=9606 GN=TM9SF3 PE=1 SV=2	9	46	4	5.28E+06	5.08E+06	6.56E+06	5.20E+06	6.47E+06	7.45E+06	4.77E+06	3.69E+06	4.83E+06	6.96E+06	5.14E+06	3.90E+06	6.01E+06	4.88E+06	1.23	0.30	0.099303025
P63151	Serine/threonine-protein phosphatase 2A 55 kDa regulatory subunit B alpha isoform OS=Homo sapiens OX=9606 GN=PPP2R2A PE=1 SV=1	13	34	4	4.89E+06	4.59E+06	7.21E+06	7.90E+06	6.68E+06	8.53E+06	5.94E+06	4.81E+06	7.54E+06	6.18E+06	5.85E+06	4.81E+06	6.63E+06	5.86E+06	1.13	0.18	0.343088155
P29401	Transketolase OS=Homo sapiens OX=9606 GN=TKT PE=1 SV=3	9	52	4	5.87E+06	5.77E+06	7.08E+06	5.03E+06	5.63E+06	4.09E+06	6.79E+06	5.49E+06	6.14E+06	6.42E+06	6.29E+06	6.94E+05	5.58E+06	5.30E+06	1.05	0.07	0.796319818
Q15691	Microtubule-associated protein RP/EB family member 1 OS=Homo sapiens OX=9606 GN=MAPRE1 PE=1 SV=3	26	40	4	9.57E+06	8.08E+06	9.56E+06	9.00E+06	9.62E+06	9.56E+06	5.66E+06	5.38E+06	7.12E+06	5.99E+06	4.59E+06	4.12E+06	9.23E+06	5.48E+06	1.69	0.75	6.85236E-05
P50502	Hsc70-interacting protein OS=Homo sapiens OX=9606 GN=ST13 PE=1 SV=2	9	81	4	1.68E+07	2.02E+07	1.98E+07	2.09E+07	1.59E+07	1.61E+07	2.00E+07	1.97E+07	9.00E+06	2.11E+07	1.71E+07	1.94E+07	1.83E+07	1.77E+07	1.03	0.05	0.784924673
P00352	Retinal dehydrogenase 1 OS=Homo sapiens OX=9606 GN=ALDH1A1 PE=1 SV=2	9	24	4	1.10E+07	1.22E+07	1.25E+07	1.39E+07	1.16E+07	1.12E+07	1.85E+07	1.59E+07	1.91E+07	1.78E+07	1.63E+07	1.45E+07	1.21E+07	1.70E+07	0.71	-0.50	0.000290323
O15031	Plexin-B2 OS=Homo sapiens OX=9606 GN=PLXNB2 PE=1 SV=3	4	48	4	8.91E+06	7.33E+06	6.55E+06	8.93E+06	9.15E+06	7.05E+06	9.00E+06	1.05E+07	1.21E+07	1.14E+07	9.17E+06	9.01E+06	7.99E+06	1.02E+07	0.78	-0.35	0.012249348
P14678	Small nuclear ribonucleoprotein-associated proteins B and B' OS=Homo sapiens OX=9606 GN=SNRPB PE=1 SV=2	15	139	4	2.24E+07	2.32E+07	2.44E+07	2.19E+07	2.61E+07	2.50E+07	1.87E+07	1.65E+07	1.69E+07	1.75E+07	2.36E+07	1.87E+07	2.38E+07	1.86E+07	1.28	0.36	0.002776556
P18621	60S ribosomal protein L17 OS=Homo sapiens OX=9606 GN=RPL17 PE=1 SV=3	27	22	4	1.17E+07	1.70E+07	4.19E+06	9.24E+06	9.04E+06	1.48E+07	2.19E+06	1.57E+07	5.12E+06	4.71E+06	7.71E+06	7.75E+06	1.10E+07	7.20E+06	1.53	0.61	0.186243026
Q15113	Procollagen C-endopeptidase enhancer 1 OS=Homo sapiens OX=9606 GN=PCOLCE PE=1 SV=2	14	46	4	4.40E+06	4.91E+06	2.93E+06	3.58E+06	2.77E+06	6.22E+06	6.21E+06	6.35E+06	3.06E+06	4.11E+06	4.09E+06	7.38E+06	4.14E+06	5.20E+06	0.79	-0.33	0.249950727
P61604	10 kDa heat shock protein, mitochondrial OS=Homo sapiens OX=9606 GN=HSPE1 PE=1 SV=2	33	64	4	1.29E+07	1.38E+07	1.49E+07	1.72E+07	1.52E+07	2.10E+07	1.11E+07	1.06E+07	1.02E+07	9.13E+06	8.29E+06	7.45E+06	1.58E+07	9.46E+06	1.67	0.74	0.001754542
Q9NRN5	Olfactomedin-like protein 3 OS=Homo sapiens OX=9606 GN=OLFML3 PE=2 SV=1	12	43	4	2.80E+07	2.63E+07	2.12E+07	2.22E+07	2.27E+07	2.10E+07	2.37E+07	1.96E+07	2.34E+07	2.15E+07	2.13E+07	9.08E+06	2.36E+07	1.98E+07	1.19	0.26	0.169956284
Q10471	Polypeptide N-acetylgalactosaminyltransferase 2 OS=Homo sapiens OX=9606 GN=GALNT2 PE=1 SV=1	10	10	4	4.49E+06	5.16E+06	3.97E+06	2.30E+06	4.45E+06	4.03E+06	4.06E+06	3.02E+06	3.67E+06	3.68E+06	1.92E+06	4.25E+06	4.07E+06	3.43E+06	1.18	0.24	0.255727416
O43172	U4/U6 small nuclear ribonucleoprotein Prp4 OS=Homo sapiens OX=9606 GN=PRPF4 PE=1 SV=2	10	16	4	3.91E+06	1.76E+06	2.82E+06	3.95E+06	3.41E+06	3.17E+06	1.37E+06	8.87E+05	2.36E+06	2.43E+06	1.17E+06	1.74E+06	3.17E+06	1.66E+06	1.91	0.94	0.005380444
P63241	Eukaryotic translation initiation factor 5A-1 OS=Homo sapiens OX=9606 GN=EIF5A PE=1 SV=2	36	65	4	1.29E+07	1.75E+07	1.41E+07	1.58E+07	1.64E+07	1.54E+07	1.13E+07	1.47E+07	1.35E+07	1.67E+07	1.11E+07	1.29E+07	1.54E+07	1.34E+07	1.15	0.20	0.100193863
Q99961	Endophilin-A2 OS=Homo sapiens OX=9606 GN=SH3GL1 PE=1 SV=1	12	24	4	4.10E+06	3.92E+06	3.92E+06	3.66E+06	3.99E+06	3.90E+06	2.22E+06	2.25E+06	2.24E+06	2.23E+06	1.59E+06	2.18E+06	3.92E+06	2.12E+06	1.85	0.89	5.87087E-07
P45974	Ubiquitin carboxyl-terminal hydrolase 5 OS=Homo sapiens OX=9606 GN=USP5 PE=1 SV=2	5	42	4	5.82E+06	4.42E+06	5.58E+06	5.72E+06	5.73E+06	4.24E+06	7.36E+06	7.47E+06	7.64E+06	5.86E+06	6.61E+06	5.56E+06	5.25E+06	6.75E+06	0.78	-0.36	0.009644704
P53004	Biliverdin reductase A OS=Homo sapiens OX=9606 GN=BLVRA PE=1 SV=2	18	50	4	4.20E+06	7.30E+06	4.04E+06	4.78E+06	6.60E+06	6.61E+06	5.90E+06	6.29E+06	5.99E+06	5.71E+06	5.53E+06	4.76E+06	5.59E+06	5.69E+06	0.98	-0.03	0.867922569
P46779	60S ribosomal protein L28 OS=Homo sapiens OX=9606 GN=RPL28 PE=1 SV=3	28	52	4	1.67E+07	1.58E+07	1.92E+07	2.10E+07	1.93E+07	1.45E+07	1.38E+07	1.34E+07	1.60E+07	1.47E+07	1.25E+07	3.13E+06	1.77E+07	1.22E+07	1.45	0.54	0.034339462
O43747	AP-1 complex subunit gamma-1 OS=Homo sapiens OX=9606 GN=AP1G1 PE=1 SV=5	6	22	4	1.90E+06	4.62E+06	7.73E+06	7.88E+06	6.92E+06	6.17E+06	2.42E+06	2.18E+06	3.07E+06	3.11E+06	4.21E+06	4.44E+06	5.87E+06	3.24E+06	1.81	0.86	0.036294548
Q9H0B6	Kinesin light chain 2 OS=Homo sapiens OX=9606 GN=KLC2 PE=1 SV=1	10	24	4	4.20E+06	5.44E+06	6.52E+06	6.42E+06	4.24E+06	6.07E+06	3.00E+06	5.02E+06	2.95E+06		4.86E+06	2.91E+06	5.48E+06	3.75E+06	1.46	0.55	0.026893434
Q8IZP0	Abl interactor 1 OS=Homo sapiens OX=9606 GN=ABI1 PE=1 SV=4	10	14	4	5.98E+06	6.32E+06	6.96E+06	6.53E+06	6.35E+06	4.84E+06	4.59E+06	4.31E+06	3.88E+06	3.94E+06	3.24E+06	4.05E+06	6.16E+06	4.00E+06	1.54	0.62	0.000210215
P07358	Complement component C8 beta chain OS=Homo sapiens OX=9606 GN=C8B PE=1 SV=3	11	21	4	3.19E+06	2.77E+06	4.11E+06	4.15E+06	3.58E+06	4.54E+06	5.86E+06	5.75E+06	5.26E+06	6.23E+06	5.02E+06	4.42E+06	3.72E+06	5.42E+06	0.69	-0.54	0.001212776
P11717	Cation-independent mannose-6-phosphate receptor OS=Homo sapiens OX=9606 GN=IGF2R PE=1 SV=3	2	54	4	1.45E+07	1.28E+07	1.14E+07	1.28E+07	1.25E+07	1.12E+07	2.01E+07	1.86E+07	1.72E+07	2.01E+07	1.33E+07	1.42E+07	1.25E+07	1.73E+07	0.73	-0.46	0.008936043
Q8WVM8	Sec1 family domain-containing protein 1 OS=Homo sapiens OX=9606 GN=SCFD1 PE=1 SV=4	12	8	4	1.45E+06	1.85E+06	1.41E+06	2.05E+06	1.86E+06	1.80E+06	1.40E+06	3.64E+06	1.05E+06	1.25E+06	1.35E+06	2.05E+06	1.74E+06	1.79E+06	0.97	-0.04	0.901008575
Q15493	Regucalcin OS=Homo sapiens OX=9606 GN=RGN PE=1 SV=1	15	64	4	2.54E+07	2.72E+07	2.20E+07	3.09E+07	2.39E+07	2.45E+07	3.19E+07	3.60E+07	3.61E+07	3.89E+07	2.73E+07	2.92E+07	2.57E+07	3.32E+07	0.77	-0.37	0.007920422
P54619	5'-AMP-activated protein kinase subunit gamma-1 OS=Homo sapiens OX=9606 GN=PRKAG1 PE=1 SV=1	19	12	4	5.40E+05	3.46E+05	6.47E+05	8.88E+05	2.40E+05	3.20E+05		4.89E+05	4.50E+05	3.98E+05	1.16E+05	8.25E+05	4.97E+05	4.56E+05	1.09	0.13	0.790746493
O43324	Eukaryotic translation elongation factor 1 epsilon-1 OS=Homo sapiens OX=9606 GN=EEF1E1 PE=1 SV=1	32	14	4	1.13E+07	1.26E+07	1.06E+07	1.11E+07	1.01E+07	9.03E+06	6.76E+06	7.27E+06	4.93E+06	5.77E+06	4.82E+06	5.37E+06	1.08E+07	5.82E+06	1.85	0.89	2.07018E-05
Q9UQE7	Structural maintenance of chromosomes protein 3 OS=Homo sapiens OX=9606 GN=SMC3 PE=1 SV=2	4	20	4	2.31E+06	7.16E+06	4.86E+06	4.67E+06	4.71E+06	4.53E+06	1.27E+06	7.65E+05	9.60E+05	9.11E+05	1.35E+06	2.26E+06	4.71E+06	1.25E+06	3.76	1.91	0.001810189
P62910	60S ribosomal protein L32 OS=Homo sapiens OX=9606 GN=RPL32 PE=1 SV=2	27	61	4	2.65E+07	2.74E+07	2.62E+07	2.96E+07	2.63E+07	2.69E+07	1.82E+07	1.48E+07	1.37E+07	1.52E+07	1.61E+07	1.56E+07	2.71E+07	1.56E+07	1.74	0.80	7.65129E-08
O75165	DnaJ homolog subfamily C member 13 OS=Homo sapiens OX=9606 GN=DNAJC13 PE=1 SV=5	2	8	4	1.72E+06	1.26E+06	1.90E+06	1.67E+06	2.75E+06	2.14E+06	2.55E+06	1.58E+06	1.47E+06	1.04E+06	1.92E+06	1.74E+06	1.91E+06	1.72E+06	1.11	0.15	0.535117128
P28070	Proteasome subunit beta type-4 OS=Homo sapiens OX=9606 GN=PSMB4 PE=1 SV=4	18	48	4	1.84E+07	2.05E+07	2.05E+07	2.37E+07	2.22E+07	1.97E+07	2.12E+07	2.01E+07	2.10E+07	2.20E+07	2.02E+07	1.66E+07	2.08E+07	2.02E+07	1.03	0.05	0.550972287
P25788	Proteasome subunit alpha type-3 OS=Homo sapiens OX=9606 GN=PSMA3 PE=1 SV=2	23	52	4	1.75E+07	2.01E+07	1.82E+07	1.65E+07	1.78E+07	1.63E+07	1.78E+07	1.77E+07	1.50E+07	1.65E+07	1.40E+07	1.38E+07	1.77E+07	1.58E+07	1.12	0.17	0.058585997
A0AVT1	Ubiquitin-like modifier-activating enzyme 6 OS=Homo sapiens OX=9606 GN=UBA6 PE=1 SV=1	4	29	4	4.95E+06	5.60E+06	5.70E+06	5.16E+06	4.92E+06	5.57E+06	5.18E+06	3.93E+06	4.13E+06	1.80E+06	4.11E+06	3.56E+06	5.32E+06	3.79E+06	1.40	0.49	0.018250787
P26373	60S ribosomal protein L13 OS=Homo sapiens OX=9606 GN=RPL13 PE=1 SV=4	19	66	4	2.49E+07	2.44E+07	2.45E+07	2.54E+07	2.25E+07	2.31E+07	1.72E+07	1.58E+07	1.30E+07	1.45E+07	1.36E+07	1.01E+07	2.41E+07	1.40E+07	1.72	0.78	3.62621E-05
P25789	Proteasome subunit alpha type-4 OS=Homo sapiens OX=9606 GN=PSMA4 PE=1 SV=1	16	50	4	1.80E+07	2.19E+07	1.84E+07	2.11E+07	2.09E+07	1.80E+07	1.94E+07	2.00E+07	1.94E+07	1.63E+07	1.90E+07	1.79E+07	1.97E+07	1.87E+07	1.06	0.08	0.286361005
O60716	Catenin delta-1 OS=Homo sapiens OX=9606 GN=CTNND1 PE=1 SV=1	7	7	4	6.04E+06	5.20E+06	5.02E+06	5.11E+06	4.87E+06	4.80E+06	5.08E+06	4.85E+06	6.14E+05	4.77E+06	4.10E+06	3.78E+06	5.17E+06	3.87E+06	1.34	0.42	0.11552467
P35555	Fibrillin-1 OS=Homo sapiens OX=9606 GN=FBN1 PE=1 SV=4	1	8	4							8.60E+05	2.40E+06	9.23E+05	6.22E+05	6.64E+05	3.14E+06		1.43E+06			
P51812	Ribosomal protein S6 kinase alpha-3 OS=Homo sapiens OX=9606 GN=RPS6KA3 PE=1 SV=1	6	39	4	3.26E+06	4.99E+06	4.70E+06	5.95E+06	5.52E+06	4.91E+06	5.38E+06	5.91E+06	3.65E+06	3.56E+06	3.99E+06	5.82E+06	4.89E+06	4.72E+06	1.04	0.05	0.778021883
P35659	Protein DEK OS=Homo sapiens OX=9606 GN=DEK PE=1 SV=1	12	54	4	8.53E+06	7.44E+06	9.49E+06	1.05E+07	9.94E+06	9.08E+06	5.63E+06	7.31E+06	9.18E+06	8.01E+06	7.53E+06	5.41E+06	9.16E+06	7.18E+06	1.28	0.35	0.023811245
Q15185	Prostaglandin E synthase 3 OS=Homo sapiens OX=9606 GN=PTGES3 PE=1 SV=1	19	39	4	4.99E+06	3.90E+06	4.96E+06	4.76E+06	1.17E+07	1.38E+07	4.64E+06	3.64E+06	4.08E+06	9.07E+06	4.33E+06	7.46E+06	7.34E+06	5.54E+06	1.33	0.41	0.383040309
Q99816	Tumor susceptibility gene 101 protein OS=Homo sapiens OX=9606 GN=TSG101 PE=1 SV=2	12	26	4	5.48E+06	5.24E+06	4.16E+06	6.52E+06	2.52E+06	4.43E+06	6.29E+06	5.27E+06	7.75E+06	6.52E+06	7.26E+06	5.47E+06	4.73E+06	6.43E+06	0.74	-0.44	0.034824239
Q13616	Cullin-1 OS=Homo sapiens OX=9606 GN=CUL1 PE=1 SV=2	5	19	4	3.37E+06	3.60E+06	1.93E+06	3.56E+06	2.88E+06	1.54E+06	3.81E+06	1.37E+06	4.52E+06	4.21E+06	4.86E+05		2.81E+06	2.88E+06	0.98	-0.03	0.944076077
P05543	Thyroxine-binding globulin OS=Homo sapiens OX=9606 GN=SERPINA7 PE=1 SV=2	9	166	4	1.71E+07	1.60E+07	1.45E+07	1.41E+07	2.19E+07	1.78E+07	1.99E+07	1.77E+07	1.69E+07	1.50E+07	1.75E+07	1.56E+07	1.69E+07	1.71E+07	0.99	-0.02	0.876206932
P48556	26S proteasome non-ATPase regulatory subunit 8 OS=Homo sapiens OX=9606 GN=PSMD8 PE=1 SV=2	9	39	4	9.68E+06	1.07E+07	9.22E+06	1.02E+07	7.02E+06	6.80E+06	9.04E+06	8.47E+06	8.72E+06	6.37E+06	9.01E+06	6.81E+06	8.95E+06	8.07E+06	1.11	0.15	0.317642821
Q16777	Histone H2A type 2-C OS=Homo sapiens OX=9606 GN=HIST2H2AC PE=1 SV=4	64	3161	5	1.04E+10	9.14E+09	9.08E+09	1.01E+10	9.55E+09	9.77E+09	1.15E+10	1.07E+10	9.90E+09	1.10E+10	9.78E+09	8.59E+09	9.67E+09	1.03E+10	0.94	-0.08	0.265918412
P02538	Keratin, type II cytoskeletal 6A OS=Homo sapiens OX=9606 GN=KRT6A PE=1 SV=3	37	547	5	1.58E+08	7.58E+07	4.61E+07	4.63E+07	4.73E+07	9.47E+07	3.48E+07	1.45E+08	9.48E+07	8.55E+07	6.01E+07	3.77E+07	7.80E+07	7.63E+07	1.02	0.03	0.948056734
Q71UI9	Histone H2A.V OS=Homo sapiens OX=9606 GN=H2AFV PE=1 SV=3	54	1139	5	6.84E+08	6.50E+08	6.56E+08	6.61E+08	7.38E+08	5.87E+08	4.20E+08	4.02E+08	3.30E+08	3.61E+08	2.88E+08	2.90E+08	6.63E+08	3.48E+08	1.90	0.93	1.30272E-06
P62140	Serine/threonine-protein phosphatase PP1-beta catalytic subunit OS=Homo sapiens OX=9606 GN=PPP1CB PE=1 SV=3	44	194	5	1.66E+07	1.59E+07	1.66E+07	1.78E+07	1.71E+07	1.69E+07	1.45E+07	1.14E+07	1.29E+07	1.36E+07	1.30E+07	1.32E+07	1.68E+07	1.31E+07	1.28	0.36	4.79012E-05
P23528	Cofilin-1 OS=Homo sapiens OX=9606 GN=CFL1 PE=1 SV=3	59	182	5	6.83E+07	7.79E+07	6.22E+07	6.70E+07	5.91E+07	7.31E+07	7.53E+07	7.33E+07	4.97E+07	4.22E+07	5.67E+07	5.19E+07	6.79E+07	5.82E+07	1.17	0.22	0.153270067
P36955	Pigment epithelium-derived factor OS=Homo sapiens OX=9606 GN=SERPINF1 PE=1 SV=4	13	616	5	3.92E+08	4.05E+08	2.86E+08	3.18E+08	3.08E+08	3.17E+08	4.62E+08	4.93E+08	3.80E+08	3.71E+08	3.53E+08	3.54E+08	3.38E+08	4.02E+08	0.84	-0.25	0.069712677
Q10567	AP-1 complex subunit beta-1 OS=Homo sapiens OX=9606 GN=AP1B1 PE=1 SV=2	14	194	5	1.70E+07	1.78E+07	1.51E+07	1.54E+07	1.49E+07	1.79E+07	1.35E+07	1.19E+07	1.11E+07	1.16E+07	1.13E+07	8.29E+06	1.64E+07	1.13E+07	1.45	0.54	0.000233445
P31946	14-3-3 protein beta/alpha OS=Homo sapiens OX=9606 GN=YWHAB PE=1 SV=3	39	89	5	6.81E+06	7.73E+06	1.28E+07	1.64E+07	9.24E+06	8.02E+06	7.86E+06	9.06E+06	8.70E+06	7.75E+06	8.55E+06	6.05E+06	1.02E+07	8.00E+06	1.27	0.35	0.219056139
P36507	Dual specificity mitogen-activated protein kinase kinase 2 OS=Homo sapiens OX=9606 GN=MAP2K2 PE=1 SV=1	36	106	5	1.36E+07	1.43E+07	1.31E+07	1.49E+07	1.33E+07	1.39E+07	9.83E+06	9.03E+06	8.01E+06	7.59E+06	6.90E+06	8.62E+06	1.39E+07	8.33E+06	1.67	0.74	2.69903E-06
P62258	14-3-3 protein epsilon OS=Homo sapiens OX=9606 GN=YWHAE PE=1 SV=1	25	83	5	1.60E+07	2.04E+07	2.12E+07	2.91E+07	2.71E+07	2.69E+07	1.40E+07	1.81E+07	1.98E+07	2.17E+07	1.76E+07	1.42E+07	2.35E+07	1.76E+07	1.34	0.42	0.039169243
P61981	14-3-3 protein gamma OS=Homo sapiens OX=9606 GN=YWHAG PE=1 SV=2	33	99	5	8.54E+06	9.68E+06	1.14E+07	1.39E+07	1.14E+07	1.15E+07	8.15E+06	1.17E+07	9.38E+06	1.24E+07	1.05E+07	8.65E+06	1.10E+07	1.01E+07	1.09	0.13	0.382799466
P34896	Serine hydroxymethyltransferase, cytosolic OS=Homo sapiens OX=9606 GN=SHMT1 PE=1 SV=1	16	151	5	1.78E+07	1.83E+07	2.28E+07	2.12E+07	2.38E+07	1.74E+07	2.33E+07	1.57E+07	2.12E+07	2.29E+07	2.05E+07	1.80E+07	2.02E+07	2.02E+07	1.00	0.00	0.987347147
P80723	Brain acid soluble protein 1 OS=Homo sapiens OX=9606 GN=BASP1 PE=1 SV=2	48	82	5	1.48E+07	1.32E+07	2.12E+07	2.34E+07	1.59E+07	1.90E+07	2.00E+07	1.84E+07	2.32E+07	2.13E+07	1.45E+07	1.44E+07	1.79E+07	1.86E+07	0.96	-0.06	0.754653108
O14773	Tripeptidyl-peptidase 1 OS=Homo sapiens OX=9606 GN=TPP1 PE=1 SV=2	15	104	5	3.14E+07	3.05E+07	3.17E+07	2.75E+07	2.89E+07	3.15E+07	2.19E+07	2.13E+07	2.09E+07	1.30E+07	2.14E+07	1.54E+07	3.02E+07	1.90E+07	1.59	0.67	0.000313032
O00303	Eukaryotic translation initiation factor 3 subunit F OS=Homo sapiens OX=9606 GN=EIF3F PE=1 SV=1	21	100	5	1.58E+07	2.47E+07	2.95E+07	3.44E+07	3.77E+07	2.31E+07	1.19E+07	1.52E+07	1.69E+07	1.30E+07	1.47E+07	1.38E+07	2.75E+07	1.42E+07	1.93	0.95	0.00888529
P06732	Creatine kinase M-type OS=Homo sapiens OX=9606 GN=CKM PE=1 SV=2	24	66	5	1.24E+07	1.40E+07	1.19E+07	1.45E+07	1.33E+07	1.46E+07	2.11E+07	2.11E+07	1.49E+07	1.32E+07	1.54E+07	1.75E+07	1.34E+07	1.72E+07	0.78	-0.35	0.038577006
P53985	Monocarboxylate transporter 1 OS=Homo sapiens OX=9606 GN=SLC16A1 PE=1 SV=3	12	48	5	1.44E+07	1.43E+07	1.49E+07	1.97E+07	1.79E+07	1.59E+07	8.07E+06	6.27E+06	8.68E+06	7.09E+06	8.55E+06	5.91E+06	1.62E+07	7.43E+06	2.18	1.12	2.84234E-05
P05161	Ubiquitin-like protein ISG15 OS=Homo sapiens OX=9606 GN=ISG15 PE=1 SV=5	31	116	5	3.86E+07	4.26E+07	3.29E+07	3.77E+07	3.94E+07	3.35E+07	2.51E+07	2.44E+07	2.49E+07	2.64E+07	2.23E+07	1.70E+07	3.74E+07	2.34E+07	1.60	0.68	4.3674E-05
P49411	Elongation factor Tu, mitochondrial OS=Homo sapiens OX=9606 GN=TUFM PE=1 SV=2	16	64	5	2.07E+07	2.02E+07	2.26E+07	2.30E+07	1.83E+07	1.96E+07	1.24E+07	1.14E+07	8.12E+06	1.10E+07	1.02E+07	8.00E+06	2.07E+07	1.02E+07	2.03	1.02	1.42903E-06

Supplementary Table 4. List of all proteins identified by label free proteomics analysis.

Accession	Description	Coverage [%]	# PSMs	# Unique Peptides	Amitriptyline 1	Amitriptyline 2	Amitriptyline 3	Amitriptyline 4	Amitriptyline 5	Amitriptyline 6	Vehicle 1	Vehicle 2	Vehicle 3	Vehicle 4	Vehicle 5	Vehicle 6	Average amitriptyline	Average vehicle	Ratio amitriptyline/ vehicle	Log 2 fold change (amitriptyline /vehicle)	P-value
P01834	Immunoglobulin kappa constant OS=Homo sapiens OX=9606 GN=IGKC PE=1 SV=2	79	15	5	1.02E+06						4.28E+06	2.56E+05			3.65E+05	1.98E+07	1.02E+06	6.18E+06	0.16	-2.60	
Q9Y240	C-type lectin domain family 11 member A OS=Homo sapiens OX=9606 GN=CLEC11A PE=1 SV=1	15	261	5	9.65E+07	8.48E+07	8.38E+07	8.63E+07	7.80E+07	7.39E+07	1.22E+08	1.10E+08	9.88E+07	1.02E+08	9.51E+07	8.28E+07	8.39E+07	1.02E+08	0.82	-0.28	0.021382525
Q02750	Dual specificity mitogen-activated protein kinase kinase 1 OS=Homo sapiens OX=9606 GN=MAP2K1 PE=1 SV=2	34	66	5	1.61E+07	1.69E+07	1.90E+07	2.26E+07	1.75E+07	1.75E+07	1.37E+07	1.63E+07	1.32E+07	1.86E+07	1.39E+07	1.52E+07	1.83E+07	1.52E+07	1.20	0.27	0.034602911
P13804	Electron transfer flavoprotein subunit alpha, mitochondrial OS=Homo sapiens OX=9606 GN=ETFA PE=1 SV=1	27	42	5	7.63E+06	7.46E+06	1.04E+07	1.03E+07	7.46E+06	1.14E+07	6.06E+06	3.16E+06	5.17E+06	2.99E+06	3.78E+06	3.32E+06	9.11E+06	4.08E+06	2.23	1.16	0.000318344
P30613	Pyruvate kinase PKLR OS=Homo sapiens OX=9606 GN=PKLR PE=1 SV=2	13	196	5	1.42E+07	1.59E+07	1.16E+07	1.16E+07	1.07E+07	1.16E+07	1.78E+07	1.98E+07	1.49E+07	1.55E+07	1.35E+07	1.17E+07	1.26E+07	1.55E+07	0.81	-0.30	0.073344756
P52907	F-actin-capping protein subunit alpha-1 OS=Homo sapiens OX=9606 GN=CAPZA1 PE=1 SV=3	43	96	5	2.30E+07	2.20E+07	1.84E+07	2.44E+07	1.80E+07	2.46E+07	1.82E+07	1.56E+07	1.55E+07	1.55E+07	1.59E+07	1.44E+07	2.17E+07	1.59E+07	1.37	0.45	0.002975255
P16278	Beta-galactosidase OS=Homo sapiens OX=9606 GN=GLB1 PE=1 SV=2	13	36	5	1.31E+07	1.24E+07	1.06E+07	8.56E+06	6.56E+06	6.15E+06	3.46E+06	3.49E+06	1.27E+06	3.84E+06	3.66E+06	2.16E+06	9.57E+06	2.98E+06	3.21	1.68	0.001890192
P17844	Probable ATP-dependent RNA helicase DDX5 OS=Homo sapiens OX=9606 GN=DDX5 PE=1 SV=1	20	105	5	9.90E+06	1.16E+07	1.05E+07	1.02E+07	1.08E+07	8.26E+06	5.33E+06	6.22E+06	4.79E+06	5.40E+06	4.52E+06	3.79E+06	1.02E+07	5.01E+06	2.04	1.03	6.16793E-06
O00487	26S proteasome non-ATPase regulatory subunit 14 OS=Homo sapiens OX=9606 GN=PSMD14 PE=1 SV=1	36	63	5	1.92E+07	2.30E+07	2.31E+07	1.72E+07	1.77E+07	1.42E+07	1.16E+07	1.92E+07	9.12E+06	7.35E+06	8.91E+06	9.83E+06	1.91E+07	1.10E+07	1.74	0.80	0.004985815
P06858	Lipoprotein lipase OS=Homo sapiens OX=9606 GN=LPL PE=1 SV=1	19	52	5	1.08E+07	9.72E+06	6.17E+06	1.01E+07	1.10E+07	9.79E+06	1.57E+07	1.35E+07	1.41E+07	1.34E+07	1.18E+07	1.25E+07	9.60E+06	1.35E+07	0.71	-0.49	0.001792388
O43175	D-3-phosphoglycerate dehydrogenase OS=Homo sapiens OX=9606 GN=PHGDH PE=1 SV=4	15	112	5	4.05E+07	4.23E+07	3.45E+07	4.36E+07	3.18E+07	3.62E+07	5.10E+07	5.20E+07	4.43E+07	4.18E+07	4.34E+07	3.86E+07	3.82E+07	4.52E+07	0.84	-0.24	0.034862253
Q9Y696	Chloride intracellular channel protein 4 OS=Homo sapiens OX=9606 GN=CLIC4 PE=1 SV=4	35	92	5	2.15E+07	1.79E+07	1.91E+07	1.93E+07	1.95E+07	1.93E+07	2.08E+07	1.79E+07	1.64E+07	2.04E+07	1.45E+07	1.52E+07	1.94E+07	1.75E+07	1.11	0.15	0.14832385
P30050	60S ribosomal protein L12 OS=Homo sapiens OX=9606 GN=RPL12 PE=1 SV=1	45	142	5	3.88E+07	4.15E+07	3.07E+07	2.72E+07	3.13E+07	3.34E+07	3.38E+07	4.05E+07	2.90E+07	3.08E+07	2.72E+07	2.65E+07	3.38E+07	3.13E+07	1.08	0.11	0.430112375
P21926	CD9 antigen OS=Homo sapiens OX=9606 GN=CD9 PE=1 SV=4	21	179	5	1.01E+08	9.41E+07	9.44E+07	1.09E+08	9.57E+07	9.34E+07	7.68E+07	7.32E+07	7.22E+07	6.42E+07	6.12E+07	6.42E+07	9.79E+07	6.86E+07	1.43	0.51	8.4871E-06
Q9Y4K0	Lysyl oxidase homolog 2 OS=Homo sapiens OX=9606 GN=LOXL2 PE=1 SV=1	11	40	5	5.42E+06	6.29E+06	5.30E+06	5.92E+06	5.19E+06	4.71E+06	1.15E+07	1.16E+07	1.16E+07	9.89E+06	9.13E+06	8.83E+06	5.47E+06	1.04E+07	0.52	-0.93	6.99966E-05
P10644	cAMP-dependent protein kinase type I-alpha regulatory subunit OS=Homo sapiens OX=9606 GN=PRKAR1A PE=1 SV=1	22	82	5	1.23E+07	1.12E+07	9.75E+06	1.27E+07	1.40E+07	1.13E+07	1.28E+07	1.24E+07	7.55E+06	7.83E+06	1.24E+07	1.08E+07	1.19E+07	1.06E+07	1.12	0.16	0.297722065
O14786	Neuropilin-1 OS=Homo sapiens OX=9606 GN=NRP1 PE=1 SV=3	11	52	5	1.34E+07	1.46E+07	1.25E+07	1.41E+07	1.16E+07	1.04E+07	1.05E+07	9.38E+06	7.56E+06	9.75E+06	7.60E+06	7.86E+06	1.28E+07	8.78E+06	1.45	0.54	0.000812827
P22234	Multifunctional protein ADE2 OS=Homo sapiens OX=9606 GN=PAICS PE=1 SV=3	14	45	5	1.19E+07	1.36E+07	6.33E+06	8.07E+06	6.12E+06	1.26E+07	5.78E+06	6.46E+06	3.37E+06	3.42E+06	5.30E+06	5.72E+06	9.77E+06	5.01E+06	1.95	0.97	0.015273477
O00151	PDZ and LIM domain protein 1 OS=Homo sapiens OX=9606 GN=PDLIM1 PE=1 SV=4	21	24	5	1.27E+07	1.09E+07	7.60E+06	9.94E+06	1.03E+07	1.01E+07	6.99E+06	7.05E+06	4.52E+06	5.86E+06	5.37E+06	5.54E+06	1.03E+07	5.89E+06	1.74	0.80	0.000500038
P20618	Proteasome subunit beta type-1 OS=Homo sapiens OX=9606 GN=PSMB1 PE=1 SV=2	32	76	5	2.01E+07	2.03E+07	2.04E+07	2.10E+07	2.19E+07	2.31E+07	2.31E+07	2.23E+07	2.30E+07	2.37E+07	2.03E+07	1.79E+07	2.11E+07	2.17E+07	0.97	-0.04	0.598565877
P27361	Mitogen-activated protein kinase 3 OS=Homo sapiens OX=9606 GN=MAPK3 PE=1 SV=4	25	76	5	2.81E+07	2.60E+07	1.81E+07	2.44E+07	2.19E+07	2.22E+07	1.88E+07	2.18E+07	1.80E+07	2.00E+07	1.77E+07	1.60E+07	2.34E+07	1.87E+07	1.25	0.33	0.020872159
P11279	Lysosome-associated membrane glycoprotein 1 OS=Homo sapiens OX=9606 GN=LAMP1 PE=1 SV=3	12	126	5	6.23E+07	5.77E+07	5.88E+07	5.94E+07	5.52E+07	5.30E+07	2.05E+07	3.26E+07	3.07E+07	1.82E+07	1.81E+07	2.63E+07	5.77E+07	2.44E+07	2.37	1.24	5.57772E-06
P61019	Ras-related protein Rab-2A OS=Homo sapiens OX=9606 GN=RAB2A PE=1 SV=1	32	80	5	1.40E+07	1.21E+07	1.52E+07	1.34E+07	1.47E+07	1.53E+07	1.27E+07	1.06E+07	1.12E+07	9.47E+06	1.09E+07	8.89E+06	1.41E+07	1.06E+07	1.32	0.41	0.000985454
P52292	Importin subunit alpha-1 OS=Homo sapiens OX=9606 GN=KPNA2 PE=1 SV=1	19	26	5	9.66E+06	6.44E+06	4.96E+06	6.12E+06	3.96E+06	7.21E+06	4.46E+06	2.14E+06	1.96E+06	8.19E+05	2.80E+06	2.43E+06	6.39E+06	2.44E+06	2.62	1.39	0.002783473
Q6V0I7	Protocadherin Fat 4 OS=Homo sapiens OX=9606 GN=FAT4 PE=1 SV=2	2	26	5	4.67E+06	3.73E+06	4.64E+06	3.10E+06	4.70E+06	3.50E+06	7.24E+06	7.46E+06	6.02E+06	5.83E+06	6.19E+06	7.14E+06	4.06E+06	6.65E+06	0.61	-0.71	8.19768E-05
Q15435	Protein phosphatase 1 regulatory subunit 7 OS=Homo sapiens OX=9606 GN=PPP1R7 PE=1 SV=1	19	90	5	1.10E+07	1.48E+07	1.20E+07	8.35E+06	1.47E+07	1.27E+07	1.37E+07	8.95E+06	1.34E+07	1.67E+07	1.18E+07	1.20E+07	1.23E+07	1.28E+07	0.96	-0.06	0.734981671
P35268	60S ribosomal protein L22 OS=Homo sapiens OX=9606 GN=RPL22 PE=1 SV=2	49	64	5	5.15E+07	4.91E+07	4.93E+07	5.14E+07	4.93E+07	4.71E+07	2.43E+07	2.47E+07	1.91E+07	2.28E+07	2.20E+07	1.92E+07	4.96E+07	2.20E+07	2.25	1.17	3.8406E-09
P60900	Proteasome subunit alpha type-6 OS=Homo sapiens OX=9606 GN=PSMA6 PE=1 SV=1	25	115	5	4.05E+07	4.58E+07	4.04E+07	3.98E+07	4.14E+07	3.38E+07	4.53E+07	4.77E+07	4.93E+07	5.82E+07	4.23E+07	3.27E+07	4.03E+07	4.59E+07	0.88	-0.19	0.179858842
Q9UBQ7	Glyoxylate reductase/hydroxypyruvate reductase OS=Homo sapiens OX=9606 GN=GRHPR PE=1 SV=1	26	66	5	8.73E+06	9.20E+06	9.21E+06	1.15E+07	8.29E+06	1.16E+07	1.00E+07	9.92E+06	9.63E+06	1.08E+07	1.09E+07	9.65E+06	9.76E+06	1.01E+07	0.96	-0.06	0.562018192
P61225	Ras-related protein Rap-2b OS=Homo sapiens OX=9606 GN=RAP2B PE=1 SV=1	33	66	5	1.85E+07	1.83E+07	1.70E+07	1.88E+07	2.06E+07	2.30E+07	1.78E+07	1.66E+07	1.59E+07	1.81E+07	1.44E+07	1.61E+07	1.94E+07	1.65E+07	1.18	0.23	0.021366338
Q09666	Neuroblast differentiation-associated protein AHNAK OS=Homo sapiens OX=9606 GN=AHNAK PE=1 SV=2	3	27	5	3.84E+06	5.61E+06	8.92E+06	9.47E+06	6.91E+06	1.13E+07	7.25E+06	8.82E+06	8.62E+06	7.20E+06	8.20E+06	5.66E+06	7.68E+06	7.63E+06	1.01	0.01	0.969113912
Q9BR76	Coronin-1B OS=Homo sapiens OX=9606 GN=CORO1B PE=1 SV=1	14	66	5	2.03E+07	1.75E+07	1.75E+07	1.75E+07	1.79E+07	1.41E+07	1.97E+07	1.82E+07	1.83E+07	1.59E+07	1.78E+07	1.53E+07	1.75E+07	1.75E+07	1.00	0.00	0.970067923
P47756	F-actin-capping protein subunit beta OS=Homo sapiens OX=9606 GN=CAPZB PE=1 SV=4	26	50	5	1.51E+07	1.50E+07	1.34E+07	1.26E+07	1.19E+07	1.39E+07	9.57E+06	1.16E+07	1.03E+07	9.71E+06	7.71E+06	7.58E+06	1.36E+07	9.41E+06	1.45	0.53	0.000481615
Q9BXS5	AP-1 complex subunit mu-1 OS=Homo sapiens OX=9606 GN=AP1M1 PE=1 SV=3	18	61	5	1.28E+07	1.29E+07	1.50E+07	1.40E+07	1.33E+07	1.23E+07	7.88E+06	7.54E+06	5.41E+06	6.78E+06	5.27E+06	5.69E+06	1.34E+07	6.43E+06	2.08	1.06	5.59084E-07
Q96IJ6	Mannose-1-phosphate guanyltransferase alpha OS=Homo sapiens OX=9606 GN=GMPPA PE=1 SV=1	15	60	5	6.63E+06	7.10E+06	8.22E+06	8.71E+06	7.61E+06	6.13E+06	9.09E+06	8.77E+06	7.53E+06	8.97E+06	7.95E+06	6.29E+06	7.40E+06	8.10E+06	0.91	-0.13	0.265446138
P12004	Proliferating cell nuclear antigen OS=Homo sapiens OX=9606 GN=PCNA PE=1 SV=1	25	40	5	6.63E+06	1.22E+07	1.52E+07	1.40E+07	1.50E+07	1.31E+07	5.89E+06	1.09E+07	6.54E+06	1.22E+07	9.93E+06	1.08E+07	1.27E+07	9.38E+06	1.35	0.44	0.077290312
P07686	Beta-hexosaminidase subunit beta OS=Homo sapiens OX=9606 GN=HEXB PE=1 SV=3	15	17	5	5.18E+06	3.34E+06	4.16E+05	2.34E+06	1.69E+06	1.77E+06	6.27E+05	1.76E+06	6.21E+05	1.22E+06	8.90E+05	1.85E+06	2.46E+06	1.16E+06	2.11	1.08	0.115498476
Q96KP4	Cytosolic non-specific dipeptidase OS=Homo sapiens OX=9606 GN=CNDP2 PE=1 SV=2	19	91	5	1.56E+07	2.93E+07	2.37E+07	1.67E+07	1.51E+07	1.82E+07	1.73E+07	2.43E+07	2.42E+07	1.88E+07	2.53E+07	1.68E+07	1.98E+07	2.11E+07	0.94	-0.09	0.643617171
P04062	Lysosomal acid glucosylceramidase OS=Homo sapiens OX=9606 GN=GBA PE=1 SV=3	15	42	5	7.62E+06	6.78E+06	7.85E+06	5.88E+06	6.87E+06	6.11E+06	5.90E+06	5.59E+06	4.51E+06	4.12E+06	5.15E+06	4.50E+06	6.85E+06	4.96E+06	1.38	0.47	0.001357749
P27797	Calreticulin OS=Homo sapiens OX=9606 GN=CALR PE=1 SV=1	17	82	5	2.02E+07	2.07E+07	2.18E+07	1.84E+07	2.20E+07	1.90E+07	1.98E+07	2.07E+07	1.78E+07	1.58E+07	1.66E+07	1.69E+07	2.03E+07	1.79E+07	1.13	0.18	0.035794604
P23368	NAD-dependent malic enzyme, mitochondrial OS=Homo sapiens OX=9606 GN=ME2 PE=1 SV=1	18	25	5	6.11E+06	3.75E+06	3.51E+06	5.36E+06	4.53E+06	5.87E+06	5.98E+06	4.01E+06	4.29E+06	5.41E+06	3.07E+06	3.32E+06	4.85E+06	4.35E+06	1.12	0.16	0.453001888
P36776	Lon protease homolog, mitochondrial OS=Homo sapiens OX=9606 GN=LONP1 PE=1 SV=2	8	17	5	2.53E+06	2.53E+06	1.78E+06	2.27E+06	2.17E+06	2.32E+06	2.19E+06	1.85E+06	1.83E+06	1.68E+06	1.66E+06	1.42E+06	2.27E+06	1.77E+06	1.28	0.36	0.009350397
P02792	Ferritin light chain OS=Homo sapiens OX=9606 GN=FTL PE=1 SV=2	40	53	5	1.13E+07	1.90E+07	1.28E+07	1.49E+07	1.22E+07	1.19E+07	2.15E+07	1.72E+07	2.19E+07	3.44E+07	2.06E+07	1.32E+07	1.37E+07	2.15E+07	0.64	-0.65	0.043533764
Q5VYK3	Proteasome adapter and scaffold protein ECM29 OS=Homo sapiens OX=9606 GN=ECPAS PE=1 SV=2	5	25	5	6.62E+06	4.18E+06	4.80E+06	4.68E+06	4.78E+06	5.28E+06	3.96E+06	2.77E+06	2.68E+06	2.96E+06	3.16E+06	2.99E+06	5.06E+06	3.09E+06	1.64	0.71	0.001122316
O75351	Vacuolar protein sorting-associated protein 4B OS=Homo sapiens OX=9606 GN=VPS4B PE=1 SV=2	20	47	5	1.08E+07	8.59E+06	8.40E+06	6.57E+06	7.51E+06	1.23E+07	6.46E+06	7.44E+06	6.18E+06	3.77E+06	5.48E+06	4.84E+06	9.03E+06	5.70E+06	1.58	0.66	0.010976714
P13861	cAMP-dependent protein kinase type II-alpha regulatory subunit OS=Homo sapiens OX=9606 GN=PRKAR2A PE=1 SV=2	15	85	5	1.74E+07	1.89E+07	1.75E+07	1.86E+07	1.74E+07	1.78E+07	2.09E+07	2.29E+07	2.00E+07	2.09E+07	1.69E+07	1.56E+07	1.79E+07	1.95E+07	0.92	-0.12	0.21276132
P43243	Matrin-3 OS=Homo sapiens OX=9606 GN=MATR3 PE=1 SV=2	12	30	5	9.61E+06	7.61E+06	8.51E+06	1.00E+07	6.20E+06	7.35E+06	2.46E+06	2.57E+06	2.07E+06	2.57E+06	3.04E+06	2.14E+06	8.22E+06	2.47E+06	3.32	1.73	0.000121366
O60506	Heterogeneous nuclear ribonucleoprotein Q OS=Homo sapiens OX=9606 GN=SYNCRIP PE=1 SV=2	11	56	5	1.62E+07	1.76E+07	1.73E+07	1.58E+07	1.71E+07	1.56E+07	1.30E+07	1.05E+07	9.27E+06	9.31E+06	1.01E+07	7.55E+06	1.66E+07	9.96E+06	1.67	0.74	7.59348E-05
P02771	Alpha-fetoprotein OS=Homo sapiens OX=9606 GN=AFP PE=1 SV=1	11	144	5	3.98E+07	3.97E+07	4.10E+07	4.88E+07	4.16E+07	3.78E+07	6.95E+07	6.76E+07	6.59E+07	6.52E+07	6.56E+07	5.71E+07	4.15E+07	6.52E+07	0.64	-0.65	1.56342E-06
P33991	DNA replication licensing factor MCM4 OS=Homo sapiens OX=9606 GN=MCM4 PE=1 SV=5	11	35	5	6.63E+06	8.08E+06	6.24E+06	5.87E+06	5.05E+06	8.19E+06	3.68E+06	5.04E+06	2.43E+06	2.28E+06	3.84E+06	2.99E+06	6.68E+06	3.38E+06	1.98	0.98	0.000596737
Q9UQ80	Proliferation-associated protein 2G4 OS=Homo sapiens OX=9606 GN=PA2G4 PE=1 SV=3	17	47	5	1.01E+07	1.24E+07	1.20E+07	1.23E+07	1.27E+07	1.18E+07	1.03E+07	1.03E+07	1.32E+07	1.24E+07	9.78E+06	9.46E+06	1.19E+07	1.09E+07	1.09	0.12	0.226877842
Q15365	Poly(rC)-binding protein 1 OS=Homo sapiens OX=9606 GN=PCBP1 PE=1 SV=2	22	110	5	3.54E+07	3.38E+07	2.77E+07	3.10E+07	3.06E+07	3.01E+07	2.63E+07	2.65E+07	2.27E+07	2.24E+07	2.06E+07	1.97E+07	3.15E+07	2.30E+07	1.37	0.45	0.000383193
Q9BTW9	Tubulin-specific chaperone D OS=Homo sapiens OX=9606 GN=TBCD PE=1 SV=2	8	32	5	6.15E+06	6.30E+06	6.13E+06	6.49E+06	6.58E+06	5.51E+06	3.11E+06	3.83E+06	4.12E+06	2.06E+06	4.03E+06	2.25E+06	6.19E+06	3.23E+06	1.92	0.94	0.000191785
P25205	DNA replication licensing factor MCM3 OS=Homo sapiens OX=9606 GN=MCM3 PE=1 SV=3	10	44	5	1.04E+07	8.82E+06	8.87E+06	8.62E+06	8.63E+06	8.66E+06	6.62E+06	6.13E+06	5.33E+06	4.92E+06	5.51E+06	5.11E+06	9.01E+06	5.60E+06	1.61	0.69	6.13952E-06
Q9Y6M1	Insulin-like growth factor 2 mRNA-binding protein 2 OS=Homo sapiens OX=9606 GN=IGF2BP2 PE=1 SV=2	11	24	5	3.93E+06	4.20E+06	3.68E+06	3.80E+06	3.99E+06	3.32E+06	1.71E+06	1.90E+06	1.87E+06	1.78E+06	1.16E+06	9.68E+05	3.82E+06	1.57E+06	2.44	1.29	1.20321E-06
P62316	Small nuclear ribonucleoprotein Sm D2 OS=Homo sapiens OX=9606 GN=SNRPD2 PE=1 SV=1	47	68	5	3.45E+07	2.64E+07	2.82E+07	2.77E+07	2.63E+07	3.42E+07	2.25E+07	2.42E+07	2.04E+07	7.11E+06	1.86E+07	2.12E+07	2.96E+07	1.90E+07	1.56	0.64	0.006621797
P27816	Microtubule-associated protein 4 OS=Homo sapiens OX=9606 GN=MAP4 PE=1 SV=3	6	56	5	1.39E+07	1.52E+07	1.48E+07	1.61E+07	1.45E+07	1.31E+07	6.64E+06	6.15E+06	6.32E+06	5.74E+06	6.10E+06	4.06E+06	1.46E+07	5.83E+06	2.50	1.32	2.52748E-08
P34932	Heat shock 70 kDa protein 4 OS=Homo sapiens OX=9606 GN=HSPA4 PE=1 SV=4	8	82	5	1.57E+07	1.51E+07	1.52E+07	1.55E+07	1.65E+07	1.76E+07	1.94E+07	1.84E+07	1.64E+07	1.84E+07	1.40E+07	1.48E+07	1.59E+07	1.69E+07	0.94	-0.09	0.345171426
Q00341	Vigilin OS=Homo sapiens OX=9606 GN=HDLBP PE=1 SV=2	6	40	5	9.11E+06	1.08E+07	7.95E+06	8.51E+06	7.19E+06	8.08E+06	5.16E+06	4.72E+06	4.06E+06	3.95E+06	5.15E+06	4.62E+06	8.60E+06	4.61E+06	1.87	0.90	0.000201907
Q9Y3F4	Serine-threonine kinase receptor-associated protein OS=Homo sapiens OX=9606 GN=STRAP PE=1 SV=1	24	34	5	1.05E+07	1.18E+07	1.01E+07	1.12E+07	1.19E+07	1.16E+07	1.14E+07	9.83E+06	1.05E+07	9.85E+06	6.47E+06	7.24E+06	1.12E+07	9.21E+06	1.21	0.28	0.054658688
P54709	Sodium/potassium-transporting ATPase subunit beta-3 OS=Homo sapiens OX=9606 GN=ATP1B3 PE=1 SV=1	21	62	5	2.02E+07	2.12E+07	1.59E+07	1.94E+07	1.76E+07	1.36E+07	1.44E+07	1.43E+07	1.33E+07	1.45E+07	1.09E+07	1.13E+07	1.80E+07	1.31E+07	1.37	0.45	0.006781486
Q99497	Protein/nucleic acid deglycase DJ-1 OS=Homo sapiens OX=9606 GN=PARK7 PE=1 SV=2	39	53	5	9.49E+06	8.05E+06	1.21E+07	1.06E+07	1.15E+07	1.01E+07	9.31E+06	7.26E+06	8.94E+06	8.18E+06	7.39E+06	7.27E+06	1.03E+07	8.06E+06	1.28	0.35	0.011315917
Q14166	Tubulin--tyrosine ligase-like protein 12 OS=Homo sapiens OX=9606 GN=TTLL12 PE=1 SV=2	11	34	5	8.17E+06	6.43E+06	5.84E+06	8.56E+06	5.17E+06	6.21E+06	6.63E+06	5.30E+06	6.24E+06	7.87E+06	6.99E+06	4.26E+06	6.73E+06	6.21E+06	1.08	0.11	0.512186462
O15118	NPC intracellular cholesterol transporter 1 OS=Homo sapiens OX=9606 GN=NPC1 PE=1 SV=2	4	42	5	1.56E+07	1.32E+07	1.37E+07	1.60E+07	1.51E+07	1.21E+07	1.32E+07	7.41E+06	1.02E+07	1.16E+07	1.11E+07	7.45E+06	1.43E+07	1.02E+07	1.40	0.49	0.006053696
P20073	Annexin A7 OS=Homo sapiens OX=9606 GN=ANXA7 PE=1 SV=3	13	108	5	1.99E+07	1.87E+07	1.36E+07	1.49E+07	1.36E+07	1.49E+07	1.67E+07	1.79E+07	1.25E+07	1.22E+07	1.20E+07	1.28E+07	1.59E+07	1.40E+07	1.14	0.18	0.238708273
Q9BY44	Eukaryotic translation initiation factor 2A OS=Homo sapiens OX=9606 GN=EIF2A PE=1 SV=3	11	26	5	8.84E+06	3.67E+06	6.74E+06	7.36E+06	7.52E+06	6.59E+06	2.47E+06	2.77E+06	3.13E+06	2.33E+06	3.50E+06	3.21E+06	6.79E+06	2.90E+06	2.34	1.23	0.002071084
P61254	60S ribosomal protein L26 OS=Homo sapiens OX=9606 GN=RPL26 PE=1 SV=1	32	91	5	2.08E+07	1.85E+07	2.54E+07	2.60E+07	2.09E+07	1.88E+07	1.58E+07	1.49E+07	1.75E+07	2.33E+07	1.14E+07	1.32E+07	2.17E+07	1.60E+07	1.35	0.44	0.025386429
Q13228	Methanethiol oxidase OS=Homo sapiens OX=9606 GN=SELENBP1 PE=1 SV=2	16	39	5	6.16E+06	6.36E+06	5.34E+06	3.99E+06	4.85E+06	6.32E+06	9.24E+06	7.76E+06	6.86E+06	4.56E+06	7.35E+06	8.62E+06	5.50E+06	7.40E+06	0.74	-0.43	0.039496506
Q9Y617	Phosphoserine aminotransferase OS=Homo sapiens OX=9606 GN=PSAT1 PE=1 SV=2	16	76	5	1.51E+07	1.64E+07	1.50E+07	1.42E+07	1.20E+07	1.34E+07	1.83E+07	1.80E+07	1.71E+07	1.42E+07	1.79E+07	1.26E+07	1.43E+07	1.63E+07	0.88	-0.19	0.117640796
Q15181	Inorganic pyrophosphatase OS=Homo sapiens OX=9606 GN=PPA1 PE=1 SV=2	26	32	5	1.06E+07	8.62E+06	8.62E+06	9.53E+06	8.18E+06	1.18E+07	8.94E+06	1.33E+07	1.19E+07	6.12E+06	5.81E+06	5.69E+06	9.55E+06	8.64E+06	1.11	0.15	0.557071521
Q9UBQ0	Vacuolar protein sorting-associated protein 29 OS=Homo sapiens OX=9606 GN=VPS29 PE=1 SV=1	34	59	5	1.73E+07	1.75E+07	1.73E+07	1.87E+07	1.90E+07	1.72E+07	1.56E+07	1.59E+07	1.57E+07	1.67E+07	1.55E+07	1.57E+07	1.78E+07	1.58E+07	1.13	0.17	0.000669906
P04181	Ornithine aminotransferase, mitochondrial OS=Homo sapiens OX=9606 GN=OAT PE=1 SV=1	15	29	5	7.33E+06	7.43E+06	6.50E+06	6.20E+06	6.96E+06	7.40E+06	4.81E+06	4.63E+06	4.32E+06	4.15E+06	3.46E+06	3.33E+06	6.97E+06	4.12E+06	1.69	0.76	5.95811E-06
P40429	60S ribosomal protein L13a OS=Homo sapiens OX=9606 GN=RPL13A PE=1 SV=2	24	70	5	3.95E+07	4.99E+07	4.55E+07	4.79E+07	4.38E+07	4.67E+07	2.89E+07	2.84E+07	2.82E+07	3.15E+07	2.93E+07	2.79E+07	4.56E+07	2.90E+07	1.57	0.65	3.23595E-05
P07339	Cathepsin D OS=Homo sapiens OX=9606 GN=CTSD PE=1 SV=1	15	86	5	2.61E+07	2.50E+07	2.32E+07	2.19E+07	3.05E+07	2.33E+07	1.36E+07	1.40E+07	1.08E+07	1.13E+07	1.17E+07	1.01E+07	2.50E+07	1.19E+07	2.10	1.07	2.45181E-05
Q8TD19	Serine/threonine-protein kinase Nek9 OS=Homo sapiens OX=9606 GN=NEK9 PE=1 SV=2	6	45	5	3.38E+06	4.54E+06	3.16E+06	3.07E+06	3.42E+06	3.07E+06	3.09E+06	3.23E+06	2.07E+06	1.72E+06	2.75E+06	3.07E+06	3.44E+06	2.66E+06	1.30	0.37	0.044037434
P46087	Probable 28S rRNA (cytosine(4447)-C(5))-methyltransferase OS=Homo sapiens OX=9606 GN=NOP2 PE=1 SV=2	9	14	5	7.31E+06	7.03E+06	5.33E+06	5.02E+06	3.93E+06	7.82E+06	7.97E+06	7.34E+06	3.93E+06	3.44E+06	4.11E+06	6.41E+06	6.07E+06	5.54E+06	1.10	0.13	0.607165816
P04040	Catalase OS=Homo sapiens OX=9606 GN=CAT PE=1 SV=3	14	18	5	5.16E+06	4.34E+06	4.65E+06	4.93E+06	4.55E+06	5.64E+06	6.97E+06	6.87E+06	1.00E+07	6.22E+06	7.38E+06	7.33E+06	4.88E+06	7.47E+06	0.65	-0.61	0.003562322
Q9NTJ3	Structural maintenance of chromosomes protein 4 OS=Homo sapiens OX=9606 GN=SMC4 PE=1 SV=2	4	14	5	4.84E+05	1.32E+06	3.60E+06	1.79E+06	1.07E+06	1.57E+06			7.95E+05	7.29E+05	7.80E+05	6.66E+05	1.64E+06	7.43E+05	2.21	1.14	0.093052534
Q04837	Single-stranded DNA-binding protein, mitochondrial OS=Homo sapiens OX=9606 GN=SSBP1 PE=1 SV=1	36	93	5	3.42E+07	3.68E+07	3.23E+07	3.45E+07	2.97E+07	3.24E+07	2.38E+07	2.66E+07	2.26E+07	2.28E+07	2.17E+07	2.06E+07	3.33E+07	2.30E+07	1.45	0.54	1.34645E-05
P05106	Integrin beta-3 OS=Homo sapiens OX=9606 GN=ITGB3 PE=1 SV=2	7	57	5	2.14E+07	1.09E+07	9.50E+06	1.16E+07	8.20E+06	2.04E+07	2.88E+07	1.35E+07	1.25E+07	1.17E+07	1.21E+07	2.48E+07	1.37E+07	1.72E+07	0.79	-0.33	0.381975866
P00749	Urokinase-type plasminogen activator OS=Homo sapiens OX=9606 GN=PLAU PE=1 SV=2	15	14	5	8.93E+06	8.22E+06	6.44E+06	7.28E+06	7.20E+06	7.57E+06	1.02E+07	1.11E+07	8.64E+06	9.66E+06	9.51E+06	8.39E+06	7.61E+06	9.59E+06	0.79	-0.33	0.004750868
P62829	60S ribosomal protein L23 OS=Homo sapiens OX=9606 GN=RPL23 PE=1 SV=1	39	60	5	2.23E+07	2.44E+07	2.13E+07	2.18E+07	1.96E+07	2.11E+07	1.50E+07	1.50E+07	1.24E+07	1.23E+07	8.00E+06	1.01E+07	2.18E+07	1.21E+07	1.79	0.84	7.8162E-05
Q86X55	Histone-arginine methyltransferase CARM1 OS=Homo sapiens OX=9606 GN=CARM1 PE=1 SV=3	11	147	5	3.95E+08	2.49E+08	3.85E+08	2.89E+08	2.64E+08	2.73E+08	4.10E+08	3.37E+08	2.33E+08	2.26E+08	2.19E+08	1.87E+08	3.09E+08	2.69E+08	1.15	0.20	0.381481246
O76021	Ribosomal L1 domain-containing protein 1 OS=Homo sapiens OX=9606 GN=RSL1D1 PE=1 SV=3	13	44	5	6.59E+06	6.39E+06	6.31E+06	6.33E+06	7.31E+06	1.55E+07	5.28E+06	4.69E+06	4.02E+06	4.83E+06	5.69E+06	5.76E+06	8.08E+06	5.04E+06	1.60	0.68	0.099416429
P78417	Glutathione S-transferase omega-1 OS=Homo sapiens OX=9606 GN=GSTO1 PE=1 SV=2	21	54	5	1.50E+07	1.73E+07	1.59E+07	1.69E+07	1.59E+07	1.54E+07	1.53E+07	1.63E+07	1.44E+07	1.36E+07	1.30E+07	1.29E+07	1.61E+07	1.42E+07	1.13	0.17	0.022482459
Q6ZNJ1	Neurobeachin-like protein 2 OS=Homo sapiens OX=9606 GN=NBEAL2 PE=1 SV=2	3	9	5	2.84E+06	2.33E+06	8.23E+06	9.92E+06	1.06E+07	2.30E+06	4.91E+06	1.72E+07	1.07E+07	8.89E+06	1.05E+07	8.69E+06	6.04E+06	1.01E+07	0.60	-0.75	0.106168347
O94874	E3 UFM1-protein ligase 1 OS=Homo sapiens OX=9606 GN=UFL1 PE=1 SV=2	8	27	5	3.37E+06	4.62E+06	3.90E+06	4.11E+06	3.47E+06	3.84E+06	1.30E+06	1.98E+06	1.95E+06	2.84E+06	1.74E+06	1.26E+06	3.88E+06	1.84E+06	2.11	1.08	6.06823E-05
Q14683	Structural maintenance of chromosomes protein 1A OS=Homo sapiens OX=9606 GN=SMC1A PE=1 SV=2	6	23	5	1.81E+08	1.64E+08	6.37E+07	6.73E+07	7.03E+07	8.31E+07	9.49E+07	1.13E+08	3.29E+07	8.94E+07	6.14E+07	7.39E+07	1.05E+08	7.75E+07	1.35	0.44	0.297287302
Q9Y2J2	Band 4.1-like protein 3 OS=Homo sapiens OX=9606 GN=EPB41L3 PE=1 SV=2	5	19	5	1.56E+07	1.61E+07	1.48E+07	1.76E+07	2.25E+07	2.24E+07	2.09E+07	2.01E+07	2.13E+07	1.87E+07	2.16E+07	2.36E+07	1.82E+07	2.10E+07	0.86	-0.21	0.109082343
P07437	Tubulin beta chain OS=Homo sapiens OX=9606 GN=TUBB PE=1 SV=2	81	5407	6	3.48E+08	3.53E+08	3.01E+08	2.74E+08	2.69E+08	3.84E+08	2.53E+08	2.71E+08	2.04E+08	2.24E+08	2.43E+08	2.67E+08	3.21E+08	2.44E+08	1.32	0.40	0.007631842
P68032	Actin, alpha cardiac muscle 1 OS=Homo sapiens OX=9606 GN=ACTC1 PE=1 SV=1	58	2921	6	9.37E+07	8.82E+07	2.05E+08	1.16E+08	1.56E+08	9.49E+07	3.79E+08	1.01E+08	1.36E+08	1.03E+08	1.26E+08	1.04E+08	1.26E+08	1.58E+08	0.79	-0.33	0.52182273
Q9NZN3	EH domain-containing protein 3 OS=Homo sapiens OX=9606 GN=EHD3 PE=1 SV=2	42	307	6	1.79E+07	1.54E+07	1.23E+07	1.57E+07	1.55E+07	1.67E+07	2.47E+07	2.46E+07	1.68E+07	1.84E+07	1.92E+07	1.81E+07	1.56E+07	2.03E+07	0.77	-0.38	0.019071455
P11217	Glycogen phosphorylase, muscle form OS=Homo sapiens OX=9606 GN=PYGM PE=1 SV=6	27	374	6	8.94E+06	1.01E+07	7.51E+06	7.45E+06	8.65E+06	7.27E+06	9.64E+06	1.08E+07	1.09E+07	1.05E+07	9.59E+06	1.06E+07	8.32E+06	1.04E+07	0.80	-0.32	0.004743457
Q9H4B7	Tubulin beta-1 chain OS=Homo sapiens OX=9606 GN=TUBB1 PE=1 SV=1	25	1622	6	6.58E+07	7.48E+07	7.20E+07	5.71E+07	5.93E+07	8.79E+07	6.44E+07	1.08E+08	8.59E+07	8.66E+07	9.03E+07	8.90E+07	6.95E+07	8.74E+07	0.79	-0.33	0.035736857
P52597	Heterogeneous nuclear ribonucleoprotein F OS=Homo sapiens OX=9606 GN=HNRNPF PE=1 SV=3	28	134	6	4.94E+07	4.04E+07	4.15E+07	4.41E+07	4.16E+07	4.26E+07	3.03E+07	2.67E+07	2.25E+07	2.59E+07	2.31E+07	2.20E+07	4.33E+07	2.51E+07	1.72	0.78	2.02455E-06
P17096	High mobility group protein HMG-I/HMG-Y OS=Homo sapiens OX=9606 GN=HMGA1 PE=1 SV=3	41	293	6	2.09E+08	2.12E+08	1.89E+08	2.16E+08	1.77E+08	2.00E+08	1.43E+08	1.32E+08	1.21E+08	1.42E+08	1.08E+08	1.09E+08	2.00E+08	1.26E+08	1.59	0.67	7.659E-06
P46782	40S ribosomal protein S5 OS=Homo sapiens OX=9606 GN=RPS5 PE=1 SV=4	43	85	6	2.11E+07	1.79E+07	1.80E+07	1.60E+07	1.94E+07	2.24E+07	1.71E+07	2.08E+07	1.37E+07	1.28E+07	1.59E+07	1.41E+07	1.91E+07	1.57E+07	1.22	0.28	0.053631251
P38919	Eukaryotic initiation factor 4A-III OS=Homo sapiens OX=9606 GN=EIF4A3 PE=1 SV=4	23	178	6	2.45E+07	2.80E+07	2.74E+07	2.92E+07	2.38E+07	2.37E+07	1.88E+07	2.30E+07	1.80E+07	1.95E+07	1.81E+07	2.04E+07	2.61E+07	1.96E+07	1.33	0.41	0.000477859
O95782	AP-2 complex subunit alpha-1 OS=Homo sapiens OX=9606 GN=AP2A1 PE=1 SV=3	18	81	6	1.08E+07	1.18E+07	1.09E+07	1.16E+07	1.23E+07	8.36E+06	8.85E+06	8.23E+06	8.22E+06	8.51E+06	8.29E+06	8.34E+06	1.09E+07	8.41E+06	1.30	0.38	0.005996316

Supplementary Table 4. List of all proteins identified by label free proteomics analysis.

Accession	Description	Coverage [%]	# PSMs	# Unique Peptides	Amitriptyline 1	Amitriptyline 2	Amitriptyline 3	Amitriptyline 4	Amitriptyline 5	Amitriptyline 6	Vehicle 1	Vehicle 2	Vehicle 3	Vehicle 4	Vehicle 5	Vehicle 6	Average amitriptyline	Average vehicle	Ratio amitriptyline/ vehicle	Log 2 fold change (amitriptyline /vehicle)	P-value
P09651	Heterogeneous nuclear ribonucleoprotein A1 OS=Homo sapiens OX=9606 GN=HNRNPA1 PE=1 SV=5	23	98	6	1.76E+07	2.55E+07	1.97E+07	2.18E+07	1.83E+07	2.23E+07	1.45E+07	1.45E+07	1.12E+07	1.13E+07	1.17E+07	9.34E+06	2.09E+07	1.21E+07	1.73	0.79	0.000206591
P07858	Cathepsin B OS=Homo sapiens OX=9606 GN=CTSB PE=1 SV=3	24	92	6	2.81E+07	2.12E+07	2.62E+07	2.42E+07	2.77E+07	2.60E+07	1.45E+07	1.37E+07	1.13E+07	1.15E+07	1.21E+07	1.06E+07	2.56E+07	1.23E+07	2.08	1.06	4.00638E-06
Q12904	Aminoacyl tRNA synthase complex-interacting multifunctional protein 1 OS=Homo sapiens OX=9606 GN=AIMP1 PE=1 SV=2	25	72	6	2.07E+07	1.86E+07	1.56E+07	1.89E+07	1.63E+07	1.60E+07	9.11E+06	8.70E+06	8.62E+06	9.56E+06	1.06E+07	7.70E+06	1.77E+07	9.05E+06	1.96	0.97	2.65149E-05
Q15555	Microtubule-associated protein RP/EB family member 2 OS=Homo sapiens OX=9606 GN=MAPRE2 PE=1 SV=1	25	123	6	1.68E+07	1.75E+07	1.89E+07	1.87E+07	1.58E+07	1.95E+07	2.30E+07	2.80E+07	2.61E+07	1.98E+07	1.81E+07	2.10E+07	1.79E+07	2.27E+07	0.79	-0.34	0.0257944
Q99714	3-hydroxyacyl-CoA dehydrogenase type-2 OS=Homo sapiens OX=9606 GN=HSD17B10 PE=1 SV=3	44	66	6	1.76E+07	1.70E+07	1.98E+07	1.21E+07	1.21E+07	1.57E+07	1.41E+07	1.19E+07	1.03E+07	8.00E+06	1.03E+07	1.01E+07	1.57E+07	1.08E+07	1.46	0.54	0.011127501
Q9Y2X3	Nucleolar protein 58 OS=Homo sapiens OX=9606 GN=NOP58 PE=1 SV=1	16	96	6	1.32E+07	1.45E+07	1.91E+07	1.66E+07	2.05E+07	1.67E+07	9.61E+06	8.58E+06	1.05E+07	9.78E+06	1.15E+07	8.40E+06	1.68E+07	9.72E+06	1.73	0.79	0.000710381
P30419	Glycylpeptide N-tetradecanoyltransferase 1 OS=Homo sapiens OX=9606 GN=NMT1 PE=1 SV=2	23	92	6	3.09E+07	2.94E+07	2.74E+07	2.53E+07	2.73E+07	2.67E+07	2.32E+07	1.97E+07	2.32E+07	2.30E+07	1.94E+07	1.73E+07	2.78E+07	2.10E+07	1.33	0.41	0.00045387
P01857	Immunoglobulin heavy constant gamma 1 OS=Homo sapiens OX=9606 GN=IGHG1 PE=1 SV=1	38	33	6	1.12E+07	1.57E+07	2.12E+07	2.34E+07	2.25E+07	2.19E+07	3.45E+07	1.78E+07	2.08E+07	1.98E+07	2.03E+07	1.07E+08	1.93E+07	3.68E+07	0.53	-0.93	0.280131754
P18669	Phosphoglycerate mutase 1 OS=Homo sapiens OX=9606 GN=PGAM1 PE=1 SV=2	37	125	6	4.05E+07	3.85E+07	2.86E+07	3.69E+07	3.00E+07	3.15E+07	3.95E+07	3.70E+07	2.89E+07	3.37E+07	3.36E+07	3.18E+07	3.43E+07	3.41E+07	1.01	0.01	0.92093334
P04745	Alpha-amylase 1 OS=Homo sapiens OX=9606 GN=AMY1A PE=1 SV=2	12	304	6	1.38E+08	1.43E+08	1.24E+08	1.37E+08	1.25E+08	1.18E+08	1.64E+08	1.49E+08	1.39E+08	1.58E+08	1.24E+08	1.33E+08	1.31E+08	1.44E+08	0.91	-0.14	0.100332006
P31146	Coronin-1A OS=Homo sapiens OX=9606 GN=CORO1A PE=1 SV=4	21	135	6	2.56E+07	2.32E+07	2.21E+07	2.74E+07	2.44E+07	2.36E+07	3.64E+07	3.98E+07	2.85E+07	3.05E+07	2.62E+07	3.04E+07	2.44E+07	3.20E+07	0.76	-0.39	0.013136093
P35613	Basigin OS=Homo sapiens OX=9606 GN=BSG PE=1 SV=2	21	131	6	5.11E+07	5.84E+07	5.56E+07	5.55E+07	5.05E+07	5.36E+07	2.78E+07	3.20E+07	3.03E+07	3.34E+07	2.75E+07	2.47E+07	5.41E+07	2.93E+07	1.85	0.88	8.32458E-08
P08243	Asparagine synthetase [glutamine-hydrolyzing] OS=Homo sapiens OX=9606 GN=ASNS PE=1 SV=4	18	52	6	9.54E+06	7.80E+06	9.53E+06	1.03E+07	6.90E+06	7.59E+06	6.53E+06	7.28E+06	6.34E+06	7.06E+06	6.78E+06	6.90E+06	8.61E+06	6.81E+06	1.26	0.34	0.02148764
O60749	Sorting nexin-2 OS=Homo sapiens OX=9606 GN=SNX2 PE=1 SV=2	20	131	6	2.13E+07	2.37E+07	2.27E+07	2.30E+07	2.12E+07	1.97E+07	2.27E+07	2.25E+07	2.10E+07	2.05E+07	2.18E+07	1.57E+07	2.19E+07	2.07E+07	1.06	0.08	0.34619375
Q93099	Homogentisate 1,2-dioxygenase OS=Homo sapiens OX=9606 GN=HGD PE=1 SV=2	19	158	6	1.75E+07	1.93E+07	2.31E+07	1.62E+07	2.53E+07	1.65E+07	2.50E+07	2.58E+07	2.19E+07	2.40E+07	2.30E+07	1.98E+07	1.97E+07	2.32E+07	0.85	-0.24	0.077537453
P27635	60S ribosomal protein L10 OS=Homo sapiens OX=9606 GN=RPL10 PE=1 SV=4	31	101	6	5.33E+07	4.82E+07	4.88E+07	5.54E+07	4.78E+07	4.54E+07	2.62E+07	2.29E+07	2.77E+07	3.15E+07	2.17E+07	1.91E+07	4.98E+07	2.48E+07	2.01	1.00	1.34317E-06
P84103	Serine/arginine-rich splicing factor 3 OS=Homo sapiens OX=9606 GN=SRSF3 PE=1 SV=1	41	152	6	4.95E+07	4.40E+07	3.66E+07	4.11E+07	4.37E+07	3.71E+07	3.21E+07	2.95E+07	2.70E+07	3.03E+07	2.61E+07	2.32E+07	4.20E+07	2.80E+07	1.50	0.58	0.000270987
Q7L2H7	Eukaryotic translation initiation factor 3 subunit M OS=Homo sapiens OX=9606 GN=EIF3M PE=1 SV=1	24	28	6	9.52E+06	8.40E+06	5.87E+06	5.96E+06	3.78E+06	5.44E+06	3.09E+06	3.76E+06	2.18E+05	1.39E+06	2.54E+06	1.33E+06	6.50E+06	2.05E+06	3.16	1.66	0.002022277
P31689	DnaJ homolog subfamily A member 1 OS=Homo sapiens OX=9606 GN=DNAJA1 PE=1 SV=2	23	55	6	1.21E+07	1.75E+07	1.47E+07	1.60E+07	1.73E+07	1.65E+07	6.92E+06	7.20E+06	7.66E+06	8.71E+06	6.53E+06	7.15E+06	1.57E+07	7.36E+06	2.13	1.09	5.72054E-05
Q16394	Exostosin-1 OS=Homo sapiens OX=9606 GN=EXT1 PE=1 SV=2	11	116	6	1.95E+07	1.89E+07	1.44E+07	1.38E+07	1.18E+07	1.75E+07	2.58E+07	2.82E+07	1.96E+07	1.61E+07	2.11E+07	2.37E+07	1.60E+07	2.24E+07	0.71	-0.49	0.016203075
P49721	Proteasome subunit beta type-2 OS=Homo sapiens OX=9606 GN=PSMB2 PE=1 SV=1	46	75	6	2.10E+07	2.27E+07	1.97E+07	1.81E+07	1.79E+07	2.01E+07	2.06E+07	2.28E+07	1.84E+07	1.55E+07	2.10E+07	1.58E+07	1.99E+07	1.90E+07	1.05	0.07	0.54208023
Q9UL46	Proteasome activator complex subunit 2 OS=Homo sapiens OX=9606 GN=PSME2 PE=1 SV=4	35	28	6	7.37E+06	4.87E+06	4.62E+06	7.52E+06	6.49E+06	5.52E+06	4.52E+06	3.71E+06	2.78E+06	3.65E+06	4.86E+06	3.40E+06	6.06E+06	3.82E+06	1.59	0.67	0.005264919
P52209	6-phosphogluconate dehydrogenase, decarboxylating OS=Homo sapiens OX=9606 GN=PGD PE=1 SV=3	20	120	6	4.99E+07	5.13E+07	5.08E+07	5.75E+07	5.54E+07	4.96E+07	6.58E+07	6.37E+07	7.60E+07	8.16E+07	5.83E+07	5.44E+07	5.24E+07	6.66E+07	0.79	-0.35	0.018681226
P30566	Adenylosuccinate lyase OS=Homo sapiens OX=9606 GN=ADSL PE=1 SV=2	23	71	6	2.29E+07	2.59E+07	2.34E+07	2.17E+07	2.08E+07	2.37E+07	2.74E+07	2.63E+07	2.20E+07	2.36E+07	2.41E+07	1.69E+07	2.31E+07	2.34E+07	0.99	-0.02	0.845010626
Q15459	Splicing factor 3A subunit 1 OS=Homo sapiens OX=9606 GN=SF3A1 PE=1 SV=1	14	35	6	8.78E+06	5.91E+06	7.94E+06	6.98E+06	6.81E+06	7.93E+06	4.34E+06	4.82E+06	6.06E+06	3.75E+06	4.32E+06	1.34E+06	7.39E+06	4.10E+06	1.80	0.85	0.002164005
P15559	NAD(P)H dehydrogenase [quinone] 1 OS=Homo sapiens OX=9606 GN=NQO1 PE=1 SV=1	31	57	6	2.66E+07	2.97E+07	3.93E+07	4.00E+07	3.68E+07	3.27E+07	2.74E+07	2.49E+07	3.00E+07	1.98E+07	1.69E+07	1.64E+07	3.42E+07	2.26E+07	1.51	0.60	0.004792335
Q9UHD8	Septin-9 OS=Homo sapiens OX=9606 GN=SEPTIN9 PE=1 SV=2	13	62	6	1.83E+07	2.21E+07	1.99E+07	2.03E+07	1.79E+07	1.80E+07	2.03E+07	1.54E+07	1.66E+07	1.70E+07	1.53E+07	1.27E+07	1.94E+07	1.62E+07	1.20	0.26	0.027412474
Q99536	Synaptic vesicle membrane protein VAT-1 homolog OS=Homo sapiens OX=9606 GN=VAT1 PE=1 SV=2	30	65	6	1.42E+07	1.51E+07	1.92E+07	1.47E+07	1.36E+07	1.73E+07	1.53E+07	1.49E+07	9.02E+06	1.78E+07	1.68E+07	1.40E+07	1.57E+07	1.46E+07	1.07	0.10	0.503314891
Q92499	ATP-dependent RNA helicase DDX1 OS=Homo sapiens OX=9606 GN=DDX1 PE=1 SV=2	10	43	6	7.75E+06	7.82E+06	9.85E+06	8.70E+06	9.69E+06	7.80E+06	6.82E+06	5.74E+06	6.85E+06	7.58E+06	6.76E+06	7.25E+06	8.60E+06	6.84E+06	1.26	0.33	0.005145748
P08174	Complement decay-accelerating factor OS=Homo sapiens OX=9606 GN=CD55 PE=1 SV=4	20	76	6	2.20E+07	1.70E+07	1.96E+07	1.78E+07	2.24E+07	1.69E+07	8.18E+06	7.82E+06	8.13E+06	6.92E+06	7.10E+06	6.14E+06	1.93E+07	7.38E+06	2.61	1.38	2.79711E-05
Q1KMD3	Heterogeneous nuclear ribonucleoprotein U-like protein 2 OS=Homo sapiens OX=9606 GN=HNRNPUL2 PE=1 SV=1	11	66	6	2.15E+07	2.07E+07	2.43E+07	1.80E+07	2.26E+07	2.01E+07	1.82E+07	2.04E+07	1.71E+07	2.28E+07	2.01E+07	1.56E+07	2.12E+07	1.90E+07	1.11	0.16	0.145696718
P46776	60S ribosomal protein L27a OS=Homo sapiens OX=9606 GN=RPL27A PE=1 SV=2	31	117	6	4.46E+07	4.34E+07	4.90E+07	4.00E+07	3.82E+07	3.96E+07	3.00E+07	2.88E+07	2.53E+07	2.32E+07	2.60E+07	2.12E+07	4.25E+07	2.57E+07	1.65	0.72	1.6396E-05
Q92743	Serine protease HTRA1 OS=Homo sapiens OX=9606 GN=HTRA1 PE=1 SV=1	15	66	6	5.77E+06	1.22E+07	9.57E+06	1.27E+07	1.01E+07	1.15E+07	1.78E+07	2.12E+07	1.54E+07	1.47E+07	1.74E+07	1.85E+07	1.03E+07	1.75E+07	0.59	-0.76	0.000459262
P25786	Proteasome subunit alpha type-1 OS=Homo sapiens OX=9606 GN=PSMA1 PE=1 SV=1	22	127	6	4.76E+07	4.74E+07	4.43E+07	3.88E+07	4.41E+07	3.70E+07	3.91E+07	4.90E+07	4.45E+07	4.22E+07	3.83E+07	3.15E+07	4.32E+07	4.08E+07	1.06	0.08	0.442285012
P49419	Alpha-aminoadipic semialdehyde dehydrogenase OS=Homo sapiens OX=9606 GN=ALDH7A1 PE=1 SV=5	17	72	6	1.41E+07	1.78E+07	1.34E+07	1.23E+07	1.25E+07	1.30E+07	1.59E+07	1.43E+07	1.12E+07	1.15E+07	1.10E+07	8.97E+06	1.38E+07	1.21E+07	1.14	0.19	0.227588154
Q92598	Heat shock protein 105 kDa OS=Homo sapiens OX=9606 GN=HSPH1 PE=1 SV=1	8	50	6	1.54E+07	1.46E+07	1.24E+07	1.23E+07	1.27E+07	1.18E+07	1.24E+07	9.98E+06	9.11E+06	1.10E+07	7.68E+06	6.71E+06	1.32E+07	9.48E+06	1.39	0.48	0.0062372
O43684	Mitotic checkpoint protein BUB3 OS=Homo sapiens OX=9606 GN=BUB3 PE=1 SV=1	21	87	6	1.86E+07	2.10E+07	2.21E+07	2.12E+07	2.19E+07	1.88E+07	1.61E+07	1.75E+07	1.66E+07	1.65E+07	1.53E+07	1.36E+07	2.06E+07	1.59E+07	1.29	0.37	0.000247396
P49736	DNA replication licensing factor MCM2 OS=Homo sapiens OX=9606 GN=MCM2 PE=1 SV=4	9	56	6	1.31E+07	1.43E+07	1.69E+07	1.33E+07	1.60E+07	1.38E+07	9.81E+06	9.82E+06	8.69E+06	8.93E+06	9.03E+06	7.76E+06	1.46E+07	9.01E+06	1.62	0.69	7.07947E-05
Q9H4A4	Aminopeptidase B OS=Homo sapiens OX=9606 GN=RNPEP PE=1 SV=2	12	40	6	1.39E+07	1.29E+07	1.26E+07	1.36E+07	1.25E+07	1.35E+07	8.93E+06	1.15E+07	4.36E+06	3.67E+06	4.94E+06	6.92E+06	1.32E+07	6.71E+06	1.96	0.97	0.002910677
Q9NVA2	Septin-11 OS=Homo sapiens OX=9606 GN=SEPTIN11 PE=1 SV=3	21	129	6	3.29E+07	3.33E+07	2.63E+07	2.76E+07	2.24E+07	2.42E+07	2.91E+07	2.87E+07	1.98E+07	1.91E+07	1.94E+07	2.20E+07	2.78E+07	2.30E+07	1.21	0.27	0.099519283
P23246	Splicing factor, proline- and glutamine-rich OS=Homo sapiens OX=9606 GN=SFPQ PE=1 SV=2	12	81	6	2.68E+07	2.37E+07	3.41E+07	2.87E+07	3.32E+07	2.77E+07	2.03E+07	2.21E+07	1.95E+07	1.94E+07	1.84E+07	1.63E+07	2.91E+07	1.93E+07	1.50	0.59	0.000880256
Q12874	Splicing factor 3A subunit 3 OS=Homo sapiens OX=9606 GN=SF3A3 PE=1 SV=1	17	44	6	1.28E+07	1.25E+07	1.14E+07	1.23E+07	1.26E+07	1.15E+07	8.11E+06	7.72E+06	7.08E+06	9.39E+06	7.56E+06	6.80E+06	1.22E+07	7.77E+06	1.57	0.65	5.90732E-06
P13674	Prolyl 4-hydroxylase subunit alpha-1 OS=Homo sapiens OX=9606 GN=P4HA1 PE=1 SV=2	17	60	6	7.72E+06	8.28E+06	6.85E+06	4.89E+06	4.89E+06	5.87E+06	8.82E+06	7.62E+06	6.69E+06	7.83E+06	7.50E+06	6.00E+06	6.42E+06	7.41E+06	0.87	-0.21	0.195447961
P09871	Complement C1s subcomponent OS=Homo sapiens OX=9606 GN=C1S PE=1 SV=1	11	13	6	5.76E+05						8.86E+05					1.88E+07	5.76E+05	9.86E+06	0.06	-4.10	#DIV/0!
Q13435	Splicing factor 3B subunit 2 OS=Homo sapiens OX=9606 GN=SF3B2 PE=1 SV=2	7	44	6	1.58E+07	1.47E+07	1.68E+07	1.44E+07	2.00E+07	1.64E+07	7.08E+06	9.87E+06	1.29E+07	2.82E+07	7.94E+06	6.02E+06	1.63E+07	1.20E+07	1.36	0.45	0.262811855
O15372	Eukaryotic translation initiation factor 3 subunit H OS=Homo sapiens OX=9606 GN=EIF3H PE=1 SV=1	24	73	6	2.12E+07	1.88E+07	2.90E+07	2.79E+07	2.95E+07	2.37E+07	1.66E+07	1.75E+07	1.88E+07	1.53E+07	1.78E+07	1.32E+07	2.50E+07	1.65E+07	1.51	0.60	0.003848281
P62263	40S ribosomal protein S14 OS=Homo sapiens OX=9606 GN=RPS14 PE=1 SV=3	39	72	6	3.80E+07	2.58E+07	2.70E+07	2.61E+07	2.73E+07	2.49E+07	1.62E+07	1.65E+07	2.15E+07	1.78E+07	1.57E+07	1.24E+07	2.82E+07	1.67E+07	1.69	0.76	0.001061454
Q14517	Protocadherin Fat 1 OS=Homo sapiens OX=9606 GN=FAT1 PE=1 SV=2	2	46	6	1.31E+07	1.45E+07	1.54E+07	1.25E+07	1.14E+07	1.40E+07	1.48E+07	7.13E+06	7.92E+06	1.27E+07	1.40E+07	1.14E+07	1.35E+07	1.13E+07	1.19	0.25	0.167339673
Q9UBG0	C-type mannose receptor 2 OS=Homo sapiens OX=9606 GN=MRC2 PE=1 SV=2	6	61	6	1.47E+07	1.32E+07	1.19E+07	1.39E+07	1.37E+07	1.37E+07	1.71E+07	1.67E+07	1.16E+07	1.48E+07	1.33E+07	1.23E+07	1.35E+07	1.43E+07	0.94	-0.08	0.46326587
Q99733	Nucleosome assembly protein 1-like 4 OS=Homo sapiens OX=9606 GN=NAP1L4 PE=1 SV=1	24	51	6	1.84E+07	1.84E+07	1.49E+07	1.42E+07	1.32E+07	1.54E+07	1.10E+07	7.33E+06	8.50E+06	6.57E+06	8.42E+06	7.27E+06	1.57E+07	8.18E+06	1.92	0.94	7.26046E-05
Q16851	UTP--glucose-1-phosphate uridylyltransferase OS=Homo sapiens OX=9606 GN=UGP2 PE=1 SV=5	16	36	6	8.19E+06	6.86E+06	6.14E+06	6.28E+06	4.99E+06	6.00E+06	1.00E+07	9.29E+06	8.24E+06	7.16E+06	7.37E+06	6.66E+06	6.41E+06	8.12E+06	0.79	-0.34	0.033310502
Q13683	Integrin alpha-7 OS=Homo sapiens OX=9606 GN=ITGA7 PE=1 SV=3	7	23	6	7.75E+06	8.52E+06	7.25E+06	8.76E+06	9.57E+06	6.80E+06	4.34E+06	5.19E+06	5.29E+06	4.20E+06	3.87E+06	2.95E+06	8.11E+06	4.31E+06	1.88	0.91	4.79915E-05
P11166	Solute carrier family 2, facilitated glucose transporter member 1 OS=Homo sapiens OX=9606 GN=SLC2A1 PE=1 SV=2	12	187	6	8.92E+07	6.32E+07	8.55E+07	6.35E+07	8.25E+07	7.13E+07	6.94E+07	6.55E+07	8.53E+07	5.97E+07	8.30E+07	6.68E+07	7.59E+07	7.16E+07	1.06	0.08	0.507956287
Q16222	UDP-N-acetylhexosamine pyrophosphorylase OS=Homo sapiens OX=9606 GN=UAP1 PE=1 SV=3	17	20	6	6.54E+06	6.43E+06	6.16E+06	4.99E+06	5.56E+06	5.04E+06	7.44E+06	6.76E+06	5.90E+06	3.17E+06	4.53E+06	4.35E+06	5.79E+06	5.36E+06	1.08	0.11	0.568264035
Q7Z6Z7	E3 ubiquitin-protein ligase HUWE1 OS=Homo sapiens OX=9606 GN=HUWE1 PE=1 SV=3	2	29	6	5.56E+06	4.88E+06	6.19E+06	5.88E+06	7.25E+06	5.81E+06	2.73E+06	4.26E+06	5.58E+06	4.87E+06	4.21E+06	1.09E+07	5.93E+06	5.43E+06	1.09	0.13	0.696891237
Q93034	Cullin-5 OS=Homo sapiens OX=9606 GN=CUL5 PE=1 SV=4	9	86	6	2.76E+08	2.68E+08	3.76E+08	4.26E+08	4.57E+08	4.00E+08	4.00E+08	4.29E+08	6.89E+08	8.41E+08	8.59E+08	6.44E+08	3.67E+08	6.44E+08	0.57	-0.81	0.016371436
Q8IWA5	Choline transporter-like protein 2 OS=Homo sapiens OX=9606 GN=SLC44A2 PE=1 SV=3	9	42	6	8.62E+06	8.41E+06	8.19E+06	1.51E+07	8.46E+06	7.89E+06	5.70E+06	5.34E+06	9.07E+06	8.18E+06	7.09E+06	5.02E+06	9.44E+06	6.73E+06	1.40	0.49	0.073208123
Q9BUF5	Tubulin beta-6 chain OS=Homo sapiens OX=9606 GN=TUBB6 PE=1 SV=1	60	2411	7	6.44E+07	4.91E+07	4.98E+07	4.22E+07	4.95E+07	4.27E+07	4.96E+07	4.88E+07	3.95E+07	3.66E+07	4.34E+07	4.43E+07	4.96E+07	4.37E+07	1.14	0.18	0.162762495
P61204	ADP-ribosylation factor 3 OS=Homo sapiens OX=9606 GN=ARF3 PE=1 SV=2	60	426	7	6.60E+07	8.32E+07	8.24E+07	8.45E+07	6.34E+07	8.96E+07	9.20E+07	1.04E+08	7.19E+07	6.10E+07	9.23E+07	7.52E+07	7.82E+07	8.27E+07	0.95	-0.08	0.581869184
P61163	Alpha-centractin OS=Homo sapiens OX=9606 GN=ACTR1A PE=1 SV=1	52	238	7	9.21E+07	8.38E+07	8.41E+07	8.55E+07	7.50E+07	8.56E+07	7.73E+07	7.98E+07	5.94E+07	6.45E+07	6.61E+07	6.40E+07	8.44E+07	6.85E+07	1.23	0.30	0.003470943
P15311	Ezrin OS=Homo sapiens OX=9606 GN=EZR PE=1 SV=4	26	288	7	2.53E+07	1.48E+07	2.03E+07	1.44E+07	1.65E+07	1.67E+07	2.35E+07	1.17E+07	1.76E+07	1.60E+07	1.40E+07	1.62E+07	1.80E+07	1.65E+07	1.09	0.13	0.530375089
P69905	Hemoglobin subunit alpha OS=Homo sapiens OX=9606 GN=HBA1 PE=1 SV=2	38	3552	7	4.25E+09	4.41E+09	5.57E+09	5.43E+09	5.89E+09	4.83E+09	5.95E+09	5.61E+09	7.16E+09	6.91E+09	6.52E+09	6.70E+09	5.06E+09	6.47E+09	0.78	-0.36	0.003064574
P67809	Y-box-binding protein 1 OS=Homo sapiens OX=9606 GN=YBX1 PE=1 SV=3	34	118	7	2.10E+07	2.50E+07	2.39E+07	2.50E+07	2.24E+07	2.62E+07	2.41E+07	2.70E+07	2.33E+07	2.64E+07	2.46E+07	1.96E+07	2.39E+07	2.42E+07	0.99	-0.01	0.863935241
P55209	Nucleosome assembly protein 1-like 1 OS=Homo sapiens OX=9606 GN=NAP1L1 PE=1 SV=1	36	142	7	3.19E+07	3.15E+07	3.25E+07	2.79E+07	2.60E+07	2.92E+07	2.32E+07	1.95E+07	1.65E+07	1.69E+07	1.80E+07	1.85E+07	2.98E+07	1.88E+07	1.59	0.67	1.72108E-05
P51884	Lumican OS=Homo sapiens OX=9606 GN=LUM PE=1 SV=2	14	923	7	5.41E+08	5.37E+08	7.21E+08	5.90E+08	7.00E+08	6.26E+08	5.77E+08	6.52E+08	8.30E+08	7.90E+08	9.92E+08	7.55E+08	6.19E+08	7.66E+08	0.81	-0.31	0.061402872
P29992	Guanine nucleotide-binding protein subunit alpha-11 OS=Homo sapiens OX=9606 GN=GNA11 PE=1 SV=2	33	141	7	4.22E+07	3.71E+07	3.72E+07	3.75E+07	3.46E+07	3.91E+07	3.01E+07	2.91E+07	2.51E+07	2.71E+07	2.35E+07	2.52E+07	3.79E+07	2.67E+07	1.42	0.51	1.79168E-05
P62979	Ubiquitin-40S ribosomal protein S27a OS=Homo sapiens OX=9606 GN=RPS27A PE=1 SV=2	44	732	7	3.76E+08	5.17E+08	4.74E+08	4.74E+08	4.48E+08	4.33E+08	2.69E+08	2.93E+08	3.61E+08	3.33E+08	3.24E+08	2.65E+08	4.54E+08	3.08E+08	1.47	0.56	0.000186257
P60953	Cell division control protein 42 homolog OS=Homo sapiens OX=9606 GN=CDC42 PE=1 SV=2	47	118	7	3.86E+07	3.29E+07	3.40E+07	2.59E+07	2.64E+07	3.38E+07	3.29E+07	3.44E+07	2.51E+07	2.44E+07	2.82E+07	2.73E+07	3.19E+07	2.87E+07	1.11	0.15	0.245962254
Q06033	Inter-alpha-trypsin inhibitor heavy chain H3 OS=Homo sapiens OX=9606 GN=ITIH3 PE=1 SV=2	6	320	7	8.87E+07	1.34E+08	8.37E+07	9.72E+07	8.58E+07	1.04E+08	1.13E+08	1.82E+08	1.13E+08	1.04E+08	1.09E+08	1.25E+08	9.89E+07	1.24E+08	0.80	-0.33	0.107814671
Q96P70	Importin-9 OS=Homo sapiens OX=9606 GN=IPO9 PE=1 SV=3	12	72	7	1.52E+08	1.10E+08	1.34E+08	1.50E+08	1.37E+08	1.59E+07	1.58E+08	1.12E+08	1.06E+08	8.80E+07	1.36E+08	1.41E+08	1.17E+08	1.24E+08	0.94	-0.08	0.774235564
Q15758	Neutral amino acid transporter B(0) OS=Homo sapiens OX=9606 GN=SLC1A5 PE=1 SV=2	22	148	7	8.06E+07	6.87E+07	7.93E+07	6.85E+07	6.46E+07	9.39E+07	5.88E+07	4.92E+07	4.55E+07	4.17E+07	5.06E+07	4.38E+07	7.59E+07	4.83E+07	1.57	0.65	0.000657092
P07305	Histone H1.0 OS=Homo sapiens OX=9606 GN=H1-0 PE=1 SV=3	24	907	7	2.67E+08	3.26E+08	2.72E+08	3.12E+08	2.62E+08	2.96E+08	2.60E+08	3.13E+08	2.60E+08	2.73E+08	2.59E+08	2.51E+08	2.89E+08	2.69E+08	1.07	0.10	0.195341041
P12109	Collagen alpha-1(VI) chain OS=Homo sapiens OX=9606 GN=COL6A1 PE=1 SV=3	9	244	7	1.13E+08	1.23E+08	9.71E+07	1.10E+08	1.10E+08	9.07E+07	1.54E+08	1.61E+08	1.53E+08	1.44E+08	1.42E+08	1.43E+08	1.07E+08	1.50E+08	0.72	-0.48	4.92381E-05
P51665	26S proteasome non-ATPase regulatory subunit 7 OS=Homo sapiens OX=9606 GN=PSMD7 PE=1 SV=2	28	80	7	2.32E+07	2.99E+07	1.96E+07	2.09E+07	1.95E+07	2.42E+07	1.29E+07	1.94E+07	9.98E+06	1.06E+07	1.62E+07	1.30E+07	2.29E+07	1.37E+07	1.67	0.74	0.001720333
P04004	Vitronectin OS=Homo sapiens OX=9606 GN=VTN PE=1 SV=1	14	362	7	4.73E+08	3.08E+08	2.89E+08	3.47E+08	2.78E+08	3.32E+08	3.78E+08	2.76E+08	2.12E+08	2.46E+08	2.51E+08	2.83E+08	3.38E+08	2.74E+08	1.23	0.30	0.119987945
Q13501	Sequestosome-1 OS=Homo sapiens OX=9606 GN=SQSTM1 PE=1 SV=1	30	52	7	2.63E+07	2.28E+07	2.24E+07	2.59E+07	2.27E+07	2.87E+07	1.89E+06	2.80E+06	2.35E+06		5.79E+05		2.48E+07	1.91E+06	13.01	3.70	2.64697E-07
P02794	Ferritin heavy chain OS=Homo sapiens OX=9606 GN=FTH1 PE=1 SV=2	56	124	7	3.11E+07	3.84E+07	4.63E+07	4.85E+07	4.42E+07	4.17E+07	5.34E+07	5.38E+07	6.87E+07	6.62E+07	5.73E+07	5.33E+07	4.17E+07	5.88E+07	0.71	-0.50	0.00119783
Q9HBI1	Beta-parvin OS=Homo sapiens OX=9606 GN=PARVB PE=1 SV=1	22	127	7	2.56E+07	2.37E+07	2.59E+07	2.53E+07	2.58E+07	2.44E+07	3.55E+07	3.27E+07	3.31E+07	2.85E+07	3.04E+07	2.72E+07	2.51E+07	3.12E+07	0.80	-0.31	0.003782095
Q9NR45	Sialic acid synthase OS=Homo sapiens OX=9606 GN=NANS PE=1 SV=2	35	134	7	4.37E+07	4.82E+07	3.69E+07	3.73E+07	3.26E+07	3.82E+07	3.31E+07	4.37E+07	3.20E+07	2.79E+07	3.34E+07	2.59E+07	3.95E+07	3.27E+07	1.21	0.27	0.072020388
Q13813	Spectrin alpha chain, non-erythrocytic 1 OS=Homo sapiens OX=9606 GN=SPTAN1 PE=1 SV=3	4	63	7	7.78E+06	1.14E+07	9.48E+06	1.11E+07	8.82E+06	1.00E+07	6.43E+06	6.21E+06	6.19E+06	5.88E+06	6.22E+06	4.38E+06	9.77E+06	5.88E+06	1.66	0.73	0.000340235
P49747	Cartilage oligomeric matrix protein OS=Homo sapiens OX=9606 GN=COMP PE=1 SV=2	18	211	7	8.29E+07	8.67E+07	8.78E+07	9.13E+07	8.82E+07	8.13E+07	1.16E+08	1.28E+08	1.07E+08	1.11E+08	1.06E+08	9.83E+07	8.63E+07	1.11E+08	0.78	-0.36	0.001160575
O43505	Beta-1,4-glucuronyltransferase 1 OS=Homo sapiens OX=9606 GN=B4GAT1 PE=1 SV=1	25	124	7	3.48E+07	3.31E+07	2.68E+07	2.90E+07	2.98E+07	4.24E+07	5.31E+07	5.40E+07	5.23E+07	3.13E+07	3.89E+07	3.61E+07	3.27E+07	4.43E+07	0.74	-0.44	0.037966268
P62195	26S proteasome regulatory subunit 8 OS=Homo sapiens OX=9606 GN=PSMC5 PE=1 SV=1	27	133	7	2.47E+07	2.74E+07	2.06E+07	1.98E+07	2.09E+07	2.76E+07	1.83E+07	1.74E+07	1.50E+07	1.32E+07	1.69E+07	1.30E+07	2.35E+07	1.56E+07	1.50	0.59	0.001466562
Q13347	Eukaryotic translation initiation factor 3 subunit I OS=Homo sapiens OX=9606 GN=EIF3I PE=1 SV=1	37	92	7	2.65E+07	2.62E+07	2.40E+07	2.58E+07	2.26E+07	2.15E+07	1.74E+07	1.59E+07	1.50E+07	1.63E+07	1.50E+07	1.40E+07	2.44E+07	1.56E+07	1.56	0.65	1.8692E-05
Q14108	Lysosome membrane protein 2 OS=Homo sapiens OX=9606 GN=SCARB2 PE=1 SV=2	24	74	7	2.33E+07	2.90E+07	2.48E+07	2.64E+07	2.25E+07	2.31E+07	7.18E+06	8.13E+06	9.35E+06	1.03E+07	8.65E+06	9.93E+06	2.49E+07	8.93E+06	2.78	1.48	1.73165E-06
P62266	40S ribosomal protein S23 OS=Homo sapiens OX=9606 GN=RPS23 PE=1 SV=3	49	127	7	8.56E+07	7.89E+07	5.66E+07	7.36E+07	6.22E+07	5.39E+07	4.96E+07	5.10E+07	3.87E+07	5.01E+07	3.43E+07	3.34E+07	6.85E+07	4.29E+07	1.60	0.68	0.002918802
P14550	Aldo-keto reductase family 1 member A1 OS=Homo sapiens OX=9606 GN=AKR1A1 PE=1 SV=3	28	293	7	1.61E+08	1.78E+08	1.56E+08	2.13E+08	1.65E+08	1.94E+08	2.25E+08	2.42E+08	2.79E+08	2.99E+08	2.58E+08	2.03E+08	1.78E+08	2.51E+08	0.71	-0.50	0.002271934
P29692	Elongation factor 1-delta OS=Homo sapiens OX=9606 GN=EEF1D PE=1 SV=5	30	183	7	4.45E+07	5.20E+07	4.26E+07	4.71E+07	5.52E+07	4.31E+07	3.27E+07	4.11E+07	4.09E+07	3.40E+07	3.40E+07	3.28E+07	4.74E+07	3.59E+07	1.32	0.40	0.00172015
Q13155	Aminoacyl tRNA synthase complex-interacting multifunctional protein 2 OS=Homo sapiens OX=9606 GN=AIMP2 PE=1 SV=2	32	58	7	2.61E+07	2.24E+07	1.98E+07	1.87E+07	2.14E+07	2.38E+07	1.19E+07	1.07E+07	1.03E+07	1.05E+07	9.29E+06	9.44E+06	2.20E+07	1.04E+07	2.13	1.09	4.55228E-05
O75131	Copine-3 OS=Homo sapiens OX=9606 GN=CPNE3 PE=1 SV=1	19	132	7	3.23E+07	2.94E+07	2.88E+07	2.83E+07	3.04E+07	2.75E+07	1.87E+07	1.47E+07	1.40E+07	1.63E+07	1.83E+07	1.41E+07	2.94E+07	1.60E+07	1.84	0.88	3.93441E-07
P28074	Proteasome subunit beta type-5 OS=Homo sapiens OX=9606 GN=PSMB5 PE=1 SV=3	27	84	7	1.84E+07	2.00E+07	1.95E+07	2.05E+07	1.93E+07	1.57E+07	1.98E+07	1.88E+07	2.02E+07	1.87E+07	1.69E+07	1.49E+07	1.89E+07	1.82E+07	1.04	0.05	0.536343382
P16152	Carbonyl reductase [NADPH] 1 OS=Homo sapiens OX=9606 GN=CBR1 PE=1 SV=3	38	85	7	2.02E+07	2.17E+07	1.56E+07	1.73E+07	1.68E+07	1.65E+07	2.20E+07	2.08E+07	1.51E+07	1.74E+07	1.72E+07	1.65E+07	1.80E+07	1.82E+07	0.99	-0.01	0.918345136
P25787	Proteasome subunit alpha type-2 OS=Homo sapiens OX=9606 GN=PSMA2 PE=1 SV=2	35	106	7	3.09E+07	2.36E+07	2.35E+07	2.90E+07	2.29E+07	2.87E+07	3.31E+07	2.49E+07	2.23E+07	2.09E+07	2.40E+07	2.04E+07	2.64E+07	2.43E+07	1.09	0.12	0.386872424
P02649	Apolipoprotein E OS=Homo sapiens OX=9606 GN=APOE PE=1 SV=1	25	348	7	2.17E+08	2.16E+08	1.98E+08	2.01E+08	1.89E+08	1.87E+08	2.27E+08	2.32E+08	2.00E+08	2.19E+08	2.07E+08	2.15E+08	2.01E+08	2.17E+08	0.93	-0.11	0.055619113
P07910	Heterogeneous nuclear ribonucleoproteins C1/C2 OS=Homo sapiens OX=9606 GN=HNRNPC PE=1 SV=4	26	255	7	8.48E+07	9.15E+07	7.37E+07	9.42E+07	7.60E+07	7.29E+07	5.74E+07	4.89E+07	5.63E+07	5.68E+07	3.94E+07	4.69E+07	8.22E+07	5.10E+07	1.61	0.69	9.39164E-05
P32119	Peroxiredoxin-2 OS=Homo sapiens OX=9606 GN=PRDX2 PE=1 SV=5	34	134	7	4.30E+07	3.34E+07	3.70E+07	3.63E+07	2.72E+07	3.13E+07	3.00E+07	2.52E+07	2.24E+07	1.94E+07	1.87E+07	1.91E+07	3.47E+07	2.25E+07	1.54	0.63	0.001765888
P50454	Serpin H1 OS=Homo sapiens OX=9606 GN=SERPINH1 PE=1 SV=2	23	116	7	2.30E+07	2.66E+07	1.73E+07	2.30E+07	2.07E+07	2.16E+07	3.03E+07	3.04E+07	2.20E+07	2.28E+07	2.65E+07	1.77E+07	2.20E+07	2.50E+07	0.88	-0.18	0.25874207
O00154	Cytosolic acyl coenzyme A thioester hydrolase OS=Homo sapiens OX=9606 GN=ACOT7 PE=1 SV=3	24	66	7	2.76E+07	3.13E+07	1.84E+07	2.40E+07	2.50E+07	2.45E+07	2.40E+07	2.46E+07	1.61E+07	1.55E+07	1.63E+07	1.70E+07	2.51E+07	1.89E+07	1.33	0.41	0.029559103
P37802	Transgelin-2 OS=Homo sapiens OX=9606 GN=TAGLN2 PE=1 SV=3	50	114	7	3.21E+07	3.31E+07	3.11E+07	3.05E+07	2.34E+07	2.66E+07	3.05E+07	2.42E+07	2.14E+07	1.83E+07	2.01E+07	2.35E+07	2.95E+07	2.30E+07	1.28	0.36	0.019334721

Supplementary Table 4. List of all proteins identified by label free proteomics analysis.

Accession	Description	Coverage [%]	# PSMs	# Unique Peptides	Amitriptyline 1	Amitriptyline 2	Amitriptyline 3	Amitriptyline 4	Amitriptyline 5	Amitriptyline 6	Vehicle 1	Vehicle 2	Vehicle 3	Vehicle 4	Vehicle 5	Vehicle 6	Average amitriptyline	Average vehicle	Ratio amitriptyline/ vehicle	Log 2 fold change (amitriptyline /vehicle)	P-value
P00736	Complement C1r subcomponent OS=Homo sapiens OX=9606 GN=C1R PE=1 SV=2	14	43	7	5.75E+06	3.35E+06	3.62E+06	6.35E+06	5.03E+06	5.67E+06	8.38E+06	7.63E+06	9.83E+06	7.21E+06	6.76E+06	3.00E+07	4.96E+06	1.16E+07	0.43	-1.23	0.131891979
Q9NR30	Nucleolar RNA helicase 2 OS=Homo sapiens OX=9606 GN=DDX21 PE=1 SV=5	14	64	7	8.58E+06	9.13E+06	8.47E+06	7.28E+06	6.59E+06	7.86E+06	7.70E+06	6.80E+06	4.86E+06	3.92E+06	5.67E+06	5.53E+06	7.98E+06	5.75E+06	1.39	0.47	0.008790562
P32969	60S ribosomal protein L9 OS=Homo sapiens OX=9606 GN=RPL9 PE=1 SV=1	59	59	7	1.53E+07	2.64E+07	1.97E+07	1.74E+07	1.70E+07	2.45E+07	1.12E+07	2.33E+07	1.45E+07	1.46E+07	2.19E+07	1.63E+07	2.00E+07	1.70E+07	1.18	0.24	0.269518433
Q06830	Peroxiredoxin-1 OS=Homo sapiens OX=9606 GN=PRDX1 PE=1 SV=1	34	142	7	5.53E+07	5.20E+07	5.12E+07	4.76E+07	4.75E+07	4.87E+07	2.41E+07	2.74E+07	2.49E+07	2.41E+07	2.85E+07	1.68E+07	5.04E+07	2.43E+07	2.07	1.05	4.18809E-07
O15230	Laminin subunit alpha-5 OS=Homo sapiens OX=9606 GN=LAMA5 PE=1 SV=8	3	46	7	1.13E+07	5.86E+06	8.21E+06	8.33E+06	1.06E+07	8.31E+06	1.20E+07	9.05E+06	7.92E+06	9.33E+06	8.67E+06	6.30E+06	8.76E+06	8.87E+06	0.99	-0.02	0.920905658
O60488	Long-chain-fatty-acid--CoA ligase 4 OS=Homo sapiens OX=9606 GN=ACSL4 PE=1 SV=2	16	58	7	2.51E+07	1.92E+07	1.17E+07	1.24E+07	1.43E+07	1.38E+07	1.22E+07	1.29E+07	8.17E+06	1.08E+07	1.00E+07	1.00E+07	1.61E+07	1.07E+07	1.50	0.59	0.050392678
P14543	Nidogen-1 OS=Homo sapiens OX=9606 GN=NID1 PE=1 SV=3	8	82	7	1.56E+07	1.78E+07	1.53E+07	1.50E+07	1.39E+07	1.35E+07	2.08E+07	2.22E+07	1.69E+07	1.96E+07	1.61E+07	1.46E+07	1.52E+07	1.84E+07	0.83	-0.27	0.049726486
P61313	60S ribosomal protein L15 OS=Homo sapiens OX=9606 GN=RPL15 PE=1 SV=2	31	137	7	6.73E+07	7.21E+07	7.68E+07	7.45E+07	6.81E+07	6.44E+07	4.58E+07	4.50E+07	4.35E+07	4.63E+07	3.94E+07	3.56E+07	7.05E+07	4.26E+07	1.66	0.73	8.53724E-07
Q16666	Gamma-interferon-inducible protein 16 OS=Homo sapiens OX=9606 GN=IFI16 PE=1 SV=3	10	73	7	1.92E+07	1.85E+07	1.95E+07	2.25E+07	2.14E+07	2.00E+07	9.15E+06	7.06E+06	8.27E+06	8.06E+06	8.27E+06	5.68E+06	2.02E+07	7.75E+06	2.60	1.38	3.29988E-08
O75475	PC4 and SFRS1-interacting protein OS=Homo sapiens OX=9606 GN=PSIP1 PE=1 SV=1	17	65	7	1.43E+07	1.48E+07	1.44E+07	1.50E+07	1.34E+07	1.39E+07	5.63E+06	5.20E+06	4.91E+06	4.04E+06	3.67E+06	5.50E+06	1.43E+07	4.82E+06	2.96	1.57	2.05195E-09
P30101	Protein disulfide-isomerase A3 OS=Homo sapiens OX=9606 GN=PDIA3 PE=1 SV=4	14	124	7	2.60E+07	2.43E+07	2.41E+07	2.47E+07	2.78E+07	2.38E+07	2.85E+07	2.99E+07	2.83E+07	2.65E+07	2.52E+07	2.38E+07	2.51E+07	2.71E+07	0.93	-0.11	0.118676508
P30041	Peroxiredoxin-6 OS=Homo sapiens OX=9606 GN=PRDX6 PE=1 SV=3	38	71	7	1.63E+07	1.64E+07	1.75E+07	1.69E+07	1.76E+07	1.89E+07	1.59E+07	1.59E+07	1.45E+07	1.50E+07	1.45E+07	1.62E+07	1.72E+07	1.53E+07	1.13	0.17	0.003671198
P39748	Flap endonuclease 1 OS=Homo sapiens OX=9606 GN=FEN1 PE=1 SV=1	17	94	7	9.95E+07	1.00E+08	9.81E+07	1.04E+08	8.75E+07	9.39E+07	8.25E+07	8.12E+07	8.73E+07	8.85E+07	7.82E+07	6.97E+07	9.72E+07	8.12E+07	1.20	0.26	0.001486215
O94776	Metastasis-associated protein MTA2 OS=Homo sapiens OX=9606 GN=MTA2 PE=1 SV=1	12	28	7	1.29E+07	1.39E+07	1.22E+07	1.42E+07	1.08E+07	1.16E+07	9.80E+06	6.46E+06	8.68E+06	7.37E+06	5.13E+06	5.52E+06	1.26E+07	7.16E+06	1.76	0.82	0.00020902
Q9NZN4	EH domain-containing protein 2 OS=Homo sapiens OX=9606 GN=EHD2 PE=1 SV=2	18	132	7	1.48E+07	1.28E+07	1.26E+07	1.39E+07	1.23E+07	1.43E+07	1.46E+07	1.23E+07	1.21E+07	1.24E+07	1.17E+07	1.05E+07	1.34E+07	1.23E+07	1.10	0.13	0.119221272
Q9Y223	Bifunctional UDP-N-acetylglucosamine 2-epimerase/N-acetylmannosamine kinase OS=Homo sapiens OX=9606 GN=GNE PE=1 SV=1	12	68	7	1.28E+07	1.41E+07	1.11E+07	1.18E+07	1.22E+07	1.03E+07	1.41E+07	1.44E+07	1.24E+07	1.38E+07	1.17E+07	9.75E+06	1.21E+07	1.27E+07	0.95	-0.07	0.503888253
P27708	CAD protein OS=Homo sapiens OX=9606 GN=CAD PE=1 SV=3	4	36	7	7.57E+06	9.99E+06	1.19E+07	1.31E+07	1.09E+07	1.13E+07	7.77E+06	8.09E+06	8.37E+06	6.71E+06	8.07E+06	7.63E+06	1.08E+07	7.77E+06	1.39	0.48	0.00991683
O43143	Pre-mRNA-splicing factor ATP-dependent RNA helicase DHX15 OS=Homo sapiens OX=9606 GN=DHX15 PE=1 SV=2	10	48	7	1.08E+07	1.34E+07	1.35E+07	1.71E+07	1.24E+07	1.30E+07	1.20E+07	1.27E+07	1.30E+07	1.19E+07	1.10E+07	7.52E+06	1.34E+07	1.13E+07	1.18	0.24	0.11746924
Q9Y2A7	Nck-associated protein 1 OS=Homo sapiens OX=9606 GN=NCKAP1 PE=1 SV=1	6	83	7	1.05E+07	1.16E+07	7.48E+06	1.18E+07	1.02E+07	8.36E+06	8.68E+06	7.82E+06	1.04E+07	9.92E+06	6.88E+06	6.63E+06	9.99E+06	8.39E+06	1.19	0.25	0.124727346
P38117	Electron transfer flavoprotein subunit beta OS=Homo sapiens OX=9606 GN=ETFB PE=1 SV=3	35	40	7	1.80E+07	1.83E+07	1.52E+07	1.77E+07	1.66E+07	1.85E+07	9.78E+06	9.01E+06	1.17E+07	1.12E+07	8.02E+06	6.54E+06	1.74E+07	9.38E+06	1.85	0.89	1.90347E-05
P62081	40S ribosomal protein S7 OS=Homo sapiens OX=9606 GN=RPS7 PE=1 SV=1	46	60	7	2.19E+07	1.64E+07	1.60E+07	1.38E+07	1.41E+07	1.62E+07	1.08E+07	1.12E+07	8.22E+06	8.43E+06	9.74E+06	8.86E+06	1.64E+07	9.55E+06	1.72	0.78	0.00123988
P43034	Platelet-activating factor acetylhydrolase IB subunit alpha OS=Homo sapiens OX=9606 GN=PAFAH1B1 PE=1 SV=2	21	46	7	2.44E+07	2.48E+07	2.75E+07	2.65E+07	3.13E+07	2.50E+07	1.79E+07	1.93E+07	1.91E+07	1.68E+07	1.66E+07	1.69E+07	2.66E+07	1.77E+07	1.50	0.58	0.000117847
P60709	Actin, cytoplasmic 1 OS=Homo sapiens OX=9606 GN=ACTB PE=1 SV=1	83	4529	8	2.68E+09	2.75E+09	2.76E+09	2.73E+09	2.74E+09	2.87E+09	2.36E+09	2.40E+09	2.21E+09	2.45E+09	2.43E+09	2.28E+09	2.75E+09	2.35E+09	1.17	0.23	1.23776E-05
P02533	Keratin, type I cytoskeletal 14 OS=Homo sapiens OX=9606 GN=KRT14 PE=1 SV=4	45	534	8	5.76E+07	4.54E+07	1.69E+07	2.94E+07	2.68E+07	6.84E+07	8.94E+06	6.32E+07	3.50E+07	3.79E+07	3.44E+07	1.94E+07	4.08E+07	3.31E+07	1.23	0.30	0.505745948
P27348	14-3-3 protein theta OS=Homo sapiens OX=9606 GN=YWHAQ PE=1 SV=1	42	159	8	4.20E+07	4.31E+07	5.30E+07	4.45E+07	4.81E+07	4.45E+07	4.50E+07	4.42E+07	4.62E+07	4.66E+07	4.69E+07	3.25E+07	4.59E+07	4.36E+07	1.05	0.07	0.428267914
P01023	Alpha-2-macroglobulin OS=Homo sapiens OX=9606 GN=A2M PE=1 SV=3	9	2864	8	9.99E+09	9.91E+09	1.19E+10	1.07E+10	1.12E+10	1.06E+10	9.80E+09	8.98E+09	1.09E+10	1.00E+10	1.13E+10	9.11E+09	1.07E+10	1.00E+10	1.07	0.10	0.180898824
O94973	AP-2 complex subunit alpha-2 OS=Homo sapiens OX=9606 GN=AP2A2 PE=1 SV=2	25	100	8	2.86E+07	3.25E+07	3.30E+07	3.31E+07	3.49E+07	3.88E+07	1.85E+07	2.06E+07	2.26E+07	1.49E+07	3.09E+07	1.96E+07	3.35E+07	2.12E+07	1.58	0.66	0.001271791
P62937	Peptidyl-prolyl cis-trans isomerase A OS=Homo sapiens OX=9606 GN=PPIA PE=1 SV=2	67	305	8	1.42E+08	1.43E+08	9.47E+07	9.63E+07	8.57E+07	1.16E+08	1.91E+08	1.44E+08	9.83E+07	1.02E+08	1.18E+08	1.20E+08	1.13E+08	1.29E+08	0.88	-0.19	0.377781509
Q15019	Septin-2 OS=Homo sapiens OX=9606 GN=SEPTIN2 PE=1 SV=1	36	190	8	4.72E+07	4.46E+07	4.02E+07	3.99E+07	3.86E+07	3.69E+07	4.29E+07	3.89E+07	2.66E+07	2.86E+07	3.24E+07	3.18E+07	4.12E+07	3.35E+07	1.23	0.30	0.031995356
Q9NQC3	Reticulon-4 OS=Homo sapiens OX=9606 GN=RTN4 PE=1 SV=2	13	131	8	4.46E+07	4.44E+07	4.48E+07	4.97E+07	4.65E+07	4.10E+07	4.26E+07	4.08E+07	4.33E+07	5.24E+07	3.73E+07	3.51E+07	4.52E+07	4.19E+07	1.08	0.11	0.27020323
P60174	Triosephosphate isomerase OS=Homo sapiens OX=9606 GN=TPI1 PE=1 SV=3	40	213	8	6.58E+07	4.64E+07	4.64E+07	4.85E+07	4.24E+07	4.63E+07	6.22E+07	4.86E+07	4.31E+07	4.26E+07	4.30E+07	3.95E+07	4.93E+07	4.65E+07	1.06	0.08	0.567813307
Q16401	26S proteasome non-ATPase regulatory subunit 5 OS=Homo sapiens OX=9606 GN=PSMD5 PE=1 SV=3	21	104	8	1.96E+07	1.58E+07	1.48E+07	1.93E+07	1.32E+07	1.60E+07	1.49E+07	1.26E+07	9.54E+06	7.63E+06	9.71E+06	1.13E+07	1.64E+07	1.09E+07	1.50	0.59	0.00393216
P22626	Heterogeneous nuclear ribonucleoproteins A2/B1 OS=Homo sapiens OX=9606 GN=HNRNPA2B1 PE=1 SV=2	27	122	8	3.88E+07	3.97E+07	3.34E+07	3.71E+07	3.07E+07	3.67E+07	2.36E+07	2.36E+07	2.04E+07	2.11E+07	2.04E+07	1.85E+07	3.61E+07	2.13E+07	1.70	0.76	1.46727E-05
P67775	Serine/threonine-protein phosphatase 2A catalytic subunit alpha isoform OS=Homo sapiens OX=9606 GN=PPP2CA PE=1 SV=1	44	140	8	5.45E+07	5.15E+07	4.01E+07	3.96E+07	4.75E+07	5.46E+07	5.02E+07	4.70E+07	3.50E+07	2.99E+07	3.59E+07	3.58E+07	4.80E+07	3.90E+07	1.23	0.30	0.060717614
Q9NY33	Dipeptidyl peptidase 3 OS=Homo sapiens OX=9606 GN=DPP3 PE=1 SV=2	17	117	8	1.95E+07	1.60E+07	1.80E+07	2.01E+07	1.67E+07	1.71E+07	2.43E+07	2.39E+07	2.49E+07	2.26E+07	2.22E+07	1.96E+07	1.79E+07	2.29E+07	0.78	-0.36	0.000668799
Q93063	Exostosin-2 OS=Homo sapiens OX=9606 GN=EXT2 PE=1 SV=1	17	106	8	1.48E+07	1.64E+07	1.52E+07	1.61E+07	1.31E+07	1.67E+07	2.85E+07	2.63E+07	2.37E+07	2.21E+07	2.86E+07	2.55E+07	1.54E+07	2.58E+07	0.60	-0.74	3.60702E-05
P19823	Inter-alpha-trypsin inhibitor heavy chain H2 OS=Homo sapiens OX=9606 GN=ITIH2 PE=1 SV=2	9	416	8	2.90E+08	3.31E+08	3.31E+08	3.32E+08	3.85E+08	2.87E+08	4.20E+08	4.60E+08	5.65E+08	4.51E+08	5.07E+08	3.40E+08	3.26E+08	4.57E+08	0.71	-0.49	0.006559864
P08582	Melanotransferrin OS=Homo sapiens OX=9606 GN=MELTF PE=1 SV=2	18	115	8	2.63E+07	2.31E+07	2.64E+07	2.30E+07	2.08E+07	2.10E+07	1.47E+07	1.13E+07	1.31E+07	1.27E+07	1.18E+07	8.00E+06	2.34E+07	1.19E+07	1.97	0.98	7.58315E-06
P17980	26S proteasome regulatory subunit 6A OS=Homo sapiens OX=9606 GN=PSMC3 PE=1 SV=3	22	141	8	3.58E+07	3.22E+07	3.17E+07	2.99E+07	2.39E+07	3.26E+07	2.60E+07	2.33E+07	1.82E+07	1.36E+07	1.86E+07	1.85E+07	3.10E+07	1.97E+07	1.57	0.65	0.00088522
P01008	Antithrombin-III OS=Homo sapiens OX=9606 GN=SERPINC1 PE=1 SV=1	21	736	8	5.84E+08	6.21E+08	7.02E+08	6.40E+08	6.61E+08	5.92E+08	7.60E+08	7.41E+08	8.97E+08	7.94E+08	8.18E+08	6.46E+08	6.33E+08	7.76E+08	0.82	-0.29	0.006791831
P01031	Complement C5 OS=Homo sapiens OX=9606 GN=C5 PE=1 SV=4	5	223	8	1.34E+08	1.42E+08	1.28E+08	1.39E+08	1.40E+08	1.25E+08	1.87E+08	1.87E+08	1.88E+08	1.88E+08	1.76E+08	1.64E+08	1.35E+08	1.81E+08	0.74	-0.43	5.50429E-06
P50570	Dynamin-2 OS=Homo sapiens OX=9606 GN=DNM2 PE=1 SV=2	15	110	8	2.89E+07	3.21E+07	2.77E+07	2.75E+07	2.56E+07	3.04E+07	2.34E+07	2.36E+07	2.28E+07	1.57E+07	2.06E+07	1.83E+07	2.87E+07	2.07E+07	1.38	0.47	0.000773146
P08621	U1 small nuclear ribonucleoprotein 70 kDa OS=Homo sapiens OX=9606 GN=SNRNP70 PE=1 SV=2	20	193	8	4.25E+07	4.27E+07	4.38E+07	4.73E+07	4.07E+07	4.33E+07	3.46E+07	3.38E+07	3.31E+07	3.42E+07	3.05E+07	2.70E+07	4.34E+07	3.22E+07	1.35	0.43	3.25549E-05
Q15907	Ras-related protein Rab-11B OS=Homo sapiens OX=9606 GN=RAB11B PE=1 SV=4	42	130	8	6.31E+07	6.65E+07	4.77E+07	5.90E+07	5.91E+07	5.52E+07	5.24E+07	5.69E+07	4.94E+07	5.08E+07	4.93E+07	4.88E+07	5.84E+07	5.12E+07	1.14	0.19	0.044612654
P61353	60S ribosomal protein L27 OS=Homo sapiens OX=9606 GN=RPL27 PE=1 SV=2	49	241	8	1.17E+08	1.19E+08	1.41E+08	1.33E+08	1.26E+08	1.38E+08	8.89E+07	9.29E+07	9.13E+07	9.59E+07	9.12E+07	7.79E+07	1.29E+08	8.97E+07	1.44	0.53	2.64042E-05
P06756	Integrin alpha-V OS=Homo sapiens OX=9606 GN=ITGAV PE=1 SV=2	12	64	8	1.48E+07	1.58E+07	1.41E+07	1.35E+07	1.47E+07	1.48E+07	9.80E+06	6.18E+06	9.05E+06	7.78E+06	8.31E+06	9.03E+06	1.46E+07	8.36E+06	1.75	0.81	5.26082E-06
P11047	Laminin subunit gamma-1 OS=Homo sapiens OX=9606 GN=LAMC1 PE=1 SV=3	7	107	8	1.60E+07	9.65E+06	1.17E+07	1.32E+07	1.12E+07	1.21E+07	1.95E+07	1.80E+07	1.57E+07	1.80E+07	1.56E+07	1.55E+07	1.23E+07	1.71E+07	0.72	-0.47	0.001789035
P38646	Stress-70 protein, mitochondrial OS=Homo sapiens OX=9606 GN=HSPA9 PE=1 SV=2	15	56	8	1.91E+07	1.80E+07	1.11E+07	1.15E+07	1.23E+07	1.80E+07	8.14E+06	1.13E+07	8.76E+06	6.02E+06	7.88E+06	7.42E+06	1.50E+07	8.25E+06	1.82	0.86	0.004856812
Q92900	Regulator of nonsense transcripts 1 OS=Homo sapiens OX=9606 GN=UPF1 PE=1 SV=2	11	49	8	1.85E+07	1.64E+07	2.09E+07	1.73E+07	1.77E+07	1.46E+07	1.21E+07	1.23E+07	1.35E+07	1.00E+07	1.23E+07	1.60E+07	1.75E+07	1.27E+07	1.38	0.47	0.002070798
Q92945	Far upstream element-binding protein 2 OS=Homo sapiens OX=9606 GN=KHSRP PE=1 SV=4	18	102	8	2.83E+07	2.78E+07	2.63E+07	3.12E+07	2.36E+07	2.51E+07	1.56E+07	1.84E+07	1.62E+07	1.42E+07	1.48E+07	1.47E+07	2.71E+07	1.56E+07	1.73	0.79	1.74522E-05
P17301	Integrin alpha-2 OS=Homo sapiens OX=9606 GN=ITGA2 PE=1 SV=1	9	64	8	1.90E+07	1.61E+07	1.58E+07	1.72E+07	1.43E+07	1.47E+07	8.75E+06	1.09E+07	9.42E+06	8.93E+06	1.06E+07	7.97E+06	1.62E+07	9.42E+06	1.72	0.78	2.91955E-05
P11766	Alcohol dehydrogenase class-3 OS=Homo sapiens OX=9606 GN=ADH5 PE=1 SV=4	24	187	8	3.74E+07	3.81E+07	3.78E+07	4.07E+07	3.21E+07	3.87E+07	5.01E+07	5.70E+07	3.78E+07	4.01E+07	4.94E+07	3.49E+07	3.75E+07	4.49E+07	0.83	-0.26	0.089698419
P62917	60S ribosomal protein L8 OS=Homo sapiens OX=9606 GN=RPL8 PE=1 SV=2	23	154	8	4.87E+07	4.96E+07	5.11E+07	5.71E+07	5.16E+07	4.62E+07	3.38E+07	3.29E+07	3.32E+07	3.65E+07	2.81E+07	2.50E+07	5.07E+07	3.16E+07	1.61	0.68	8.62458E-06
Q13263	Transcription intermediary factor 1-beta OS=Homo sapiens OX=9606 GN=TRIM28 PE=1 SV=5	19	43	8	1.53E+07	1.62E+07	1.23E+07	1.24E+07	1.73E+07	1.43E+07	6.57E+06	5.53E+06	5.32E+06	8.10E+06	6.01E+06	4.43E+06	1.46E+07	5.99E+06	2.44	1.29	1.59513E-05
Q13838	Spliceosome RNA helicase DDX39B OS=Homo sapiens OX=9606 GN=DDX39B PE=1 SV=1	21	157	8	1.97E+07	2.60E+07	2.28E+07	2.15E+07	2.24E+07	2.26E+07	1.85E+07	2.16E+07	1.89E+07	1.64E+07	1.54E+07	1.24E+07	2.25E+07	1.72E+07	1.31	0.39	0.007938427
P61221	ATP-binding cassette sub-family E member 1 OS=Homo sapiens OX=9606 GN=ABCE1 PE=1 SV=1	23	84	8	1.78E+07	2.09E+07	2.38E+07	2.07E+07	1.96E+07	1.73E+07	1.47E+07	1.20E+07	1.63E+07	1.51E+07	1.58E+07	1.37E+07	2.00E+07	1.46E+07	1.37	0.45	0.001276361
P21281	V-type proton ATPase subunit B, brain isoform OS=Homo sapiens OX=9606 GN=ATP6V1B2 PE=1 SV=3	21	54	8	1.42E+07	1.28E+07	1.34E+07	1.25E+07	1.28E+07	1.31E+07	1.26E+07	1.37E+07	1.05E+07	9.22E+06	9.38E+06	1.03E+07	1.31E+07	1.09E+07	1.20	0.26	0.029433453
P15144	Aminopeptidase N OS=Homo sapiens OX=9606 GN=ANPEP PE=1 SV=4	12	40	8	8.50E+06	1.12E+07	1.14E+07	8.86E+06	9.72E+06	9.20E+06	3.79E+06	4.35E+06	4.98E+06	3.39E+06	4.84E+06	6.03E+06	9.80E+06	4.56E+06	2.15	1.10	1.11135E-05
Q02809	Procollagen-lysine,2-oxoglutarate 5-dioxygenase 1 OS=Homo sapiens OX=9606 GN=PLOD1 PE=1 SV=2	13	67	8	1.61E+07	1.18E+07	1.21E+07	1.15E+07	1.10E+07	1.09E+07	1.40E+07	1.53E+07	1.80E+07	1.55E+07	1.22E+07	1.23E+07	1.22E+07	1.46E+07	0.84	-0.25	0.08155761
P33992	DNA replication licensing factor MCM5 OS=Homo sapiens OX=9606 GN=MCM5 PE=1 SV=5	14	76	8	2.52E+07	2.60E+07	2.12E+07	2.32E+07	2.15E+07	2.08E+07	1.51E+07	1.57E+07	1.37E+07	1.44E+07	1.21E+07	1.12E+07	2.30E+07	1.37E+07	1.68	0.75	1.3824E-05
Q12860	Contactin-1 OS=Homo sapiens OX=9606 GN=CNTN1 PE=1 SV=1	10	62	8	1.96E+07	1.30E+07	1.38E+07	1.95E+07	1.11E+07	1.44E+07	1.62E+07	1.62E+07	1.40E+07	1.75E+07	1.80E+07	1.19E+07	1.52E+07	1.56E+07	0.97	-0.04	0.813448145
Q01082	Spectrin beta chain, non-erythrocytic 1 OS=Homo sapiens OX=9606 GN=SPTBN1 PE=1 SV=2	5	43	8	1.10E+07	1.06E+07	1.03E+07	1.11E+07	1.14E+07	1.20E+07	7.27E+06	7.06E+06	8.15E+06	6.19E+06	6.53E+06	6.95E+06	1.11E+07	7.02E+06	1.58	0.66	6.96185E-07
Q92973	Transportin-1 OS=Homo sapiens OX=9606 GN=TNPO1 PE=1 SV=2	13	57	8	1.85E+07	1.73E+07	1.80E+07	1.85E+07	1.64E+07	1.74E+07	1.25E+07	1.20E+07	1.13E+07	1.10E+07	1.07E+07	8.06E+06	1.77E+07	1.09E+07	1.62	0.69	1.83323E-05
P54577	Tyrosine--tRNA ligase, cytoplasmic OS=Homo sapiens OX=9606 GN=YARS PE=1 SV=4	18	52	8	1.21E+07	1.23E+07	1.35E+07	1.43E+07	1.49E+07	1.32E+07	8.28E+06	9.78E+06	9.88E+06	7.45E+06	6.91E+06	1.61E+07	1.34E+07	9.73E+06	1.38	0.46	0.042814489
O00203	AP-3 complex subunit beta-1 OS=Homo sapiens OX=9606 GN=AP3B1 PE=1 SV=3	10	47	8	1.11E+08	1.18E+08	5.69E+07	4.55E+07	8.81E+07	5.21E+07	4.64E+07	4.90E+07	2.88E+07	5.36E+07	4.81E+07	4.17E+07	7.85E+07	4.46E+07	1.76	0.81	0.045268859
Q93008	Probable ubiquitin carboxyl-terminal hydrolase FAF-X OS=Homo sapiens OX=9606 GN=USP9X PE=1 SV=3	3	26	8	8.62E+06	9.68E+06	1.05E+07	1.13E+07	9.99E+06	8.78E+06	7.69E+06	8.59E+06	8.72E+06	9.61E+06	7.29E+06	6.43E+06	9.81E+06	8.06E+06	1.22	0.28	0.019114085
P46459	Vesicle-fusing ATPase OS=Homo sapiens OX=9606 GN=NSF PE=1 SV=3	13	55	8	1.56E+07	1.59E+07	1.58E+07	1.50E+07	1.34E+07	1.30E+07	1.23E+07	1.08E+07	1.24E+07	1.17E+07	1.12E+07	1.00E+07	1.48E+07	1.14E+07	1.29	0.37	0.000463665
P00734	Prothrombin OS=Homo sapiens OX=9606 GN=F2 PE=1 SV=2	11	3066	9	8.01E+08	7.08E+08	8.31E+08	8.31E+08	9.22E+08	7.84E+08	1.02E+09	1.03E+09	1.12E+09	1.21E+09	1.16E+09	1.01E+09	8.13E+08	1.09E+09	0.74	-0.43	0.000100157
P50395	Rab GDP dissociation inhibitor beta OS=Homo sapiens OX=9606 GN=GDI2 PE=1 SV=2	40	386	9	1.20E+08	8.70E+07	9.20E+07	9.64E+07	9.06E+07	1.00E+08	1.47E+08	1.20E+08	1.10E+08	8.74E+07	1.29E+08	1.11E+08	9.77E+07	1.17E+08	0.83	-0.26	0.073244611
O00560	Syntenin-1 OS=Homo sapiens OX=9606 GN=SDCBP PE=1 SV=1	59	216	9	7.15E+07	6.29E+07	5.89E+07	5.89E+07	6.12E+07	5.68E+07	7.19E+07	6.80E+07	6.23E+07	5.64E+07	6.03E+07	5.38E+07	6.17E+07	6.21E+07	0.99	-0.01	0.907255649
P13647	Keratin, type II cytoskeletal 5 OS=Homo sapiens OX=9606 GN=KRT5 PE=1 SV=3	28	498	9	6.50E+07	7.00E+07	2.60E+07	4.25E+07	4.26E+07	5.87E+07	1.93E+07	5.90E+07	3.99E+07	3.70E+07	4.14E+07	2.92E+07	5.08E+07	3.76E+07	1.35	0.43	0.162039929
P11940	Polyadenylate-binding protein 1 OS=Homo sapiens OX=9606 GN=PABPC1 PE=1 SV=2	24	228	9	7.64E+07	7.53E+07	6.74E+07	6.82E+07	7.07E+07	7.41E+07	5.39E+07	5.54E+07	4.58E+07	4.45E+07	4.53E+07	4.09E+07	7.20E+07	4.76E+07	1.51	0.60	1.42994E-05
P61160	Actin-related protein 2 OS=Homo sapiens OX=9606 GN=ACTR2 PE=1 SV=1	35	78	9	2.17E+07	2.46E+07	2.43E+07	2.52E+07	2.43E+07	2.45E+07	2.18E+07	2.68E+07	1.81E+07	2.21E+07	2.05E+07	1.91E+07	2.41E+07	2.14E+07	1.13	0.17	0.085349431
P05388	60S acidic ribosomal protein P0 OS=Homo sapiens OX=9606 GN=RPLP0 PE=1 SV=1	42	160	9	4.09E+07	4.27E+07	2.96E+07	3.50E+07	3.67E+07	3.80E+07	4.53E+07	4.47E+07	3.39E+07	3.27E+07	3.67E+07	3.21E+07	3.72E+07	3.76E+07	0.99	-0.02	0.891558759
O00232	26S proteasome non-ATPase regulatory subunit 12 OS=Homo sapiens OX=9606 GN=PSMD12 PE=1 SV=3	22	136	9	3.36E+07	3.44E+07	4.02E+07	4.22E+07	4.25E+07	3.70E+07	2.79E+07	2.53E+07	2.65E+07	2.78E+07	2.91E+07	2.37E+07	3.83E+07	2.67E+07	1.43	0.52	0.00026546
P09211	Glutathione S-transferase P OS=Homo sapiens OX=9606 GN=GSTP1 PE=1 SV=2	57	171	9	5.80E+07	6.38E+07	5.36E+07	5.53E+07	5.47E+07	5.06E+07	6.44E+07	6.33E+07	5.61E+07	6.47E+07	5.87E+07	6.16E+07	5.60E+07	6.14E+07	0.91	-0.13	0.041210727
Q99988	Growth/differentiation factor 15 OS=Homo sapiens OX=9606 GN=GDF15 PE=1 SV=3	40	198	9	5.54E+07	5.52E+07	6.27E+07	5.68E+07	6.13E+07	5.23E+07	6.05E+07	6.11E+07	4.94E+07	5.86E+07	4.89E+07	4.73E+07	5.73E+07	5.43E+07	1.05	0.08	0.360980918
P62191	26S proteasome regulatory subunit 4 OS=Homo sapiens OX=9606 GN=PSMC1 PE=1 SV=1	26	163	9	7.57E+07	7.45E+07	7.12E+07	6.68E+07	7.14E+07	6.96E+07	4.97E+07	5.61E+07	4.22E+07	4.90E+07	4.20E+07	3.87E+07	7.16E+07	4.63E+07	1.55	0.63	4.23889E-05
P10768	S-formylglutathione hydrolase OS=Homo sapiens OX=9606 GN=ESD PE=1 SV=2	50	155	9	5.12E+07	4.97E+07	4.80E+07	4.15E+07	4.51E+07	4.50E+07	4.28E+07	3.80E+07	4.14E+07	4.88E+07	4.04E+07	4.06E+07	4.68E+07	4.20E+07	1.11	0.15	0.046050263
O14818	Proteasome subunit alpha type-7 OS=Homo sapiens OX=9606 GN=PSMA7 PE=1 SV=1	40	174	9	5.27E+07	6.95E+07	1.15E+08	4.62E+07	4.56E+07	5.54E+07	5.14E+07	6.65E+07	4.57E+07	4.49E+07	3.49E+07	2.01E+08	6.41E+07	7.40E+07	0.87	-0.21	0.73465334
P09543	2',3'-cyclic-nucleotide 3'-phosphodiesterase OS=Homo sapiens OX=9606 GN=CNP PE=1 SV=2	29	81	9	1.56E+07	1.50E+07	1.73E+07	1.55E+07	1.57E+07	1.56E+07	8.02E+06	1.11E+07	8.51E+06	1.13E+07	1.04E+07	6.98E+06	1.58E+07	9.38E+06	1.68	0.75	9.51072E-05
P07737	Profilin-1 OS=Homo sapiens OX=9606 GN=PFN1 PE=1 SV=2	71	775	9	1.12E+08	1.04E+08	1.92E+08	1.32E+08	1.78E+08	1.20E+08	9.25E+07	7.79E+07	1.96E+08	1.28E+08	2.05E+08	1.16E+08	1.40E+08	1.36E+08	1.03	0.04	0.891070543
Q13418	Integrin-linked protein kinase OS=Homo sapiens OX=9606 GN=ILK PE=1 SV=2	28	188	9	3.53E+07	3.83E+07	3.05E+07	3.66E+07	3.10E+07	3.63E+07	4.58E+07	4.37E+07	3.67E+07	3.91E+07	3.37E+07	3.54E+07	3.47E+07	3.91E+07	0.89	-0.17	0.094881845
O15067	Phosphoribosylformylglycinamidine synthase OS=Homo sapiens OX=9606 GN=PFAS PE=1 SV=4	9	102	9	6.49E+07	6.28E+07	4.35E+07	5.14E+07	4.44E+07	5.66E+07	6.76E+07	8.71E+07	6.03E+07	5.62E+07	6.16E+07	6.54E+07	5.39E+07	6.64E+07	0.81	-0.30	0.058764056
O76094	Signal recognition particle subunit SRP72 OS=Homo sapiens OX=9606 GN=SRP72 PE=1 SV=3	20	92	9	2.78E+07	2.75E+07	2.25E+07	2.85E+07	2.54E+07	2.73E+07	1.40E+07	1.18E+07	1.23E+07	1.33E+07	1.13E+07	1.18E+07	2.65E+07	1.24E+07	2.14	1.09	2.15844E-06
P17655	Calpain-2 catalytic subunit OS=Homo sapiens OX=9606 GN=CAPN2 PE=1 SV=6	22	56	9	1.37E+07	1.52E+07	1.36E+07	9.32E+06	1.69E+07	1.14E+07	1.65E+07	1.08E+07	6.80E+06	1.30E+07	1.13E+07	7.92E+06	1.34E+07	1.11E+07	1.21	0.27	0.234843222
P02452	Collagen alpha-1(I) chain OS=Homo sapiens OX=9606 GN=COL1A1 PE=1 SV=5	9	26	9	1.15E+06	8.39E+05	6.36E+05	1.57E+06	7.73E+05	1.21E+06	5.38E+06	3.71E+06	2.86E+06	2.34E+06	2.32E+06	3.00E+07	1.03E+06	7.77E+06	0.13	-2.92	0.192156695
Q07020	60S ribosomal protein L18 OS=Homo sapiens OX=9606 GN=RPL18 PE=1 SV=2	39	347	9	1.49E+08	1.57E+08	1.34E+08	1.46E+08	1.44E+08	1.46E+08	1.10E+08	1.13E+08	9.49E+07	1.08E+08	9.83E+07	9.17E+07	1.46E+08	1.03E+08	1.42	0.51	4.32067E-06
P49189	4-trimethylaminobutyraldehyde dehydrogenase OS=Homo sapiens OX=9606 GN=ALDH9A1 PE=1 SV=3	23	337	9	1.34E+08	1.46E+08	1.29E+08	1.70E+08	1.62E+08	1.60E+08	1.80E+08	1.97E+08	2.11E+08	2.14E+08	1.86E+08	1.62E+08	1.50E+08	1.92E+08	0.78	-0.35	0.002838842
P02768	Serum albumin OS=Homo sapiens OX=9606 GN=ALB PE=1 SV=2	13	1326	9	1.76E+09	1.81E+09	1.82E+09	1.90E+09	2.12E+09	2.05E+09	3.15E+09	3.12E+09	3.84E+09	3.51E+09	3.44E+09	2.80E+09	1.91E+09	3.31E+09	0.58	-0.79	7.63559E-05
Q16658	Fascin OS=Homo sapiens OX=9606 GN=FSCN1 PE=1 SV=3	27	96	9	1.95E+07	1.80E+07	1.85E+07	1.63E+07	1.52E+07	1.82E+07	1.70E+07	1.88E+07	1.68E+07	1.71E+07	1.50E+07	1.77E+07	1.76E+07	1.71E+07	1.03	0.04	0.531155237
P51149	Ras-related protein Rab-7a OS=Homo sapiens OX=9606 GN=RAB7A PE=1 SV=1	52	225	9	6.92E+07	6.53E+07	7.49E+07	5.76E+07	6.23E+07	6.36E+07	6.34E+07	6.12E+07	5.95E+07	5.41E+07	5.62E+07	4.97E+07	6.55E+07	5.73E+07	1.14	0.19	0.029785659
O43795	Unconventional myosin-Ib OS=Homo sapiens OX=9606 GN=MYO1B PE=1 SV=3	10	75	9	1.34E+07	1.30E+07	1.55E+07	1.61E+07	1.35E+07	1.31E+07	9.80E+06	8.86E+06	8.47E+06	1.03E+07	6.56E+06	5.13E+06	1.41E+07	8.19E+06	1.72	0.78	0.000209614
Q7KZF4	Staphylococcal nuclease domain-containing protein 1 OS=Homo sapiens OX=9606 GN=SND1 PE=1 SV=1	15	90	9	2.32E+07	2.47E+07	2.12E+07	2.23E+07	2.16E+07	2.33E+07	1.54E+07	1.59E+07	1.62E+07	1.59E+07	1.45E+07	1.13E+07	2.27E+07	1.49E+07	1.53	0.61	1.44295E-05
P07741	Adenine phosphoribosyltransferase OS=Homo sapiens OX=9606 GN=APRT PE=1 SV=2	54	158	9	4.91E+07	4.39E+07	3.86E+07	3.15E+07	3.25E+07	3.61E+07	3.46E+07	3.38E+07	3.16E+07	2.59E+07	2.90E+07	2.81E+07	3.86E+07	3.05E+07	1.27	0.34	0.033032681
P17812	CTP synthase 1 OS=Homo sapiens OX=9606 GN=CTPS1 PE=1 SV=2	19	62	9	1.62E+07	1.66E+07	1.96E+07	1.31E+07	1.98E+07	1.34E+07	1.50E+07	1.19E+07	8.01E+06	9.70E+06	1.13E+07	1.25E+07	1.65E+07	1.14E+07	1.44	0.53	0.008538244
P62277	40S ribosomal protein S13 OS=Homo sapiens OX=9606 GN=RPS13 PE=1 SV=2	44	178	9	6.04E+07	5.87E+07	5.66E+07	5.97E+07	5.51E+07	5.56E+07	3.66E+07	4.07E+07	3.28E+07	3.30E+07	3.67E+07	4.37E+07	5.77E+07	3.73E+07	1.55	0.63	1.07631E-05
O60832	H/ACA ribonucleoprotein complex subunit DKC1 OS=Homo sapiens OX=9606 GN=DKC1 PE=1 SV=3	29	68	9	3.95E+07	3.06E+07	2.10E+07	1.87E+07	2.39E+07	2.60E+07	1.74E+07	2.41E+07	1.07E+07	1.10E+07	9.26E+06	1.50E+07	2.66E+07	1.46E+07	1.82	0.87	0.011629865
P08648	Integrin alpha-5 OS=Homo sapiens OX=9606 GN=ITGA5 PE=1 SV=2	12	79	9	3.38E+07	3.04E+07	2.66E+07	2.68E+07	3.43E+07	2.31E+07	1.25E+07	9.50E+06	1.13E+07	1.26E+07	1.10E+07	1.04E+07	2.92E+07	1.12E+07	2.60	1.38	9.5166E-05

Supplementary Table 4. List of all proteins identified by label free proteomics analysis.

Accession	Description	Coverage [%]	# PSMs	# Unique Peptides	Amitriptyline 1	Amitriptyline 2	Amitriptyline 3	Amitriptyline 4	Amitriptyline 5	Amitriptyline 6	Vehicle 1	Vehicle 2	Vehicle 3	Vehicle 4	Vehicle 5	Vehicle 6	Average amitriptyline	Average vehicle	Ratio amitriptyline/ vehicle	Log 2 fold change (amitriptyline /vehicle)	P-value
Q9NSD9	Phenylalanine--tRNA ligase beta subunit OS=Homo sapiens OX=9606 GN=FARSB PE=1 SV=3	18	72	9	1.66E+07	1.58E+07	1.80E+07	2.25E+07	1.52E+07	2.04E+07	1.42E+07	1.35E+07	1.15E+07	1.41E+07	1.32E+07	1.37E+07	1.81E+07	1.34E+07	1.35	0.44	0.008214467
Q04637	Eukaryotic translation initiation factor 4 gamma 1 OS=Homo sapiens OX=9606 GN=EIF4G1 PE=1 SV=4	8	76	9	2.43E+07	2.43E+07	2.31E+07	2.50E+07	2.23E+07	2.11E+07	1.08E+07	1.20E+07	1.09E+07	1.32E+07	1.07E+07	9.81E+06	2.33E+07	1.13E+07	2.07	1.05	4.12192E-08
Q07955	Serine/arginine-rich splicing factor 1 OS=Homo sapiens OX=9606 GN=SRSF1 PE=1 SV=2	35	68	9	1.62E+07	1.55E+07	1.62E+07	1.71E+07	1.48E+07	1.57E+07	1.52E+07	1.58E+07	1.45E+07	1.40E+07	1.29E+07	1.32E+07	1.59E+07	1.43E+07	1.12	0.16	0.016194833
Q9Y6G9	Cytoplasmic dynein 1 light intermediate chain 1 OS=Homo sapiens OX=9606 GN=DYNC1LI1 PE=1 SV=3	22	47	9	8.72E+07	8.26E+07	8.82E+07	8.81E+07	9.80E+07	8.93E+07	9.47E+07	1.11E+08	9.29E+07	1.12E+08	9.19E+07	7.83E+07	8.89E+07	9.68E+07	0.92	-0.12	0.205676263
P04439	HLA class I histocompatibility antigen, A alpha chain OS=Homo sapiens OX=9606 GN=HLA-A PE=1 SV=2	52	729	10	3.40E+08	3.67E+08	3.35E+08	3.67E+08	3.49E+08	3.52E+08	2.03E+08	2.21E+08	1.95E+08	2.14E+08	1.88E+08	1.74E+08	3.52E+08	1.99E+08	1.77	0.82	2.07833E-08
P63104	14-3-3 protein zeta/delta OS=Homo sapiens OX=9606 GN=YWHAZ PE=1 SV=1	53	210	10	4.59E+07	3.89E+07	4.21E+07	5.23E+07	4.67E+07	4.03E+07	4.65E+07	4.39E+07	4.42E+07	4.25E+07	3.57E+07	3.66E+07	4.43E+07	4.16E+07	1.07	0.09	0.326383081
O43707	Alpha-actinin-4 OS=Homo sapiens OX=9606 GN=ACTN4 PE=1 SV=2	23	198	10	2.65E+07	2.46E+07	1.98E+07	2.32E+07	2.10E+07	2.21E+07	2.46E+07	2.07E+07	1.94E+07	1.82E+07	1.75E+07	1.69E+07	2.29E+07	1.95E+07	1.17	0.23	0.053517293
Q16181	Septin-7 OS=Homo sapiens OX=9606 GN=SEPTIN7 PE=1 SV=2	29	139	10	3.77E+07	2.80E+07	3.42E+07	3.22E+07	3.11E+07	3.62E+07	3.98E+07	3.01E+07	3.23E+07	2.72E+07	2.72E+07	2.99E+07	3.32E+07	3.11E+07	1.07	0.10	0.398713546
O75533	Splicing factor 3B subunit 1 OS=Homo sapiens OX=9606 GN=SF3B1 PE=1 SV=3	14	121	10	2.24E+07	2.11E+07	2.33E+07	2.28E+07	2.54E+07	2.29E+07	1.99E+07	1.88E+07	1.24E+07	1.35E+07	1.72E+07	1.36E+07	2.30E+07	1.59E+07	1.44	0.53	0.001566099
O15371	Eukaryotic translation initiation factor 3 subunit D OS=Homo sapiens OX=9606 GN=EIF3D PE=1 SV=1	28	105	10	2.09E+07	1.66E+07	2.64E+07	1.69E+07	2.56E+07	2.85E+07	9.59E+06	9.56E+06	1.41E+07	7.46E+06	1.49E+07	1.42E+07	2.25E+07	1.16E+07	1.93	0.95	0.001958966
P41091	Eukaryotic translation initiation factor 2 subunit 3 OS=Homo sapiens OX=9606 GN=EIF2S3 PE=1 SV=3	37	128	10	3.39E+07	3.87E+07	3.39E+07	3.80E+07	3.54E+07	3.72E+07	2.79E+07	2.86E+07	2.38E+07	2.64E+07	2.73E+07	2.59E+07	3.62E+07	2.66E+07	1.36	0.44	7.93979E-06
P43121	Cell surface glycoprotein MUC18 OS=Homo sapiens OX=9606 GN=MCAM PE=1 SV=2	24	106	10	3.71E+07	3.03E+07	3.18E+07	2.89E+07	2.73E+07	3.22E+07	1.54E+07	1.51E+07	1.24E+07	1.19E+07	1.26E+07	1.40E+07	3.13E+07	1.36E+07	2.30	1.20	9.26782E-06
Q9BUJ2	Heterogeneous nuclear ribonucleoprotein U-like protein 1 OS=Homo sapiens OX=9606 GN=HNRNPUL1 PE=1 SV=2	22	73	10	2.09E+07	1.55E+07	1.95E+07	2.32E+07	2.42E+07	2.04E+07	1.75E+07	1.09E+07	1.04E+07	1.24E+07	9.57E+06	8.70E+06	2.06E+07	1.16E+07	1.78	0.83	0.000525162
Q02878	60S ribosomal protein L6 OS=Homo sapiens OX=9606 GN=RPL6 PE=1 SV=3	42	185	10	9.32E+07	9.06E+07	7.39E+07	6.28E+07	7.31E+07	6.57E+07	5.74E+07	5.36E+07	4.93E+07	4.22E+07	4.92E+07	3.67E+07	7.66E+07	4.81E+07	1.59	0.67	0.001378615
P26599	Polypyrimidine tract-binding protein 1 OS=Homo sapiens OX=9606 GN=PTBP1 PE=1 SV=1	28	126	10	3.28E+07	3.04E+07	2.67E+07	3.01E+07	2.80E+07	3.16E+07	2.33E+07	2.15E+07	1.77E+07	1.83E+07	1.80E+07	1.38E+07	2.99E+07	1.88E+07	1.60	0.67	8.70672E-05
P61978	Heterogeneous nuclear ribonucleoprotein K OS=Homo sapiens OX=9606 GN=HNRNPK PE=1 SV=1	28	161	10	7.59E+07	8.44E+07	7.77E+07	7.31E+07	7.04E+07	7.33E+07	4.64E+07	5.21E+07	4.07E+07	4.36E+07	4.11E+07	3.97E+07	7.58E+07	4.40E+07	1.72	0.79	4.35594E-07
Q16531	DNA damage-binding protein 1 OS=Homo sapiens OX=9606 GN=DDB1 PE=1 SV=1	14	105	10	3.41E+07	3.71E+07	2.40E+07	2.48E+07	2.52E+07	2.33E+07	2.37E+07	2.19E+07	1.60E+07	1.73E+07	1.78E+07	1.74E+07	2.81E+07	1.90E+07	1.48	0.56	0.0113477
P14866	Heterogeneous nuclear ribonucleoprotein L OS=Homo sapiens OX=9606 GN=HNRNPL PE=1 SV=2	26	78	10	2.48E+07	2.29E+07	2.19E+07	1.98E+07	1.99E+07	2.39E+07	1.19E+07	1.23E+07	1.21E+07	1.21E+07	1.14E+07	6.79E+06	2.22E+07	1.11E+07	2.00	1.00	3.65452E-06
O00571	ATP-dependent RNA helicase DDX3X OS=Homo sapiens OX=9606 GN=DDX3X PE=1 SV=3	20	96	10	2.64E+07	2.56E+07	2.61E+07	2.49E+07	1.99E+07	2.56E+07	1.81E+07	1.92E+07	1.55E+07	1.26E+07	1.63E+07	1.14E+07	2.48E+07	1.55E+07	1.59	0.67	0.000212078
P22087	rRNA 2'-O-methyltransferase fibrillarin OS=Homo sapiens OX=9606 GN=FBL PE=1 SV=2	41	127	10	3.48E+07	3.73E+07	3.76E+07	3.30E+07	3.77E+07	3.88E+07	2.55E+07	2.71E+07	2.09E+07	2.20E+07	1.90E+07	2.09E+07	3.65E+07	2.26E+07	1.62	0.69	8.52681E-06
P53618	Coatomer subunit beta OS=Homo sapiens OX=9606 GN=COPB1 PE=1 SV=3	17	90	10	2.82E+07	3.15E+07	3.62E+07	2.66E+07	4.54E+07	3.74E+07	1.96E+07	2.07E+07	2.41E+07	1.85E+07	1.99E+07	1.61E+07	3.42E+07	1.98E+07	1.73	0.79	0.002640345
Q15582	Transforming growth factor-beta-induced protein ig-h3 OS=Homo sapiens OX=9606 GN=TGFBI PE=1 SV=1	19	94	10	1.63E+07	1.66E+07	2.40E+07	2.19E+07	2.22E+07	2.39E+07	3.79E+07	2.42E+07	3.34E+07	3.04E+07	3.88E+07	3.80E+07	2.08E+07	3.38E+07	0.62	-0.70	0.0013146
Q9NTK5	Obg-like ATPase 1 OS=Homo sapiens OX=9606 GN=OLA1 PE=1 SV=2	28	148	10	3.21E+07	3.01E+07	2.74E+07	2.57E+07	2.97E+07	2.58E+07	3.69E+07	3.37E+07	3.18E+07	2.56E+07	3.38E+07	1.96E+07	2.85E+07	3.02E+07	0.94	-0.09	0.548411122
Q08945	FACT complex subunit SSRP1 OS=Homo sapiens OX=9606 GN=SSRP1 PE=1 SV=1	19	93	10	3.07E+07	2.70E+07	3.08E+07	3.08E+07	2.70E+07	2.86E+07	2.07E+07	1.59E+07	1.74E+07	1.56E+07	1.28E+07	1.03E+07	2.92E+07	1.55E+07	1.89	0.92	5.03591E-05
Q92616	eIF-2-alpha kinase activator GCN1 OS=Homo sapiens OX=9606 GN=GCN1 PE=1 SV=6	6	45	10	3.90E+07	3.54E+07	3.28E+07	2.19E+07	3.39E+07	3.02E+07	3.42E+07	3.87E+07	3.93E+07	2.11E+07	3.84E+07	1.86E+07	3.22E+07	3.17E+07	1.01	0.02	0.919279436
Q16610	Extracellular matrix protein 1 OS=Homo sapiens OX=9606 GN=ECM1 PE=1 SV=2	28	28	10	4.92E+06	4.40E+06	1.29E+06	3.02E+06	1.36E+06	3.21E+06	1.45E+07	4.69E+06	6.20E+06	1.45E+07	5.83E+06	5.18E+07	3.03E+06	1.62E+07	0.19	-2.42	0.131775697
P27694	Replication protein A 70 kDa DNA-binding subunit OS=Homo sapiens OX=9606 GN=RPA1 PE=1 SV=2	24	94	10	3.02E+07	3.32E+07	2.30E+07	2.59E+07	2.36E+07	3.01E+07	2.36E+07	2.38E+07	1.84E+07	1.73E+07	1.66E+07	1.52E+07	2.77E+07	1.91E+07	1.44	0.53	0.003686854
P62249	40S ribosomal protein S16 OS=Homo sapiens OX=9606 GN=RPS16 PE=1 SV=2	49	420	10	1.68E+08	1.76E+08	1.57E+08	1.58E+08	1.52E+08	1.47E+08	1.22E+08	1.27E+08	1.02E+08	1.06E+08	1.14E+08	1.03E+08	1.60E+08	1.12E+08	1.42	0.50	1.45355E-05
P43490	Nicotinamide phosphoribosyltransferase OS=Homo sapiens OX=9606 GN=NAMPT PE=1 SV=1	30	77	10	2.78E+07	2.95E+07	2.70E+07	2.76E+07	2.73E+07	2.24E+07	2.44E+07	2.02E+07	1.80E+07	2.02E+07	2.24E+07	1.59E+07	2.69E+07	2.02E+07	1.33	0.42	0.001806899
P35573	Glycogen debranching enzyme OS=Homo sapiens OX=9606 GN=AGL PE=1 SV=3	9	130	10	2.65E+07	2.66E+07	2.20E+07	1.90E+07	1.97E+07	2.48E+07	3.69E+07	3.59E+07	2.96E+07	2.56E+07	3.36E+07	2.33E+07	2.31E+07	3.08E+07	0.75	-0.42	0.01975363
O60763	General vesicular transport factor p115 OS=Homo sapiens OX=9606 GN=USO1 PE=1 SV=2	11	94	10	8.87E+07	9.47E+07	1.34E+08	1.54E+08	1.55E+08	1.21E+08	1.33E+08	1.22E+08	1.88E+08	1.68E+08	1.38E+08	1.19E+08	1.25E+08	1.45E+08	0.86	-0.21	0.244413418
P22102	Trifunctional purine biosynthetic protein adenosine-3 OS=Homo sapiens OX=9606 GN=GART PE=1 SV=1	16	60	10	2.28E+07	2.67E+07	2.14E+07	2.88E+07	2.37E+07	2.06E+07	1.44E+07	1.62E+07	1.36E+07	1.55E+07	1.19E+07	1.07E+07	2.40E+07	1.37E+07	1.75	0.80	0.000120978
O00429	Dynamin-1-like protein OS=Homo sapiens OX=9606 GN=DNM1L PE=1 SV=2	19	81	10	1.56E+07	2.01E+07	1.72E+07	1.49E+07	1.72E+07	1.56E+07	1.48E+07	1.45E+07	1.78E+07	1.41E+07	1.88E+07	1.30E+07	1.68E+07	1.55E+07	1.08	0.11	0.316199479
P20774	Mimecan OS=Homo sapiens OX=9606 GN=OGN PE=1 SV=1	27	295	10	1.03E+08	9.22E+07	9.30E+07	1.25E+08	1.11E+08	9.77E+07	1.46E+08	1.28E+08	1.27E+08	1.42E+08	1.29E+08	1.17E+08	1.04E+08	1.32E+08	0.79	-0.35	0.001925452
P18206	Vinculin OS=Homo sapiens OX=9606 GN=VCL PE=1 SV=4	10	94	10	2.48E+07	2.66E+07	3.00E+07	3.05E+07	2.24E+07	2.70E+07	3.10E+07	2.94E+07	3.32E+07	3.17E+07	3.14E+07	3.15E+07	2.69E+07	3.14E+07	0.86	-0.22	0.013918794
Q9UNH7	Sorting nexin-6 OS=Homo sapiens OX=9606 GN=SNX6 PE=1 SV=1	22	64	10	1.76E+07	2.24E+07	2.11E+07	2.02E+07	1.99E+07	1.82E+07	1.57E+07	1.89E+07	1.78E+07	1.45E+07	1.78E+07	1.78E+07	1.99E+07	1.71E+07	1.16	0.22	0.018710378
P08779	Keratin, type I cytoskeletal 16 OS=Homo sapiens OX=9606 GN=KRT16 PE=1 SV=4	53	480	11	1.66E+08	7.54E+07	4.25E+07	5.44E+07	4.59E+07	1.39E+08	2.00E+07	1.66E+08	8.20E+07	9.51E+07	5.68E+07	3.56E+07	8.72E+07	7.59E+07	1.15	0.20	0.715868387
P06748	Nucleophosmin OS=Homo sapiens OX=9606 GN=NPM1 PE=1 SV=2	38	225	11	1.21E+08	1.30E+08	1.53E+08	1.42E+08	1.36E+08	1.55E+08	9.02E+07	8.84E+07	9.02E+07	6.97E+07	7.51E+07	7.11E+07	1.39E+08	8.08E+07	1.73	0.79	8.36565E-06
P16401	Histone H1.5 OS=Homo sapiens OX=9606 GN=H1-5 PE=1 SV=3	36	739	11	8.66E+08	8.83E+08	8.33E+08	8.93E+08	8.87E+08	8.12E+08	7.64E+08	7.75E+08	8.18E+08	8.20E+08	7.09E+08	6.48E+08	8.62E+08	7.56E+08	1.14	0.19	0.009254022
P12814	Alpha-actinin-1 OS=Homo sapiens OX=9606 GN=ACTN1 PE=1 SV=2	25	185	11	4.80E+07	4.40E+07	4.02E+07	3.82E+07	3.56E+07	4.15E+07	3.72E+07	3.15E+07	2.38E+07	2.43E+07	2.76E+07	2.64E+07	4.13E+07	2.85E+07	1.45	0.54	0.000939916
P00338	L-lactate dehydrogenase A chain OS=Homo sapiens OX=9606 GN=LDHA PE=1 SV=2	51	387	11	1.40E+08	1.53E+08	1.33E+08	1.43E+08	1.43E+08	1.29E+08	9.49E+07	9.04E+07	9.97E+07	1.01E+08	8.53E+07	8.32E+07	1.40E+08	9.25E+07	1.52	0.60	1.18618E-06
P23142	Fibulin-1 OS=Homo sapiens OX=9606 GN=FBLN1 PE=1 SV=4	16	591	11	2.25E+08	2.13E+08	1.96E+08	2.24E+08	1.89E+08	1.91E+08	3.24E+08	3.25E+08	2.76E+08	3.48E+08	2.69E+08	2.54E+08	2.06E+08	2.99E+08	0.69	-0.54	0.000983748
P07195	L-lactate dehydrogenase B chain OS=Homo sapiens OX=9606 GN=LDHB PE=1 SV=2	50	544	11	2.07E+08	1.91E+08	1.82E+08	1.88E+08	1.86E+08	1.63E+08	1.68E+08	1.60E+08	1.68E+08	1.77E+08	1.70E+08	1.56E+08	1.86E+08	1.67E+08	1.12	0.16	0.018377262
P00966	Argininosuccinate synthase OS=Homo sapiens OX=9606 GN=ASS1 PE=1 SV=2	41	372	11	1.21E+08	1.16E+08	1.30E+08	1.25E+08	1.27E+08	1.35E+08	1.98E+08	1.81E+08	1.68E+08	1.67E+08	1.75E+08	1.47E+08	1.26E+08	1.73E+08	0.73	-0.46	0.00050468
Q9ULV4	Coronin-1C OS=Homo sapiens OX=9606 GN=CORO1C PE=1 SV=1	30	244	11	6.23E+07	6.02E+07	5.89E+07	5.74E+07	5.43E+07	4.94E+07	6.24E+07	5.12E+07	5.33E+07	4.33E+07	4.56E+07	4.28E+07	5.71E+07	4.98E+07	1.15	0.20	0.074563415
Q9Y265	RuvB-like 1 OS=Homo sapiens OX=9606 GN=RUVBL1 PE=1 SV=1	32	216	11	5.63E+07	5.52E+07	4.22E+07	4.58E+07	4.16E+07	4.78E+07	3.88E+07	4.20E+07	2.87E+07	3.48E+07	3.03E+07	2.88E+07	4.81E+07	3.39E+07	1.42	0.50	0.002162321
Q92841	Probable ATP-dependent RNA helicase DDX17 OS=Homo sapiens OX=9606 GN=DDX17 PE=1 SV=2	26	154	11	6.10E+07	6.65E+07	5.71E+07	6.29E+07	6.32E+07	5.89E+07	4.85E+07	4.42E+07	3.78E+07	4.24E+07	3.36E+07	3.26E+07	6.16E+07	3.99E+07	1.55	0.63	8.79285E-05
P46821	Microtubule-associated protein 1B OS=Homo sapiens OX=9606 GN=MAP1B PE=1 SV=2	8	69	11	2.06E+07	2.04E+07	1.70E+07	1.81E+07	1.61E+07	1.90E+07	1.21E+07	1.36E+07	9.90E+06	1.02E+07	9.57E+06	1.07E+07	1.86E+07	1.10E+07	1.68	0.75	1.75459E-05
P55263	Adenosine kinase OS=Homo sapiens OX=9606 GN=ADK PE=1 SV=2	35	173	11	5.46E+07	4.62E+07	4.31E+07	6.32E+07	5.59E+07	5.94E+07	7.04E+07	7.28E+07	6.43E+07	6.00E+07	6.26E+07	5.23E+07	5.37E+07	6.37E+07	0.84	-0.25	0.045136293
P01871	Immunoglobulin heavy constant mu OS=Homo sapiens OX=9606 GN=IGHM PE=1 SV=4	31	44	11	9.35E+05	8.21E+05		4.69E+05		3.15E+05	8.75E+06	1.57E+06	4.66E+05			7.40E+07	6.35E+05	2.12E+07	0.03	-5.06	0.329295506
P62333	26S proteasome regulatory subunit 10B OS=Homo sapiens OX=9606 GN=PSMC6 PE=1 SV=1	34	140	11	5.19E+07	4.97E+07	4.26E+07	4.42E+07	4.56E+07	4.76E+07	3.50E+07	3.60E+07	3.47E+07	3.29E+07	3.12E+07	2.81E+07	4.69E+07	3.30E+07	1.42	0.51	2.3661E-05
P33176	Kinesin-1 heavy chain OS=Homo sapiens OX=9606 GN=KIF5B PE=1 SV=1	18	94	11	2.13E+07	1.83E+07	2.05E+07	2.04E+07	2.01E+07	2.24E+07	1.08E+07	8.95E+06	8.30E+06	8.43E+06	8.53E+06	8.81E+06	2.05E+07	8.96E+06	2.29	1.19	5.34554E-08
P33993	DNA replication licensing factor MCM7 OS=Homo sapiens OX=9606 GN=MCM7 PE=1 SV=4	22	108	11	2.79E+07	2.39E+07	2.49E+07	2.60E+07	2.32E+07	2.40E+07	1.27E+07	1.43E+07	1.29E+07	1.34E+07	1.25E+07	1.11E+07	2.50E+07	1.28E+07	1.95	0.96	3.55614E-07
Q99873	Protein arginine N-methyltransferase 1 OS=Homo sapiens OX=9606 GN=PRMT1 PE=1 SV=3	30	165	11	5.12E+07	5.65E+07	5.33E+07	5.57E+07	5.20E+07	4.87E+07	4.43E+07	4.30E+07	3.91E+07	4.16E+07	3.71E+07	3.65E+07	5.29E+07	4.03E+07	1.31	0.39	3.3042E-05
Q14203	Dynactin subunit 1 OS=Homo sapiens OX=9606 GN=DCTN1 PE=1 SV=3	12	91	11	2.45E+07	2.60E+07	2.64E+07	2.68E+07	2.70E+07	2.61E+07	1.31E+07	1.44E+07	1.63E+07	1.48E+07	1.42E+07	1.32E+07	2.61E+07	1.43E+07	1.83	0.87	7.37935E-09
Q7L576	Cytoplasmic FMR1-interacting protein 1 OS=Homo sapiens OX=9606 GN=CYFIP1 PE=1 SV=1	10	141	11	3.54E+07	3.15E+07	2.30E+07	2.78E+07	2.78E+07	2.41E+07	2.41E+07	2.09E+07	2.03E+07	2.21E+07	1.94E+07	1.81E+07	2.83E+07	2.08E+07	1.36	0.44	0.00889316
O94979	Protein transport protein Sec31A OS=Homo sapiens OX=9606 GN=SEC31A PE=1 SV=3	11	122	11	3.68E+07	3.97E+07	3.11E+07	3.55E+07	3.64E+07	3.76E+07	3.10E+07	2.71E+07	2.65E+07	2.48E+07	2.48E+07	2.38E+07	3.62E+07	2.63E+07	1.37	0.46	9.75311E-05
Q02543	60S ribosomal protein L18a OS=Homo sapiens OX=9606 GN=RPL18A PE=1 SV=2	44	214	11	9.26E+07	8.26E+07	7.36E+07	8.74E+07	8.43E+07	8.05E+07	5.81E+07	5.09E+07	4.45E+07	5.21E+07	4.48E+07	4.56E+07	8.35E+07	4.93E+07	1.69	0.76	2.02155E-06
Q9P2R3	Rabankyrin-5 OS=Homo sapiens OX=9606 GN=ANKFY1 PE=1 SV=2	13	62	11	1.95E+07	1.84E+07	1.68E+07	1.54E+07	1.49E+07	1.75E+07	1.71E+07	1.45E+07	1.70E+07	1.29E+07	1.47E+07	1.47E+07	1.71E+07	1.52E+07	1.13	0.17	0.078339289
P49591	Serine--tRNA ligase, cytoplasmic OS=Homo sapiens OX=9606 GN=SARS PE=1 SV=3	31	147	11	2.75E+07	2.76E+07	2.58E+07	2.40E+07	2.64E+07	3.08E+07	1.85E+07	1.74E+07	1.30E+07	1.25E+07	1.50E+07	1.34E+07	2.70E+07	1.50E+07	1.81	0.85	5.81886E-06
P47897	Glutamine--tRNA ligase OS=Homo sapiens OX=9606 GN=QARS PE=1 SV=1	18	115	11	2.85E+07	2.53E+07	2.30E+07	3.19E+07	2.51E+07	2.34E+07	1.47E+07	1.33E+07	1.98E+07	1.72E+07	1.52E+07	1.38E+07	2.62E+07	1.57E+07	1.67	0.74	0.000166012
P46781	40S ribosomal protein S9 OS=Homo sapiens OX=9606 GN=RPS9 PE=1 SV=3	38	376	11	1.46E+08	1.61E+08	1.80E+08	1.68E+08	1.80E+08	1.43E+08	1.18E+08	1.23E+08	1.16E+08	1.20E+08	1.01E+08	8.01E+07	1.63E+08	1.10E+08	1.49	0.57	0.000204504
Q9BXJ9	N-alpha-acetyltransferase 15, NatA auxiliary subunit OS=Homo sapiens OX=9606 GN=NAA15 PE=1 SV=1	13	76	11	2.13E+07	1.90E+07	1.90E+07	1.97E+07	1.83E+07	2.14E+07	1.44E+07	1.39E+07	1.05E+07	1.22E+07	1.27E+07	1.11E+07	1.98E+07	1.25E+07	1.59	0.66	5.6599E-06
P68366	Tubulin alpha-4A chain OS=Homo sapiens OX=9606 GN=TUBA4A PE=1 SV=1	75	3579	12	3.82E+08	3.64E+08	2.90E+08	2.84E+08	2.80E+08	3.51E+08	3.82E+08	4.03E+08	3.42E+08	2.93E+08	3.91E+08	3.20E+08	3.25E+08	3.55E+08	0.92	-0.13	0.271398581
P68104	Elongation factor 1-alpha 1 OS=Homo sapiens OX=9606 GN=EEF1A1 PE=1 SV=1	53	1737	12	1.45E+09	1.56E+09	1.49E+09	1.58E+09	1.60E+09	1.62E+09	1.23E+09	1.33E+09	1.43E+09	1.36E+09	1.43E+09	1.11E+09	1.55E+09	1.32E+09	1.18	0.23	0.004161394
P06576	ATP synthase subunit beta, mitochondrial OS=Homo sapiens OX=9606 GN=ATP5F1B PE=1 SV=3	43	227	12	4.97E+07	5.47E+07	5.59E+07	4.16E+07	5.42E+07	4.97E+07	5.14E+07	5.91E+07	4.67E+07	4.52E+07	5.16E+07	4.65E+07	5.10E+07	5.01E+07	1.02	0.03	0.769530694
P08865	40S ribosomal protein SA OS=Homo sapiens OX=9606 GN=RPSA PE=1 SV=4	44	427	12	2.68E+08	2.89E+08	2.13E+08	2.48E+08	2.18E+08	2.37E+08	2.00E+08	2.27E+08	1.84E+08	2.00E+08	1.87E+08	1.82E+08	2.45E+08	1.97E+08	1.25	0.32	0.007669479
P09525	Annexin A4 OS=Homo sapiens OX=9606 GN=ANXA4 PE=1 SV=4	50	226	12	5.51E+07	4.82E+07	4.95E+07	5.84E+07	4.55E+07	5.70E+07	5.13E+07	4.61E+07	4.60E+07	3.68E+07	3.96E+07	3.66E+07	5.23E+07	4.27E+07	1.22	0.29	0.015035297
P61224	Ras-related protein Rap-1b OS=Homo sapiens OX=9606 GN=RAP1B PE=1 SV=1	73	360	12	1.86E+08	1.83E+08	1.73E+08	1.91E+08	1.71E+08	1.83E+08	2.09E+08	1.98E+08	1.96E+08	1.91E+08	2.07E+08	1.88E+08	1.81E+08	1.98E+08	0.91	-0.13	0.004417743
O75390	Citrate synthase, mitochondrial OS=Homo sapiens OX=9606 GN=CS PE=1 SV=2	34	274	12	8.63E+07	9.06E+07	7.76E+07	9.45E+07	9.24E+07	8.71E+07	7.71E+07	7.70E+07	6.26E+07	6.73E+07	6.63E+07	6.43E+07	8.81E+07	6.91E+07	1.27	0.35	0.000347916
P06396	Gelsolin OS=Homo sapiens OX=9606 GN=GSN PE=1 SV=1	20	523	12	1.95E+08	1.96E+08	1.56E+08	1.86E+08	1.62E+08	1.69E+08	2.54E+08	2.36E+08	2.25E+08	2.57E+08	1.93E+08	1.83E+08	1.77E+08	2.25E+08	0.79	-0.34	0.011782566
P04075	Fructose-bisphosphate aldolase A OS=Homo sapiens OX=9606 GN=ALDOA PE=1 SV=2	55	201	12	6.24E+07	5.46E+07	5.45E+07	6.10E+07	5.42E+07	5.59E+07	4.61E+07	4.94E+07	4.87E+07	5.05E+07	4.49E+07	4.03E+07	5.71E+07	4.67E+07	1.22	0.29	0.000613766
P68400	Casein kinase II subunit alpha OS=Homo sapiens OX=9606 GN=CSNK2A1 PE=1 SV=1	44	213	12	1.01E+08	9.81E+07	7.92E+07	8.27E+07	7.58E+07	8.39E+07	8.75E+07	7.54E+07	6.92E+07	6.40E+07	7.07E+07	6.99E+07	8.68E+07	7.28E+07	1.19	0.25	0.02688942
P15880	40S ribosomal protein S2 OS=Homo sapiens OX=9606 GN=RPS2 PE=1 SV=2	41	382	12	2.99E+08	2.89E+08	2.95E+08	3.31E+08	2.88E+08	2.82E+08	1.98E+08	2.00E+08	1.94E+08	2.01E+08	1.84E+08	1.71E+08	2.97E+08	1.91E+08	1.55	0.64	8.86675E-07
P00488	Coagulation factor XIII A chain OS=Homo sapiens OX=9606 GN=F13A1 PE=1 SV=4	22	225	12	7.38E+07	5.69E+07	6.09E+07	6.05E+07	6.36E+07	6.21E+07	9.12E+07	7.53E+07	5.66E+07	6.21E+07	6.20E+07	1.41E+08	6.30E+07	8.13E+07	0.77	-0.37	0.218186447
Q08431	Lactadherin OS=Homo sapiens OX=9606 GN=MFGE8 PE=1 SV=3	41	289	12	5.92E+07	5.27E+07	4.39E+07	4.99E+07	4.55E+07	4.56E+07	9.13E+07	1.01E+08	7.57E+07	7.90E+07	7.34E+07	6.73E+07	4.95E+07	8.13E+07	0.61	-0.72	0.000741471
Q9BQG0	Myb-binding protein 1A OS=Homo sapiens OX=9606 GN=MYBBP1A PE=1 SV=2	13	132	12	2.29E+07	3.08E+07	2.44E+07	2.72E+07	2.82E+07	2.62E+07	1.61E+07	1.65E+07	1.19E+07	1.50E+07	1.37E+07	1.36E+07	2.66E+07	1.45E+07	1.84	0.88	1.30784E-05
Q96CW1	AP-2 complex subunit mu OS=Homo sapiens OX=9606 GN=AP2M1 PE=1 SV=2	37	237	12	8.04E+07	8.16E+07	6.98E+07	7.69E+07	7.36E+07	7.14E+07	5.47E+07	5.10E+07	4.86E+07	5.38E+07	4.74E+07	4.28E+07	7.56E+07	4.97E+07	1.52	0.61	2.2248E-06
P26639	Threonine--tRNA ligase 1, cytoplasmic OS=Homo sapiens OX=9606 GN=TARS1 PE=1 SV=3	23	222	12	4.32E+07	3.93E+07	4.30E+07	4.88E+07	4.61E+07	4.06E+07	3.86E+07	4.34E+07	4.10E+07	3.19E+07	4.05E+07	3.41E+07	4.35E+07	3.83E+07	1.14	0.19	0.045553176
P15121	Aldo-keto reductase family 1 member B1 OS=Homo sapiens OX=9606 GN=AKR1B1 PE=1 SV=3	49	268	12	8.76E+07	1.03E+08	8.75E+07	9.73E+07	9.47E+07	9.66E+07	9.82E+07	1.15E+08	8.47E+07	9.71E+07	9.87E+07	8.90E+07	9.45E+07	9.71E+07	0.97	-0.04	0.61722682
Q15008	26S proteasome non-ATPase regulatory subunit 6 OS=Homo sapiens OX=9606 GN=PSMD6 PE=1 SV=1	42	116	12	2.64E+07	3.90E+07	2.47E+07	3.05E+07	2.83E+07	2.53E+07	1.93E+07	1.36E+07	1.43E+07	1.54E+07	1.60E+07	1.19E+07	2.90E+07	1.51E+07	1.93	0.94	0.000603031
P11413	Glucose-6-phosphate 1-dehydrogenase OS=Homo sapiens OX=9606 GN=G6PD PE=1 SV=4	25	203	12	4.16E+07	4.05E+07	3.89E+07	4.38E+07	3.95E+07	3.70E+07	3.85E+07	3.37E+07	3.71E+07	3.46E+07	3.33E+07	3.05E+07	4.02E+07	3.46E+07	1.16	0.22	0.0041236
P50995	Annexin A11 OS=Homo sapiens OX=9606 GN=ANXA11 PE=1 SV=1	27	215	12	6.26E+07	5.86E+07	5.42E+07	5.74E+07	5.20E+07	5.20E+07	5.72E+07	5.51E+07	4.70E+07	4.53E+07	4.33E+07	4.74E+07	5.61E+07	4.92E+07	1.14	0.19	0.037287364
P62269	40S ribosomal protein S18 OS=Homo sapiens OX=9606 GN=RPS18 PE=1 SV=3	51	302	12	9.69E+07	9.40E+07	7.63E+07	9.97E+07	9.21E+07	8.33E+07	7.80E+07	7.73E+07	7.84E+07	8.40E+07	6.94E+07	5.91E+07	9.04E+07	7.44E+07	1.22	0.28	0.01061015
P60842	Eukaryotic initiation factor 4A-I OS=Homo sapiens OX=9606 GN=EIF4A1 PE=1 SV=1	73	595	13	2.85E+08	3.39E+08	2.85E+08	3.00E+08	2.88E+08	3.15E+08	2.00E+08	2.10E+08	2.03E+08	2.02E+08	2.16E+08	2.25E+08	3.02E+08	2.09E+08	1.44	0.53	2.91951E-05
P01889	HLA class I histocompatibility antigen, B alpha chain OS=Homo sapiens OX=9606 GN=HLA-B PE=1 SV=3	57	672	13	4.13E+08	4.02E+08	3.71E+08	4.12E+08	3.90E+08	3.62E+08	1.84E+08	1.76E+08	1.64E+08	1.67E+08	1.61E+08	1.43E+08	3.92E+08	1.66E+08	2.36	1.24	7.51874E-09
Q9H4M9	EH domain-containing protein 1 OS=Homo sapiens OX=9606 GN=EHD1 PE=1 SV=2	54	379	13	1.16E+08	1.15E+08	1.08E+08	1.08E+08	1.08E+08	1.02E+08	1.23E+08	1.33E+08	1.11E+08	1.19E+08	1.14E+08	1.05E+08	1.10E+08	1.18E+08	0.93	-0.10	0.116809906
P63010	AP-2 complex subunit beta OS=Homo sapiens OX=9606 GN=AP2B1 PE=1 SV=1	29	277	13	7.94E+07	7.43E+07	8.73E+07	7.45E+07	8.36E+07	7.78E+07	5.87E+07	4.70E+07	5.57E+07	4.15E+07	5.58E+07	4.36E+07	7.95E+07	5.04E+07	1.58	0.66	2.08862E-05
Q9UMS4	Pre-mRNA-processing factor 19 OS=Homo sapiens OX=9606 GN=PRPF19 PE=1 SV=1	44	208	13	5.98E+07	6.56E+07	7.82E+07	6.10E+07	6.77E+07	6.94E+07	4.45E+07	4.92E+07	4.27E+07	4.54E+07	5.11E+07	4.54E+07	6.69E+07	4.64E+07	1.44	0.53	0.000229779
O00299	Chloride intracellular channel protein 1 OS=Homo sapiens OX=9606 GN=CLIC1 PE=1 SV=4	69	326	13	1.06E+08	1.06E+08	7.33E+07	9.03E+07	8.58E+07	8.89E+07	1.04E+08	9.73E+07	7.42E+07	8.60E+07	7.60E+07	7.32E+07	9.18E+07	8.52E+07	1.08	0.11	0.391998483
P27105	Erythrocyte band 7 integral membrane protein OS=Homo sapiens OX=9606 GN=STOM PE=1 SV=3	61	353	13	1.05E+08	1.14E+08	9.16E+07	1.33E+08	1.07E+08	9.77E+07	9.19E+07	9.65E+07	9.31E+07	8.83E+07	9.58E+07	1.08E+08	1.08E+08	9.56E+07	1.13	0.18	0.094139126
O14980	Exportin-1 OS=Homo sapiens OX=9606 GN=XPO1 PE=1 SV=1	21	209	13	4.68E+07	4.47E+07	4.53E+07	4.57E+07	5.30E+07	4.11E+07	3.25E+07	2.32E+07	2.40E+07	2.56E+07	2.85E+07	2.17E+07	4.61E+07	2.59E+07	1.78	0.83	4.48877E-06
Q08380	Galectin-3-binding protein OS=Homo sapiens OX=9606 GN=LGALS3BP PE=1 SV=1	26	605	13	1.88E+08	1.93E+08	1.44E+08	1.56E+08	1.43E+08	1.57E+08	2.39E+08	2.15E+08	2.02E+08	1.87E+08	1.81E+08	1.79E+08	1.63E+08	2.01E+08	0.81	-0.30	0.016795738
P05121	Plasminogen activator inhibitor 1 OS=Homo sapiens OX=9606 GN=SERPINE1 PE=1 SV=1	39	389	13	1.16E+08	1.21E+08	1.11E+08	1.04E+08	1.04E+08	1.13E+08	1.66E+08	1.60E+08	1.65E+08	1.35E+08	1.54E+08	1.10E+08	1.11E+08	1.48E+08	0.75	-0.41	0.00813218
P04792	Heat shock protein beta-1 OS=Homo sapiens OX=9606 GN=HSPB1 PE=1 SV=2	75	371	13	1.22E+08	1.24E+08	9.38E+07	9.80E+07	8.79E+07	9.16E+07	8.13E+07	8.75E+07	6.49E+07	6.39E+07	6.08E+07	6.28E+07	1.03E+08	7.02E+07	1.47	0.55	0.002675706
Q14254	Flotillin-2 OS=Homo sapiens OX=9606 GN=FLOT2 PE=1 SV=2	35	169	13	2.30E+07	3.36E+07	3.18E+07	3.16E+07	2.73E+07	3.60E+07	4.88E+07	5.50E+07	4.68E+07	4.03E+07	4.07E+07	4.53E+07	3.05E+07	4.61E+07	0.66	-0.60	0.000376662
P12259	Coagulation factor V OS=Homo sapiens OX=9606 GN=F5 PE=1 SV=4	6	583	13	2.64E+08	2.73E+08	2.70E+08	2.61E+08	2.96E+08	2.73E+08	3.12E+08	3.12E+08	2.66E+08	3.20E+08	3.04E+08	2.47E+08	2.73E+08	2.93E+08	0.93	-0.10	0.16884312
O00567	Nucleolar protein 56 OS=Homo sapiens OX=9606 GN=NOP56 PE=1 SV=4	25	159	13	5.71E+07	5.30E+07	4.29E+07	5.53E+07	4.92E+07	5.08E+07	3.90E+07	3.44E+07	3.24E+07	3.04E+07	3.19E+07	2.87E+07	5.14E+07	3.28E+07	1.57	0.65	4.16516E-05
P04114	Apolipoprotein B-100 OS=Homo sapiens OX=9606 GN=APOB PE=1 SV=2	3	448	13	1.12E+08	1.24E+08	1.40E+08	1.30E+08	1.30E+08	1.10E+08	1.62E+08	1.56E+08	1.79E+08	1.77E+08	1.66E+08	1.40E+08	1.24E+08	1.63E+08	0.76	-0.40	0.000466927

Supplementary Table 4. List of all proteins identified by label free proteomics analysis.

Accession	Description	Coverage [%]	# PSMs	# Unique Peptides	Amitriptyline 1	Amitriptyline 2	Amitriptyline 3	Amitriptyline 4	Amitriptyline 5	Amitriptyline 6	Vehicle 1	Vehicle 2	Vehicle 3	Vehicle 4	Vehicle 5	Vehicle 6	Average amitriptyline	Average vehicle	Ratio amitriptyline/ vehicle	Log 2 fold change (amitriptyline /vehicle)	P-value
Q9Y3I0	tRNA-splicing ligase RtcB homolog OS=Homo sapiens OX=9606 GN=RTCB PE=1 SV=1	39	174	13	4.43E+07	4.57E+07	4.55E+07	4.29E+07	3.63E+07	4.20E+07	3.60E+07	3.78E+07	3.16E+07	2.88E+07	3.30E+07	2.99E+07	4.28E+07	3.28E+07	1.30	0.38	0.000582275
O00622	CCN family member 1 OS=Homo sapiens OX=9606 GN=CCN1 PE=1 SV=1	36	309	13	6.61E+07	5.16E+07	5.93E+07	6.32E+07	6.69E+07	6.31E+07	1.39E+08	1.39E+08	1.38E+08	1.49E+08	1.14E+08	1.28E+08	6.17E+07	1.35E+08	0.46	-1.13	2.82828E-06
O95373	Importin-7 OS=Homo sapiens OX=9606 GN=IPO7 PE=1 SV=1	17	108	13	2.99E+07	3.47E+07	2.87E+07	2.90E+07	3.48E+07	3.36E+07	2.41E+07	1.89E+07	2.26E+07	1.96E+07	2.61E+07	2.17E+07	3.18E+07	2.22E+07	1.43	0.52	0.000149478
O00231	26S proteasome non-ATPase regulatory subunit 11 OS=Homo sapiens OX=9606 GN=PSMD11 PE=1 SV=3	44	282	13	5.68E+07	5.65E+07	5.48E+07	5.72E+07	5.47E+07	5.32E+07	7.87E+07	4.10E+07	3.80E+07	3.92E+07	3.78E+07	3.19E+07	5.55E+07	4.44E+07	1.25	0.32	0.171866531
Q15063	Periostin OS=Homo sapiens OX=9606 GN=POSTN PE=1 SV=2	19	142	13	3.71E+07	3.23E+07	3.30E+07	2.89E+07	2.73E+07	3.27E+07	5.11E+07	4.86E+07	3.23E+07	3.92E+07	4.29E+07	3.78E+07	3.19E+07	4.20E+07	0.76	-0.40	0.015345511
Q99460	26S proteasome non-ATPase regulatory subunit 1 OS=Homo sapiens OX=9606 GN=PSMD1 PE=1 SV=2	21	166	13	4.70E+07	5.05E+07	4.69E+07	4.76E+07	4.21E+07	4.40E+07	3.77E+07	3.78E+07	2.97E+07	3.23E+07	3.17E+07	2.58E+07	4.64E+07	3.25E+07	1.43	0.51	0.000219888
P55786	Puromycin-sensitive aminopeptidase OS=Homo sapiens OX=9606 GN=NPEPPS PE=1 SV=2	21	168	13	3.89E+07	4.96E+07	3.77E+07	4.32E+07	3.92E+07	4.45E+07	4.48E+07	4.57E+07	4.64E+07	4.05E+07	4.73E+07	3.96E+07	4.22E+07	4.40E+07	0.96	-0.06	0.430260256
P12429	Annexin A3 OS=Homo sapiens OX=9606 GN=ANXA3 PE=1 SV=3	44	233	13	7.70E+07	7.38E+07	6.16E+07	6.67E+07	7.53E+07	6.37E+07	6.64E+07	6.68E+07	6.38E+07	5.80E+07	5.76E+07	4.89E+07	6.97E+07	6.03E+07	1.16	0.21	0.035041928
Q9Y230	RuvB-like 2 OS=Homo sapiens OX=9606 GN=RUVBL2 PE=1 SV=3	30	152	13	4.60E+07	4.63E+07	4.38E+07	4.80E+07	5.04E+07	4.38E+07	3.20E+07	3.20E+07	2.90E+07	3.15E+07	2.89E+07	2.48E+07	4.64E+07	2.97E+07	1.56	0.64	7.89841E-07
Q86UX7	Fermitin family homolog 3 OS=Homo sapiens OX=9606 GN=FERMT3 PE=1 SV=1	22	216	13	5.65E+07	6.21E+07	5.95E+07	6.33E+07	5.82E+07	5.49E+07	8.40E+07	7.88E+07	7.89E+07	7.84E+07	7.93E+07	7.97E+07	5.91E+07	7.98E+07	0.74	-0.43	5.14872E-07
P60228	Eukaryotic translation initiation factor 3 subunit E OS=Homo sapiens OX=9606 GN=EIF3E PE=1 SV=1	35	209	13	5.87E+07	6.31E+07	6.35E+07	6.71E+07	6.77E+07	5.83E+07	4.81E+07	3.68E+07	4.52E+07	4.42E+07	4.50E+07	4.00E+07	6.31E+07	4.32E+07	1.46	0.55	6.81537E-06
P62906	60S ribosomal protein L10a OS=Homo sapiens OX=9606 GN=RPL10A PE=1 SV=2	62	358	14	2.20E+08	2.45E+08	2.28E+08	2.40E+08	2.31E+08	2.09E+08	1.80E+08	1.81E+08	1.54E+08	1.60E+08	1.59E+08	1.50E+08	2.29E+08	1.64E+08	1.40	0.48	7.01911E-06
Q14974	Importin subunit beta-1 OS=Homo sapiens OX=9606 GN=KPNB1 PE=1 SV=2	24	282	14	7.64E+07	7.15E+07	6.88E+07	7.08E+07	7.82E+07	6.72E+07	6.22E+07	4.97E+07	4.71E+07	4.24E+07	5.28E+07	3.88E+07	7.22E+07	4.88E+07	1.48	0.56	0.000349151
P39023	60S ribosomal protein L3 OS=Homo sapiens OX=9606 GN=RPL3 PE=1 SV=2	43	274	14	1.15E+08	1.15E+08	1.16E+08	1.11E+08	1.16E+08	1.11E+08	7.18E+07	6.26E+07	5.41E+07	6.76E+07	5.72E+07	5.83E+07	1.14E+08	6.19E+07	1.84	0.88	1.36959E-06
P11021	Endoplasmic reticulum chaperone BiP OS=Homo sapiens OX=9606 GN=HSPA5 PE=1 SV=2	31	323	14	4.66E+07	4.68E+07	4.07E+07	3.68E+07	3.89E+07	3.58E+07	4.08E+07	3.79E+07	2.87E+07	3.07E+07	2.97E+07	3.20E+07	4.09E+07	3.33E+07	1.23	0.30	0.021029702
P06493	Cyclin-dependent kinase 1 OS=Homo sapiens OX=9606 GN=CDK1 PE=1 SV=3	56	238	14	8.99E+07	9.42E+07	8.03E+07	8.18E+07	9.00E+07	8.91E+07	4.63E+07	5.46E+07	4.57E+07	4.38E+07	5.08E+07	4.92E+07	8.75E+07	4.84E+07	1.81	0.85	1.3434E-07
P62753	40S ribosomal protein S6 OS=Homo sapiens OX=9606 GN=RPS6 PE=1 SV=1	40	309	14	1.44E+08	1.61E+08	1.31E+08	1.36E+08	1.22E+08	1.37E+08	7.79E+07	9.17E+07	6.42E+07	7.97E+07	6.74E+07	6.11E+07	1.38E+08	7.37E+07	1.88	0.91	4.80964E-06
P55060	Exportin-2 OS=Homo sapiens OX=9606 GN=CSE1L PE=1 SV=3	22	168	14	4.61E+07	4.33E+07	4.85E+07	4.60E+07	5.06E+07	4.75E+07	3.38E+07	3.37E+07	3.00E+07	3.48E+07	3.39E+07	2.71E+07	4.70E+07	3.22E+07	1.46	0.54	3.9913E-06
P20591	Interferon-induced GTP-binding protein Mx1 OS=Homo sapiens OX=9606 GN=MX1 PE=1 SV=4	32	182	14	6.06E+07	5.34E+07	5.73E+07	5.24E+07	4.74E+07	5.23E+07	2.31E+07	2.33E+07	2.02E+07	1.88E+07	1.76E+07	2.21E+07	5.39E+07	2.08E+07	2.59	1.37	5.1142E-07
P46777	60S ribosomal protein L5 OS=Homo sapiens OX=9606 GN=RPL5 PE=1 SV=3	47	322	14	1.86E+08	1.61E+08	1.38E+08	1.51E+08	1.49E+08	1.48E+08	1.13E+08	1.11E+08	9.69E+07	1.02E+08	9.48E+07	8.27E+07	1.56E+08	1.00E+08	1.55	0.64	8.30677E-05
Q9H223	EH domain-containing protein 4 OS=Homo sapiens OX=9606 GN=EHD4 PE=1 SV=1	42	198	14	5.51E+07	5.49E+07	4.75E+07	4.87E+07	4.77E+07	4.95E+07	3.91E+07	4.00E+07	3.70E+07	3.09E+07	3.21E+07	3.49E+07	5.05E+07	3.57E+07	1.42	0.50	3.27392E-05
P38606	V-type proton ATPase catalytic subunit A OS=Homo sapiens OX=9606 GN=ATP6V1A PE=1 SV=2	38	145	14	3.11E+07	3.34E+07	3.46E+07	3.17E+07	2.93E+07	3.07E+07	3.43E+07	3.31E+07	3.03E+07	2.71E+07	3.14E+07	2.75E+07	3.18E+07	3.06E+07	1.04	0.06	0.423305356
Q92626	Peroxidasin homolog OS=Homo sapiens OX=9606 GN=PXDN PE=1 SV=2	15	136	14	1.61E+07	1.48E+07	1.57E+07	1.62E+07	1.88E+07	1.84E+07	3.94E+07	2.93E+07	2.76E+07	2.46E+07	2.55E+07	2.81E+07	1.67E+07	2.91E+07	0.57	-0.80	0.001656955
O00410	Importin-5 OS=Homo sapiens OX=9606 GN=IPO5 PE=1 SV=4	22	120	14	4.54E+07	3.60E+07	2.72E+07	3.32E+07	2.95E+07	2.81E+07	2.72E+07	1.52E+07	2.04E+07	1.63E+07	1.90E+07	1.51E+07	3.32E+07	1.89E+07	1.76	0.82	0.002178959
P14625	Endoplasmin OS=Homo sapiens OX=9606 GN=HSP90B1 PE=1 SV=1	22	317	14	4.28E+07	4.22E+07	3.45E+07	4.00E+07	3.74E+07	3.51E+07	4.55E+07	4.58E+07	4.23E+07	4.56E+07	4.30E+07	3.85E+07	3.87E+07	4.35E+07	0.89	-0.17	0.027812594
O43854	EGF-like repeat and discoidin I-like domain-containing protein 3 OS=Homo sapiens OX=9606 GN=EDIL3 PE=1 SV=1	35	185	14	8.84E+07	9.20E+07	7.83E+07	9.00E+07	8.49E+07	7.63E+07	1.09E+08	1.12E+08	1.09E+08	1.15E+08	9.30E+07	8.98E+07	8.50E+07	1.05E+08	0.81	-0.30	0.004109821
P35998	26S proteasome regulatory subunit 7 OS=Homo sapiens OX=9606 GN=PSMC2 PE=1 SV=3	42	161	14	4.70E+07	4.55E+07	3.77E+07	4.42E+07	3.87E+07	3.77E+07	3.36E+07	2.94E+07	3.15E+07	3.09E+07	2.28E+07	1.94E+07	4.18E+07	2.80E+07	1.50	0.58	0.000815204
Q5SSJ5	Heterochromatin protein 1-binding protein 3 OS=Homo sapiens OX=9606 GN=HP1BP3 PE=1 SV=1	26	148	14	4.52E+07	4.14E+07	4.68E+07	4.68E+07	4.59E+07	4.16E+07	3.21E+07	3.06E+07	3.33E+07	3.32E+07	3.16E+07	2.17E+07	4.46E+07	3.04E+07	1.47	0.55	0.000125567
P62280	40S ribosomal protein S11 OS=Homo sapiens OX=9606 GN=RPS11 PE=1 SV=3	63	350	14	1.70E+08	1.70E+08	1.69E+08	1.71E+08	1.75E+08	1.52E+08	1.23E+08	1.26E+08	1.24E+08	1.24E+08	1.11E+08	9.68E+07	1.68E+08	1.18E+08	1.43	0.51	1.09494E-05
P11388	DNA topoisomerase 2-alpha OS=Homo sapiens OX=9606 GN=TOP2A PE=1 SV=3	12	94	14	2.05E+07	2.16E+07	2.21E+07	2.14E+07	1.98E+07	1.91E+07	1.93E+07	1.37E+07	1.61E+07	1.73E+07	1.60E+07	1.03E+07	2.08E+07	1.54E+07	1.34	0.43	0.006887621
P07942	Laminin subunit beta-1 OS=Homo sapiens OX=9606 GN=LAMB1 PE=1 SV=2	10	133	14	2.07E+07	2.39E+07	2.72E+07	2.58E+07	2.45E+07	2.63E+07	3.64E+07	3.49E+07	3.43E+07	3.24E+07	3.08E+07	2.67E+07	2.47E+07	3.26E+07	0.76	-0.40	0.00142394
P62826	GTP-binding nuclear protein Ran OS=Homo sapiens OX=9606 GN=RAN PE=1 SV=3	49	719	15	4.54E+08	5.14E+08	4.04E+08	4.17E+08	4.32E+08	4.24E+08	3.87E+08	4.25E+08	4.09E+08	3.57E+08	3.54E+08	3.14E+08	4.41E+08	3.74E+08	1.18	0.24	0.01683434
Q16555	Dihydropyrimidinase-related protein 2 OS=Homo sapiens OX=9606 GN=DPYSL2 PE=1 SV=1	46	280	15	6.41E+07	6.79E+07	6.25E+07	5.95E+07	5.68E+07	6.28E+07	6.77E+07	7.03E+07	7.12E+07	6.42E+07	5.79E+07	5.96E+07	6.23E+07	6.52E+07	0.96	-0.07	0.318854539
P62241	40S ribosomal protein S8 OS=Homo sapiens OX=9606 GN=RPS8 PE=1 SV=2	60	826	15	3.14E+08	3.23E+08	3.32E+08	3.12E+08	3.21E+08	3.45E+08	2.09E+08	2.20E+08	1.95E+08	2.02E+08	1.98E+08	1.75E+08	3.25E+08	2.00E+08	1.62	0.70	4.09199E-08
P62424	60S ribosomal protein L7a OS=Homo sapiens OX=9606 GN=RPL7A PE=1 SV=2	51	367	15	1.63E+08	1.58E+08	1.57E+08	1.65E+08	1.50E+08	1.38E+08	9.09E+07	1.00E+08	8.18E+07	9.68E+07	8.98E+07	7.82E+07	1.55E+08	8.97E+07	1.73	0.79	2.79207E-07
Q9NZM1	Myoferlin OS=Homo sapiens OX=9606 GN=MYOF PE=1 SV=1	11	174	15	4.47E+07	4.29E+07	3.88E+07	4.10E+07	3.92E+07	3.94E+07	3.04E+07	2.42E+07	2.81E+07	2.69E+07	2.43E+07	2.10E+07	4.10E+07	2.58E+07	1.59	0.67	7.72441E-06
Q9UHB9	Signal recognition particle subunit SRP68 OS=Homo sapiens OX=9606 GN=SRP68 PE=1 SV=2	32	139	15	4.23E+07	4.33E+07	3.87E+07	3.92E+07	3.53E+07	3.89E+07	2.15E+07	1.72E+07	1.62E+07	1.78E+07	1.78E+07	1.53E+07	3.96E+07	1.76E+07	2.24	1.17	7.87903E-08
O75955	Flotillin-1 OS=Homo sapiens OX=9606 GN=FLOT1 PE=1 SV=3	38	175	15	3.40E+07	3.62E+07	3.73E+07	4.10E+07	4.21E+07	3.79E+07	5.37E+07	5.26E+07	5.08E+07	5.00E+07	4.72E+07	4.49E+07	3.81E+07	4.99E+07	0.76	-0.39	7.60348E-05
O75891	Cytosolic 10-formyltetrahydrofolate dehydrogenase OS=Homo sapiens OX=9606 GN=ALDH1L1 PE=1 SV=2	19	425	15	1.97E+08	2.08E+08	1.74E+08	1.85E+08	1.70E+08	1.63E+08	2.51E+08	2.65E+08	2.09E+08	2.36E+08	2.15E+08	2.03E+08	1.83E+08	2.30E+08	0.80	-0.33	0.004454169
P02786	Transferrin receptor protein 1 OS=Homo sapiens OX=9606 GN=TFRC PE=1 SV=2	24	313	15	7.51E+07	7.97E+07	7.55E+07	7.74E+07	6.29E+07	6.89E+07	8.12E+07	6.92E+07	9.12E+07	9.10E+07	9.38E+07	5.93E+07	7.32E+07	8.10E+07	0.90	-0.14	0.25765062
Q9Y6K5	2'-5'-oligoadenylate synthase 3 OS=Homo sapiens OX=9606 GN=OAS3 PE=1 SV=3	22	134	15	3.30E+07	3.44E+07	2.69E+07	3.10E+07	2.87E+07	2.98E+07	2.43E+07	2.10E+07	1.92E+07	1.83E+07	2.02E+07	1.56E+07	3.06E+07	1.98E+07	1.55	0.63	5.8463E-05
Q9UNM6	26S proteasome non-ATPase regulatory subunit 13 OS=Homo sapiens OX=9606 GN=PSMD13 PE=1 SV=2	45	180	15	6.48E+07	6.22E+07	6.07E+07	6.30E+07	6.85E+07	5.67E+07	4.65E+07	4.48E+07	4.85E+07	5.50E+07	4.34E+07	3.85E+07	6.27E+07	4.61E+07	1.36	0.44	0.000198056
Q15393	Splicing factor 3B subunit 3 OS=Homo sapiens OX=9606 GN=SF3B3 PE=1 SV=4	15	180	15	7.23E+07	7.29E+07	6.35E+07	6.96E+07	6.52E+07	6.38E+07	4.27E+07	4.36E+07	4.69E+07	4.33E+07	3.89E+07	3.81E+07	6.79E+07	4.23E+07	1.61	0.68	6.47687E-07
P14868	Aspartate--tRNA ligase, cytoplasmic OS=Homo sapiens OX=9606 GN=DARS PE=1 SV=2	33	282	15	9.72E+07	1.06E+08	8.81E+07	8.51E+07	9.17E+07	8.53E+07	8.01E+07	6.84E+07	7.53E+07	6.55E+07	7.07E+07	5.23E+07	9.22E+07	6.87E+07	1.34	0.42	0.001086047
O43242	26S proteasome non-ATPase regulatory subunit 3 OS=Homo sapiens OX=9606 GN=PSMD3 PE=1 SV=2	36	207	15	4.80E+07	5.86E+07	5.84E+07	4.98E+07	4.68E+07	5.16E+07	3.38E+07	3.84E+07	3.65E+07	3.87E+07	3.91E+07	3.31E+07	5.22E+07	3.66E+07	1.43	0.51	0.000234261
P43686	26S proteasome regulatory subunit 6B OS=Homo sapiens OX=9606 GN=PSMC4 PE=1 SV=2	53	319	16	7.50E+07	7.84E+07	7.71E+07	7.54E+07	8.29E+07	7.85E+07	5.32E+07	5.85E+07	5.37E+07	5.11E+07	5.49E+07	4.76E+07	7.79E+07	5.32E+07	1.46	0.55	2.35414E-07
P30153	Serine/threonine-protein phosphatase 2A 65 kDa regulatory subunit A alpha isoform OS=Homo sapiens OX=9606 GN=PPP2R1A PE=1 SV=4	31	219	16	7.16E+07	5.21E+07	7.03E+07	6.15E+07	5.44E+07	6.33E+07	6.39E+07	5.85E+07	5.35E+07	5.37E+07	6.96E+07	5.29E+07	6.22E+07	5.87E+07	1.06	0.08	0.429990125
P54136	Arginine--tRNA ligase, cytoplasmic OS=Homo sapiens OX=9606 GN=RARS PE=1 SV=2	26	193	16	5.75E+07	6.11E+07	5.41E+07	5.27E+07	5.84E+07	5.20E+07	3.65E+07	3.79E+07	2.92E+07	3.25E+07	3.36E+07	2.71E+07	5.60E+07	3.28E+07	1.71	0.77	1.34381E-06
P26038	Moesin OS=Homo sapiens OX=9606 GN=MSN PE=1 SV=3	41	346	16	1.34E+08	1.31E+08	1.01E+08	1.05E+08	9.52E+07	1.01E+08	1.07E+08	1.06E+08	8.74E+07	8.81E+07	8.25E+07	8.84E+07	1.11E+08	9.32E+07	1.19	0.26	0.053169262
Q01813	ATP-dependent 6-phosphofructokinase, platelet type OS=Homo sapiens OX=9606 GN=PFKP PE=1 SV=2	26	199	16	7.24E+07	7.37E+07	6.31E+07	6.70E+07	5.99E+07	6.55E+07	5.45E+07	5.24E+07	4.80E+07	4.93E+07	5.65E+07	4.48E+07	6.69E+07	5.09E+07	1.31	0.39	0.000227945
P41250	Glycine--tRNA ligase OS=Homo sapiens OX=9606 GN=GARS1 PE=1 SV=3	29	132	16	4.50E+07	4.63E+07	4.81E+07	4.60E+07	4.20E+07	4.48E+07	3.43E+07	2.82E+07	2.75E+07	2.85E+07	2.77E+07	2.47E+07	4.53E+07	2.85E+07	1.59	0.67	2.40513E-06
P23921	Ribonucleoside-diphosphate reductase large subunit OS=Homo sapiens OX=9606 GN=RRM1 PE=1 SV=1	28	216	16	6.58E+07	6.47E+07	5.27E+07	5.71E+07	5.95E+07	5.04E+07	7.02E+07	6.32E+07	6.82E+07	6.70E+07	5.59E+07	6.68E+07	5.84E+07	6.52E+07	0.89	-0.16	0.064327183
P34897	Serine hydroxymethyltransferase, mitochondrial OS=Homo sapiens OX=9606 GN=SHMT2 PE=1 SV=3	47	310	16	1.14E+08	1.28E+08	1.04E+08	1.07E+08	1.08E+08	1.10E+08	8.45E+07	9.95E+07	7.82E+07	8.56E+07	8.80E+07	7.78E+07	1.12E+08	8.56E+07	1.31	0.39	0.000280862
P21980	Protein-glutamine gamma-glutamyltransferase 2 OS=Homo sapiens OX=9606 GN=TGM2 PE=1 SV=2	33	275	16	7.16E+07	5.84E+07	5.17E+07	5.69E+07	5.78E+07	5.73E+07	4.59E+07	4.60E+07	3.57E+07	3.68E+07	4.94E+07	2.91E+07	5.90E+07	4.05E+07	1.46	0.54	0.001392039
Q99613	Eukaryotic translation initiation factor 3 subunit C OS=Homo sapiens OX=9606 GN=EIF3C PE=1 SV=1	18	288	16	7.68E+07	7.62E+07	7.21E+07	7.61E+07	7.00E+07	6.81E+07	4.90E+07	4.55E+07	4.10E+07	4.48E+07	3.76E+07	3.46E+07	7.32E+07	4.21E+07	1.74	0.80	1.14604E-06
P49588	Alanine--tRNA ligase, cytoplasmic OS=Homo sapiens OX=9606 GN=AARS PE=1 SV=2	22	231	16	6.97E+07	6.58E+07	7.05E+07	7.06E+07	7.12E+07	6.47E+07	4.60E+07	4.87E+07	4.47E+07	5.01E+07	4.85E+07	4.12E+07	6.87E+07	4.65E+07	1.48	0.56	2.26638E-07
P24821	Tenascin OS=Homo sapiens OX=9606 GN=TNC PE=1 SV=3	11	200	16	1.96E+07	1.70E+07	2.56E+07	3.14E+07	2.08E+07	2.54E+07	5.46E+07	5.10E+07	5.26E+07	4.48E+07	4.26E+07	4.44E+07	2.33E+07	4.83E+07	0.48	-1.05	6.92534E-06
P05362	Intercellular adhesion molecule 1 OS=Homo sapiens OX=9606 GN=ICAM1 PE=1 SV=2	25	306	16	1.23E+08	1.26E+08	1.26E+08	1.30E+08	1.12E+08	1.21E+08	7.09E+07	8.23E+07	6.40E+07	6.27E+07	5.83E+07	5.82E+07	1.23E+08	6.61E+07	1.86	0.90	6.72191E-07
O43776	Asparagine--tRNA ligase, cytoplasmic OS=Homo sapiens OX=9606 GN=NARS1 PE=1 SV=1	30	187	16	6.19E+07	6.22E+07	5.71E+07	6.09E+07	6.18E+07	5.35E+07	5.16E+07	4.70E+07	4.68E+07	5.10E+07	4.30E+07	3.45E+07	5.96E+07	4.56E+07	1.31	0.38	0.001570899
P29317	Ephrin type-A receptor 2 OS=Homo sapiens OX=9606 GN=EPHA2 PE=1 SV=2	18	186	16	5.57E+07	5.87E+07	5.38E+07	6.14E+07	5.78E+07	5.49E+07	4.08E+07	4.15E+07	4.29E+07	4.53E+07	4.40E+07	3.62E+07	5.71E+07	4.18E+07	1.37	0.45	5.66128E-06
P02545	Prelamin-A/C OS=Homo sapiens OX=9606 GN=LMNA PE=1 SV=1	28	169	16	5.94E+07	6.30E+07	6.27E+07	7.17E+07	6.45E+07	5.95E+07	3.80E+07	3.95E+07	3.95E+07	4.47E+07	3.95E+07	3.14E+07	6.35E+07	3.88E+07	1.64	0.71	2.08857E-06
P42224	Signal transducer and activator of transcription 1-alpha/beta OS=Homo sapiens OX=9606 GN=STAT1 PE=1 SV=2	28	185	16	4.36E+07	4.76E+07	4.12E+07	3.82E+07	4.69E+07	3.63E+07	3.51E+07	3.53E+07	2.80E+07	2.81E+07	2.84E+07	2.33E+07	4.23E+07	2.97E+07	1.42	0.51	0.000833302
P21589	5'-nucleotidase OS=Homo sapiens OX=9606 GN=NT5E PE=1 SV=1	41	398	17	1.63E+08	1.53E+08	1.30E+08	1.28E+08	1.15E+08	1.33E+08	1.08E+08	9.56E+07	7.62E+07	7.37E+07	8.00E+07	7.90E+07	1.37E+08	8.54E+07	1.60	0.68	0.000237758
P36578	60S ribosomal protein L4 OS=Homo sapiens OX=9606 GN=RPL4 PE=1 SV=5	39	429	17	1.72E+08	1.76E+08	1.43E+08	1.54E+08	1.38E+08	1.57E+08	7.92E+07	8.11E+07	6.72E+07	6.71E+07	6.46E+07	7.49E+07	1.57E+08	7.24E+07	2.17	1.11	5.20448E-06
P0C0L4	Complement C4-A OS=Homo sapiens OX=9606 GN=C4A PE=1 SV=2	11	968	17	5.92E+08	6.03E+08	5.22E+08	5.78E+08	5.62E+08	4.73E+08	5.90E+08	6.16E+08	5.40E+08	5.93E+08	4.99E+08	5.61E+08	5.55E+08	5.66E+08	0.98	-0.03	0.676630844
P49915	GMP synthase [glutamine-hydrolyzing] OS=Homo sapiens OX=9606 GN=GMPS PE=1 SV=1	30	207	17	5.12E+07	5.58E+07	4.69E+07	4.72E+07	4.65E+07	4.39E+07	3.42E+07	3.64E+07	3.36E+07	3.25E+07	3.48E+07	2.80E+07	4.86E+07	3.33E+07	1.46	0.55	4.9949E-05
Q96QK1	Vacuolar protein sorting-associated protein 35 OS=Homo sapiens OX=9606 GN=VPS35 PE=1 SV=2	29	192	17	4.67E+07	5.10E+07	4.89E+07	4.20E+07	4.93E+07	4.31E+07	4.66E+07	4.13E+07	4.28E+07	4.01E+07	4.14E+07	3.29E+07	4.68E+07	4.08E+07	1.15	0.20	0.029799799
P26006	Integrin alpha-3 OS=Homo sapiens OX=9606 GN=ITGA3 PE=1 SV=5	19	317	17	1.48E+08	1.44E+08	1.23E+08	1.29E+08	1.24E+08	1.21E+08	8.25E+07	7.90E+07	7.74E+07	8.81E+07	6.92E+07	6.20E+07	1.31E+08	7.64E+07	1.72	0.78	5.53512E-06
Q9Y678	Coatomer subunit gamma-1 OS=Homo sapiens OX=9606 GN=COPG1 PE=1 SV=1	32	163	17	5.11E+07	4.68E+07	4.79E+07	4.44E+07	4.54E+07	3.68E+07	2.86E+07	2.72E+07	2.42E+07	2.45E+07	2.47E+07	1.82E+07	4.54E+07	2.46E+07	1.85	0.89	1.17894E-05
P12111	Collagen alpha-3(VI) chain OS=Homo sapiens OX=9606 GN=COL6A3 PE=1 SV=5	6	293	17	5.72E+07	6.40E+07	5.34E+07	5.48E+07	4.79E+07	5.71E+07	8.72E+07	9.68E+07	8.09E+07	7.42E+07	7.49E+07	7.96E+07	5.57E+07	8.23E+07	0.68	-0.56	0.00015959
P49748	Very long-chain specific acyl-CoA dehydrogenase, mitochondrial OS=Homo sapiens OX=9606 GN=ACADVL PE=1 SV=1	29	174	17	6.79E+07	6.19E+07	5.16E+07	5.07E+07	4.53E+07	4.67E+07	3.73E+07	3.75E+07	3.09E+07	3.14E+07	3.16E+07	2.76E+07	5.40E+07	3.27E+07	1.65	0.72	0.001139755
P18124	60S ribosomal protein L7 OS=Homo sapiens OX=9606 GN=RPL7 PE=1 SV=1	57	391	17	1.59E+08	1.54E+08	1.26E+08	1.49E+08	1.37E+08	1.42E+08	8.82E+07	8.95E+07	9.47E+07	8.31E+07	8.01E+07	7.12E+07	1.44E+08	8.45E+07	1.71	0.77	3.6428E-06
P04083	Annexin A1 OS=Homo sapiens OX=9606 GN=ANXA1 PE=1 SV=2	53	523	18	2.61E+08	2.35E+08	2.30E+08	2.19E+08	2.16E+08	2.25E+08	2.32E+08	2.14E+08	2.01E+08	1.94E+08	1.92E+08	1.58E+08	2.31E+08	1.98E+08	1.16	0.22	0.02539966
Q01518	Adenylyl cyclase-associated protein 1 OS=Homo sapiens OX=9606 GN=CAP1 PE=1 SV=5	47	368	18	1.33E+08	1.15E+08	1.17E+08	1.30E+08	1.18E+08	1.25E+08	1.07E+08	1.10E+08	9.96E+07	1.06E+08	1.02E+08	9.41E+07	1.23E+08	1.03E+08	1.19	0.25	0.000571893
P26641	Elongation factor 1-gamma OS=Homo sapiens OX=9606 GN=EEF1G PE=1 SV=3	67	491	18	2.15E+08	2.12E+08	2.06E+08	2.04E+08	2.08E+08	1.98E+08	2.09E+08	2.10E+08	2.02E+08	2.22E+08	1.97E+08	1.74E+08	2.07E+08	2.02E+08	1.02	0.03	0.515144864
P63244	Receptor of activated protein C kinase 1 OS=Homo sapiens OX=9606 GN=RACK1 PE=1 SV=3	81	426	18	1.58E+08	1.39E+08	1.24E+08	1.19E+08	1.18E+08	1.36E+08	9.15E+07	8.32E+07	8.59E+07	7.43E+07	8.02E+07	8.36E+07	1.32E+08	8.31E+07	1.59	0.67	0.000235396
P61158	Actin-related protein 3 OS=Homo sapiens OX=9606 GN=ACTR3 PE=1 SV=3	59	274	18	8.41E+07	6.45E+07	5.76E+07	6.88E+07	7.29E+07	6.20E+07	6.67E+07	7.04E+07	6.46E+07	5.64E+07	5.58E+07	5.45E+07	6.83E+07	6.14E+07	1.11	0.15	0.174091944
P61247	40S ribosomal protein S3a OS=Homo sapiens OX=9606 GN=RPS3A PE=1 SV=2	56	333	18	2.54E+08	2.74E+08	2.23E+08	2.39E+08	2.38E+08	2.26E+08	2.02E+08	2.25E+08	2.03E+08	1.95E+08	1.73E+08	1.67E+08	2.42E+08	1.94E+08	1.25	0.32	0.002070637
O75083	WD repeat-containing protein 1 OS=Homo sapiens OX=9606 GN=WDR1 PE=1 SV=4	46	529	19	2.27E+08	2.39E+08	2.07E+08	1.89E+08	1.91E+08	1.84E+08	2.53E+08	2.35E+08	2.15E+08	2.25E+08	2.22E+08	1.80E+08	2.06E+08	2.22E+08	0.93	-0.10	0.276843875
P00558	Phosphoglycerate kinase 1 OS=Homo sapiens OX=9606 GN=PGK1 PE=1 SV=3	55	634	19	2.78E+08	2.88E+08	2.29E+08	2.47E+08	2.17E+08	2.49E+08	2.96E+08	3.25E+08	2.30E+08	2.42E+08	2.45E+08	2.44E+08	2.51E+08	2.64E+08	0.95	-0.07	0.534270937
Q00839	Heterogeneous nuclear ribonucleoprotein U OS=Homo sapiens OX=9606 GN=HNRNPU PE=1 SV=6	29	335	19	1.53E+08	1.35E+08	1.24E+08	1.56E+08	1.37E+08	1.39E+08	7.50E+07	7.96E+07	6.56E+07	6.37E+07	6.21E+07	5.58E+07	1.41E+08	6.70E+07	2.10	1.07	5.55736E-07
P56192	Methionine--tRNA ligase, cytoplasmic OS=Homo sapiens OX=9606 GN=MARS PE=1 SV=2	33	264	19	8.06E+07	8.28E+07	7.22E+07	7.27E+07	7.33E+07	7.32E+07	4.71E+07	4.93E+07	3.60E+07	3.88E+07	3.80E+07	3.58E+07	7.58E+07	4.08E+07	1.86	0.89	7.18303E-07
O60701	UDP-glucose 6-dehydrogenase OS=Homo sapiens OX=9606 GN=UGDH PE=1 SV=1	52	330	19	8.34E+07	8.77E+07	8.54E+07	8.43E+07	8.85E+07	9.67E+07	1.06E+08	9.84E+07	8.79E+07	8.68E+07	1.07E+08	8.84E+07	8.77E+07	9.58E+07	0.92	-0.13	0.097750282
Q06210	Glutamine--fructose-6-phosphate aminotransferase [isomerizing] 1 OS=Homo sapiens OX=9606 GN=GFPT1 PE=1 SV=3	36	282	19	7.70E+07	8.02E+07	6.49E+07	6.94E+07	6.86E+07	6.49E+07	7.82E+07	8.24E+07	7.38E+07	7.36E+07	7.13E+07	6.18E+07	7.08E+07	7.35E+07	0.96	-0.05	0.503681628
O00468	Agrin OS=Homo sapiens OX=9606 GN=AGRN PE=1 SV=6	15	192	19	2.31E+07	2.74E+07	2.19E+07	2.45E+07	2.58E+07	2.27E+07	4.79E+07	4.69E+07	4.27E+07	3.81E+07	3.62E+07	3.69E+07	2.43E+07	4.15E+07	0.59	-0.77	0.00016833
P11387	DNA topoisomerase 1 OS=Homo sapiens OX=9606 GN=TOP1 PE=1 SV=2	26	438	19	1.46E+08	1.52E+08	1.60E+08	1.47E+08	1.56E+08	1.38E+08	1.48E+08	1.57E+08	1.60E+08	1.41E+08	1.38E+08	1.23E+08	1.50E+08	1.45E+08	1.04	0.05	0.441249888
P26640	Valine--tRNA ligase OS=Homo sapiens OX=9606 GN=VARS PE=1 SV=4	22	193	19	3.58E+07	3.63E+07	3.99E+07	3.63E+07	3.79E+07	3.53E+07	2.61E+07	2.77E+07	2.82E+07	2.49E+07	2.38E+07	2.34E+07	3.69E+07	2.57E+07	1.44	0.52	1.25035E-06
P62805	Histone H4 OS=Homo sapiens OX=9606 GN=H4C1 PE=1 SV=2	65	9740	20	1.16E+10	1.10E+10	1.35E+10	1.27E+10	1.27E+10	1.27E+10	1.17E+10	1.08E+10	1.32E+10	1.31E+10	1.27E+10	1.27E+10	1.24E+10	1.24E+10	1.00	0.00	0.963183175
P11216	Glycogen phosphorylase, brain form OS=Homo sapiens OX=9606 GN=PYGB PE=1 SV=5	42	611	20	8.74E+07	9.30E+07	1.00E+08	9.44E+07	9.24E+07	9.41E+07	8.76E+07	9.36E+07	8.52E+07	8.79E+07	8.36E+07	7.14E+07	9.36E+07	8.49E+07	1.10	0.14	0.037576908
O75367	Core histone macro-H2A.1 OS=Homo sapiens OX=9606 GN=H2AFY PE=1 SV=4	60	714	20	3.84E+08	4.43E+08	3.23E+08	3.69E+08	3.55E+08	4.38E+08	4.56E+08	5.55E+08	3.89E+08	3.67E+08	4.70E+08	3.90E+08	3.85E+08	4.38E+08	0.88	-0.18	0.16388985
P08670	Vimentin OS=Homo sapiens OX=9606 GN=VIM PE=1 SV=4	45	354	20	1.29E+08	1.35E+08	1.33E+08	1.46E+08	1.59E+08	1.33E+08	6.43E+07	6.37E+07	5.97E+07	6.08E+07	6.44E+07	5.39E+07	1.39E+08	6.11E+07	2.28	1.19	2.47387E-06
P55884	Eukaryotic translation initiation factor 3 subunit B OS=Homo sapiens OX=9606 GN=EIF3B PE=1 SV=3	31	257	20	6.42E+07	5.92E+07	6.81E+07	5.85E+07	6.40E+07	5.65E+07	5.34E+07	4.28E+07	4.26E+07	3.50E+07	4.06E+07	3.51E+07	6.18E+07	4.16E+07	1.48	0.57	0.000213992
Q14697	Neutral alpha-glucosidase AB OS=Homo sapiens OX=9606 GN=GANAB PE=1 SV=3	29	265	20	7.86E+07	7.47E+07	7.10E+07	7.26E+07	7.27E+07	7.55E+07	6.37E+07	6.30E+07	4.71E+07	4.76E+07	4.87E+07	4.74E+07	7.42E+07	5.29E+07	1.40	0.49	0.000834923
P50990	T-complex protein 1 subunit theta OS=Homo sapiens OX=9606 GN=CCT8 PE=1 SV=4	44	531	20	1.82E+08	1.90E+08	1.76E+08	1.77E+08	1.81E+08	1.62E+08	1.21E+08	1.33E+08	1.31E+08	1.18E+08	1.09E+08	1.20E+08	1.78E+08	1.22E+08	1.46	0.55	8.70307E-07
P23526	Adenosylhomocysteinase OS=Homo sapiens OX=9606 GN=AHCY PE=1 SV=4	45	1122	21	1.03E+09	1.07E+09	1.24E+09	1.24E+09	1.22E+09	1.09E+09	1.50E+09	1.36E+09	1.58E+09	1.56E+09	1.49E+09	1.26E+09	1.15E+09	1.46E+09	0.79	-0.35	0.000722513
Q13200	26S proteasome non-ATPase regulatory subunit 2 OS=Homo sapiens OX=9606 GN=PSMD2 PE=1 SV=3	33	309	21	1.01E+08	9.62E+07	8.49E+07	9.35E+07	9.36E+07	8.54E+07	6.72E+07	6.70E+07	6.32E+07	5.90E+07	5.91E+07	4.67E+07	9.25E+07	6.04E+07	1.53	0.61	1.49926E-05
P12956	X-ray repair cross-complementing protein 6 OS=Homo sapiens OX=9606 GN=XRCC6 PE=1 SV=2	43	450	21	1.02E+08	1.06E+08	1.08E+08	9.56E+07	1.07E+08	9.65E+07	9.81E+07	9.57E+07	1.09E+08	8.10E+07	9.81E+07	7.80E+07	1.03E+08	9.34E+07	1.10	0.13	0.126294519
P62701	40S ribosomal protein S4, X isoform OS=Homo sapiens OX=9606 GN=RPS4X PE=1 SV=2	59	446	21	1.96E+08	2.05E+08	1.96E+08	2.16E+08	1.95E+08	1.84E+08	1.58E+08	1.59E+08	1.59E+08	1.58E+08	1.51E+08	1.27E+08	1.99E+08	1.52E+08	1.31	0.39	5.18458E-05
Q9Y262	Eukaryotic translation initiation factor 3 subunit L OS=Homo sapiens OX=9606 GN=EIF3L PE=1 SV=1	43	323	21	8.32E+07	9.09E+07	9.52E+07	7.49E+07	8.57E+07	7.17E+07	5.98E+07	5.83E+07	5.63E+07	5.73E+07	5.41E+07	4.42E+07	8.36E+07	5.50E+07	1.52	0.60	0.000144126

Supplementary Table 4. List of all proteins identified by label free proteomics analysis.

Accession	Description	Coverage [%]	# PSMs	# Unique Peptides	Amitriptyline 1	Amitriptyline 2	Amitriptyline 3	Amitriptyline 4	Amitriptyline 5	Amitriptyline 6	Vehicle 1	Vehicle 2	Vehicle 3	Vehicle 4	Vehicle 5	Vehicle 6	Average amitriptyline	Average vehicle	Ratio amitriptyline/ vehicle	Log 2 fold change (amitriptyline /vehicle)	P-value
Q86VP6	Cullin-associated NEDD8-dissociated protein 1 OS=Homo sapiens OX=9606 GN=CAND1 PE=1 SV=2	24	316	21	9.30E+07	9.16E+07	8.64E+07	8.76E+07	8.37E+07	8.43E+07	9.53E+07	8.02E+07	6.86E+07	7.16E+07	6.46E+07	7.08E+07	8.78E+07	7.52E+07	1.17	0.22	0.038356109
P36871	Phosphoglucomutase-1 OS=Homo sapiens OX=9606 GN=PGM1 PE=1 SV=3	50	249	21	1.18E+08	1.10E+08	1.18E+08	1.26E+08	1.25E+08	1.14E+08	1.57E+08	1.57E+08	1.62E+08	1.49E+08	1.37E+08	1.05E+08	1.18E+08	1.45E+08	0.82	-0.29	0.027021135
P08473	Neprilysin OS=Homo sapiens OX=9606 GN=MME PE=1 SV=2	40	278	21	7.90E+07	7.76E+07	9.87E+07	8.40E+07	9.29E+07	8.00E+07	6.14E+07	5.84E+07	7.07E+07	8.06E+07	5.84E+07	4.12E+07	8.54E+07	6.18E+07	1.38	0.47	0.005749762
Q9P2J5	Leucine--tRNA ligase, cytoplasmic OS=Homo sapiens OX=9606 GN=LARS PE=1 SV=2	23	258	21	7.95E+07	7.32E+07	7.08E+07	6.19E+07	7.17E+07	6.54E+07	5.73E+07	3.84E+07	4.38E+07	4.23E+07	4.18E+07	3.68E+07	7.04E+07	4.34E+07	1.62	0.70	4.74058E-05
Q5T4S7	E3 ubiquitin-protein ligase UBR4 OS=Homo sapiens OX=9606 GN=UBR4 PE=1 SV=1	7	102	21	2.18E+07	1.92E+07	2.20E+07	2.17E+07	2.23E+07	2.09E+07	1.63E+07	1.05E+07	1.34E+07	1.19E+07	1.42E+07	1.15E+07	2.13E+07	1.30E+07	1.65	0.72	3.28881E-05
P23396	40S ribosomal protein S3 OS=Homo sapiens OX=9606 GN=RPS3 PE=1 SV=2	78	566	21	1.71E+08	1.81E+08	1.66E+08	1.47E+08	1.63E+08	1.46E+08	1.25E+08	1.34E+08	1.24E+08	1.16E+08	1.21E+08	1.01E+08	1.62E+08	1.20E+08	1.35	0.43	0.000208221
Q15029	116 kDa U5 small nuclear ribonucleoprotein component OS=Homo sapiens OX=9606 GN=EFTUD2 PE=1 SV=1	33	201	21	5.50E+07	5.46E+07	5.28E+07	5.45E+07	5.85E+07	5.42E+07	3.82E+07	3.65E+07	3.86E+07	3.87E+07	4.25E+07	3.41E+07	5.49E+07	3.81E+07	1.44	0.53	7.64206E-07
P08238	Heat shock protein HSP 90-beta OS=Homo sapiens OX=9606 GN=HSP90AB1 PE=1 SV=4	52	1866	22	8.71E+08	9.37E+08	8.56E+08	8.81E+08	8.96E+08	8.14E+08	8.91E+08	9.15E+08	8.41E+08	8.06E+08	8.12E+08	7.02E+08	8.76E+08	8.28E+08	1.06	0.08	0.206432937
P06737	Glycogen phosphorylase, liver form OS=Homo sapiens OX=9606 GN=PYGL PE=1 SV=4	40	787	22	2.48E+08	2.43E+08	2.23E+08	2.23E+08	2.25E+08	2.26E+08	2.52E+08	2.53E+08	2.43E+08	2.24E+08	2.33E+08	1.95E+08	2.31E+08	2.33E+08	0.99	-0.01	0.834485045
P21399	Cytoplasmic aconitate hydratase OS=Homo sapiens OX=9606 GN=ACO1 PE=1 SV=3	34	668	22	2.47E+08	2.44E+08	2.31E+08	7.36E+08	2.15E+08	2.33E+08	8.52E+08	3.18E+08	2.68E+08	2.66E+08	2.61E+08	2.51E+08	3.18E+08	3.69E+08	0.86	-0.22	0.694913113
P05023	Sodium/potassium-transporting ATPase subunit alpha-1 OS=Homo sapiens OX=9606 GN=ATP1A1 PE=1 SV=1	26	335	22	1.31E+08	1.28E+08	1.21E+08	1.26E+08	1.24E+08	1.16E+08	1.02E+08	1.04E+08	9.44E+07	8.91E+07	8.88E+07	7.44E+07	1.24E+08	9.21E+07	1.35	0.43	0.000237934
P35606	Coatomer subunit beta' OS=Homo sapiens OX=9606 GN=COPB2 PE=1 SV=2	38	231	22	6.22E+07	7.29E+07	5.98E+07	6.90E+07	6.16E+07	6.42E+07	3.66E+07	3.92E+07	3.39E+07	3.69E+07	4.04E+07	3.17E+07	6.50E+07	3.64E+07	1.78	0.83	1.50556E-06
P04406	Glyceraldehyde-3-phosphate dehydrogenase OS=Homo sapiens OX=9606 GN=GAPDH PE=1 SV=3	82	4882	23	1.82E+09	1.71E+09	1.73E+09	1.64E+09	1.81E+09	1.91E+09	1.69E+09	1.72E+09	1.61E+09	1.60E+09	1.59E+09	1.55E+09	1.77E+09	1.63E+09	1.09	0.12	0.013562483
P07900	Heat shock protein HSP 90-alpha OS=Homo sapiens OX=9606 GN=HSP90AA1 PE=1 SV=5	50	1956	23	3.02E+08	2.93E+08	3.25E+08	3.36E+08	3.11E+08	2.85E+08	3.04E+08	3.40E+08	3.59E+08	3.17E+08	3.37E+08	2.82E+08	3.09E+08	3.23E+08	0.96	-0.07	0.322908266
P08195	4F2 cell-surface antigen heavy chain OS=Homo sapiens OX=9606 GN=SLC3A2 PE=1 SV=3	40	505	23	2.10E+08	2.14E+08	1.84E+08	1.79E+08	1.76E+08	1.88E+08	8.27E+07	8.29E+07	5.68E+07	6.45E+07	7.38E+07	6.18E+07	1.92E+08	7.04E+07	2.73	1.45	1.18049E-07
Q9Y5B9	FACT complex subunit SPT16 OS=Homo sapiens OX=9606 GN=SUPT16H PE=1 SV=1	29	295	23	7.51E+07	8.30E+07	8.24E+07	8.02E+07	6.67E+07	7.82E+07	3.79E+07	4.55E+07	3.80E+07	4.33E+07	3.80E+07	3.57E+07	7.76E+07	3.97E+07	1.95	0.97	7.78542E-07
Q15149	Plectin OS=Homo sapiens OX=9606 GN=PLEC PE=1 SV=3	7	143	23	3.00E+07	3.30E+07	3.08E+07	3.88E+07	3.33E+07	3.19E+07	2.19E+07	1.83E+07	2.27E+07	2.31E+07	2.02E+07	1.84E+07	3.30E+07	2.08E+07	1.59	0.67	2.85463E-05
P06733	Alpha-enolase OS=Homo sapiens OX=9606 GN=ENO1 PE=1 SV=2	72	1037	24	5.26E+08	6.02E+08	4.50E+08	4.65E+08	4.48E+08	5.30E+08	5.68E+08	6.82E+08	4.69E+08	5.25E+08	5.39E+08	5.18E+08	5.04E+08	5.50E+08	0.92	-0.13	0.253643333
P48643	T-complex protein 1 subunit epsilon OS=Homo sapiens OX=9606 GN=CCT5 PE=1 SV=1	56	678	24	2.22E+08	2.23E+08	1.91E+08	2.00E+08	1.96E+08	2.06E+08	1.47E+08	1.56E+08	1.40E+08	1.26E+08	1.33E+08	1.09E+08	2.06E+08	1.35E+08	1.53	0.61	1.24491E-05
P50991	T-complex protein 1 subunit delta OS=Homo sapiens OX=9606 GN=CCT4 PE=1 SV=4	51	660	24	2.95E+08	2.90E+08	2.69E+08	2.72E+08	2.43E+08	2.83E+08	1.98E+08	1.90E+08	1.63E+08	1.81E+08	1.71E+08	1.57E+08	2.75E+08	1.77E+08	1.56	0.64	2.15189E-06
P19338	Nucleolin OS=Homo sapiens OX=9606 GN=NCL PE=1 SV=3	33	322	24	9.01E+07	8.93E+07	9.76E+07	1.31E+08	9.84E+07	1.05E+08	6.56E+07	6.26E+07	6.58E+07	5.77E+07	6.44E+07	5.27E+07	1.02E+08	6.15E+07	1.66	0.73	0.000859936
P08758	Annexin A5 OS=Homo sapiens OX=9606 GN=ANXA5 PE=1 SV=2	80	991	25	5.08E+08	4.55E+08	4.84E+08	4.77E+08	5.04E+08	4.41E+08	3.91E+08	3.55E+08	3.40E+08	3.38E+08	3.74E+08	2.94E+08	4.78E+08	3.49E+08	1.37	0.46	3.00974E-05
P78371	T-complex protein 1 subunit beta OS=Homo sapiens OX=9606 GN=CCT2 PE=1 SV=4	61	555	25	2.83E+08	2.85E+08	2.43E+08	2.51E+08	2.35E+08	2.30E+08	1.92E+08	2.02E+08	1.60E+08	1.69E+08	1.84E+08	1.51E+08	2.55E+08	1.76E+08	1.44	0.53	0.000124783
P11142	Heat shock cognate 71 kDa protein OS=Homo sapiens OX=9606 GN=HSPA8 PE=1 SV=1	66	2437	26	9.83E+08	9.82E+08	9.35E+08	1.01E+09	9.65E+08	9.54E+08	9.54E+08	1.01E+09	9.02E+08	9.39E+08	8.91E+08	8.46E+08	9.72E+08	9.23E+08	1.05	0.07	0.093247678
P40227	T-complex protein 1 subunit zeta OS=Homo sapiens OX=9606 GN=CCT6A PE=1 SV=3	53	709	26	2.97E+08	2.95E+08	2.92E+08	3.03E+08	3.06E+08	2.83E+08	2.21E+08	2.17E+08	2.05E+08	1.92E+08	2.01E+08	1.77E+08	2.96E+08	2.02E+08	1.46	0.55	3.16712E-06
P13010	X-ray repair cross-complementing protein 5 OS=Homo sapiens OX=9606 GN=XRCC5 PE=1 SV=3	53	374	26	1.10E+08	1.14E+08	1.09E+08	1.01E+08	1.12E+08	1.13E+08	7.94E+07	8.32E+07	7.40E+07	7.05E+07	6.78E+07	6.29E+07	1.10E+08	7.30E+07	1.51	0.59	4.82714E-06
P17987	T-complex protein 1 subunit alpha OS=Homo sapiens OX=9606 GN=TCP1 PE=1 SV=1	66	692	26	2.89E+08	3.16E+08	3.03E+08	2.56E+08	2.93E+08	2.80E+08	2.04E+08	2.37E+08	1.97E+08	2.10E+08	2.14E+08	1.72E+08	2.90E+08	2.05E+08	1.41	0.50	3.73317E-05
Q8WUM4	Programmed cell death 6-interacting protein OS=Homo sapiens OX=9606 GN=PDCD6IP PE=1 SV=1	41	379	26	8.91E+07	9.65E+07	9.81E+07	9.75E+07	9.05E+07	9.14E+07	8.57E+07	7.93E+07	8.23E+07	8.04E+07	8.38E+07	7.47E+07	9.38E+07	8.10E+07	1.16	0.21	0.000202288
Q14152	Eukaryotic translation initiation factor 3 subunit A OS=Homo sapiens OX=9606 GN=EIF3A PE=1 SV=1	21	374	26	1.17E+08	1.15E+08	1.11E+08	1.12E+08	8.94E+07	1.05E+08	6.75E+07	6.08E+07	6.15E+07	6.26E+07	6.03E+07	5.66E+07	1.08E+08	6.15E+07	1.76	0.82	3.26269E-05
P02679	Fibrinogen gamma chain OS=Homo sapiens OX=9606 GN=FGG PE=1 SV=3	67	603	27	6.97E+07	6.14E+07	6.84E+07	6.97E+07	6.63E+07	7.04E+07	3.38E+08	1.48E+08	9.16E+07	8.50E+07	7.93E+07	3.84E+09	6.77E+07	7.64E+08	0.09	-3.50	0.310239774
P0DMV9	Heat shock 70 kDa protein 1B OS=Homo sapiens OX=9606 GN=HSPA1B PE=1 SV=1	63	974	27	2.82E+08	2.89E+08	2.39E+08	2.48E+08	2.24E+08	2.51E+08	2.79E+08	2.98E+08	2.46E+08	2.28E+08	2.38E+08	2.05E+08	2.55E+08	2.49E+08	1.03	0.04	0.712895103
P49368	T-complex protein 1 subunit gamma OS=Homo sapiens OX=9606 GN=CCT3 PE=1 SV=4	54	599	27	2.47E+08	2.40E+08	1.93E+08	1.83E+08	1.84E+08	2.15E+08	1.59E+08	1.58E+08	1.24E+08	1.40E+08	1.33E+08	1.34E+08	2.10E+08	1.41E+08	1.49	0.57	0.0008459
P31939	Bifunctional purine biosynthesis protein PURH OS=Homo sapiens OX=9606 GN=ATIC PE=1 SV=3	57	530	27	1.55E+08	1.54E+08	1.45E+08	1.47E+08	1.54E+08	1.35E+08	1.82E+08	1.85E+08	1.57E+08	1.53E+08	1.56E+08	1.37E+08	1.48E+08	1.62E+08	0.92	-0.12	0.151560445
Q99832	T-complex protein 1 subunit eta OS=Homo sapiens OX=9606 GN=CCT7 PE=1 SV=2	61	772	28	3.51E+08	3.48E+08	3.08E+08	3.07E+08	3.34E+08	3.28E+08	2.33E+08	2.52E+08	2.20E+08	2.33E+08	2.34E+08	2.02E+08	3.29E+08	2.29E+08	1.44	0.52	2.22104E-06
P05556	Integrin beta-1 OS=Homo sapiens OX=9606 GN=ITGB1 PE=1 SV=2	38	775	28	3.82E+08	3.88E+08	3.73E+08	3.77E+08	3.64E+08	3.64E+08	2.69E+08	2.76E+08	2.38E+08	2.28E+08	2.21E+08	2.18E+08	3.75E+08	2.42E+08	1.55	0.63	1.13194E-05
P11586	C-1-tetrahydrofolate synthase, cytoplasmic OS=Homo sapiens OX=9606 GN=MTHFD1 PE=1 SV=3	36	445	28	1.37E+08	1.40E+08	1.31E+08	1.33E+08	1.21E+08	1.23E+08	1.23E+08	1.30E+08	1.31E+08	1.25E+08	1.09E+08	1.22E+08	1.31E+08	1.23E+08	1.06	0.08	0.123660787
P09874	Poly [ADP-ribose] polymerase 1 OS=Homo sapiens OX=9606 GN=PARP1 PE=1 SV=4	40	499	30	2.05E+08	1.95E+08	2.07E+08	2.09E+08	2.01E+08	1.95E+08	1.08E+08	1.04E+08	9.42E+07	9.77E+07	8.86E+07	8.98E+07	2.02E+08	9.70E+07	2.08	1.06	5.55454E-10
P53621	Coatomer subunit alpha OS=Homo sapiens OX=9606 GN=COPA PE=1 SV=2	34	420	30	2.86E+08	2.80E+08	3.24E+08	4.11E+08	4.45E+08	3.24E+08	3.67E+08	3.78E+08	4.07E+08	5.65E+08	5.21E+08	3.01E+08	3.45E+08	4.23E+08	0.82	-0.29	0.148602083
P41252	Isoleucine--tRNA ligase, cytoplasmic OS=Homo sapiens OX=9606 GN=IARS PE=1 SV=2	32	497	30	1.33E+08	1.45E+08	1.15E+08	1.29E+08	1.26E+08	1.26E+08	8.39E+07	7.45E+07	7.54E+07	7.08E+07	7.22E+07	7.20E+07	1.29E+08	7.48E+07	1.72	0.79	4.89517E-06
P98160	Basement membrane-specific heparan sulfate proteoglycan core protein OS=Homo sapiens OX=9606 GN=HSPG2 PE=1 SV=4	10	269	30	9.29E+07	6.69E+07	5.83E+07	6.04E+07	5.34E+07	6.05E+07	9.00E+07	8.58E+07	5.08E+07	5.65E+07	5.69E+07	6.93E+07	6.54E+07	6.82E+07	0.96	-0.06	0.758444528
Q6P2Q9	Pre-mRNA-processing-splicing factor 8 OS=Homo sapiens OX=9606 GN=PRPF8 PE=1 SV=2	19	225	30	6.08E+07	5.59E+07	5.37E+07	4.66E+07	5.16E+07	5.28E+07	3.53E+07	2.13E+07	2.35E+07	3.38E+07	2.73E+07	2.22E+07	5.36E+07	2.72E+07	1.97	0.98	1.10292E-05
P35908	Keratin, type II cytoskeletal 2 epidermal OS=Homo sapiens OX=9606 GN=KRT2 PE=1 SV=2	70	1456	31	4.85E+08	1.21E+09	2.35E+08	3.33E+08	4.06E+08	4.98E+08	1.23E+08	6.51E+08	2.20E+08	2.93E+08	3.74E+08	2.68E+08	5.28E+08	3.22E+08	1.64	0.72	0.236554245
Q08211	ATP-dependent RNA helicase A OS=Homo sapiens OX=9606 GN=DHX9 PE=1 SV=4	34	577	31	2.17E+08	2.10E+08	2.16E+08	2.24E+08	2.19E+08	2.16E+08	1.81E+08	1.85E+08	1.48E+08	1.65E+08	1.63E+08	1.49E+08	2.17E+08	1.65E+08	1.31	0.39	0.000259641
O00159	Unconventional myosin-Ic OS=Homo sapiens OX=9606 GN=MYO1C PE=1 SV=4	41	488	32	1.18E+08	1.07E+08	8.96E+07	8.97E+07	9.79E+07	9.14E+07	8.50E+07	8.28E+07	8.19E+07	7.51E+07	7.66E+07	6.03E+07	9.90E+07	7.70E+07	1.29	0.36	0.004669494
P13645	Keratin, type I cytoskeletal 10 OS=Homo sapiens OX=9606 GN=KRT10 PE=1 SV=6	58	1611	33	1.34E+09	2.38E+09	5.96E+08	8.69E+08	1.02E+09	1.21E+09	2.75E+08	1.47E+09	5.68E+08	7.63E+08	9.44E+08	6.51E+08	1.24E+09	7.78E+08	1.59	0.67	0.165045892
P02671	Fibrinogen alpha chain OS=Homo sapiens OX=9606 GN=FGA PE=1 SV=2	39	719	33	1.54E+07	1.65E+07	1.29E+07	1.45E+07	9.89E+06	1.58E+07	2.67E+08	1.03E+08	6.26E+07	6.13E+07	3.80E+07	3.26E+09	1.42E+07	6.32E+08	0.02	-5.48	0.293603515
P53396	ATP-citrate synthase OS=Homo sapiens OX=9606 GN=ACLY PE=1 SV=3	36	770	33	2.01E+08	2.03E+08	1.84E+08	1.69E+08	1.68E+08	1.95E+08	1.73E+08	1.85E+08	1.55E+08	1.62E+08	1.68E+08	1.55E+08	1.87E+08	1.66E+08	1.12	0.17	0.029325133
P35527	Keratin, type I cytoskeletal 9 OS=Homo sapiens OX=9606 GN=KRT9 PE=1 SV=3	69	1049	34	7.49E+08	7.17E+08	3.45E+08	7.96E+08	6.78E+08	1.27E+09	3.70E+08	9.10E+08	3.49E+08	6.27E+08	8.38E+08	6.80E+08	7.59E+08	6.29E+08	1.21	0.27	0.419000351
Q14764	Major vault protein OS=Homo sapiens OX=9606 GN=MVP PE=1 SV=4	57	669	35	2.44E+08	2.41E+08	2.58E+08	2.62E+08	2.59E+08	2.45E+08	2.16E+08	2.31E+08	2.27E+08	2.00E+08	2.21E+08	1.74E+08	2.51E+08	2.12E+08	1.19	0.25	0.004458123
Q6UVK1	Chondroitin sulfate proteoglycan 4 OS=Homo sapiens OX=9606 GN=CSPG4 PE=1 SV=2	24	263	36	8.48E+07	8.39E+07	7.44E+07	7.44E+07	7.14E+07	7.15E+07	3.34E+07	3.14E+07	2.91E+07	2.56E+07	2.89E+07	2.61E+07	7.67E+07	2.91E+07	2.64	1.40	3.46182E-07
P04264	Keratin, type II cytoskeletal 1 OS=Homo sapiens OX=9606 GN=KRT1 PE=1 SV=6	62	2340	37	2.47E+09	2.77E+09	1.26E+09	2.08E+09	2.08E+09	3.06E+09	1.37E+09	2.81E+09	1.64E+09	2.06E+09	2.39E+09	1.85E+09	2.29E+09	2.02E+09	1.13	0.18	0.446670947
P55072	Transitional endoplasmic reticulum ATPase OS=Homo sapiens OX=9606 GN=VCP PE=1 SV=4	54	900	37	3.83E+08	3.70E+08	3.19E+08	3.25E+08	3.09E+08	3.69E+08	4.46E+08	3.92E+08	2.78E+08	2.68E+08	3.57E+08	3.22E+08	3.46E+08	3.44E+08	1.01	0.01	0.955918535
P46940	Ras GTPase-activating-like protein IQGAP1 OS=Homo sapiens OX=9606 GN=IQGAP1 PE=1 SV=1	35	378	37	8.21E+07	9.03E+07	8.61E+07	8.35E+07	7.60E+07	8.05E+07	7.51E+07	6.36E+07	6.87E+07	5.56E+07	6.55E+07	6.20E+07	8.31E+07	6.51E+07	1.28	0.35	0.000410721
P07355	Annexin A2 OS=Homo sapiens OX=9606 GN=ANXA2 PE=1 SV=2	78	1569	38	1.08E+09	1.05E+09	1.09E+09	9.28E+08	9.95E+08	1.02E+09	8.68E+08	8.87E+08	1.01E+09	8.21E+08	8.89E+08	7.72E+08	1.03E+09	8.74E+08	1.17	0.23	0.004093417
P22314	Ubiquitin-like modifier-activating enzyme 1 OS=Homo sapiens OX=9606 GN=UBA1 PE=1 SV=3	52	1085	40	3.25E+08	3.25E+08	2.84E+08	2.99E+08	2.72E+08	3.07E+08	3.14E+08	3.12E+08	2.71E+08	2.54E+08	2.79E+08	2.60E+08	3.02E+08	2.82E+08	1.07	0.10	0.168276897
P07996	Thrombospondin-1 OS=Homo sapiens OX=9606 GN=THBS1 PE=1 SV=2	45	1374	41	4.14E+08	4.27E+08	4.35E+08	4.16E+08	3.86E+08	4.18E+08	6.52E+08	6.48E+08	6.56E+08	6.24E+08	6.06E+08	5.96E+08	4.16E+08	6.31E+08	0.66	-0.60	5.27845E-08
P07814	Bifunctional glutamate/proline--tRNA ligase OS=Homo sapiens OX=9606 GN=EPRS PE=1 SV=5	40	562	41	1.54E+08	1.45E+08	1.40E+08	1.58E+08	1.41E+08	1.47E+08	9.20E+07	8.36E+07	8.59E+07	8.29E+07	7.67E+07	7.46E+07	1.47E+08	8.26E+07	1.78	0.84	1.67041E-08
P01024	Complement C3 OS=Homo sapiens OX=9606 GN=C3 PE=1 SV=2	31	5444	42	3.85E+09	4.06E+09	4.88E+09	4.75E+09	4.91E+09	4.65E+09	5.43E+09	5.29E+09	6.73E+09	5.99E+09	7.00E+09	5.55E+09	4.52E+09	6.00E+09	0.75	-0.41	0.002371545
O75643	U5 small nuclear ribonucleoprotein 200 kDa helicase OS=Homo sapiens OX=9606 GN=SNRNP200 PE=1 SV=2	29	405	42	9.26E+07	9.55E+07	8.26E+07	8.89E+07	8.61E+07	9.39E+07	6.74E+07	6.62E+07	5.79E+07	5.88E+07	5.47E+07	5.32E+07	8.99E+07	5.97E+07	1.51	0.59	2.77894E-06
P14618	Pyruvate kinase PKM OS=Homo sapiens OX=9606 GN=PKM PE=1 SV=4	76	2764	43	1.42E+09	1.58E+09	1.26E+09	1.33E+09	1.33E+09	1.49E+09	1.38E+09	1.56E+09	1.23E+09	1.27E+09	1.35E+09	1.24E+09	1.40E+09	1.34E+09	1.05	0.07	0.39160608
P02675	Fibrinogen beta chain OS=Homo sapiens OX=9606 GN=FGB PE=1 SV=2	76	920	44	2.55E+08	2.49E+08	2.60E+08	2.65E+08	2.44E+08	2.23E+08	6.61E+08	4.18E+08	3.60E+08	4.02E+08	2.81E+08	5.15E+09	2.49E+08	1.21E+09	0.21	-2.28	0.276911003
P13639	Elongation factor 2 OS=Homo sapiens OX=9606 GN=EEF2 PE=1 SV=4	58	1610	45	7.71E+08	6.92E+08	7.21E+08	6.51E+08	6.65E+08	7.04E+08	7.10E+08	6.66E+08	6.21E+08	6.33E+08	6.38E+08	5.61E+08	7.01E+08	6.38E+08	1.10	0.13	0.042285128
Q07954	Prolow-density lipoprotein receptor-related protein 1 OS=Homo sapiens OX=9606 GN=LRP1 PE=1 SV=2	14	685	45	1.43E+08	1.50E+08	1.33E+08	1.44E+08	1.34E+08	1.43E+08	1.95E+08	1.97E+08	1.70E+08	1.80E+08	1.77E+08	1.55E+08	1.41E+08	1.79E+08	0.79	-0.34	0.001256892
P08133	Annexin A6 OS=Homo sapiens OX=9606 GN=ANXA6 PE=1 SV=3	73	1213	48	4.77E+08	5.03E+08	4.71E+08	4.95E+08	4.79E+08	4.74E+08	3.95E+08	3.94E+08	3.68E+08	3.41E+08	3.85E+08	3.23E+08	4.83E+08	3.68E+08	1.31	0.39	6.75775E-05
P78527	DNA-dependent protein kinase catalytic subunit OS=Homo sapiens OX=9606 GN=PRKDC PE=1 SV=3	17	505	49	1.14E+08	1.22E+08	9.54E+07	1.17E+08	1.14E+08	1.03E+08	8.25E+07	7.09E+07	6.37E+07	7.06E+07	6.91E+07	5.91E+07	1.11E+08	6.93E+07	1.60	0.68	1.50396E-05
O75369	Filamin-B OS=Homo sapiens OX=9606 GN=FLNB PE=1 SV=2	37	870	54	1.92E+08	1.90E+08	1.68E+08	1.85E+08	1.59E+08	1.77E+08	1.44E+08	1.51E+08	1.33E+08	1.23E+08	1.23E+08	1.09E+08	1.78E+08	1.31E+08	1.37	0.45	0.000201024
Q14315	Filamin-C OS=Homo sapiens OX=9606 GN=FLNC PE=1 SV=3	33	902	55	2.18E+08	2.18E+08	2.05E+08	2.11E+08	1.97E+08	2.09E+08	1.50E+08	1.43E+08	1.36E+08	1.36E+08	1.24E+08	1.16E+08	2.10E+08	1.34E+08	1.56	0.64	9.32938E-07
P49327	Fatty acid synthase OS=Homo sapiens OX=9606 GN=FASN PE=1 SV=3	45	1668	80	6.59E+08	6.70E+08	6.05E+08	6.16E+08	6.02E+08	6.00E+08	5.70E+08	5.93E+08	4.93E+08	5.00E+08	5.19E+08	4.17E+08	6.25E+08	5.15E+08	1.21	0.28	0.00569581
P35579	Myosin-9 OS=Homo sapiens OX=9606 GN=MYH9 PE=1 SV=4	53	2773	89	8.51E+08	8.59E+08	8.02E+08	8.28E+08	7.72E+08	8.64E+08	9.69E+08	9.69E+08	8.65E+08	9.07E+08	9.15E+08	9.07E+08	8.29E+08	9.22E+08	0.90	-0.15	0.001936962
Q9Y490	Talin-1 OS=Homo sapiens OX=9606 GN=TLN1 PE=1 SV=3	60	3010	94	1.31E+09	1.27E+09	1.14E+09	1.16E+09	1.17E+09	1.22E+09	1.49E+09	1.58E+09	1.38E+09	1.36E+09	1.37E+09	1.34E+09	1.21E+09	1.42E+09	0.85	-0.23	0.001995176
Q00610	Clathrin heavy chain 1 OS=Homo sapiens OX=9606 GN=CLTC PE=1 SV=5	62	2680	96	1.08E+09	1.10E+09	1.07E+09	1.01E+09	1.05E+09	1.04E+09	7.58E+08	7.68E+08	7.06E+08	6.83E+08	7.89E+08	6.20E+08	1.06E+09	7.20E+08	1.47	0.56	3.84425E-06
P02751	Fibronectin OS=Homo sapiens OX=9606 GN=FN1 PE=1 SV=5	57	5321	97	2.38E+09	2.53E+09	2.46E+09	2.37E+09	2.31E+09	2.33E+09	3.82E+09	3.94E+09	3.35E+09	3.33E+09	3.23E+09	5.70E+09	2.40E+09	3.89E+09	0.62	-0.70	0.010667989
P21333	Filamin-A OS=Homo sapiens OX=9606 GN=FLNA PE=1 SV=4	61	3031	105	1.45E+09	1.46E+09	1.39E+09	1.42E+09	1.34E+09	1.40E+09	1.06E+09	1.10E+09	9.80E+08	9.63E+08	8.95E+08	9.30E+08	1.41E+09	9.88E+08	1.43	0.51	3.16207E-06
Q14204	Cytoplasmic dynein 1 heavy chain 1 OS=Homo sapiens OX=9606 GN=DYNC1H1 PE=1 SV=5	35	1951	127	5.89E+08	5.63E+08	5.49E+08	5.70E+08	5.52E+08	5.19E+08	5.43E+08	4.82E+08	4.66E+08	4.44E+08	4.72E+08	3.93E+08	5.57E+08	4.67E+08	1.19	0.26	0.00454779
